# Bacteria Cultivated From Sponges and Bacteria Not Yet Cultivated From Sponges—A Review

**DOI:** 10.3389/fmicb.2021.737925

**Published:** 2021-11-10

**Authors:** Ton That Huu Dat, Georg Steinert, Nguyen Thi Kim Cuc, Hauke Smidt, Detmer Sipkema

**Affiliations:** ^1^Mientrung Institute for Scientific Research, Vietnam Academy of Science and Technology, Hanoi, Vietnam; ^2^Laboratory of Microbiology, Wageningen University & Research, Wageningen, Netherlands; ^3^Institute of Marine Biochemistry, Vietnam Academy of Science and Technology, Hanoi, Vietnam

**Keywords:** sponge-associated bacteria, cultivation, cultivable bacteria, sponge-specific bacteria, sponge-enriched bacteria

## Abstract

The application of high-throughput microbial community profiling as well as “omics” approaches unveiled high diversity and host-specificity of bacteria associated with marine sponges, which are renowned for their wide range of bioactive natural products. However, exploration and exploitation of bioactive compounds from sponge-associated bacteria have been limited because the majority of the bacteria remains recalcitrant to cultivation. In this review, we (i) discuss recent/novel cultivation techniques that have been used to isolate sponge-associated bacteria, (ii) provide an overview of bacteria isolated from sponges until 2017 and the associated culture conditions and identify the bacteria not yet cultured from sponges, and (iii) outline promising cultivation strategies for cultivating the uncultivated majority of bacteria from sponges in the future. Despite intensive cultivation attempts, the diversity of bacteria obtained through cultivation remains much lower than that seen through cultivation-independent methods, which is particularly noticeable for those taxa that were previously marked as “sponge-specific” and “sponge-enriched.” This poses an urgent need for more efficient cultivation methods. Refining cultivation media and conditions based on information obtained from metagenomic datasets and cultivation under simulated natural conditions are the most promising strategies to isolate the most wanted sponge-associated bacteria.

## Introduction

As the most ancient of multi-cellular metazoans (Feuda et al., 2017), marine sponges represent an ecologically important and highly diverse component of marine benthic communities (Wulff, 2006; Bell, 2008; Maldonado et al., 2012; De Goeij et al., 2013). While sponges exhibit a relatively simple body plan, the different sponge cell layers (Hentschel et al., 2012) provide unique ecological niches for a wide range of different symbionts, such as archaea, bacteria, and micro- and macro-eukaryotes (Taylor et al., 2007; Duris et al., 2011; Lattig and Martín, 2011; Li et al., 2011; Hentschel et al., 2012; Thomas et al., 2016). Microorganisms can constitute up to one-third of the sponge’s biomass (Hentschel et al., 2006) and perform diverse metabolic functions in the holobiont, including nitrogen, carbon, sulfur, and phosphorus cycling (Zhang et al., 2015; Li et al., 2016; Pita et al., 2018). Sponge-associated microorganisms also produce secondary metabolites that contribute to the defense of the host against predation, fouling, and diseases (Hentschel et al., 2006, 2012; Taylor et al., 2007; Pawlik, 2011).

During the last decade, especially high-throughput sequencing provided a wealth of information with respect to the composition, host specificity and spatio-temporal dynamics of sponge-associated microbial communities (Schmitt et al., 2012; White et al., 2012; Cleary et al., 2013; Steinert et al., 2017; Dat et al., 2018). To date, more than 60 bacterial phyla, including newly discovered candidate phyla that lack any cultured representative, have been reported from sponges (Thomas et al., 2016; Moitinho-Silva et al., 2017; Taylor et al., 2021). Although molecular methods have provided a lot of information about the diversity and composition of bacteria associated with sponges, studies on the cultivation of pure bacterial strains from sponges have not lost their relevance. Phenotypic characteristics can only be comprehensively characterized based on pure cultures. Omics-based approaches (e.g., metagenomics, metatranscriptomics, metaproteomics, and metabolomics) are highly relevant to make predictions of the lifestyle of currently uncultivated bacteria, but bacterial isolates are needed for the verification of such predictions (Gutleben et al., 2018). Furthermore, omics-based approaches have limited predictive power for truly novel or unexpected physiological functions of not-yet-cultured bacteria since functional predictions mainly rely on available well-annotated genomes from cultured microorganisms. Thus, a large fraction of detected genes cannot be unequivocally assigned to any function and/or metabolic pathway (Overmann et al., 2017).

Sponge-associated bacteria are also a prolific and rich source of natural products, which include pharmacologically valuable compounds (Abdelmohsen et al., 2014; Indraningrat et al., 2016; Brinkmann et al., 2017; Fehmida et al., 2017; Zhang et al., 2017). Unfortunately, despite their tremendous pharmaceutical and biotechnological potential, we still have been unable to access most of these secondary metabolites. This is mainly due to lack of cultivation success for most sponge-associated bacteria as only a minor fraction of the bacterial community (0.1–14%) has been successfully cultured in the laboratory (Olson et al., 2000; Webster and Hill, 2001; Sipkema et al., 2011; Fuerst, 2014; Montalvo et al., 2014). Artificial growth media and culture conditions are often not quite right to mimic the natural conditions (e.g., physicochemical properties in the sponge, the interactions of bacteria in a community as well as between bacteria and sponge) required for microbial growth (Alain and Querellou, 2009). The development of novel cultivation techniques based on knowledge about interactions between sponge cells and bacteria (or between different bacteria) can enhance the cultivability of previously uncultured bacteria from sponges as well as stimulate the production of bioactive compounds (Romano et al., 2018).

Previous reviews related to sponges and their symbionts (e.g., Taylor et al., 2007; Hentschel et al., 2012; Simister R.L. et al., 2012; Moitinho-Silva et al., 2017) focus on insights related to diversity, evolution, ecology, biotechnological potential as well as interactions between sponges and their symbionts, whereas a comprehensive review on the cultivability of sponge-associated bacteria is lacking. In this review, we (i) discuss cultivation techniques that have been used to cultivate sponge-associated bacteria, (ii) evaluate and analyze the cultivable bacterial diversity from sponges and the associated culture conditions, and (iii) highlight promising cultivation strategies for cultivating currently uncultivable bacteria from sponges.

## Cultivation Techniques Used for Isolation of Sponge-Associated Bacteria

### Agar Plate-Based Cultivation

The traditional cultivation of bacteria using agar plates was introduced by Robert Koch in 1881, and is still the most popularly used method until today. Consequently, agar plate-based cultivation has also been the most commonly used method for the cultivation of sponge-associated bacteria to date. A wide variety of growth media and culture conditions (e.g., with respect to oxygen, temperature, etc.) have been tested to provide the right growth conditions and enhance the cultivability of sponge-associated bacteria on agar plates (Sipkema et al., 2011; Öztürk et al., 2013; Lavy et al., 2014; Montalvo et al., 2014; Matobole et al., 2017; Indraningrat et al., 2019; Gutleben et al., 2020; Dat et al., 2021). For example, Sipkema et al. (2011) used a diverse set of 19 culture media for cultivating bacteria from *Haliclona* (Gellius) sp. In addition, different supplements (e.g., antibiotics, sponge extracts, siderophores, and bacterial signal molecules) were added to culture media to improve the recovery of bacteria from this sponge. Members of phyla/classes that are generally less or not successfully cultured (e.g., *Deltaproteobacteria*, *Planctomycetes*, *Verrucomicrobia*) were also isolated, and the fraction of cultivable bacteria from the sponge represented 14% of the bacterial species detected by cultivation-independent means. In another study, Lavy et al. (2014) used physiological information of the sponge and genomic information of the associated bacteria to design specific culture media and conditions for bacterial taxa present in the sponge. They isolated 59 Operational Taxonomic Units (OTUs: clusters of 16S rRNA gene sequences within a percent sequence similarity threshold, typically 97%) from the sponge *Theonella swinhoei*, of which 22 OTUs were identified as novel species based on 97% 16S rRNA gene sequence identity. The addition of specific components to culture media can also increase the cultivability of sponge-associated bacteria. For example, the addition of sodium pyruvate, catalase (Olson et al., 2000), alpha-butyrolactone (Selvin et al., 2009), crude sponge extracts (Webster et al., 2001; Sfanos et al., 2005; Selvin et al., 2009; Abdelmohsen et al., 2010, 2014; Sipkema et al., 2011; Zeng et al., 2013; Steinert et al., 2014; Esteves et al., 2016) or sponge skeleton (Kaboré et al., 2019) to culture media enhanced the cultivability of sponge symbionts. Notably, Kaboré et al. (2019) showed that the addition of spongin-based sponge skeleton and autoclaved aqueous filtrate of sponge skeleton to culture media significantly improved the growth of *Gemmata* spp. (*Planctomycetes*). Antibiotics have been often used in media targeting *Actinomycetes* to inhibit the growth of other, fast-growing bacteria (Montalvo et al., 2005, 2014; Li and Liu, 2006; Zhang et al., 2006; Abdelmohsen et al., 2010; Vicente et al., 2013). In addition, the use of a light spectrum and intensity similar to the natural conditions was shown to be important to improve the cultivability of sponge-associated cyanobacteria and other light-harvesting bacteria (Pagliara and Caroppo, 2011; Izumi et al., 2013; Lavy et al., 2014; Keren et al., 2015; Gutleben et al., 2020).

Although the number of studies applying a range of culture media and conditions is still limited (many studies only use media with high organic carbon concentrations), the strategy of using a large variety of media and conditions generally results in improved cultivability of sponge-associated bacteria. In addition, it is believed that the use of a wide range of culture media and conditions may also be beneficial for secondary metabolite production (Bader et al., 2010), and is likely to yield a higher chemical diversity of metabolites (Fuerst, 2014). However, although a diversified cultivation strategy is easy to design, it is laborious and time-consuming, and requires considerable consumables (e.g., media, materials).

### Other Solid Substrates

Apart from traditional cultivation relying on agar, other solid matrix-based approaches have been introduced. For example, Zengler et al. (2002) developed a microcapsule-based cultivation approach to encapsulate single cells in combination with parallel microbial cultivation under low nutrient conditions. Single encapsulated cells that are able to grow under these conditions form microcolonies within the microcapsules (Zengler et al., 2002, 2005). Gerardo Toledo et al. (2012) applied this method to cultivate bacteria from the sponge *Mycale armata*. Furthermore, the isolates were de-replicated via Fourier transform infrared (FT/IR) spectroscopy. This study showed that 42% of the isolated strains obtained by using the microcapsule-based cultivation were novel (<98% 16S rRNA gene identity) as compared to 7% using the traditional agar plate isolation technique (Toledo et al., 2012). Furthermore, the use of FT/IR spectroscopy to de-replicate isolates before identification reduced the time and cost investment to obtain novel isolates.

Floating filter cultivation is an alternative strategy that has been applied for the cultivation of bacteria from sponges. This approach was for the first time used to isolate bacteria from sponges by Sipkema et al. (2011), who used polycarbonate filters floating on top of a liquid medium in order to mimic the inner structures of the filter-feeding sponge. By applying this approach, Sipkema et al. (2011) recovered 23 OTUs that were neither isolated on agar plates nor in liquid cultures. Esteves et al. (2016) also used this approach for the cultivation of bacteria from *Cymbastela concentrica* and *Scopalina* sp. Although low bacterial numbers and richness were observed on the floating filter, the study identified three floating filter-specific OTUs (Esteves et al., 2016).

### Liquid Cultures

Although agar-based cultivation is popular to recover bacterial isolates, many “interesting” bacteria seem incapable of growing at the solid-air interface. Therefore, liquid culture techniques are commonly used as an alternative approach to cultivate bacteria. In addition, liquid cultures may be more effective than agar-based cultures for the cultivation of abundant, but slow-growing bacteria from environmental samples (Reinhold et al., 1988; Cartwright et al., 1994; Kataoka et al., 1996). To date, liquid cultivation has been successfully used as an enrichment technique for isolation of the most abundant seawater bacterium, *Pelagibacter ubique*, as well as anaerobic bacteria and photosynthetic bacteria (Beck, 1971; van Niel, 1971; Connon and Giovannoni, 2002; Takenaka et al., 2013; Ňancucheo et al., 2016; Vekeman et al., 2017).

Liquid cultures have been applied in several studies aiming at the cultivation of sponge symbionts (Ishikawa et al., 2003; Huang et al., 2011; Sipkema et al., 2011; Izumi et al., 2013; Öztürk et al., 2013; Esteves et al., 2016). Sipkema et al. (2011) used liquid cultures to isolate bacteria from the sponge *Haliclona* (Gellius) sp. and recovered 10 OTUs that were exclusively obtained from liquid cultures and not on agar-based media or floating filters. In another study, Izumi et al. (2013) used liquid culture to enrich and isolate members of the phylum *Planctomycetes* from the sponge *Niphates* sp. Liquid enrichment cultivation led to the isolation of 17 *Planctomycetes* strains, including novel lineages (<91% 16S rRNA gene sequence identity). Ishikawa et al. (2003) isolated six novel halophilic and alkaliphilic lactic acid bacterial strains (*Marinilactibacillus psychrotolerans*) from an unidentified sponge using liquid enrichment cultures. Furthermore, Huang et al. (2011) successfully isolated 139 2-haloacid degrading bacteria from the sponge *Hymeniacidon perlevis* using liquid enrichment cultures.

Other liquid-based culture methods (i.e., liquid-solid media cultivation and liquid Winogradsky columns) have been applied by Gutleben et al. (2020) in order to isolate sponge-associated bacteria. For liquid-solid media cultivation, bacteria were incubated in a liquid medium and subsequently transferred to petri dishes containing the same medium solidified with gelrite and covered with the same liquid growth medium. In the case of “liquid” Winogradsky columns, bacteria were incubated in the liquid phase in 25 mL glass culture tubes that were present above artificial sediment composed of silica sand and crystalline cellulose as a carbon source. The columns were closed with metal caps and aluminum foil to allow oxygen diffusion and then incubated at room temperature under natural light conditions for 130 days. By applying these methods, Gutleben et al. (2020) recovered many bacteria that were not obtained from agar plates, including novel *Planctomycetes*, and many bacteria were cultivated solely by one of the cultivation approaches.

### Co-culture, Community Culture, Culture *in situ*, Culture in Simulated Natural Conditions

In natural environments, microorganisms often occur in complex networks with other organisms. For example, many obligate symbionts are extremely difficult to culture and isolate under laboratory conditions because they have co-evolved with their host, which leads to complex nutritional requirements or other growth conditions that are still unknown or hard to mimic *in vitro* (Taylor et al., 2007). It has been shown that co-culture can be a fruitful strategy for cultivation of recalcitrant microorganisms, and co-culture has yielded a number of previously uncultured bacteria, for example, microorganisms from marine sediments (Doesburg et al., 2006; D’Onofrio et al., 2010). A few recent studies have used co-cultivation to isolate bacteria associated with sponges and to stimulate the production of bioactive compounds that bacteria often fail to produce in pure cultures (Dashti et al., 2014; Adnani et al., 2015, 2017; Knobloch et al., 2019; S Hifnawy et al., 2020).

Bollmann et al. (2007) developed a diffusion-chamber-based cultivation approach, which allows microorganisms to grow in their natural environment by *in situ* cultivation. Microorganisms are incubated in a diffusion chamber, which is sealed off from the environment by membranes. The membranes are impermeable for microorganisms, but allow diffusion of molecules from the environment (e.g., nutrients, the “natural” sponge metabolome, and other signaling molecules) to the diffusion chamber, thereby giving the microorganisms access to metabolites and other molecules from their natural environment (Bollmann et al., 2007). Application of this method to cultivate sponge-associated bacteria was first reported by Steinert et al. (2014). A cell suspension of homogenized sponge tissue was mixed with growth media containing low, medium, or high nutrient concentrations and then poured into the diffusion chambers. Fully assembled diffusion chambers were inserted into the tissue of the sponge *Rhabdastrella globostellata* and incubated *in situ* for 4 weeks. After this incubation, the enrichment cultures from the diffusion chamber were further isolated on agar plates. By applying this method, the authors isolated 15 bacterial species that were not previously cultured. Thus, the study showed that the diffusion chamber method is one of the potential methods to enrich and subsequently isolate novel sponge-associated bacteria. However, it should be noted that not all sponge species are suitable for applying this method, as *Stylissa massa* rejected the growth chamber by retracting its tissue where the chamber had been inserted. In addition, encrusting sponges may be too small to fit the diffusion chamber. Therefore, modified diffusion growth chambers (e.g., smaller versions) are desirable.

Similarly, the I-tip developed by Jung et al. (2014) uses a yellow pipette tip as the basic element for *in situ* cultivation. It allows bacteria to enter and natural chemical compounds to diffuse into the tip, thereby allowing the bacteria to grow on a solid support in their natural environment. This device was applied for cultivation of bacteria and fungi from sponges from Lake Baikal and resulted in the recovery of a substantial fraction of the microbial diversity compared to standard agar plate cultivation at both genus and phylum levels.

Knobloch et al. (2019) used a co-cultivation approach to enhance the cultivability of sponge-associated bacteria in a multi-chamber device where *Halichondria panicea* explants and microbes were separated by a membrane. In brief, the bottom chamber of a microfiltration apparatus was filled with marine agar and the chambers were inoculated with sponge-derived bacterial suspension. These chambers were separated from the top part of the apparatus holding the sponge explants through a membrane. The devices were clamped together and placed in a seawater aquarium for 10 weeks. By applying this co-cultivation technique, Knobloch et al. (2019) showed that bacterial classes, such as *Spirochaetia*, *Fusobacteriia*, *Delta*- and *Epsilonproteobacteria*, and *Clostridia* were enriched compared to standard agar plate cultivation.

In summary, the above-mentioned cultivation techniques often result in substantially improving the cultivability of sponge-associated bacteria or at least in capturing different bacteria than with more traditional methods. However, these non-standard cultivation approaches have been only scarcely applied for the cultivation of bacteria from sponges and may be important to not retrieve the same opportunistic bacteria over and over again from sponges.

## Cultivable Bacterial Diversity From Sponges and Impact of Culture Conditions

### Composition of Cultivable Bacteria Associated With Sponges

**FIGURE 1:**
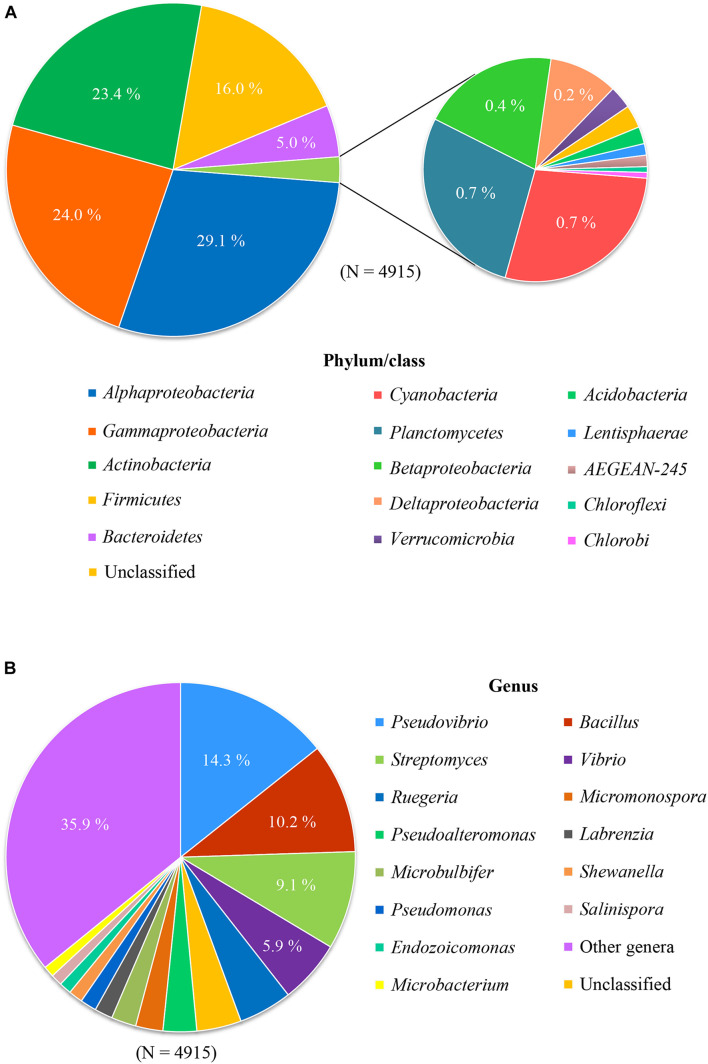
Composition of cultured bacteria associated with sponge at phylum/class level **(A)** and genus level **(B)**.

In order to investigate the diversity of the cultivable bacteria associated with sponges, a total of 4,915 16S rRNA gene sequences of cultured bacteria from sponges were selected based on published papers updated till 2017, retrieved from NCBI Genbank (see Supplementary File S1), and then re-classified based on the Silva database (v128) using the mothur pipeline (Schloss et al., 2009). Based on these collected 16S rRNA gene sequences of bacteria cultured from sponges, representatives of 11 bacterial phyla were retrieved, including *Proteobacteria*, *Actinobacteria, Firmicutes*, *Bacteroidetes*, *Cyanobacteria*, *Planctomycetes*, *Verrucomicrobia*, *Acidobacteria*, *Lentisphaerae*, *Chloroflexi*, and *Chlorobi* (Figure 1A). However, cultivable bacteria from sponges were dominated by four phyla: *Proteobacteria* (53.8%), *Actinobacteria* (23.4%), *Firmicutes* (16.0%), and *Bacteroidetes* (5.0%). Each of the remaining phyla accounted for <1% of the sequences. Almost all cultivable *Proteobacteria* belonged to two classes: *Alphaproteobacteria* (29.1%) and *Gammaproteobacteria* (24.0%).

These results show that extensive cultivation efforts have resulted in the isolation of a large phylogenetic diversity of sponge-associated bacteria. However, it is still far away from capturing the diversity of bacteria in sponges as described by culture-independent approaches. More than 60 bacterial phyla and candidate phyla have been detected from sponges by culture-independent methods, and predominant phyla include *Chloroflexi, Acidobacteria*, and *Cyanobacteria* (Thomas et al., 2016; Moitinho-Silva et al., 2017) that are rarely obtained in culture. At the genus level, the most cultured genera include *Pseudovibrio* (14.3%), *Bacillus* (10.2%), *Streptomyces* (9.1%), followed by *Vibrio* (5.9%) and *Ruegeria* (4.9%) (Figure 1B). However, use of cultivation-independent approaches has indicated that these genera were not prevalent in the original sponge samples, leading to the assumption that cultivation selects for opportunistic “weedy” bacteria that grow quickly in nutrient-rich media and outcompete slow-growing species that are more abundant in the original sample (Garland et al., 2001). Indeed, previous investigations where the bacterial species from the same sponge sample were studied by cultivation-dependent and cultivation-independent methods have shown little overlap (Li et al., 2011; Sipkema et al., 2011; Hardoim and Costa, 2014; Montalvo et al., 2014; Versluis et al., 2017).

### Impact of Isolation Methods and Culture Conditions on the Cultivable Bacteria Retrieved

For this review, information about culture conditions (e.g., isolation method, culture media, temperature) of strains was also retrieved from corresponding published papers for investigating the impact of culture conditions on the composition of the cultivable bacteria from those sponges. It is important to note that the dataset is not balanced and that there are large differences in the frequency at which certain methods have been used. This means that, in most cases, we have restricted ourselves to observational reporting of the data and not used statistics as the data distribution did not allow meaningful statistics.

**FIGURE 2:**
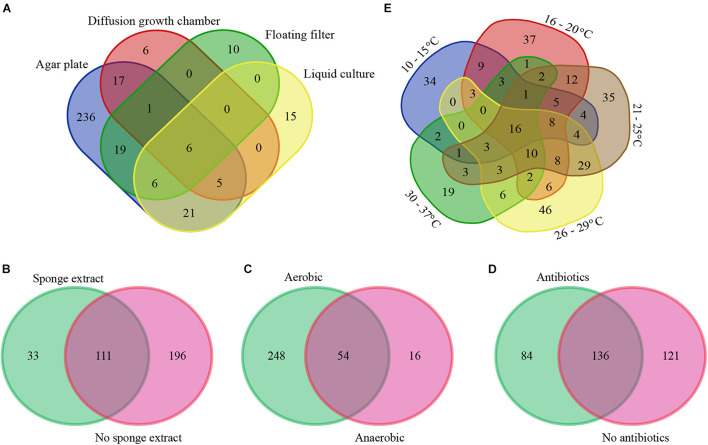
Venn diagram of sponge-associated bacteria cultured under different conditions: isolation methods **(A)**, with/without of sponge extracts in culture media **(B)**, with/without oxygen **(C)**, with/without antibiotics in culture media **(D)**, and temperatures **(E)** at genus level. The numbers in the figure indicate the number of genera.

**FIGURE 3:**
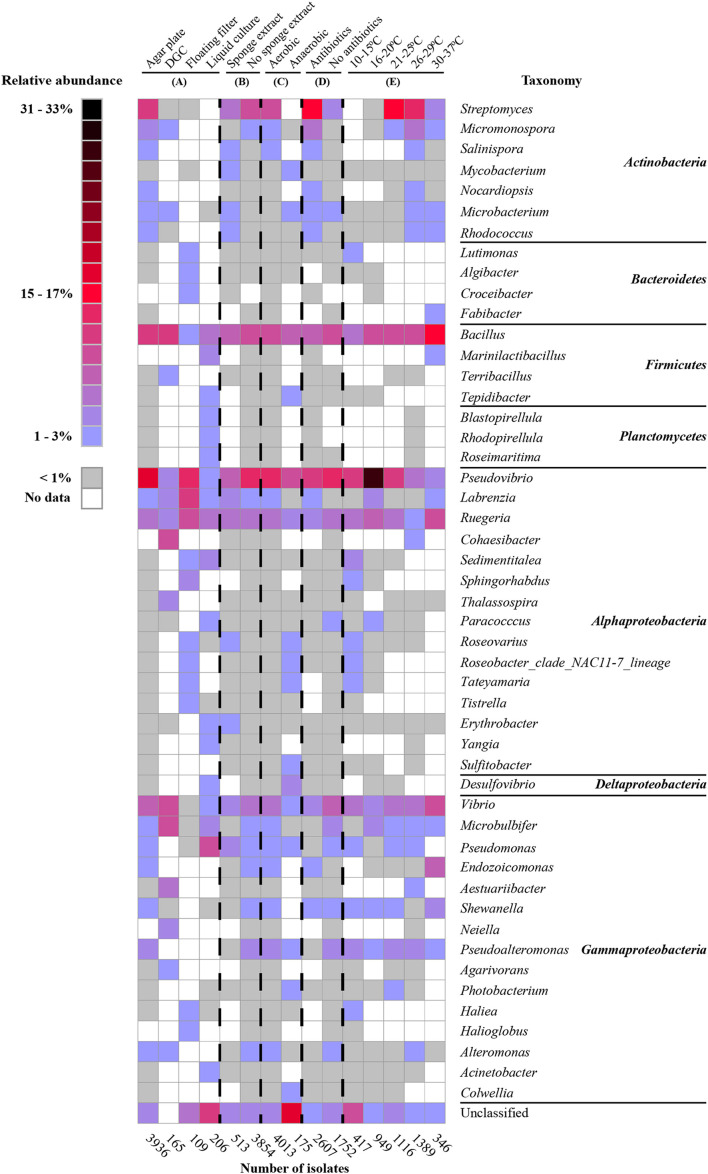
Heat map of the most abundant genera (50 genera) under different cultivation conditions: isolation methods **(A)**, with/without sponge extracts in culture media **(B)**, with/without oxygen **(C)**, with/without antibiotics in culture media **(D)**, and temperatures **(E)**. The percentages in the heatmap were calculated for each of the categories (sum for each column is 100%).

With respect to isolation methods, we only considered the following main methods: agar plates, diffusion growth chambers, floating filters, and liquid cultures (representing 4,398 out of the total of 4,915 isolates). Based on the collected data, agar plate-based cultivation has been the most popular method for the isolation of bacteria from sponges (used to isolate 89.1% of the cultivable bacteria), whereas bacteria that were isolated using the remaining methods, i.e., diffusion-growth chamber, liquid culture, and floating filter only accounted for 4.7, 3.7, and 2.5%, respectively. The use of different isolation methods has resulted in the recovery of different cultivable bacterial communities (Figures 2A, 3A). A total of 265 genera was only isolated by one method: 236 genera only on agar plates, 6 only in diffusion growth chambers, 10 only on floating filters, and 15 only in liquid culture (Figure 2A). Furthermore, several of the most abundant genera were isolated by only one method, such as *Salinispora, Nocardiopsis*, *Endozoicomonas*, *Pseudoalteromonas* (agar plate), *Cohaesibacter, Neiella* (diffusion-chamber), *Croceibacter, Halioglobus* (floating filter), and *Marinilactibacillus* (liquid cultures) (Figure 3A). Other genera were not only, but preferentially isolated through one of the methods. For example, cultivable bacteria on agar plates were dominated by *Streptomyces*, *Rhodococcus*, and *Shewanella*, whereas diffusion-chamber-based isolates were dominated by *Terribacillus, Thalassospira, Aestuariibacter*, and *Agarivorans*. Predominant floating filter-based isolates belonged to the genera *Lutimonas, Algibacter, Sphingorhabdus, Roseovarius*, *Roseobacter*, *Tateyamaria*, *Tistrella*, *Desulfovibrio*, and *Haliea*, and isolates from liquid cultures by *Tepidibacter, Blastopirellula*, *Rhodopirellula*, *Roseimaritima*, *Paracocccus*, *Erythrobacter*, *Yangia*, and *Acinetobacter* (Figure 3A). Other bacterial genera isolated from sponges were always obtained at high frequencies, no matter which cultivation method was used: *Bacillus*, *Pseudovibrio*, *Labrenzia*, *Ruegeria*, and *Vibrio*.

We also specifically considered studies that added sponge extract to culture media to provide the “natural nutrients” that sponge-associated bacteria may be exposed to Li and Liu (2006), Selvin et al. (2009), Sun et al. (2010), Sipkema et al. (2011), Steinert et al. (2014), Esteves et al. (2016). From the isolates obtained from media with (11.8%) and without (88.3%) sponge extract, 33 bacterial genera were only isolated from media with sponge extract, 196 bacterial genera only from media without sponge extract, and 111 genera from media both with and without sponge extract (Figure 2B). Generally, the relative abundance of the most frequently isolated genera was quite similar with and without sponge extract (Figure 3B). However, some genera were predominantly isolated from media either with or without sponge extracts, such as *Salinispora*, *Mycobacterium*, *Microbacterium*, *Rhodococcus* and *Erythrobacter* (with sponge extract), and *Micromonospora*, *Microbulbifer*, *Endozoicomonas*, *Pseudoalteromonas*, *Shewanella*, and *Alteromonas* (without sponge extract) (Figure 3B).

Similarly, cultivation with (95.7% of the isolates) or without oxygen (4.3%) led (as could be expected) to different bacterial recovery rates. Fifty-four genera were isolated both using oxic and anoxic conditions, whereas 248 genera were only isolated under oxic conditions and 16 genera only under anoxic conditions (Figure 2C). The most abundant genera isolated under oxic conditions belonged to the phylum *Actinobacteria* and the class *Gammaproteobacteria*. On the other hand, several genera such as *Mycobacterium*, *Microbacterium* (*Actinobacteria*), *Tepidibacter* (*Firmicutes*), *Roseobacter, Roseovarius, Tateyamaria* (*Alphaproteobacteria*), *Desulfovibrio* (*Deltaproteobacteria*), and *Photobacterium* and *Colwellia* (*Gammaproteobacteria*) were relatively more often obtained using anoxic conditions (Figure 3C).

Furthermore, the addition of antibiotics to culture media affected the composition of the isolated bacteria. In the presence of antibiotics, 84 bacterial genera were isolated that were not obtained from media without antibiotics. Similarly, cultivation without antibiotics resulted in the isolation of 121 bacterial genera that were not isolated from media with antibiotics (Figure 2D). Genera from the phylum *Actinobacteria* were isolated at higher frequencies from media supplemented with antibiotics, whereas media without antibiotics generally enhanced the recovery of genera from the class *Gammaproteobacteria* (Figure 3D).

The use of different incubation temperatures led to the isolation of different bacterial genera: 34 genera were only isolated at a temperature ranging from 10 to 15°C, 37 genera only at 16 to 20°C, 35 genera only at 21 to 25°C, 46 genera only at 26 to 29°C, and 19 genera only at 30 to 37°C, whereas relatively lower numbers of genera were isolated at multiple temperature ranges (Figure 2E). Except for a limited number of genera, such as *Bacillus*, *Pseudovibrio*, *Ruegeria*, and *Vibrio* that were isolated at a wide range of temperatures, other bacterial genera were primarily recovered at a more restricted temperature range. For example, genera belonging to the phylum *Actinobacteria* tended to grow best at a range of 21–37°C, whereas many genera of *Alphaproteobacteria* grew better at lower temperatures (Figure 3E).

**FIGURE 4:**
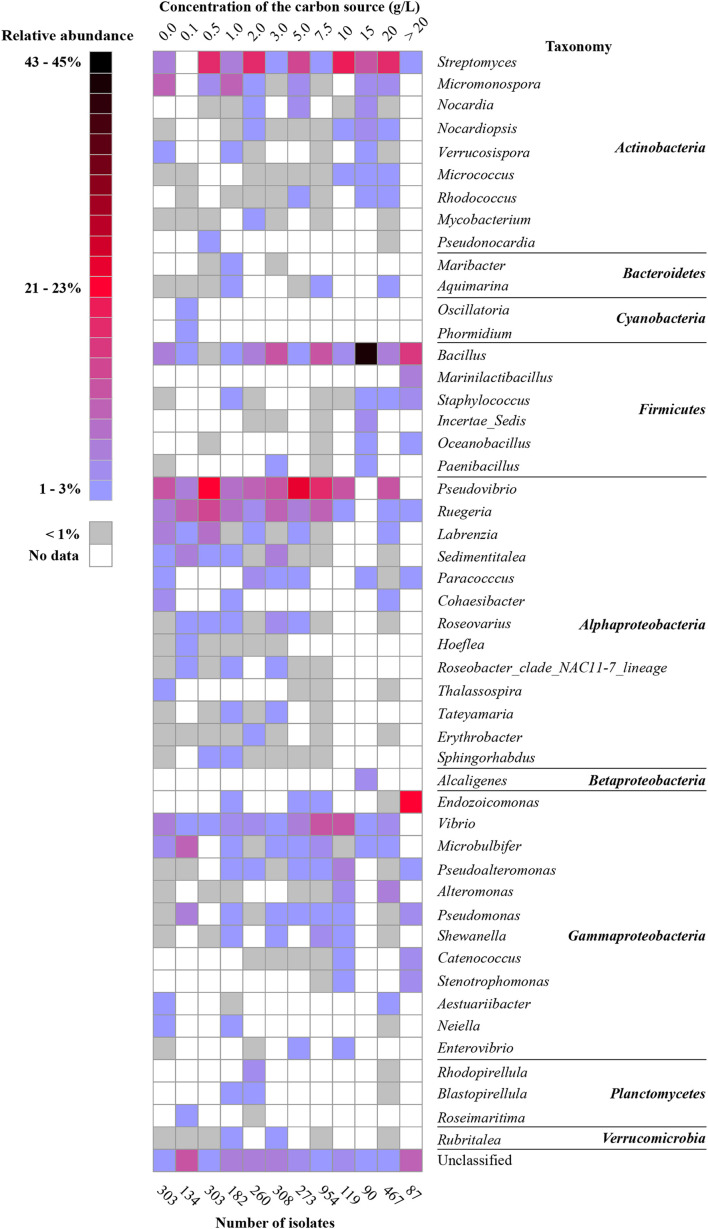
Heat map of the most abundant genera (50 genera) based on carbon amount in culture media. The percentage was calculated for each of the categories (sum for each column is 100%).

The impact of medium composition can be assessed for many different medium components. In this review, we chose to investigate the impact of the amount of organic carbon in culture media on the isolated bacterial diversity from sponges. Several genera were isolated for the entire range of carbon concentrations from culture media containing low organic carbon concentrations to media with high organic carbon concentrations. These genera include *Bacillus, Pseudovibrio, Ruegeria, Vibrio*, *Pseudomonas*, and *Streptomyces* (Figure 4). In contrast, other genera were particularly recovered from seawater without additional carbon sources, such as *Micromonospora, Labrenzia, Cohaesibacter*, and *Microbulbifer*. Yet other genera, mainly belonging to phyla/classes *Cyanobacteria*, *Alphaproteobacteria*, *Planctomycetes*, *Bacteroidetes*, and *Verrucomicrobia* were relatively more often retrieved from media with lower organic carbon concentrations (≤5.0 g/L), while many genera belonging to phyla/classes *Actinobacteria*, *Firmicutes*, and *Gammaproteobacteria* grew better in media containing higher organic carbon concentrations (>5 g/L) (Figure 4).

Overall, our meta-analysis showed that isolation methods, culture conditions, and growth medium composition substantially influence the composition of the cultivable bacterial community from sponges. Although this result of differentially recovered bacteria may in part be the result of the severe imbalance in the data in that some methods and media are overrepresented, and that isolation from a given sample was only done by a subset of methods and media, it may provide leads for future experiments targeting particular members of the sponge-associated microbiota. Although agar plate-based experiments may be the most straightforward method to obtain pure cultures, the growth of single strains poorly reflects the situation in the mesohyl, especially for high-microbial-abundance sponges where a large number of different bacterial species is densely packed together. Thus, unless the nature and relevance of interactions between sponge cells and bacteria (or between different bacteria) are known and deducted how these interactions can be compensated for by supplying specific media components, agar plate-based assays are unlikely to result in the recovery of the uncultivated majority of sponge symbionts. Supplying a sponge extract may alleviate some of these issues (e.g., Li and Liu, 2006; Selvin et al., 2009; Sun et al., 2010; Sipkema et al., 2011; Steinert et al., 2014; Esteves et al., 2016); however, the often used sponge extracts (e.g., organic sponge extract, aqueous sponge extract) may not be the best choice to capture primary metabolites that may be exchanged between the sponge host and bacteria. In addition, the concentrations used may be too low to sustain sufficient bacterial cell divisions to obtain visible colonies. On the other hand, in many attempts to isolate bacteria from sponges, carbon (and other nutrients) concentrations are much higher than those expected to prevail inside the sponge tissue and are likely to select for bacteria such as *Pseudovibrio*, *Bacillus*, *Ruegeria, Vibrio*, *Pseudomonas*, and *Streptomyces* spp. One aspect that we did not consider for this review is the duration of cultivation experiments. We know little about generation times of bacteria inside the sponge tissue, but dividing bacteria are not commonly seen on electron microscopic images (Gloeckner et al., 2014), which suggests that division rates in the natural environment are relatively low. As such, long cultivation experiments spanning several weeks or months may be required to successfully isolate sponge-associated bacteria. Having said that, the duration of cultivation experiments was often not accurately reported for individual isolates, and therefore the impact of cultivation duration could not be properly assessed.

### Sponge-Specific and Sponge-Coral-Specific Cultivable Bacteria

Cultivation-independent sponge-associated bacterial profiling studies have revealed that sponges host bacterial communities distinct from communities in the surrounding seawater (Naim et al., 2014; Alex and Antunes, 2015; Rodríguez-Marconi et al., 2015; Weigel and Erwin, 2015; Steinert et al., 2016). Initial studies based on electron microscopy have shown the existence of three groups of sponge-associated microbes: (i) abundant microbes in the sponge mesohyl, (ii) a small number of intracellular microbes, and (iii) transient microbes from the surrounding seawater (Vacelet, 1975; Wilkinson, 1978). In addition, microscopic studies of sponge larvae and embryos, and subsequent molecular studies have shown that many sponge-associated bacteria can be acquired vertically and/or horizontally (Wilkinson et al., 1981; Ereskovsky et al., 2005; Enticknap et al., 2006; De Caralt et al., 2007; Schmitt et al., 2007; Sharp et al., 2007; Lee et al., 2009; Sipkema et al., 2015). Recent sponge microbiology studies based on phylogenetic analyses of the bacterial 16S rRNA genes retrieved from sponges revealed the existence of sponge-specific (SC) bacteria, i.e., bacteria that are highly enriched in sponges compared to other environments where they are rarely present (Hentschel et al., 2002; Taylor et al., 2007; Simister R.L. et al., 2012). Simister R. et al. (2012) published a comprehensive phylogenetic study based on near-complete 16S rRNA gene sequences of sponge-associated microorganisms and identified that 27% of 7,546 sponge-derived 16S rRNA gene sequences belonged to sponge-specific clusters (SCs) or sponge-coral-specific clusters (SCCs), the latter comprising sequences highly enriched in both sponges and corals. This comprehensive phylogeny included 205 bacterial SC(C)s. In order to determine which SC(C)s have cultured representatives, we integrated the 16S rRNA gene sequences of bacteria cultured from sponges from published papers (until 2017) into the phylogenetic tree constructed by Simister R. et al. (2012). Briefly, the 4,915 sequences collected for this review as well as the sequences from the phylogenetic tree from Simister R. et al. (2012) were aligned and then the new sequences collected for this review were added to the previous phylogeny using the RAxML algorithm (see Supplementary File S1). Based on our phylogeny, 201 sequences from cultured sponge-derived bacteria were clustered with 37 of the SC(C)s proposed by Simister R. et al. (2012) (Supplementary Table S1 and Supplementary Figures S1–S11). These cultured isolates within SC(C)s belong to the phyla/classes *Alphaproteobacteria, Betaproteobacteria, Gammaproteobacteria*, *Actinobacteria, Bacteroidetes, Firmicutes*, and *Cyanobacteria*. The majority of cultivable bacteria that belong to SC(C) represent frequently cultured genera, such as *Pseudovibrio*, *Shewanella*, *Vibrio*, and *Bacillus* (Supplementary Table S1). Although the isolation of most bacteria belonging to SC(C) was achieved using agar plates, liquid culture and floating filter cultivation also contributed to the isolation of seven and two bacteria belonging to SC(C)s, respectively. The use of antibiotics as well as sponge extracts in culture media in some cases also allowed to isolate additional bacteria from SC(C)s. Forty-one bacterial strains belonging to SC(C)s were retrieved from culture media supplemented with antibiotics, whereas 19 bacterial strains belonging to SC(C)s were retrieved from culture media supplemented with sponge extract.

**FIGURE 5:**
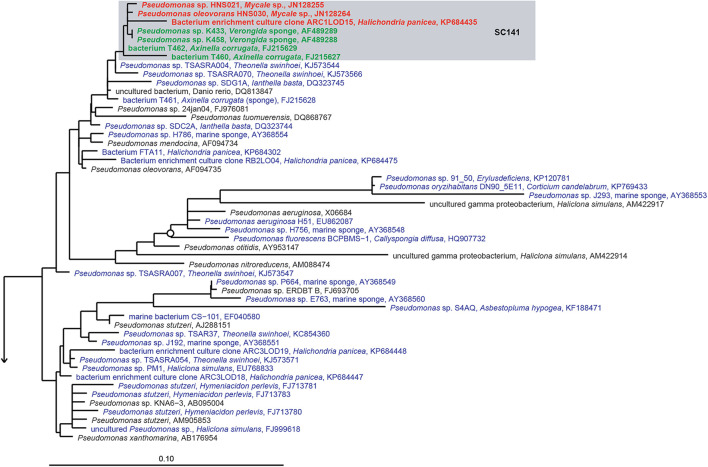
16S rRNA gene-based phylogeny of sponge-specific cluster SC141 within the *Gammaproteobacteria*. Filled circles indicate bootstrap support of ≥90%, and open circles represent bootstrap support of ≥75%. SC indicates sponge-specific cluster, and SCC indicates sponge- and coral-specific cluster. Blue letters indicate cultured bacteria from sponges, bold green letters indicate cultured bacteria belonging to known SC(S)s by Simister R.L. et al. (2012), and bold red letters indicate newly cultured bacteria added into known SC(C)s.

**FIGURE 6:**
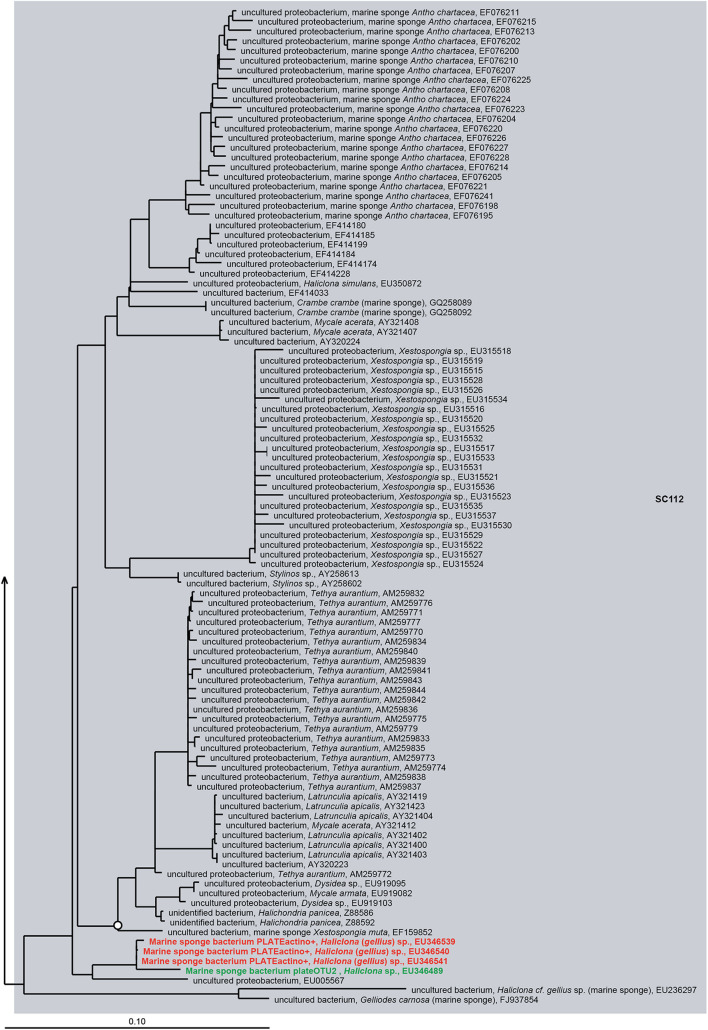
16S rRNA gene-based phylogeny of sponge-specific cluster SC112 within the *Betaproteobacteria*. Details are as provided for Figure 5.

Notably, some SC(C)s contain only sequences obtained from bacterial isolates and were not found by cultivation-independent means (SC11, SC13, SC14, SC55, SC58, SC59, SC60, SC61, SC78, SC84, SC85, SC93, SC108, SC126, SC127, SC128, SC133, SC138, SC139, SC141) (Figure 5 and Supplementary Table S1). These clusters mainly represent isolates from the genera *Bacillus, Pseudovibrio, Ruegeria, Nocardiopsis, Brevibacterium, Pseudomonas*, *Rheinheimera*, *Vibrio*, *Pseudoalteromonas, Aquimarina*, and *Erythrobacter*, which are usually absent or found at very low abundances by cultivation-independent methods (Li et al., 2011; Sipkema et al., 2011; Hardoim and Costa, 2014; Montalvo et al., 2014; Versluis et al., 2017). This implies that, although representatives from these clusters have been isolated from multiple sponge species, they are most likely present as part of the rare biosphere or as spores (for some *Firmicutes* and *Actinobacteria*) in sponges. However, the fact they are part of the rare biosphere does not exclude that these bacteria may have specific relationships with sponges, as they have been selectively isolated from sponges and not (yet) from other environments. For example, for sponge-associated *Pseudovibrio* spp. making part of SC84 and SC85, it has been found that their genomes are enriched in SEL1 and tetratricopeptide repeats type III, IV, and VI secretion systems, which have been implicated in host colonization (Versluis et al., 2018). On the other hand, it has become obvious that bacteria from these genera are not abundant in sponges and they are unlikely to have a substantial quantitative role in sponge holobiont metabolism. The isolates that make part of other SC(C)s including sequences from both uncultivated and cultivated bacteria (SC26, SC27, SC28, SC56, SC62, SC86, SC94, SC112, SC130, SC132, SC137, SC149, SCC6, SCC7, SCC18, SCC28, SCC31) (Figure 6 and Supplementary Figures S1–S11) are more likely to represent true sponge symbionts. These clusters belong to a range of different taxa, including TK85 (*Acidobacteria*), *Candidatus* Branchiomonas (*Betaproteobacteria*), the Pir4 lineage (*Planctomycetes*), *Flavobacteriaceae* and *Fabibacter* (*Bacteroidetes*), *Oscillatoria* (*Cyanobacteria*), *Clostridiaceae* (*Firmicutes*), *Rhodospirillales*, *Erythrobacter* and *Tistlia* (*Alphaproteobacteria*), *Alteromonadales*, BD1-7 clade, *Enterobacteriaceae*, *Endozoicomonas*, and *Stenotrophomonas* (*Gammaproteobacteria*). These taxa are often found at a high relative abundance in sponges by cultivation-independent methods, which implies that these bacteria may play important roles in the sponge holobiont, which makes them particularly interesting in the study of sponge-symbiont relationships. However, based on the current data of successful isolation of representatives of sponge-specific bacteria, it is not straightforward which conditions or media are recipes for success in isolating sponge-specific bacteria. Understanding the sponge-symbiont and symbiont-symbiont interactions and the molecules that mediate these interactions may be keys to develop more successful protocols for cultivation of sponge-specific bacteria.

### Sponge-Enriched Cultivable Bacteria

Recent deep sequencing studies revealed that some sequences that make part of SC(C)s are also found in seawater and sediment samples, albeit at very low relative abundances (Webster et al., 2010; Taylor et al., 2013; Thomas et al., 2016). Therefore, these clusters can no longer be strictly called “sponge-specific,” but are better described as “sponge-enriched” (Taylor et al., 2013; Thomas et al., 2016). In order to examine which cultivable bacteria can be classified as ‘‘sponge-enriched,’’ the collected 16S rRNA gene sequences of cultivable bacteria in this review were blasted against 64,424 ASVs that were extracted from the sponge Earth Microbiome Project (EMP) database.^1^ In total, 3636 out of 4,915 16S rRNA gene sequences of cultivable bacteria from sponges were 100% similar to 577 ASVs from the sponge Earth Microbiome Project (EMP) database. However, the majority of the cultivable bacteria from sponges were found to be significantly enriched in seawater and sediment samples, and only 180 cultivable bacteria (21 ASVs) were significantly enriched in sponges (Supplementary Table S2). As such, comparing the 16S rRNA gene sequences from sponge isolates with the larger sponge EMP project dataset of shorter sequences confirms that a substantial number of cultivable bacteria from sponges are probably not true sponge symbionts (e.g., bacteria from the outer surface of sponges, transient bacteria from the surrounding seawater) because they are enriched in the environment rather than in sponges.

## Promising Strategies and New Methods for Cultivation of Yet Uncultured Sponge-Associated Bacteria in the Future

As mentioned above, extensive cultivation efforts have led to a number of successes in the cultivation of sponge-associated bacteria. However, the large majority of sponge-associated bacteria has remained uncultivable up to now. Therefore, innovative strategies are needed.

### Modification of the Preparation of Culture Media

One of the major reasons for the poor cultivability of sponge-associated bacteria in the laboratory is that we might not know the required nutrients to support the growth of sponge-associated bacteria. Some low hanging fruits may be picked by small modifications in the preparation of cultivation media. Chemical reactions between medium components during autoclaving, for instance, between phosphate and sugars, proteins, or agar have been found to produce compounds that inhibit the growth of bacteria (e.g., hydrogen peroxide) (Finkelstein and Lankford, 1957; Nakashima et al., 2010; Tanaka et al., 2014; Kawasaki and Kamagata, 2017). The addition of hydrogen peroxide scavengers such as catalase and pyruvate to culture media were shown to remove hydrogen peroxide and yield higher colony counts (Kawasaki and Kamagata, 2017). Furthermore, replacing agar with alternative gelling agents (e.g., gellan gum) in solid media is expected to improve the cultivability of some sponge-associated bacteria. Several studies on soil, freshwater sediment, and seawater have shown that numbers of viable counts and colonies increase on substrates solidified with gellan gum compared to media solidified with agar (Shungu et al., 1983; Lin and Casida, 1984; Harris, 1985; Rule and Alexander, 1986; Janssen et al., 2002; Tamaki et al., 2005, 2009; Rygaard et al., 2017), resulting in the isolation of previously uncultured bacteria.

### Development of Culture Media Based on “Omics”-Derived Information

Although some bacteria may be “rescued” by more fine-tuned media preparation, it remains mostly a black-box approach. The development of “omics” approaches can provide more functional information about sponge microbiota, e.g., presence or absence of metabolic pathways and gene expression levels, which may be used to design new omics-inspired culture media (Gutleben et al., 2018 and references therein). For instance, genome analyses revealed that *Poribacteria* has the genomic repertoire for autotrophic CO_2_-fixation through the Wood-Ljungdahl pathway (Kamke et al., 2014). The same was found for another well-known sponge symbiont, the sponge-specific archaeon *Cenarchaeum symbiosum*, for which it has been shown that it uses ammonia as an energy source and carbon dioxide as a carbon source (Hallam et al., 2006), a carbon source rarely used to isolate bacteria from sponges. The genome of a sponge-specific uncultured delta-proteobacterium was found to possess a glutathione porter that allows for growth on glutathione as the sole sulfur source, which can be exploited in cultivation experiments. In addition, its genome encodes a tetracycline resistance protein (Liu et al., 2011) and points toward the incorporation of tetracycline in the medium to limit the growth of non-target bacteria. Furthermore, genomes of *Aplysina aerophoba*-associated bacteria revealed the nutritional specialization of two symbiont groups. The first group, including members of SAR202, Sva0996, OM1, TK85, *Nitrospinae*, *Desulfurellaceae*, *Rhodospirillaceae*, and *Alphaproteobacteria*, has genomes that are enriched in genes related to carnitine metabolism, while genomes of the second group, including members of *Albidovulum*, *Poribacteria*, *Spirochaetaceae*, *Caldilineaceae*, and *Chloroflexi* are enriched in genes related to sulfated polysaccharide metabolism. Both are abundant molecules of the sponge extracellular matrix, and therefore it is hypothesized that the sponge symbionts feed on the sponge cells that are shed as part of the cell turnover, and on components of the sponge extracellular matrix (Slaby et al., 2017). Thus, a growing body of literature points toward testing autotrophic growth conditions and the application of a range of sponge host-derived molecules as carbon sources.

### Innovative Cultivation Methods

A number of innovations in bacterial cultivation have been developed, and although they have occasionally been applied for the isolation of bacteria from sponges, their potential is far from exhausted. A series of *in situ* cultivation methods, such as diffusion chamber cultivation (Steinert et al., 2014), I-tip (Jung et al., 2014), iCHIP (Nichols et al., 2010), hollow-fiber membrane chamber (HFMC) (Aoi et al., 2009), single-colony co-cultivation (Tanaka and Benno, 2014), substrate membrane system (Svenning et al., 2003), cultivation trap (Gavrish et al., 2008), and cultivation in agar spheres (Ben-Dov et al., 2009), have only scarcely been used for the isolation of sponge-associated bacteria, but may be used to overcome the limitations of pure cultures.

In addition, cultivable bacteria from sponges are usually species that are present at low relative abundances in the sponge mesohyl, whereas the most abundant bacteria detected in sponges by culture-independent methods still have not been cultured (Li et al., 2011; Sipkema et al., 2011; Hardoim and Costa, 2014; Montalvo et al., 2014; Versluis et al., 2017). It is likely that fast-growing microbes compete for resources with uncultured slow-growing species. As such, it may be important to separate single microbial cells from complex microbial communities, e.g., through dilution-to-extinction, microfluidics, flow cytometry, micromanipulation, and compartmentalization to grow targeted bacteria physically separated from each other (Ben-Dov et al., 2009; Ishii et al., 2010; Ma et al., 2014; Pivetal et al., 2014; Jiang et al., 2016; Zhou et al., 2018; Benítez et al., 2021), while perhaps retaining possibilities for interactions using the cultivation setups listed earlier in this paragraph. These methods have led to the isolation of previously uncultured bacterial species of clades SAR11 (Alphaproteobacteria), OM43 (Betaproteobacteria), SAR92 (Gammaproteobacteria), and OM60/OM241 (γ subclass) (Connon and Giovannoni, 2002; Simu and Hagström, 2004; Stingl et al., 2007; Song et al., 2009) that are numerically abundant in their environment.

The last point that deserves attention is the dereplication of bacterial isolates. Every cultivation study may result in the isolation of hundreds or even thousands of colonies/cultures. However, the majority of the strains retrieved have been isolated before or are replicate colonies of the same strain. Therefore, the use of dereplication technologies to rapidly screen for replicate isolates is important to save time and resources. Several dereplication tools have been used and shown their effectiveness in rapid differentiation and identification of bacteria in cultivation studies, such as matrix-assisted desorption/ionization-time-of-light mass spectrometry (MALDI-TOF MS) (Dieckmann et al., 2005, 2008; Lagier et al., 2012; Pfleiderer et al., 2013; Dubourg et al., 2014; Strejcek et al., 2018) and Fourier transform-infrared (FT-IR) spectroscopy (Kirschner et al., 2001; Oberreuter et al., 2002; Ngo-Thi et al., 2003; Naumann, 2006; Rebuffo et al., 2006; Beekes et al., 2007). The specificity of these techniques is rather high and may allow to differentiate bacteria down to the subspecies or strain level (Naumann, 2006; Beekes et al., 2007). As such, these tools may allow quick identification of “usual suspects” among the sponge-associated isolates, such as *Pseudovibrio*, *Bacillus*, and *Ruegeria* and direct efforts toward the yet uncultured bacteria.

## Conclusion

Although the majority of bacteria from sponges have remained resistant to cultivation in the laboratory, the accumulative cultivation efforts of many researchers have resulted in the cultivation of bacteria from 11 bacterial phyla. These isolates are dominated by the phyla *Proteobacteria*, *Actinobacteria*, *Firmicutes*, and *Bacteroidetes* and the genera *Pseudovibrio*, *Bacillus*, *Streptomyces*, *Vibrio*, and *Ruegeria*. Furthermore, among the isolates are representatives of 21 sponge-enriched bacteria as defined by the sponge EMP (Moitinho-Silva et al., 2017) and 37 SC(C)s as defined by Simister R.L. et al. (2012). However, this also implies that 168 SC(C)s have no representative isolate. Meta-analysis of culture conditions and growth media used suggests a substantial impact of these on the taxa isolated, most obvious for cultivation temperature and carbon concentration present in the media. In conclusion, the cultivation of especially the most abundant sponge symbionts is still far from realized because of our limited understanding of the complex sponge-symbiont interactions and translating them to pseudo natural growth conditions for these symbionts in the laboratory. A next level of cultivation strategies will be needed to recover those bacteria in isolation as well. This would also be a major step forward to the application of bioactive compounds found in sponges.

## Author Contributions

TD, GS, NC, DS, and HS designed the hypothesis. TD conducted the literature collection and literature research, and drafted the original manuscript. TD and GS analyzed the data. All authors critically contributed to manuscript revision and approved the submitted version.

## Conflict of Interest

The authors declare that the research was conducted in the absence of any commercial or financial relationships that could be construed as a potential conflict of interest.

## Publisher’s Note

All claims expressed in this article are solely those of the authors and do not necessarily represent those of their affiliated organizations, or those of the publisher, the editors and the reviewers. Any product that may be evaluated in this article, or claim that may be made by its manufacturer, is not guaranteed or endorsed by the publisher.

## Footnotes

1
github.com/amnona/SpongeEMP
