# Supplementary material for: Bacteria Cultivated From Sponges and Bacteria Not Yet Cultivated From Sponges—A Review
Source: Front Microbiol. 2021 Nov 10;12:737925. doi: 10.3389/fmicb.2021.737925 (PMC8634882; doi:10.3389/fmicb.2021.737925)
Supplement: Supplementary file 1 [file Image_1.pdf]

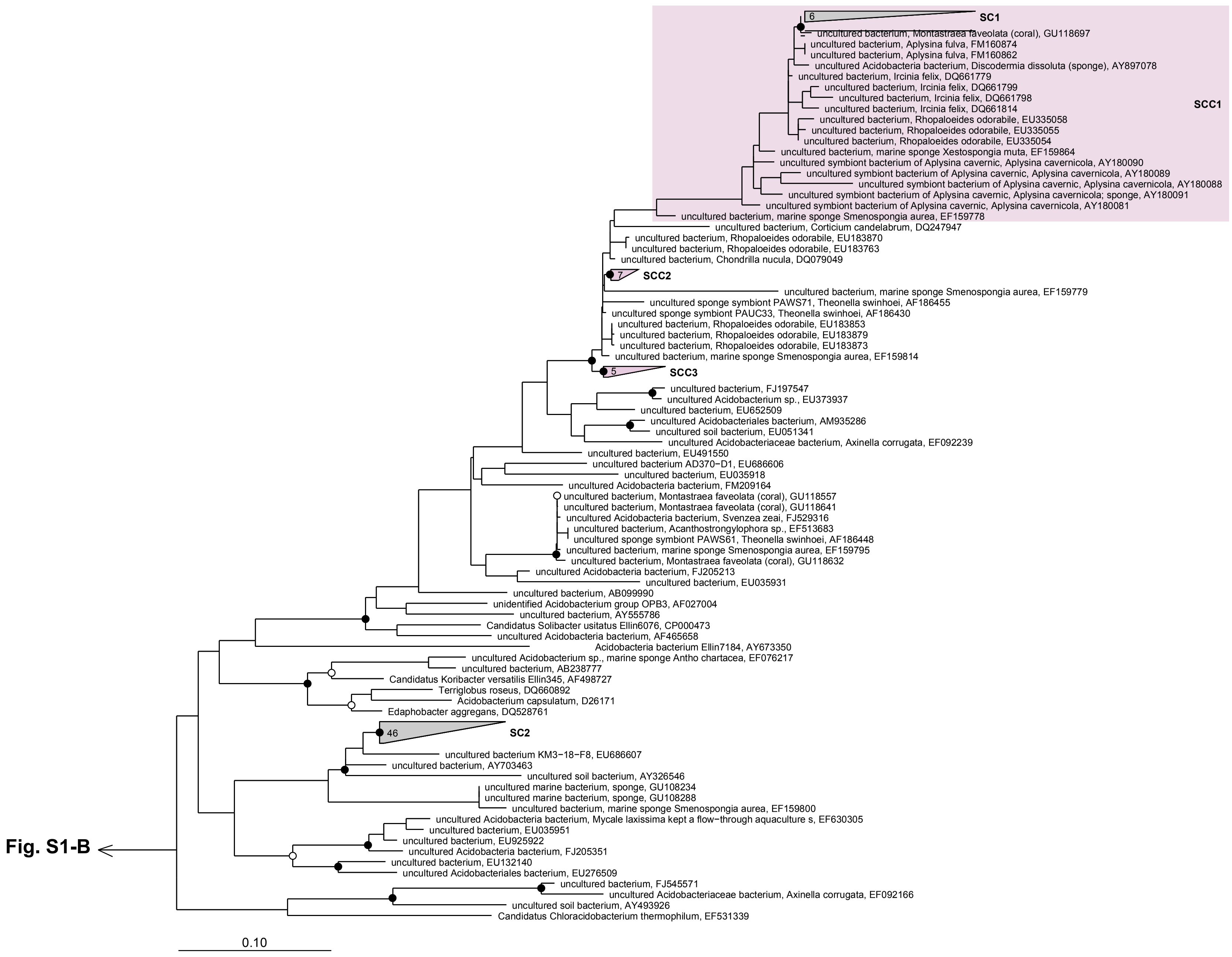

Figure S1-A. 16S rRNA gene-based phylogeny of sponge-associated Acidobacteria. Filled circles indicate bootstrap support of ≥ 90%, and open circles represent bootstrap support of ≥ 75%. SC indicates sponge-specific cluster, and SCC indicates sponge- and coral-specific cluster. Blue letters indicate cultured bacteria from sponges, bold green letters indicate cultured bacteria belonging to known SC(C)s by Simister et al. (2012), and bold red letters indicate newly cultured bacteria added into known SC(C)s.

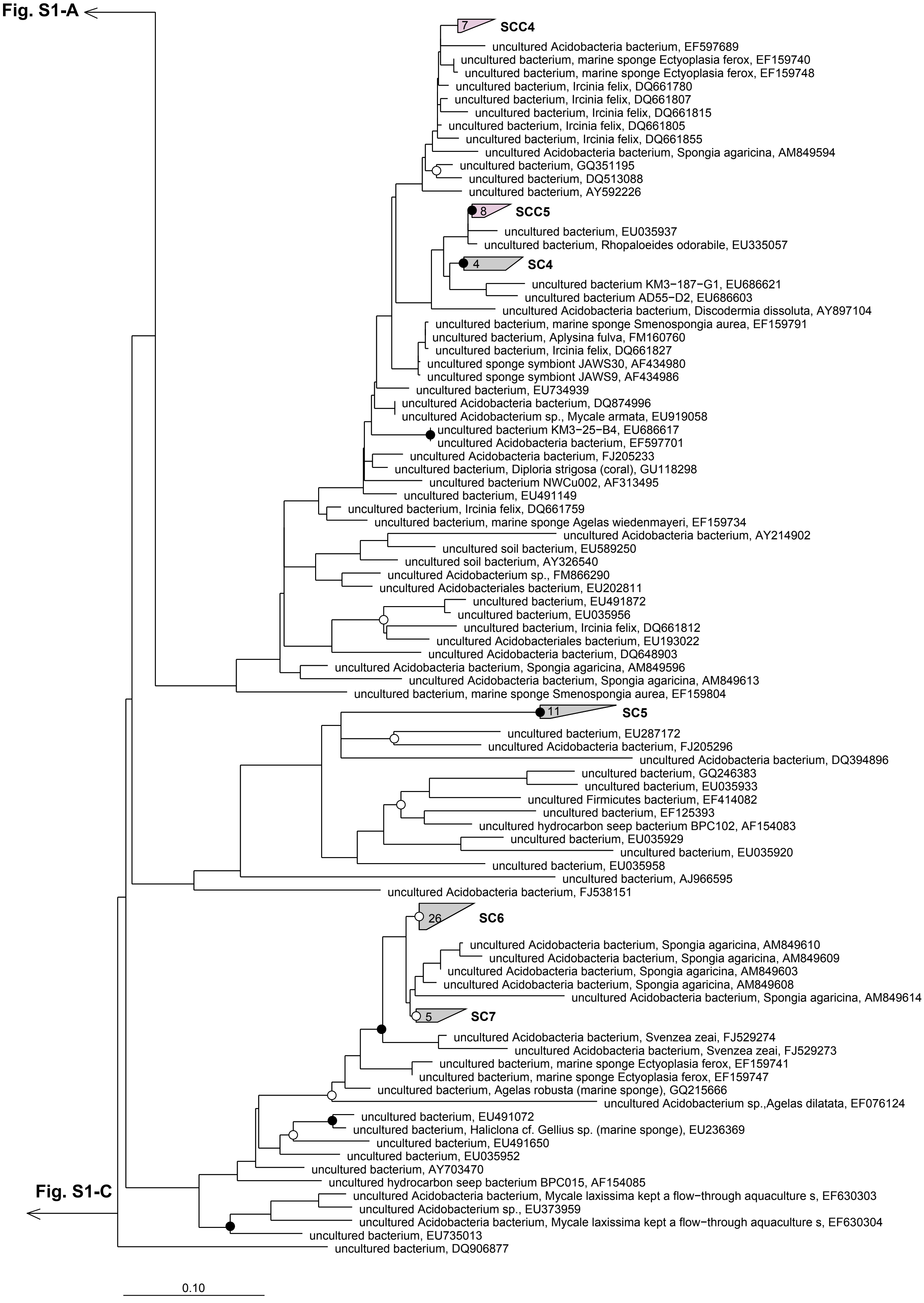

Figure S1-B. 16S rRNA gene-based phylogeny of sponge-associated Acidobacteria. Details are as provided for Figure S1-A

Fig. S1-B

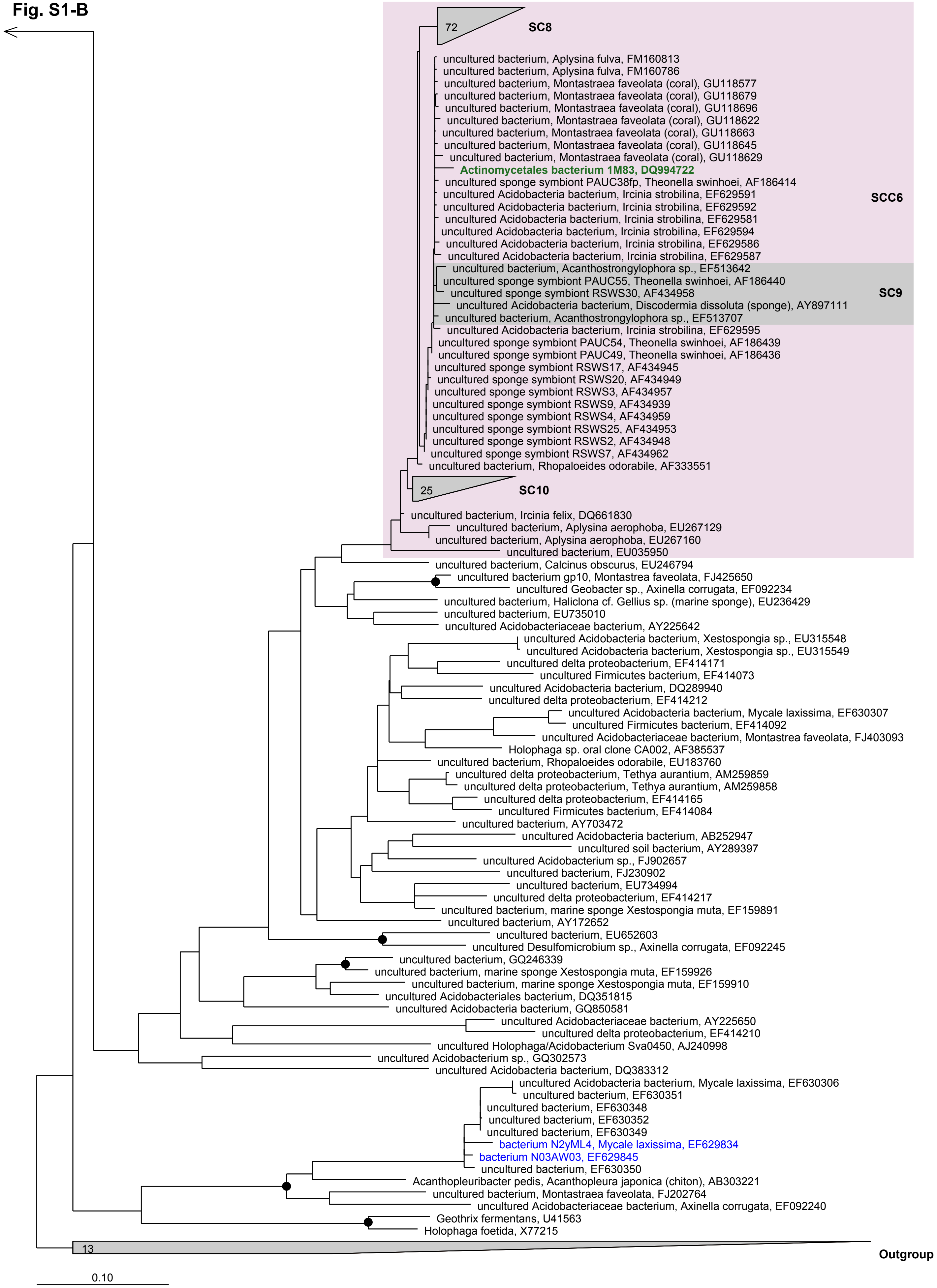

Figure S1-C. 16S rRNA gene-based phylogeny of sponge-associated Acidobacteria. Details are as provided for Figure S1-A
