# Supplementary material for: Bacteria Cultivated From Sponges and Bacteria Not Yet Cultivated From Sponges—A Review
Source: Front Microbiol. 2021 Nov 10;12:737925. doi: 10.3389/fmicb.2021.737925 (PMC8634882; doi:10.3389/fmicb.2021.737925)
Supplement: Supplementary file 2 [file Image_2.pdf]

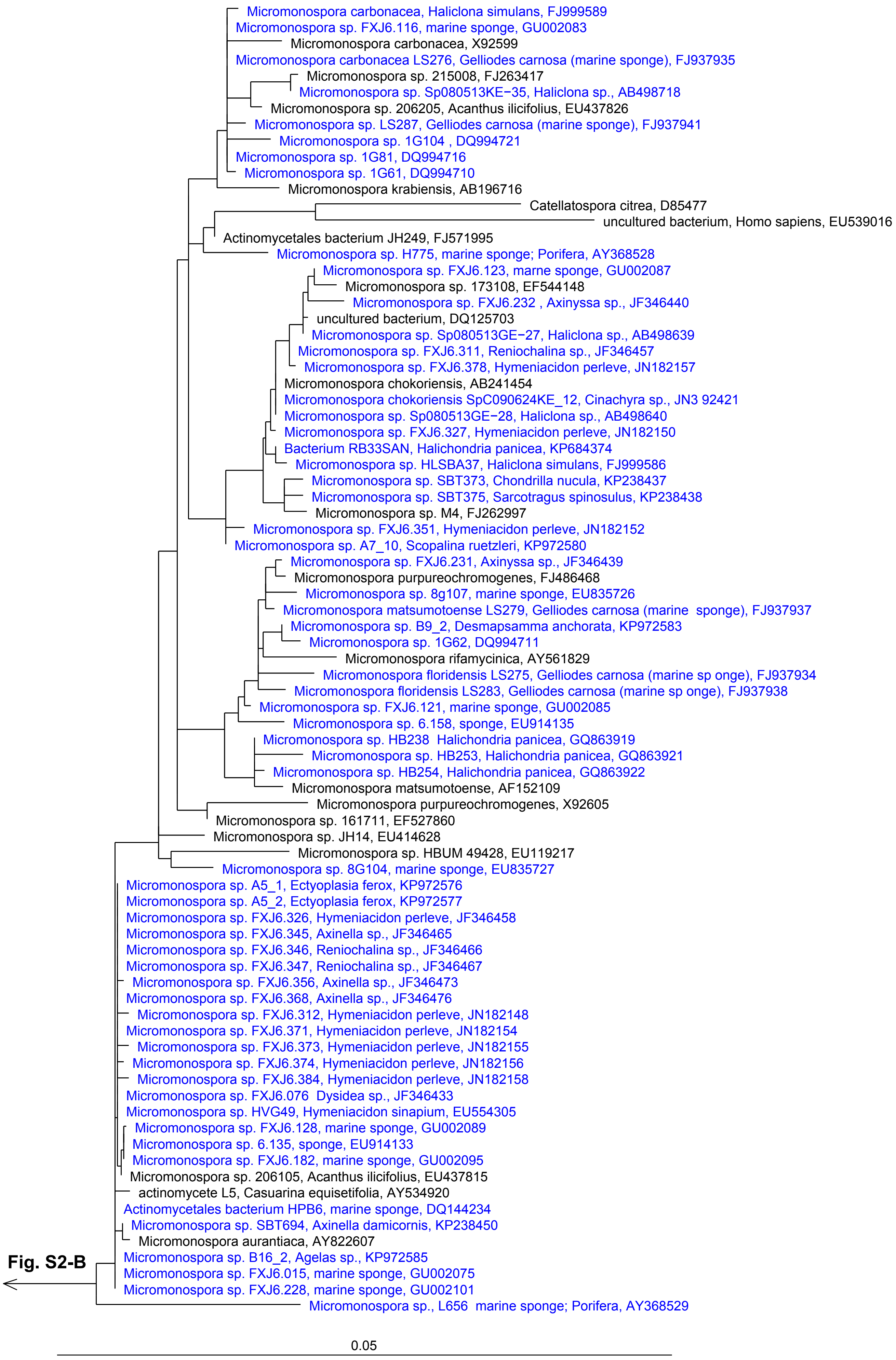

Figure S2-A. 16S rRNA gene-based phylogeny of sponge-associated Actinobacteria. Details are as provided for Figure S1

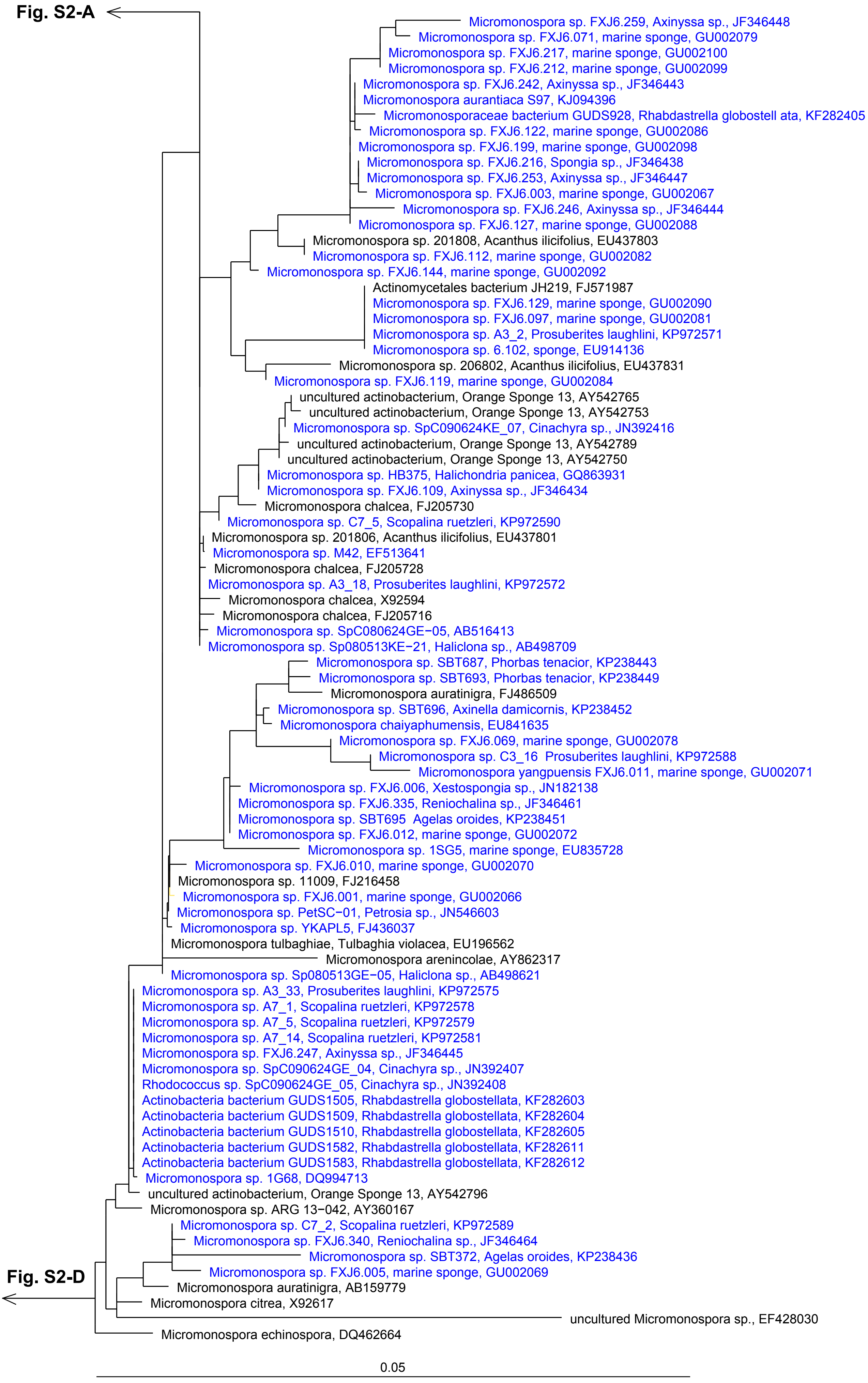

Figure S2-B. 16S rRNA gene-based phylogeny of sponge-associated Actinobacteria. Details are as provided for Figure S1

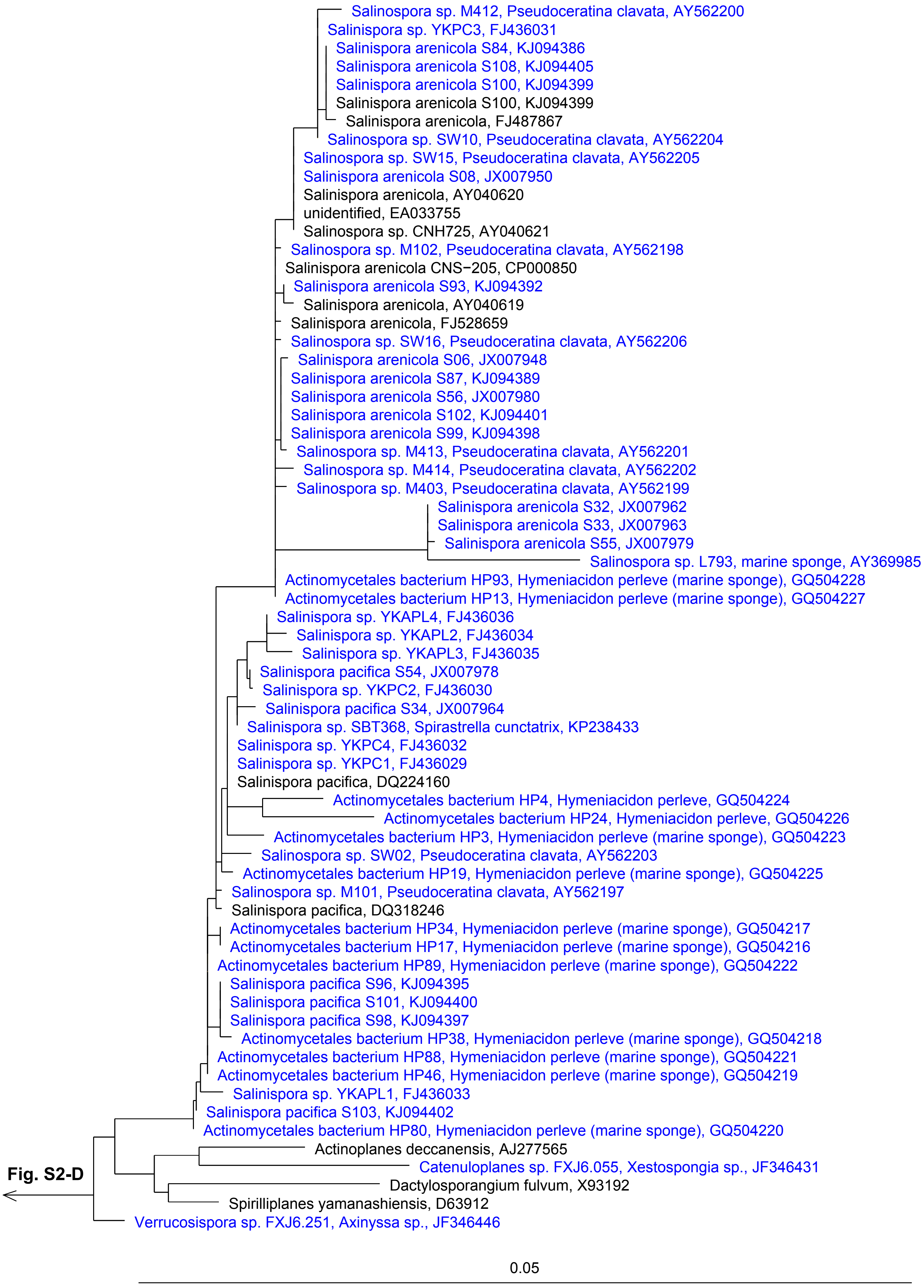

Figure S2-C. 16S rRNA gene-based phylogeny of sponge-associated Actinobacteria. Details are as provided for Figure S1

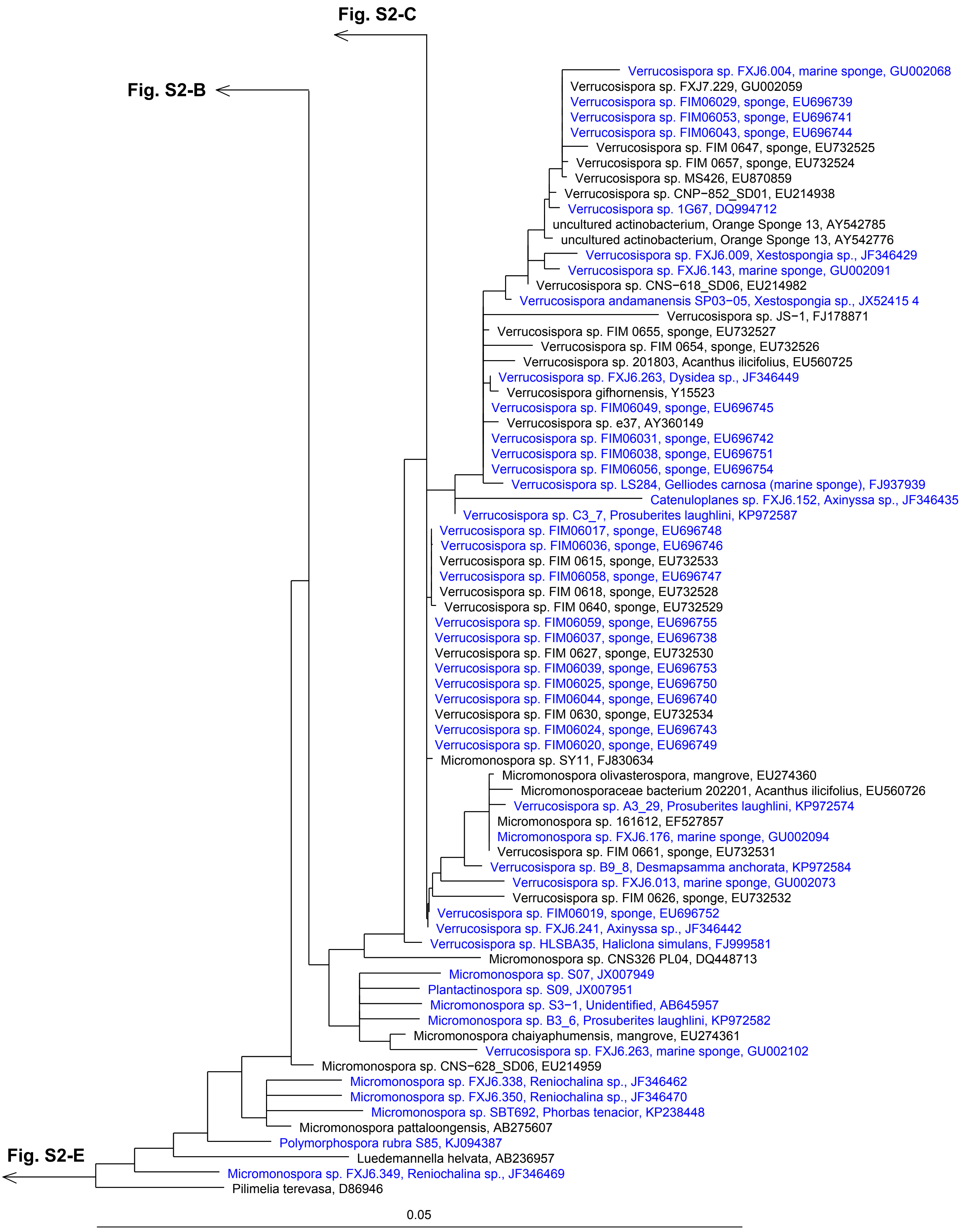

Figure S2-D. 16S rRNA gene-based phylogeny of sponge-associated Actinobacteria. Details are as provided for Figure S1

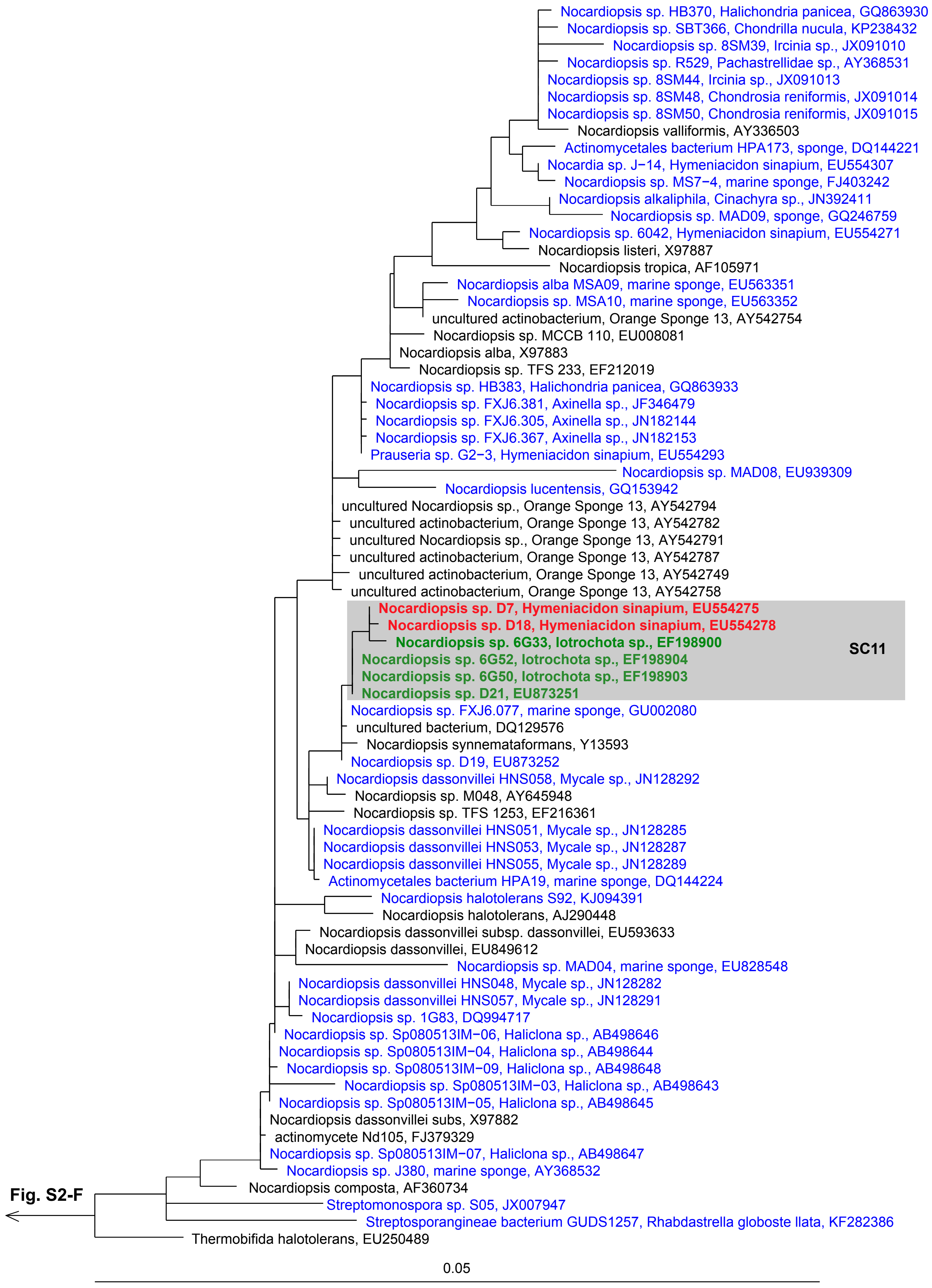

Figure S2-E. 16S rRNA gene-based phylogeny of sponge-associated Actinobacteria. Details are as provided for Figure S1

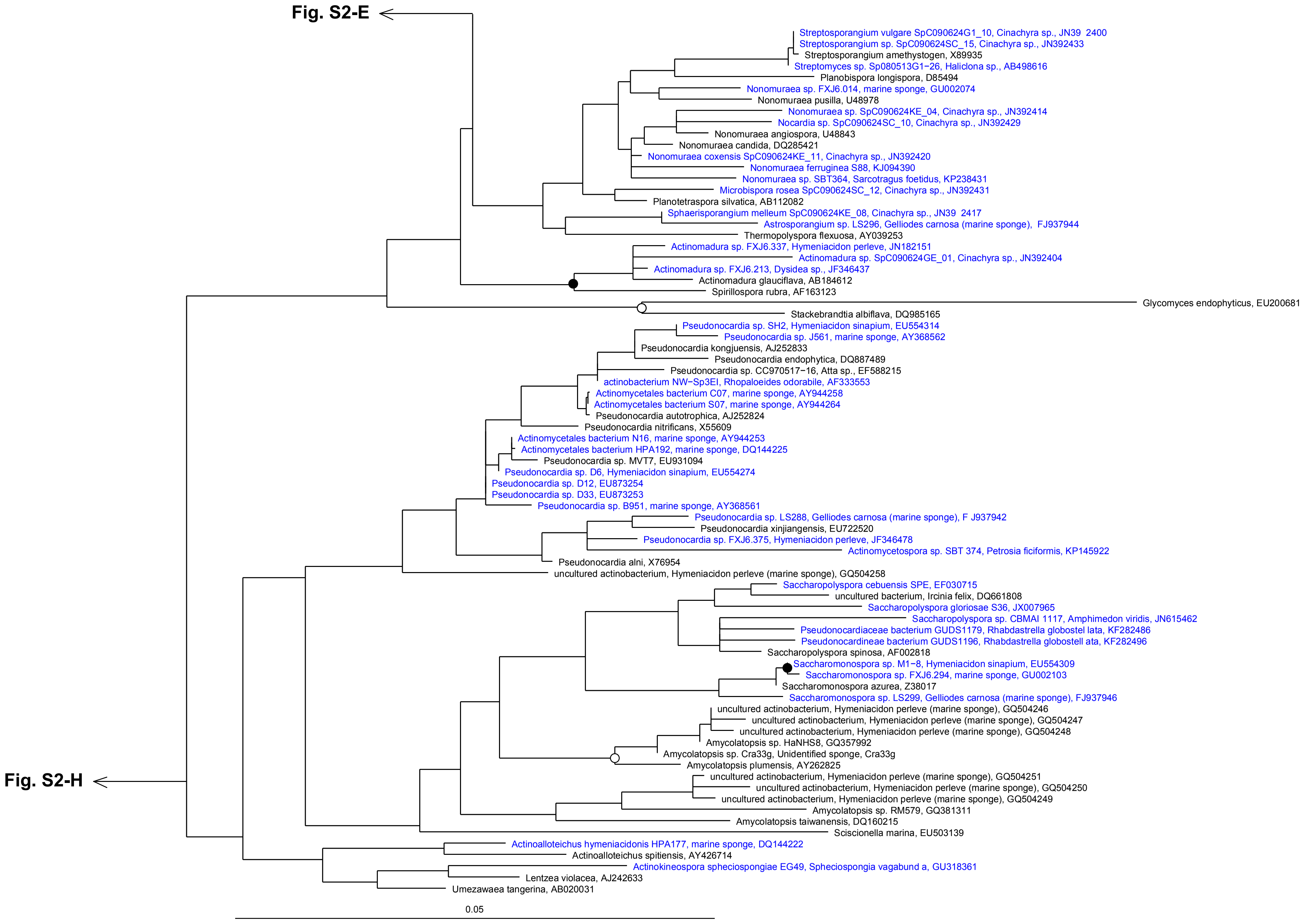

Figure S2-F. 16S rRNA gene-based phylogeny of sponge-associated Actinobacteria. Details are as provided for Figure S1

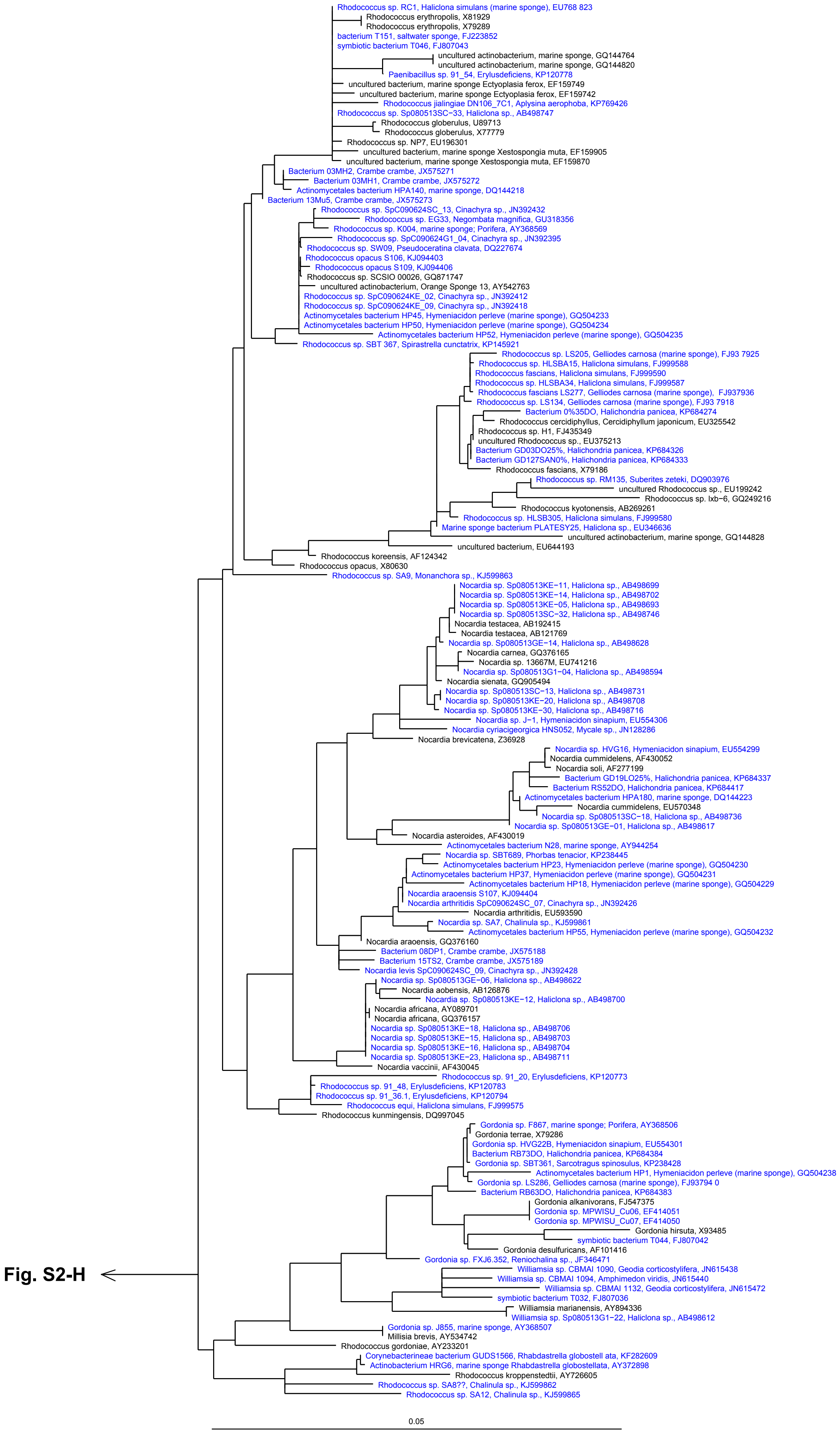

Figure S2-G. 16S rRNA gene-based phylogeny of sponge-associated Actinobacteria. Details are as provided for Figure S1

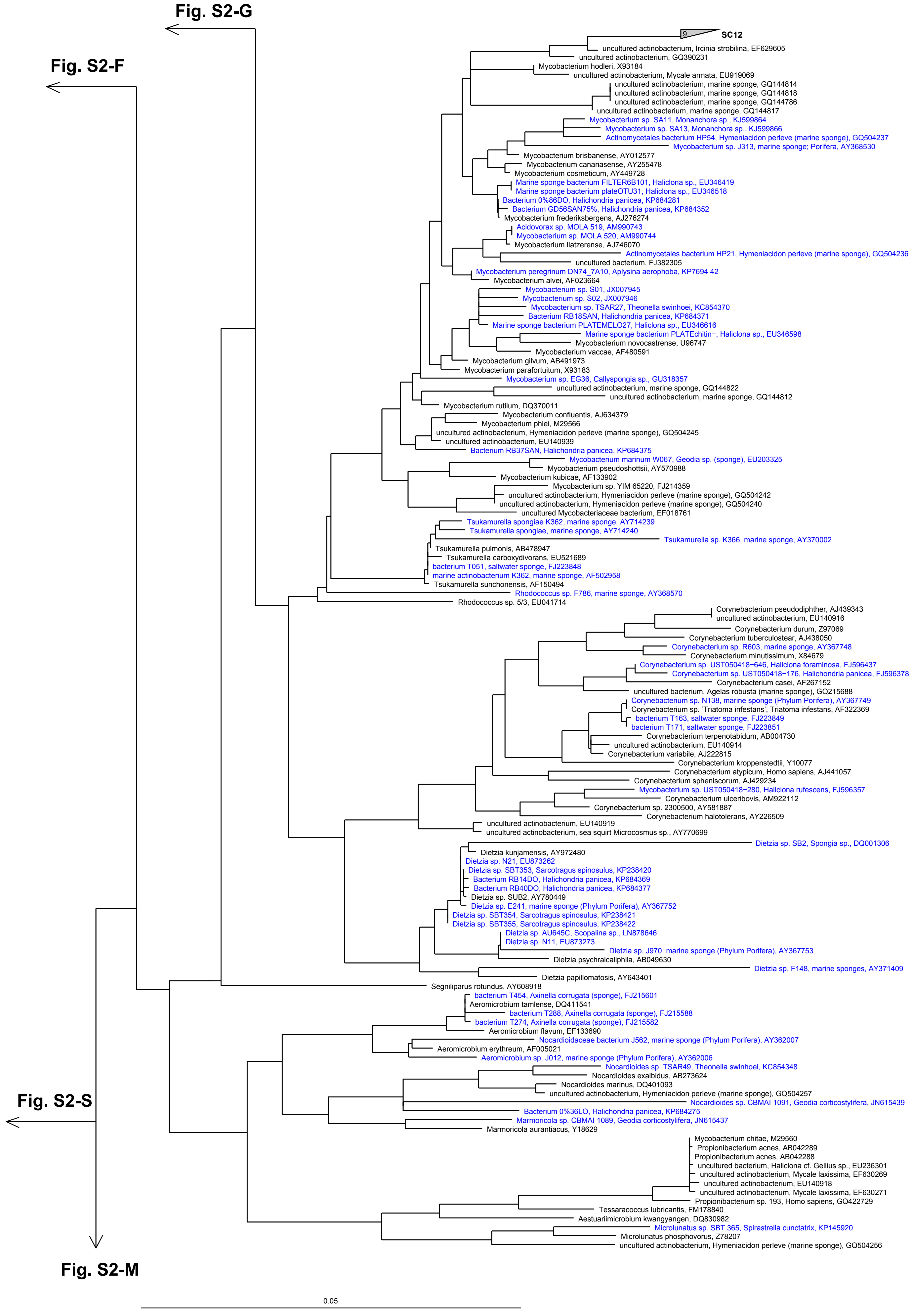

Figure S2-H. 16S rRNA gene-based phylogeny of sponge-associated Actinobacteria. Details are as provided for Figure S1

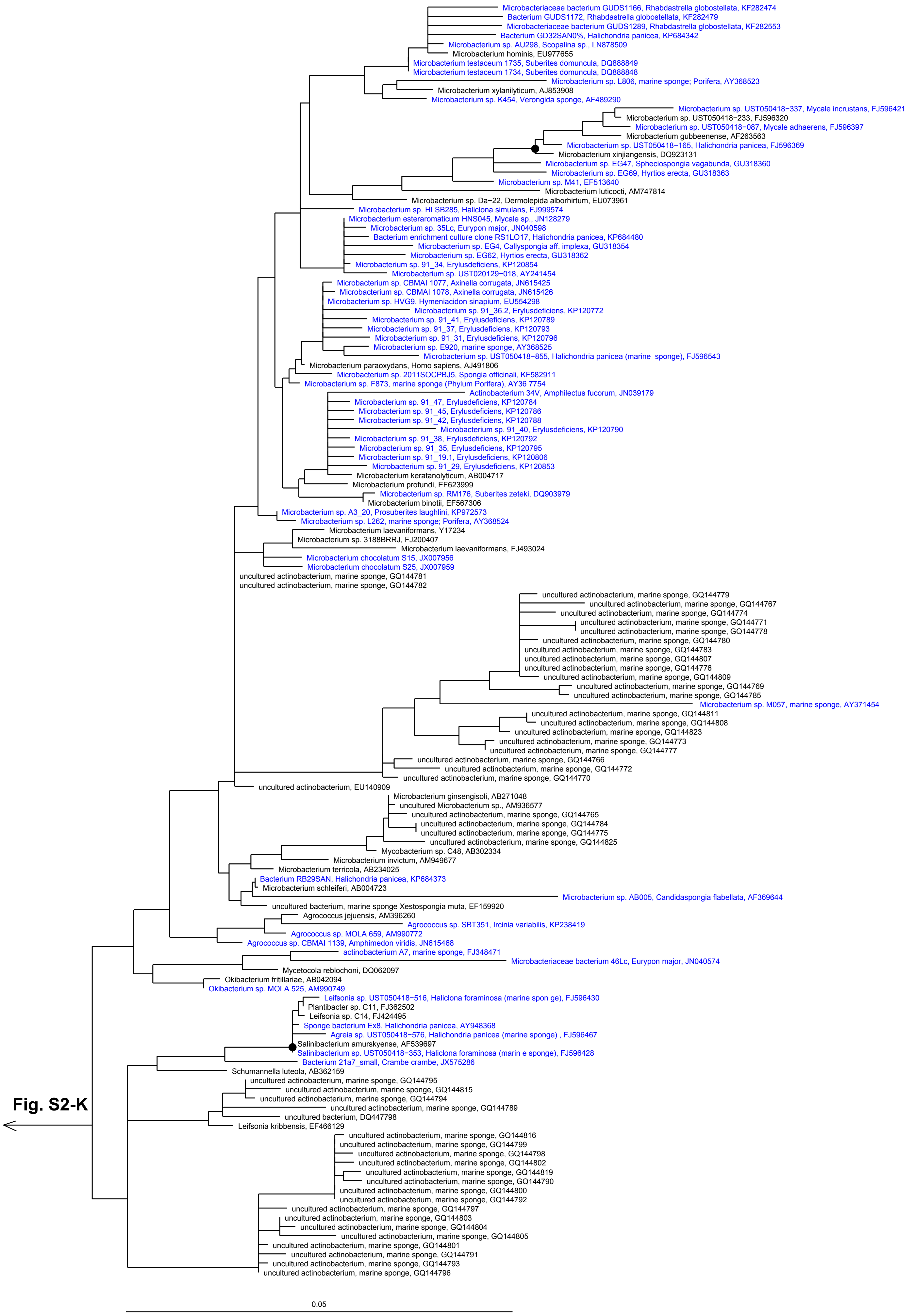

Figure S2-I. 16S rRNA gene-based phylogeny of sponge-associated Actinobacteria. Details are as provided for Figure S1

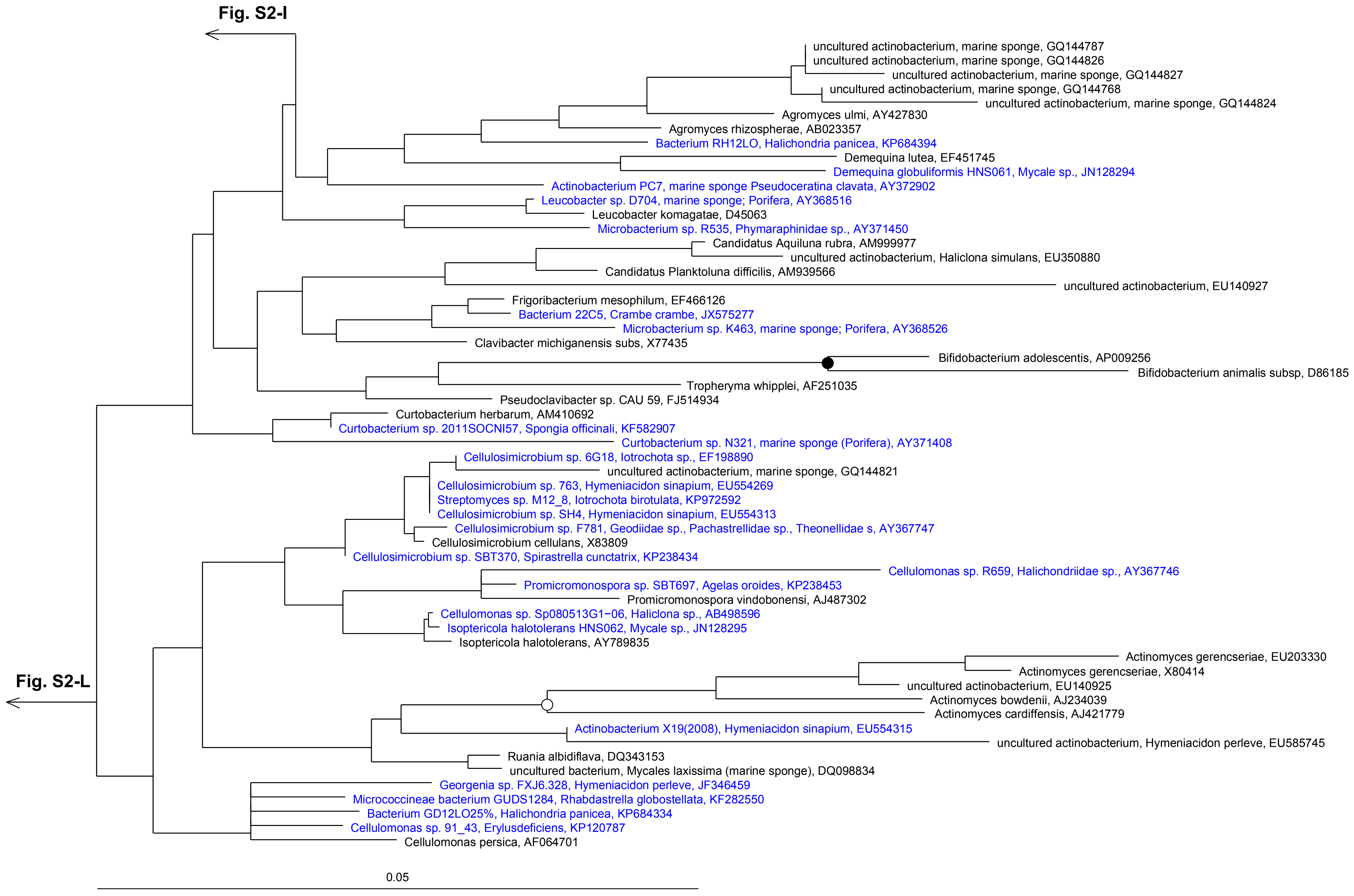

Figure S2-K. 16S rRNA gene-based phylogeny of sponge-associated Actinobacteria. Details are as provided for Figure S1

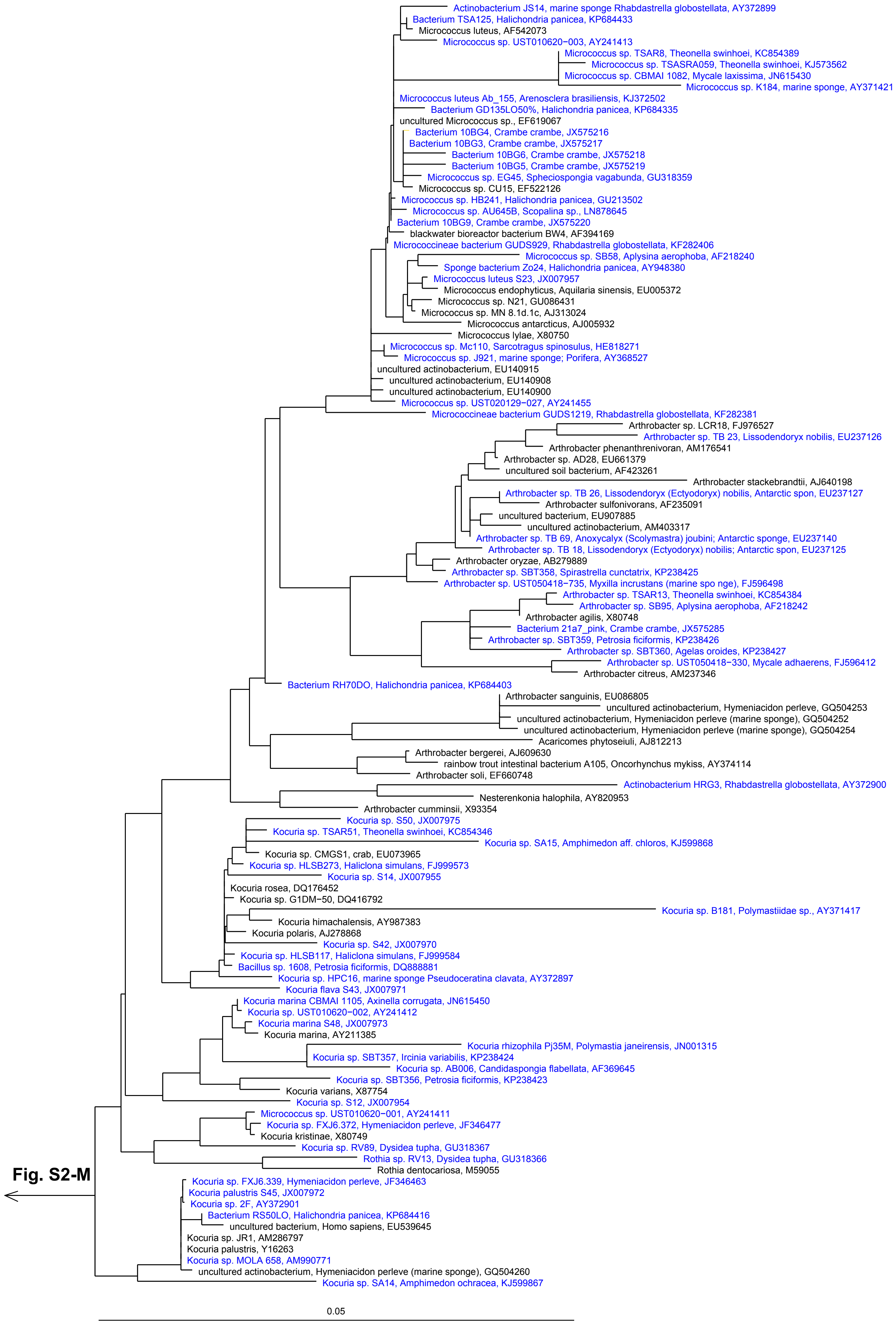

Figure S2-L. 16S rRNA gene-based phylogeny of sponge-associated Actinobacteria. Details are as provided for Figure S1

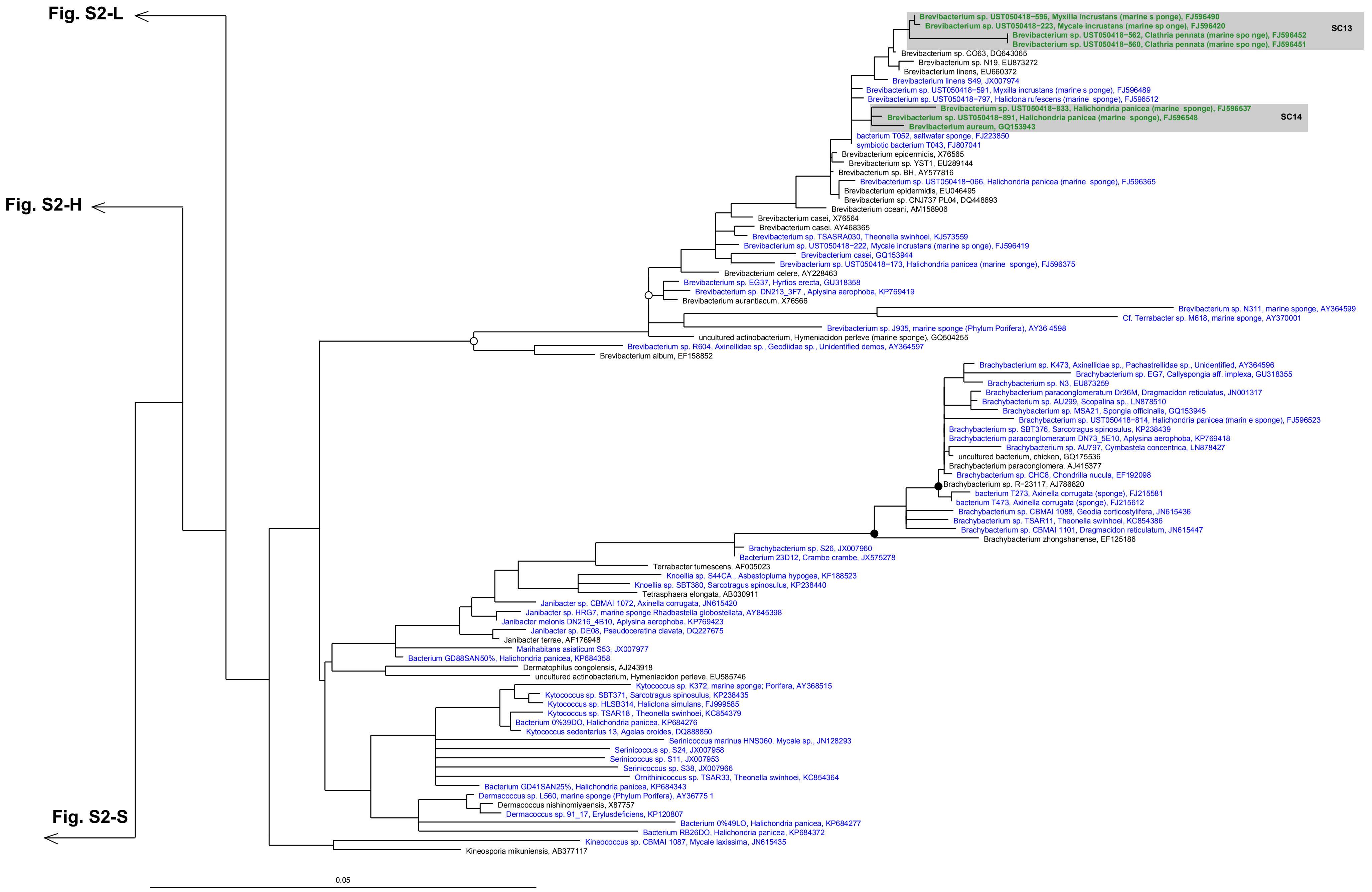

Figure S2-M. 16S rRNA gene-based phylogeny of sponge-associated Actinobacteria. Details are as provided for Figure S1

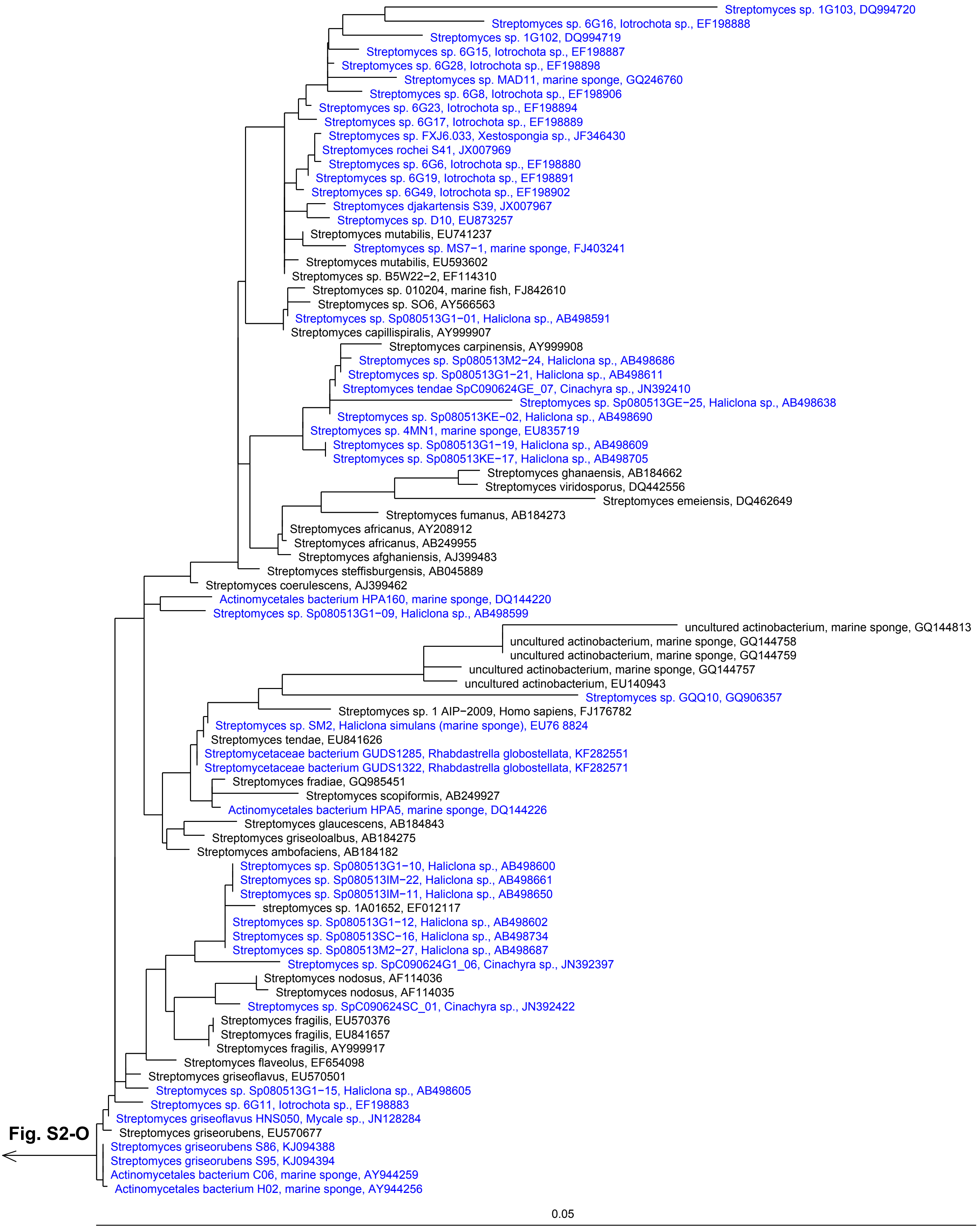

Figure S2-N. 16S rRNA gene-based phylogeny of sponge-associated Actinobacteria. Details are as provided for Figure S1

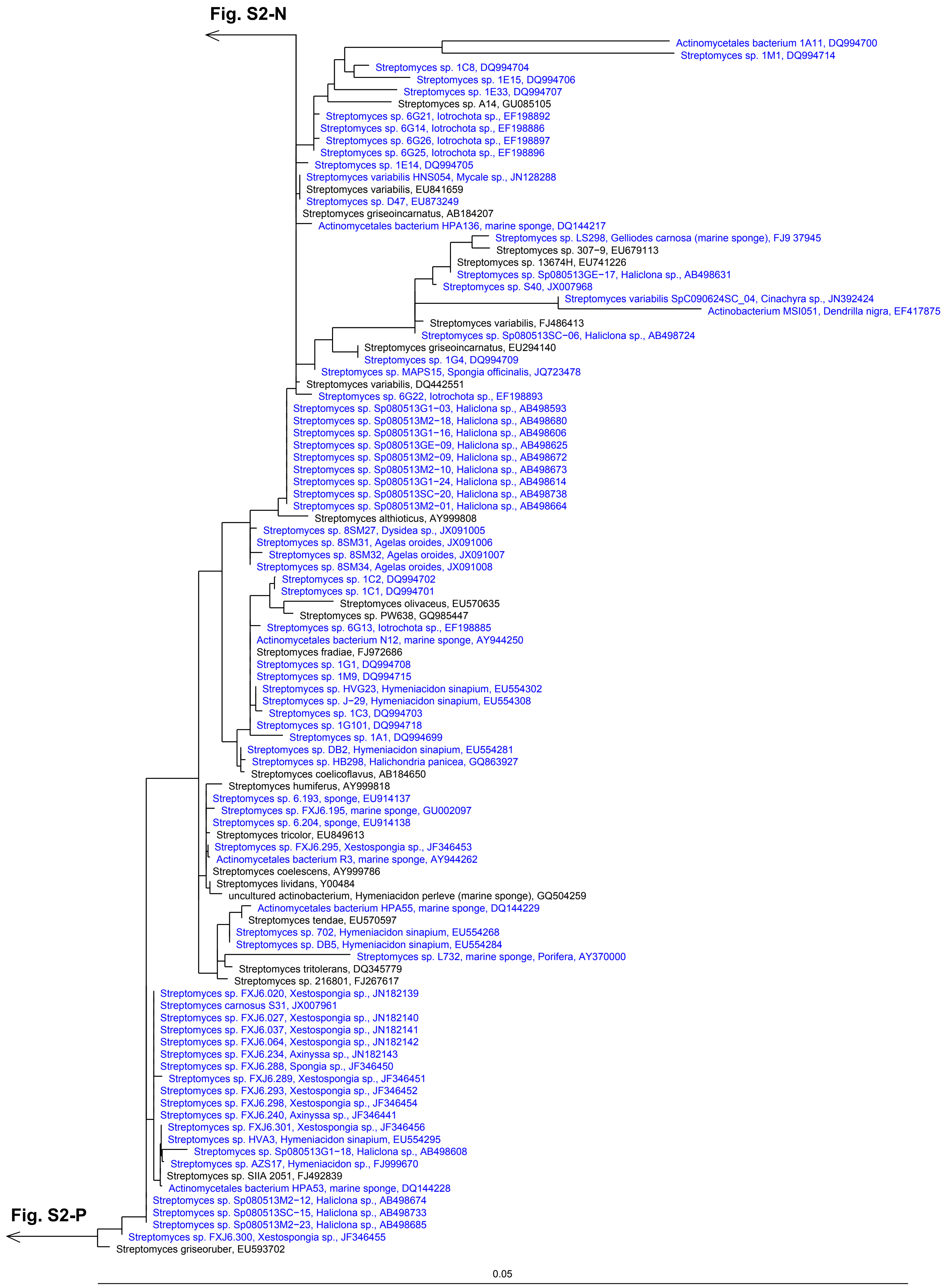

Figure S2-O. 16S rRNA gene-based phylogeny of sponge-associated Actinobacteria. Details are as provided for Figure S1

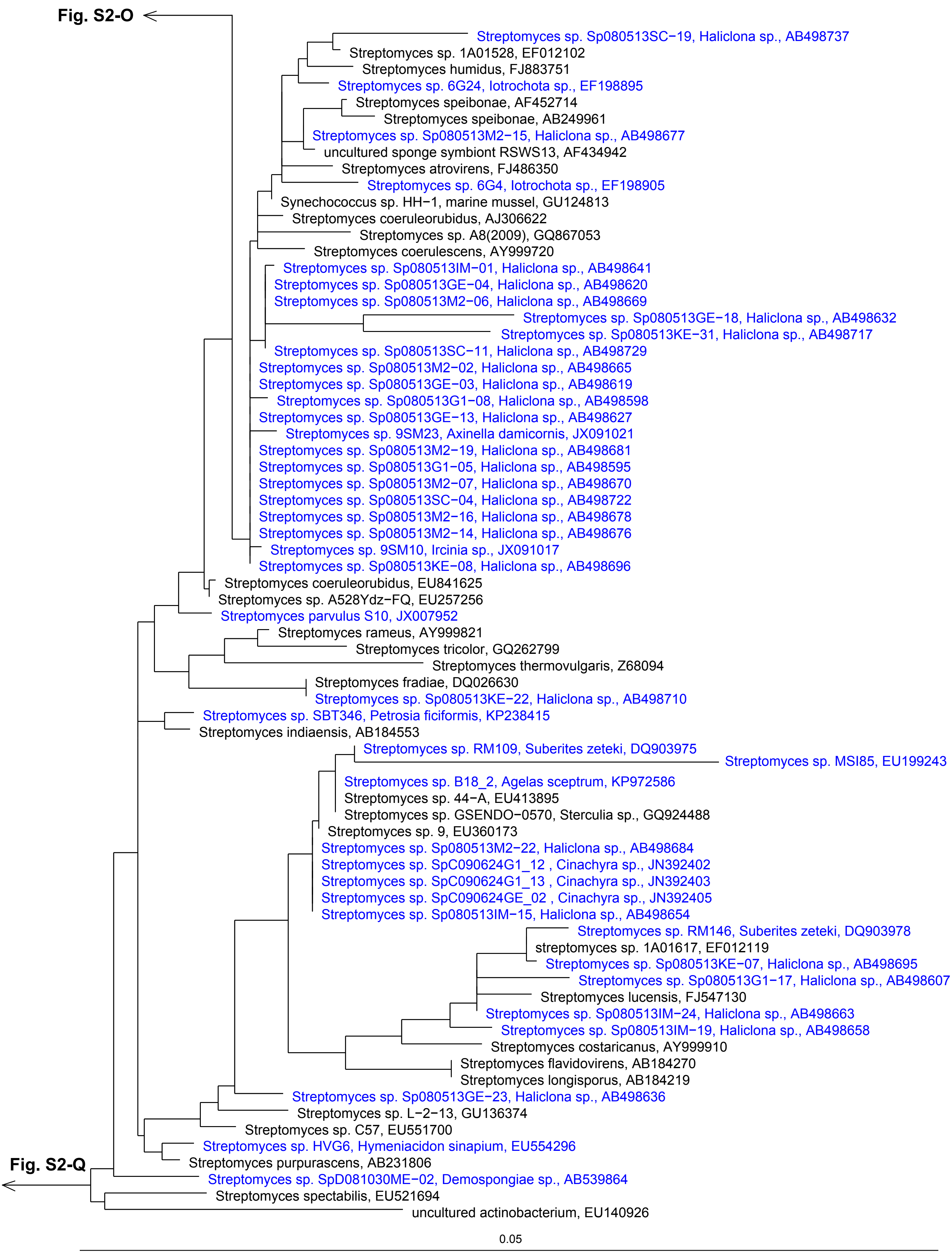

Figure S2-P. 16S rRNA gene-based phylogeny of sponge-associated Actinobacteria. Details are as provided for Figure S1

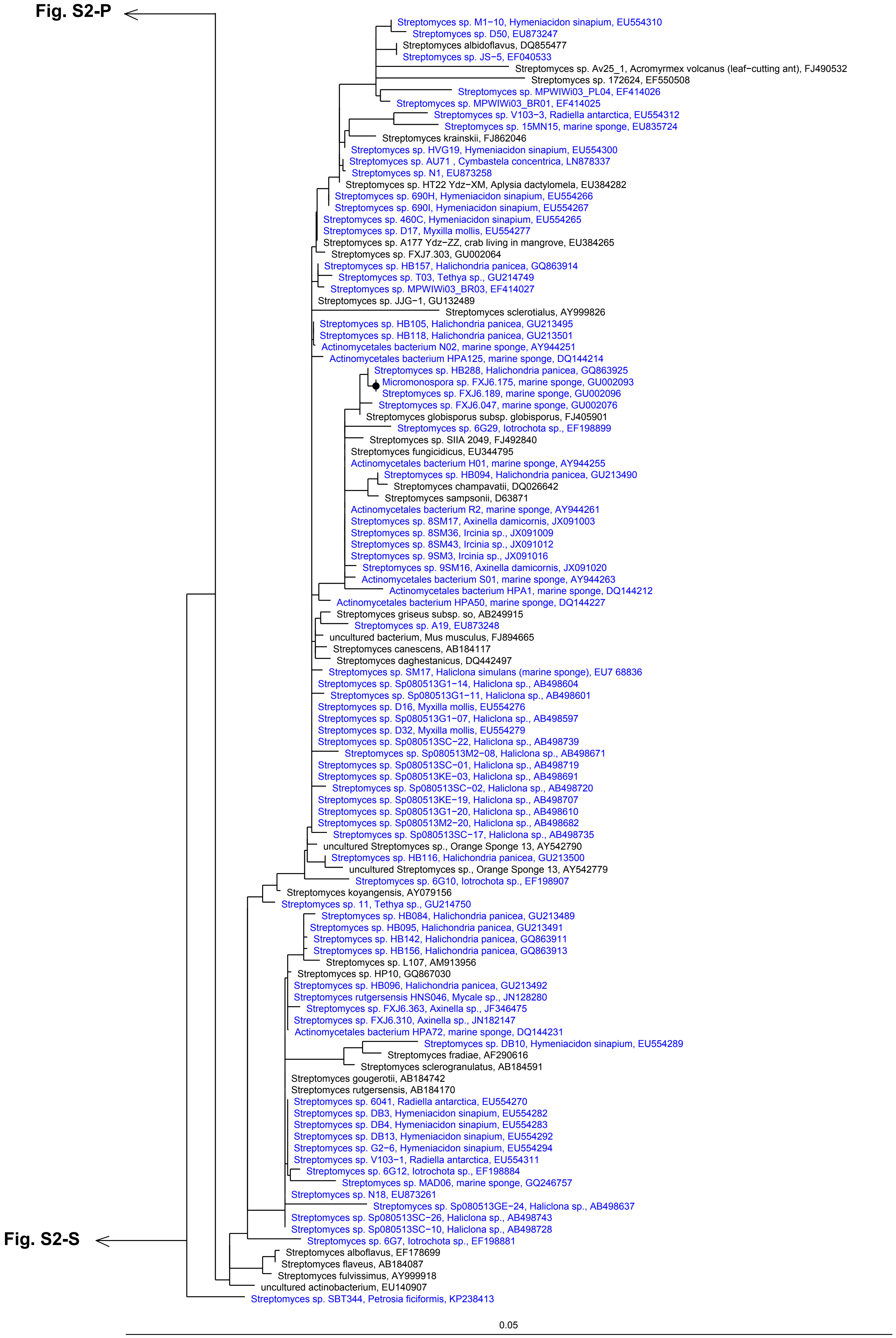

Figure S2-Q. 16S rRNA gene-based phylogeny of sponge-associated Actinobacteria. Details are as provided for Figure S1

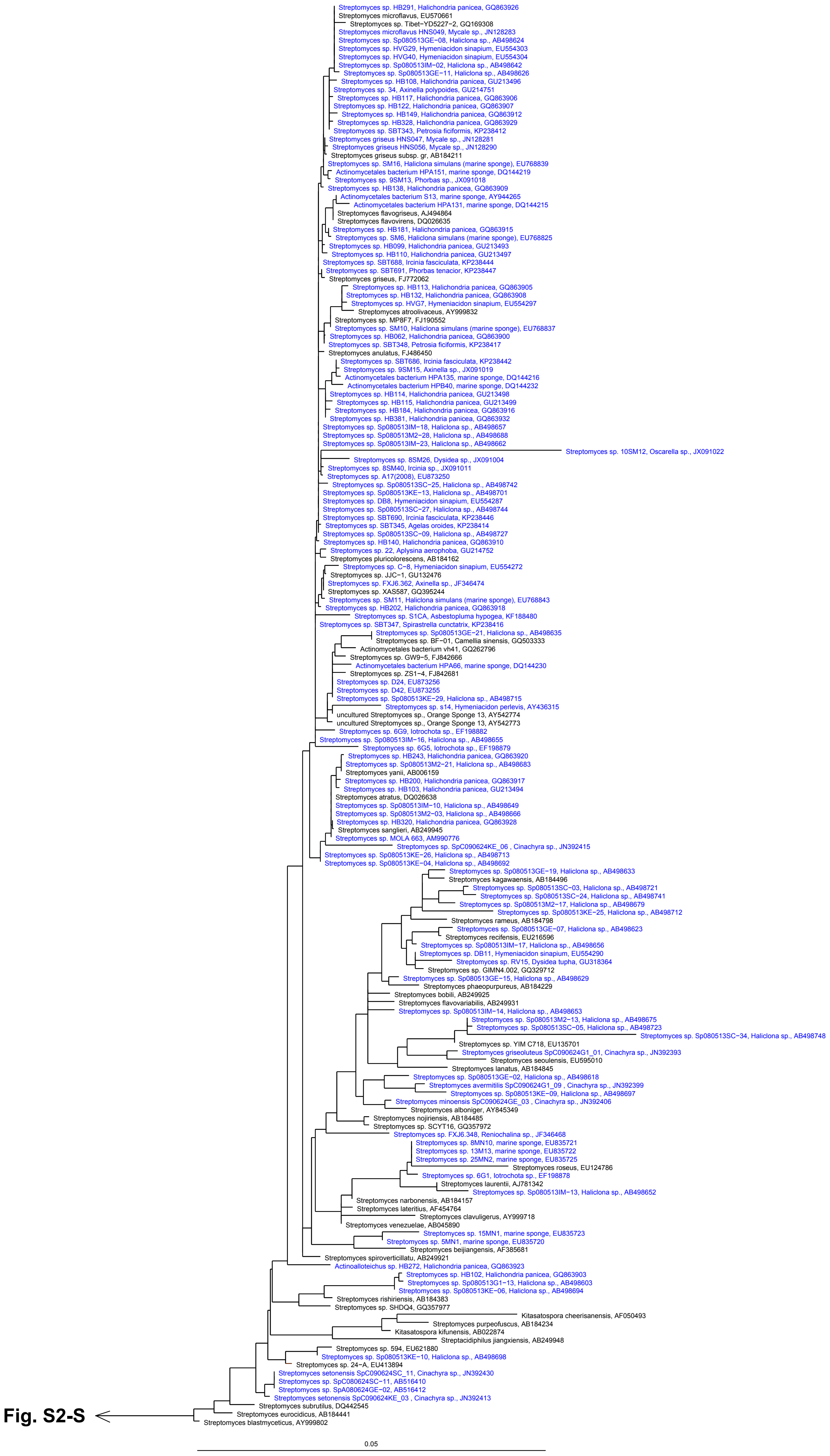

Figure S2-R. 16S rRNA gene-based phylogeny of sponge-associated Actinobacteria. Details are as provided for Figure S1

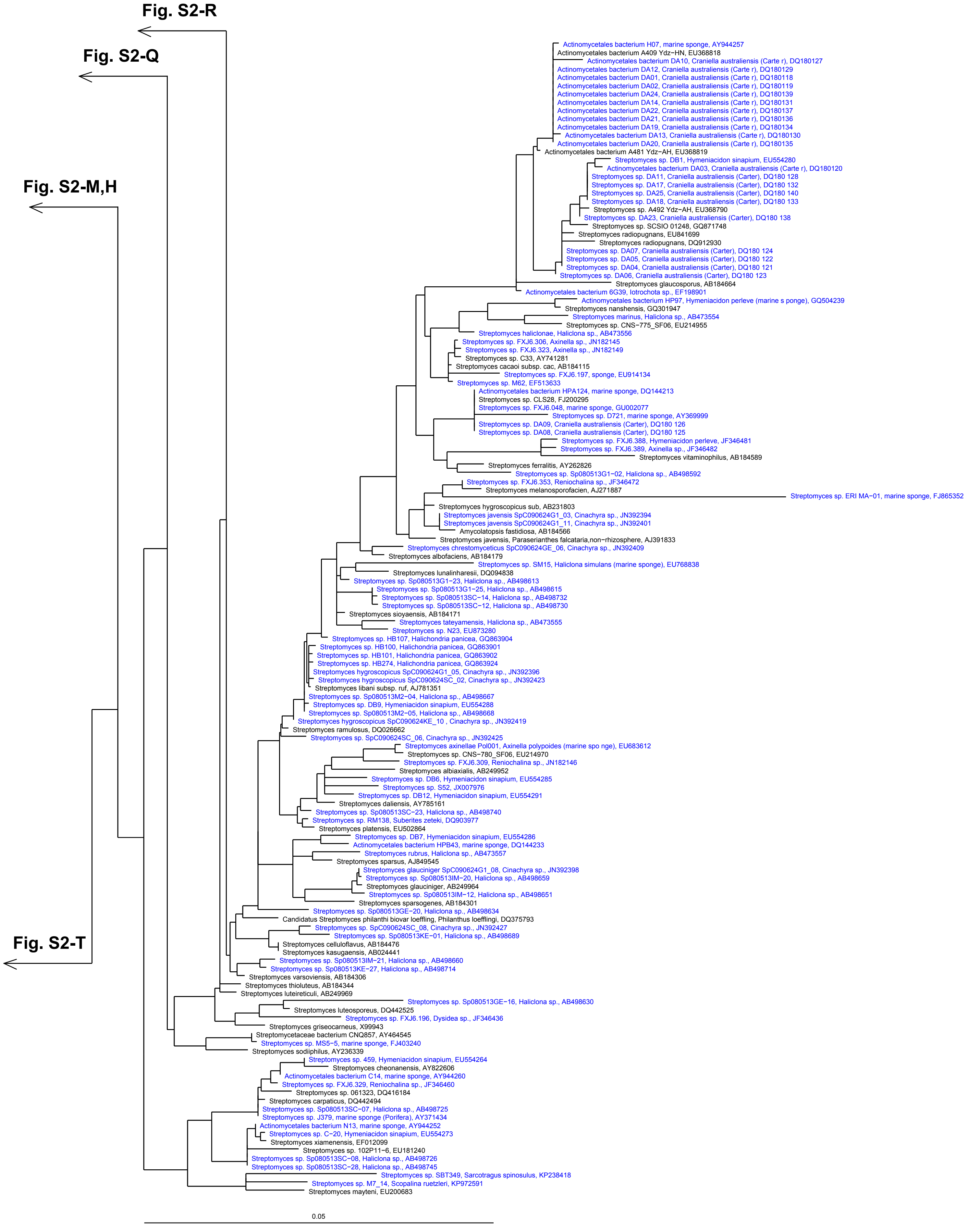

Figure S2-S. 16S rRNA gene-based phylogeny of sponge-associated Actinobacteria. Details are as provided for Figure S1

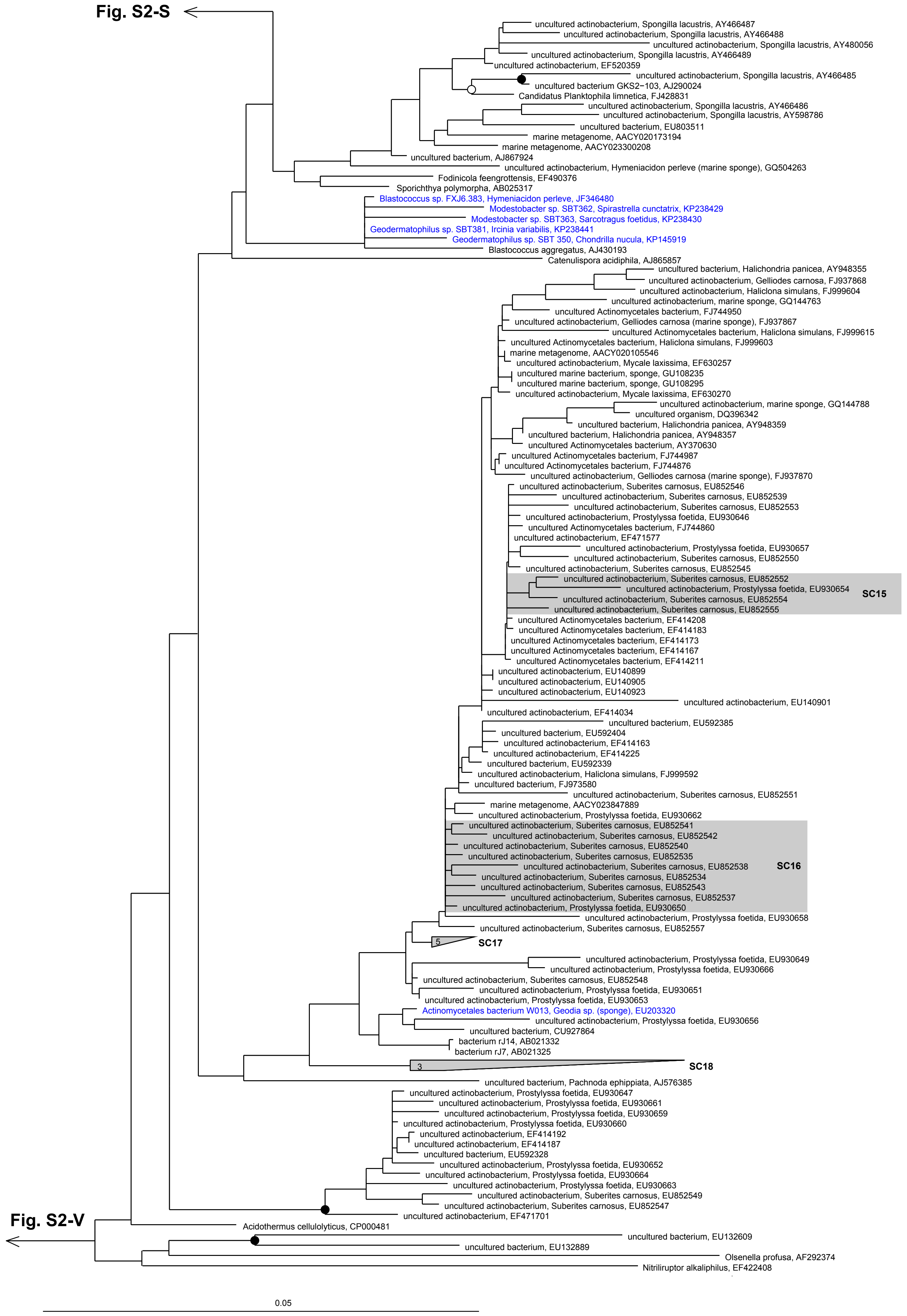

Figure S2-T. 16S rRNA gene-based phylogeny of sponge-associated Actinobacteria. Details are as provided for Figure S1

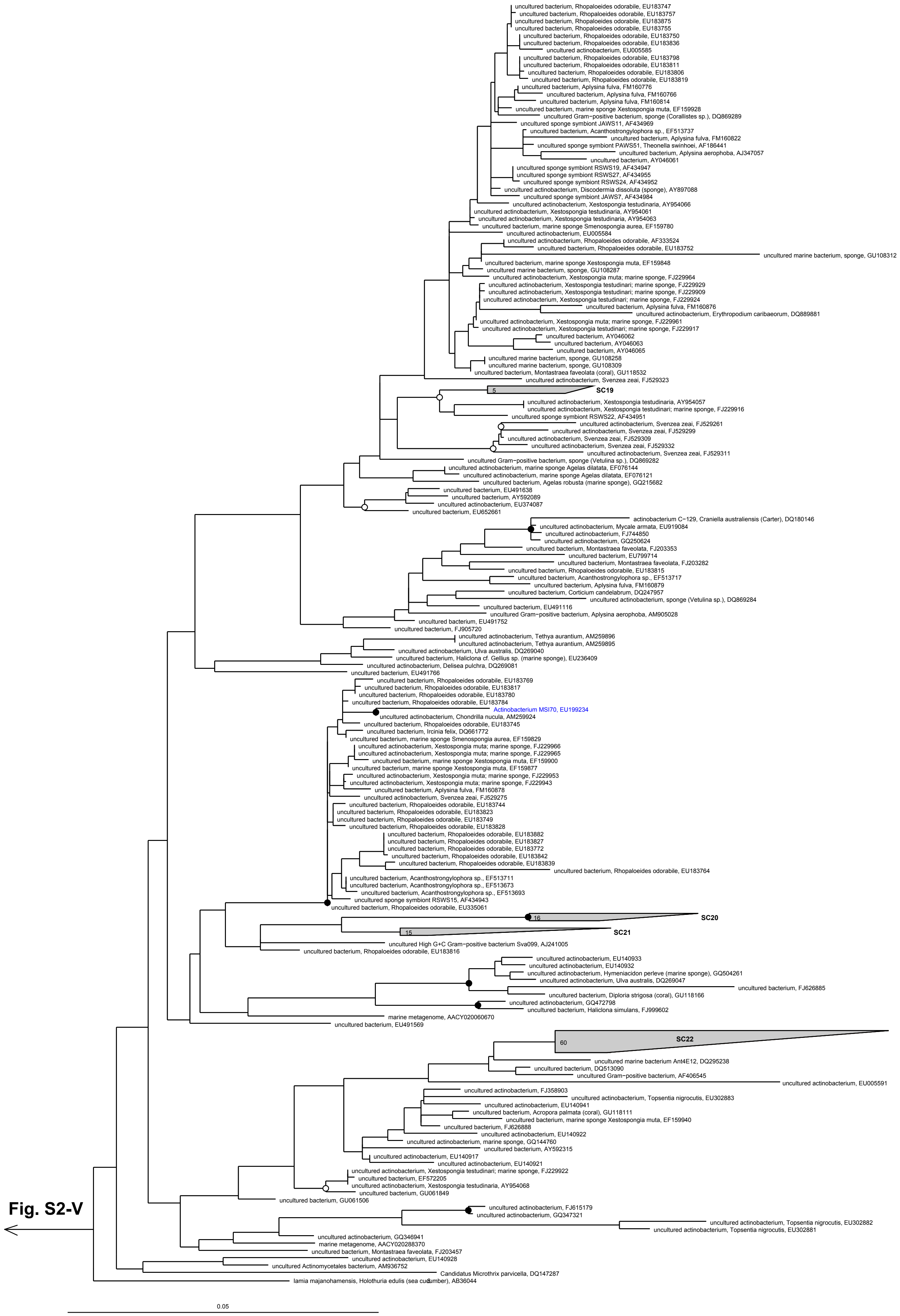

Figure S2-U. 16S rRNA gene-based phylogeny of sponge-associated Actinobacteria. Details are as provided for Figure S1

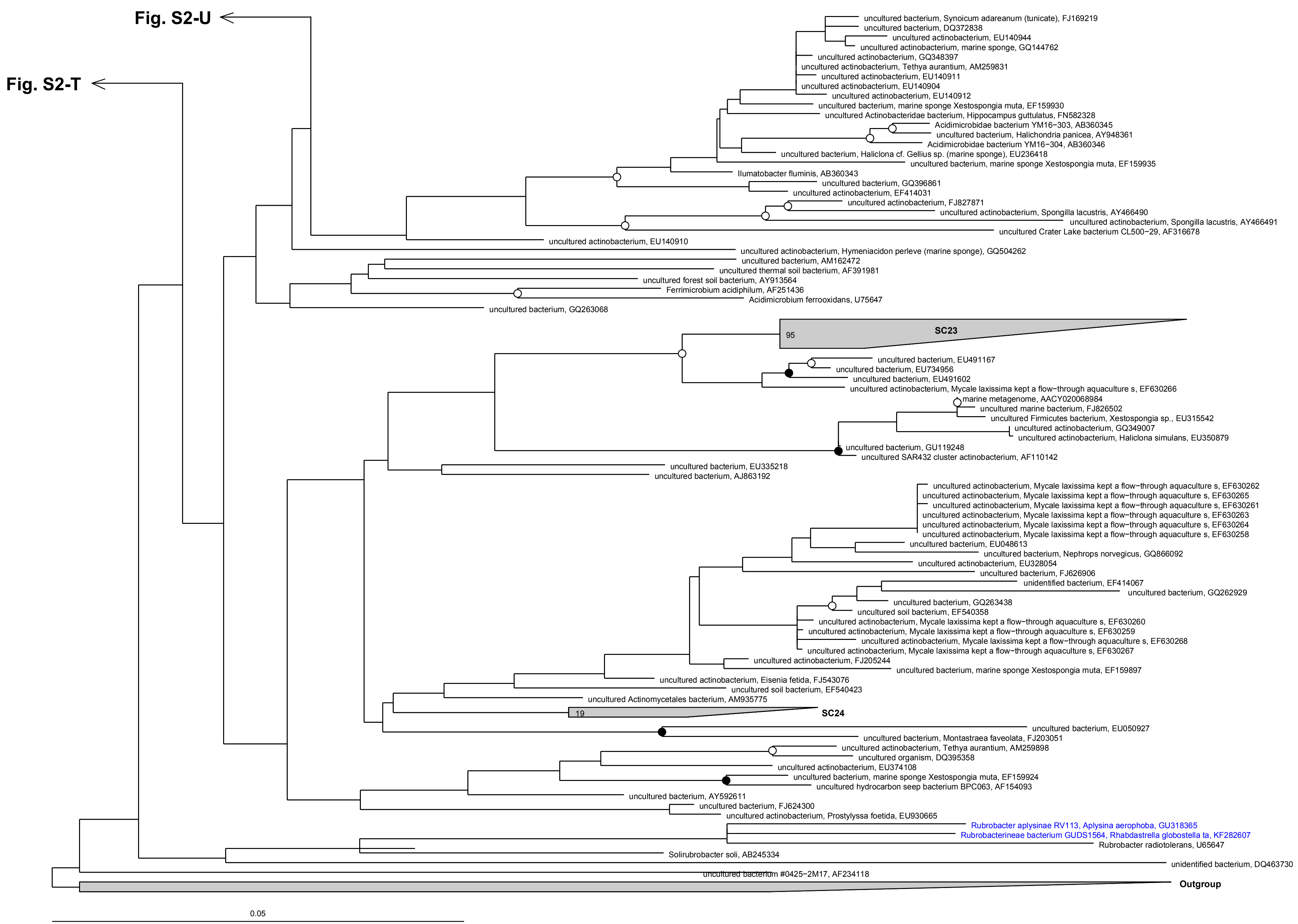

Figure S2-V. 16S rRNA gene-based phylogeny of sponge-associated Actinobacteria. Details are as provided for Figure S1
