# Supplementary material for: Bacteria Cultivated From Sponges and Bacteria Not Yet Cultivated From Sponges—A Review
Source: Front Microbiol. 2021 Nov 10;12:737925. doi: 10.3389/fmicb.2021.737925 (PMC8634882; doi:10.3389/fmicb.2021.737925)
Supplement: Supplementary file 3 [file Image_3.pdf]

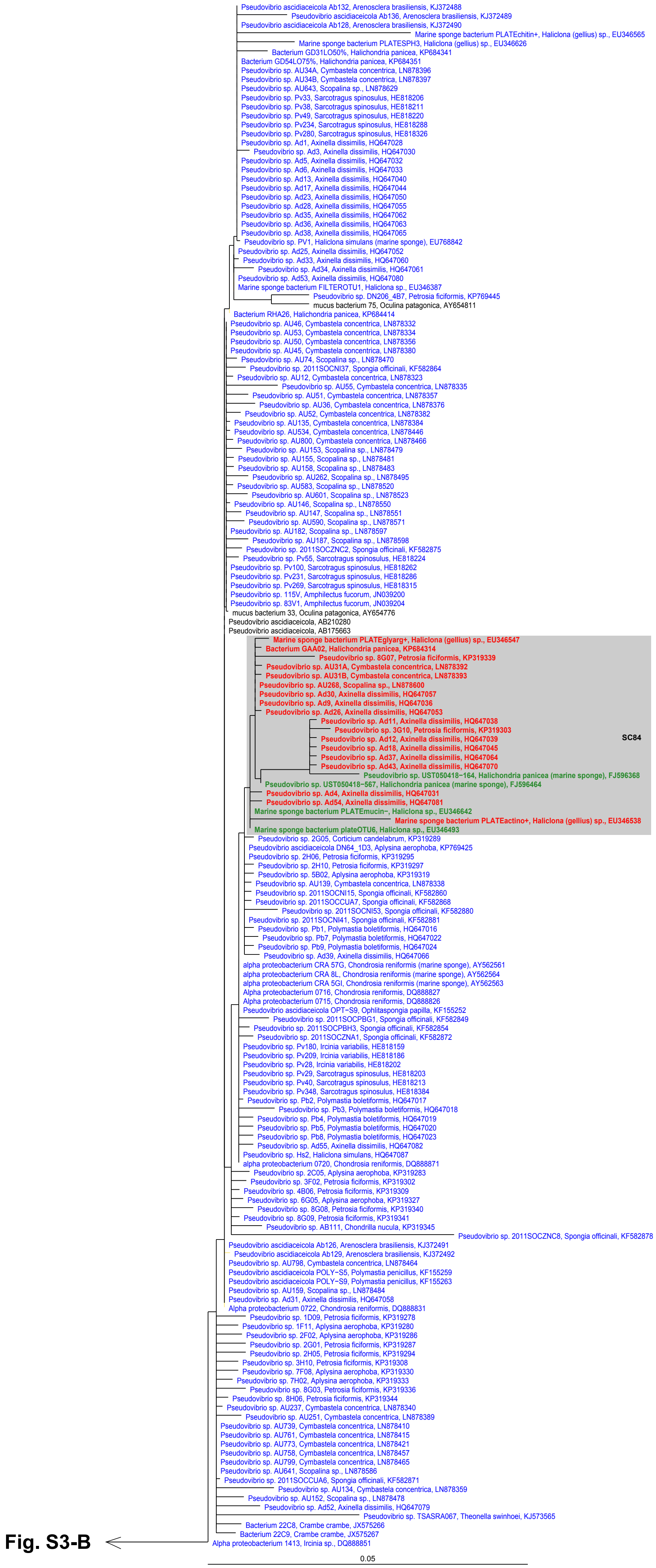

Figure S3-A. 16S rRNA gene-based phylogeny of sponge-associated Alphaproteobacteria. Details are as provided for Figure S1

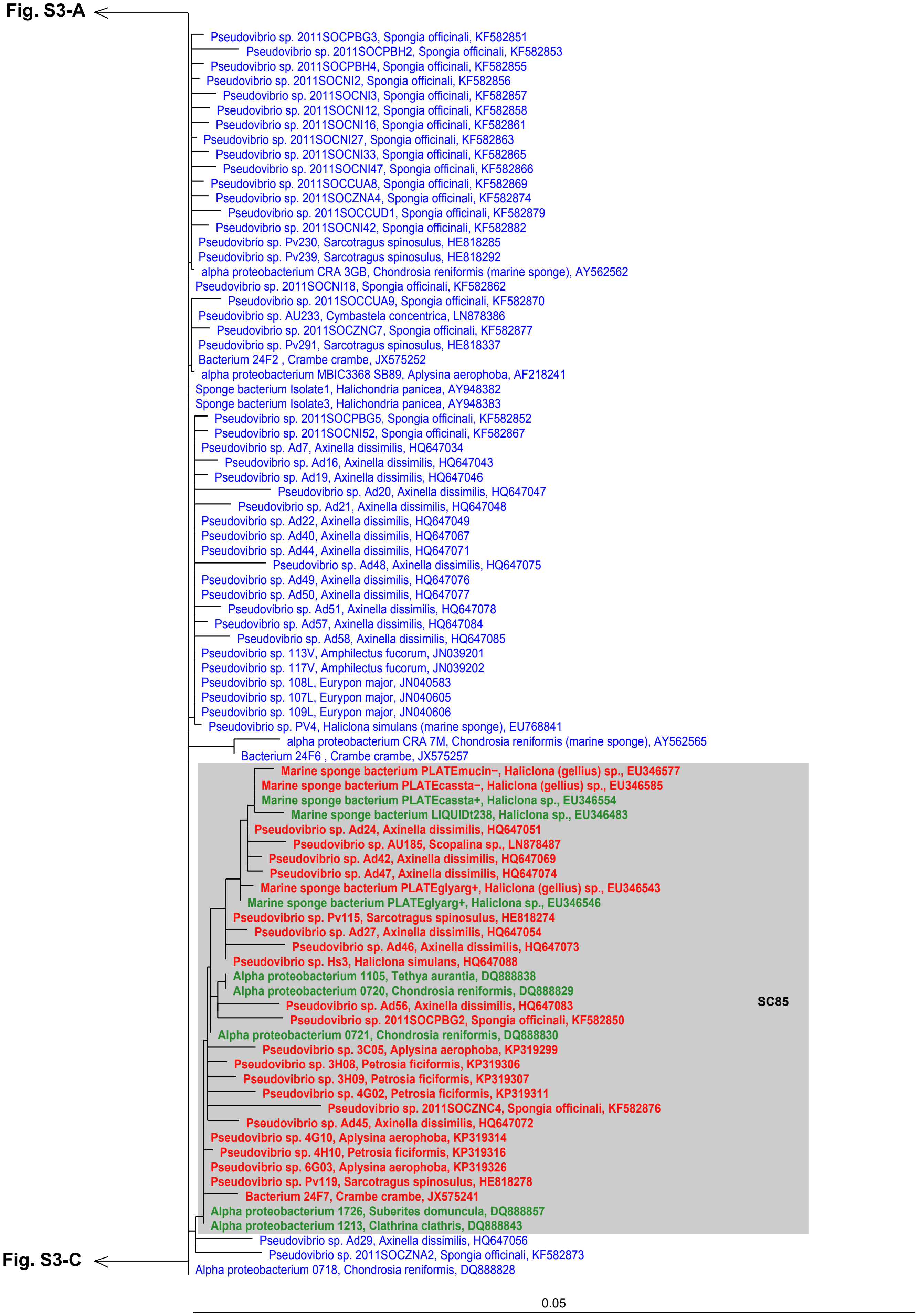

Figure S3-B. 16S rRNA gene-based phylogeny of sponge-associated Alphaproteobacteria. Details are as provided for Figure S1

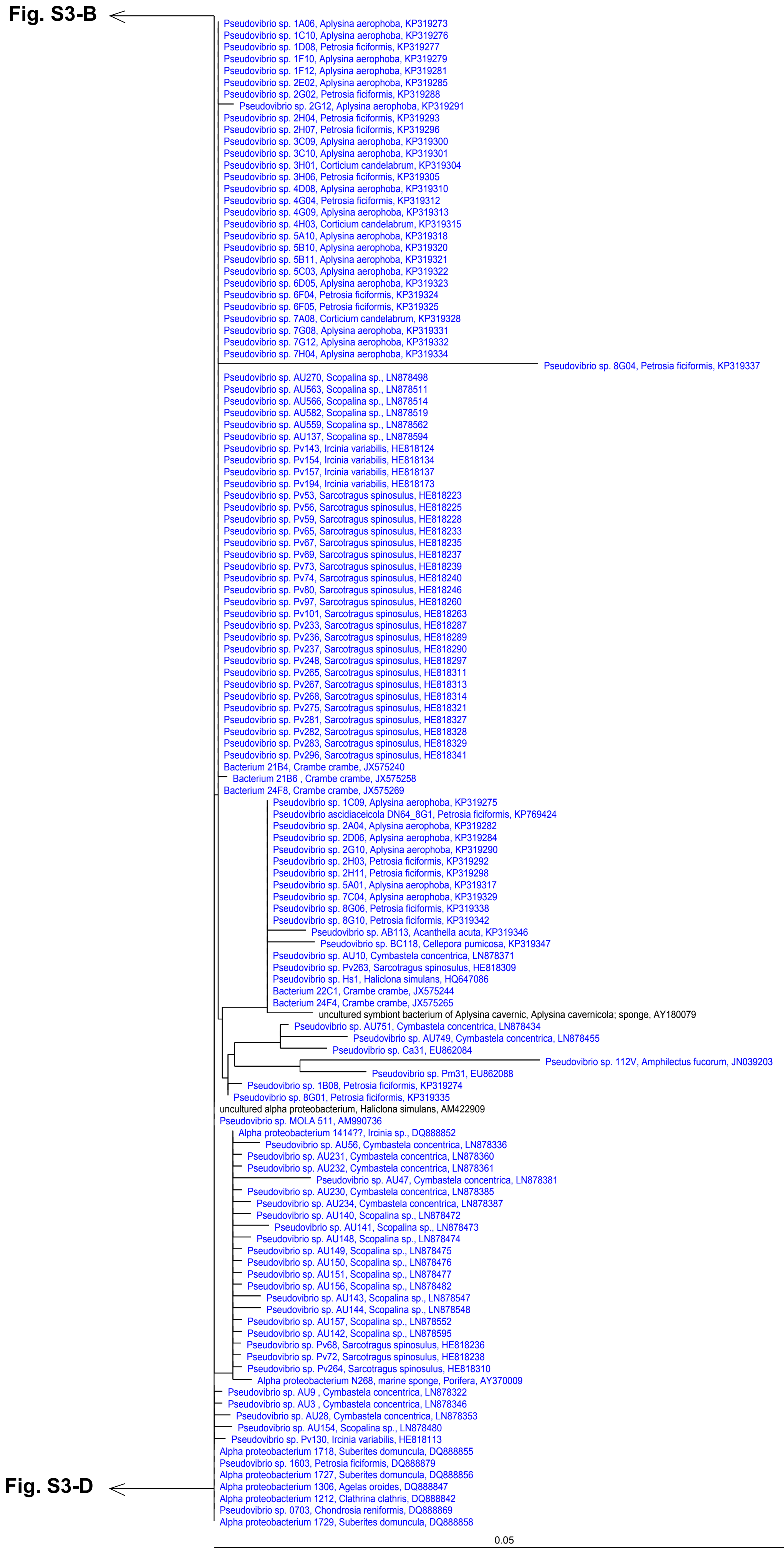

Figure S3-C. 16S rRNA gene-based phylogeny of sponge-associated Alphaproteobacteria. Details are as provided for Figure S1

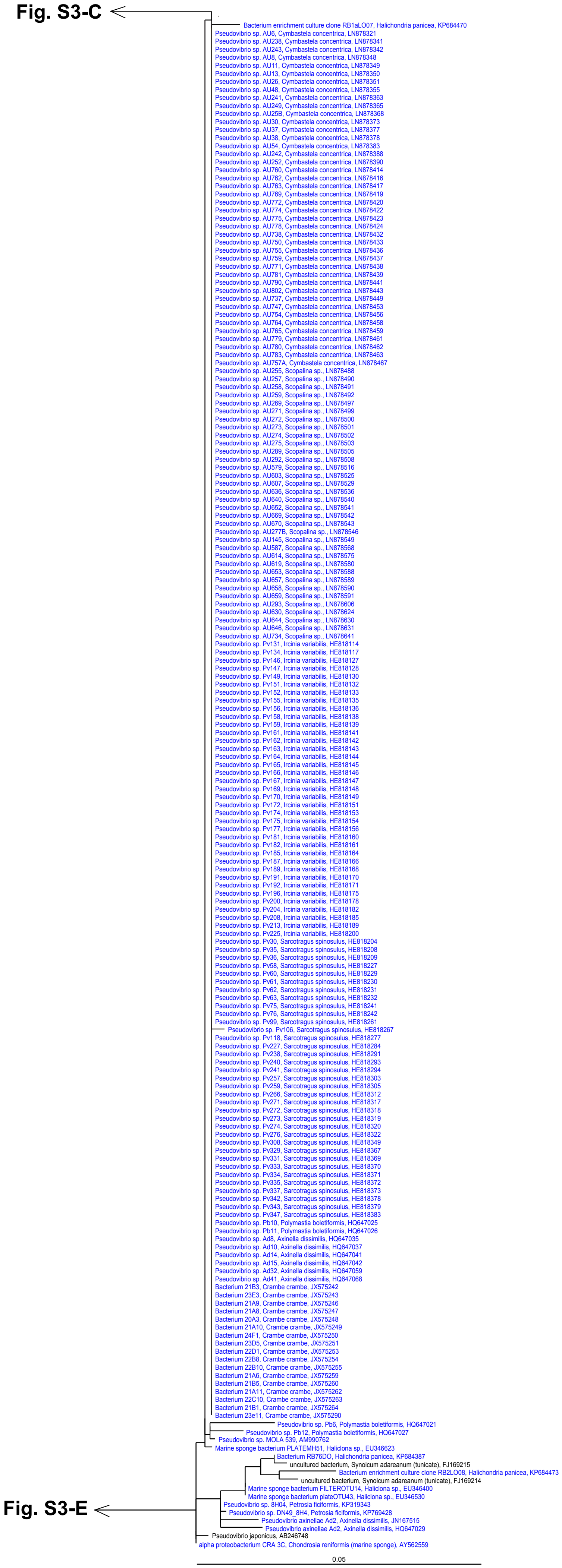

Figure S3-D. 16S rRNA gene-based phylogeny of sponge-associated Alphaproteobacteria. Details are as provided for Figure S1

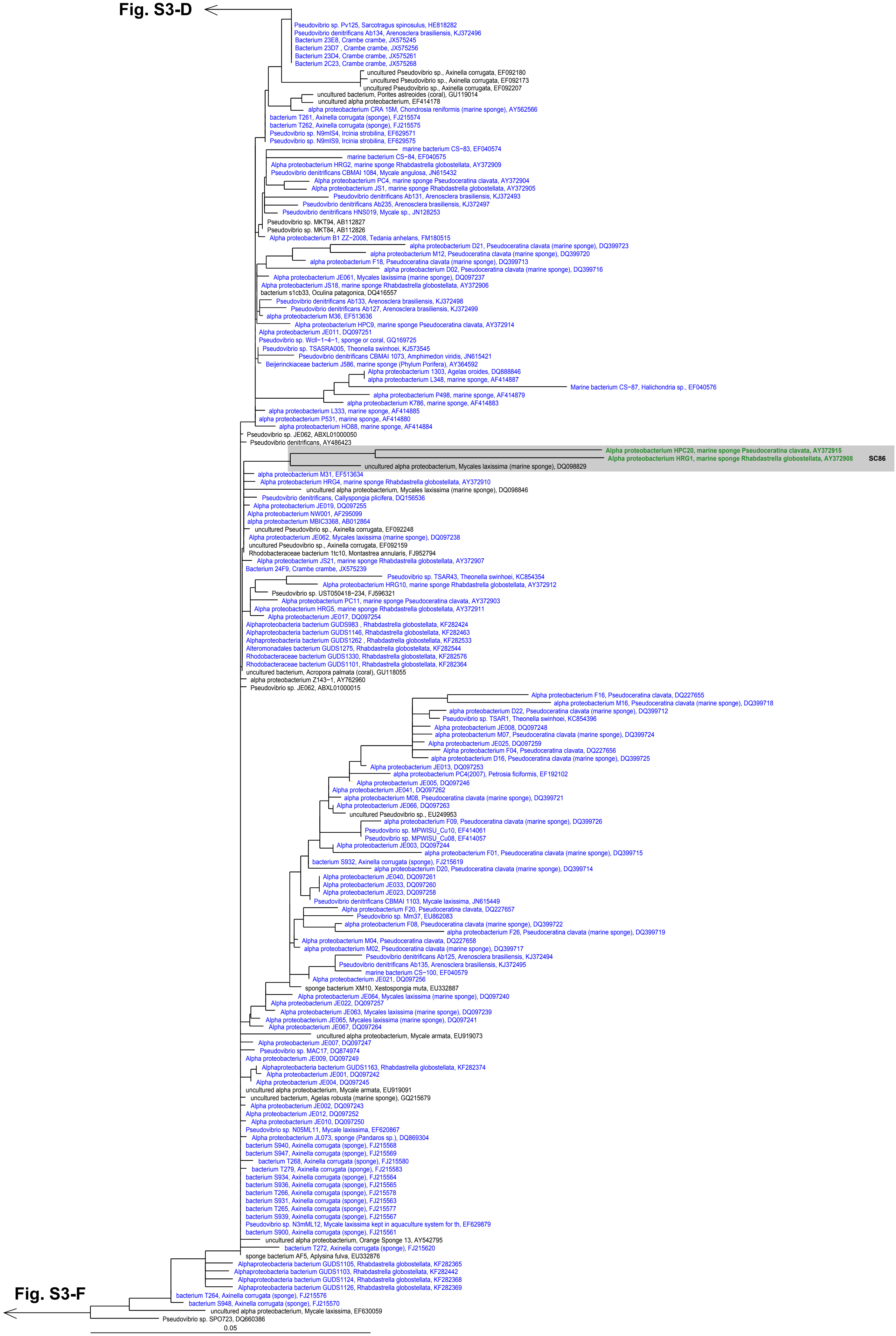

Figure S3-E. 16S rRNA gene-based phylogeny of sponge-associated Alphaproteobacteria. Details are as provided for Figure S1

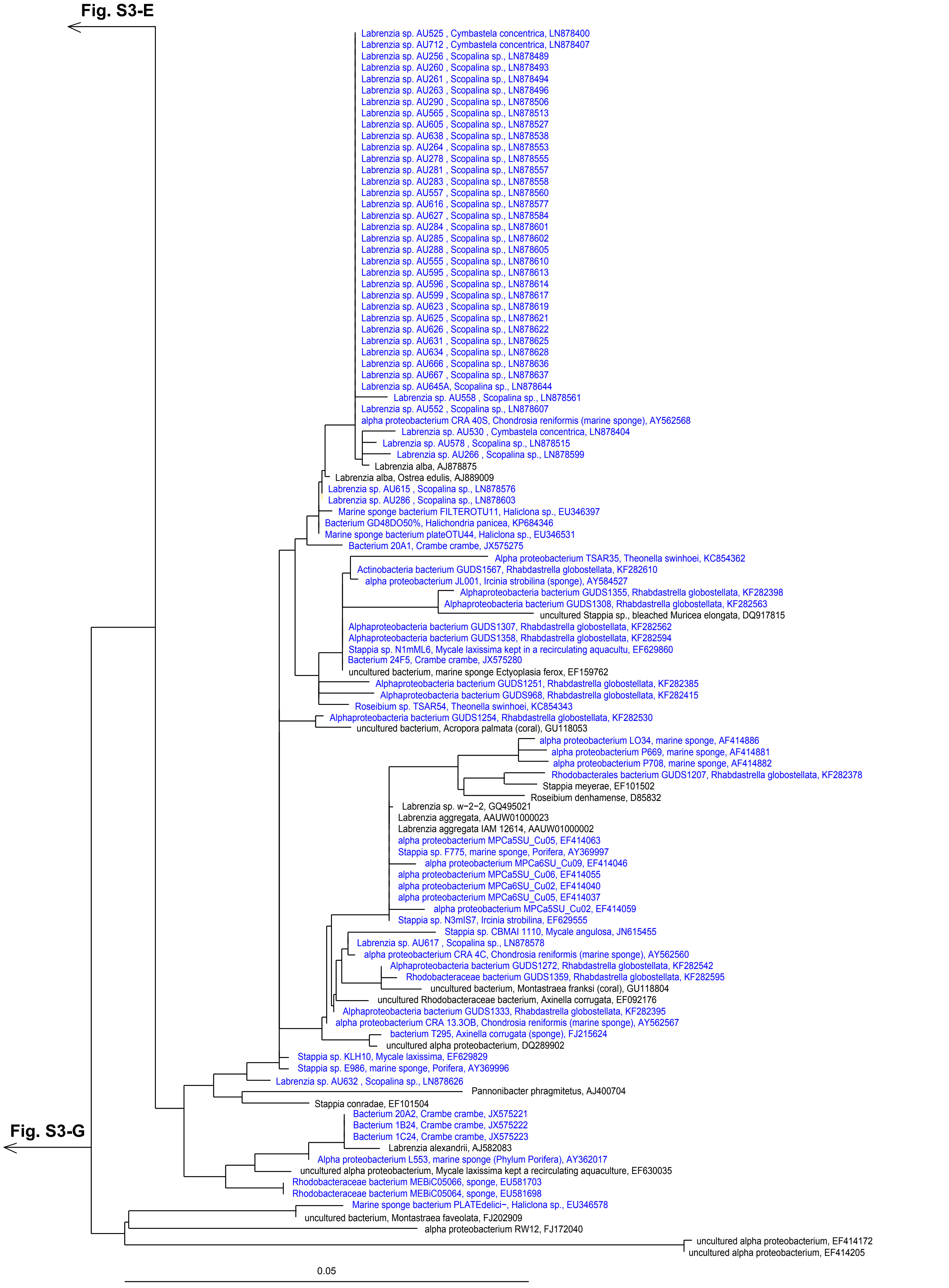

Figure S3-F. 16S rRNA gene-based phylogeny of sponge-associated Alphaproteobacteria. Details are as provided for Figure S1

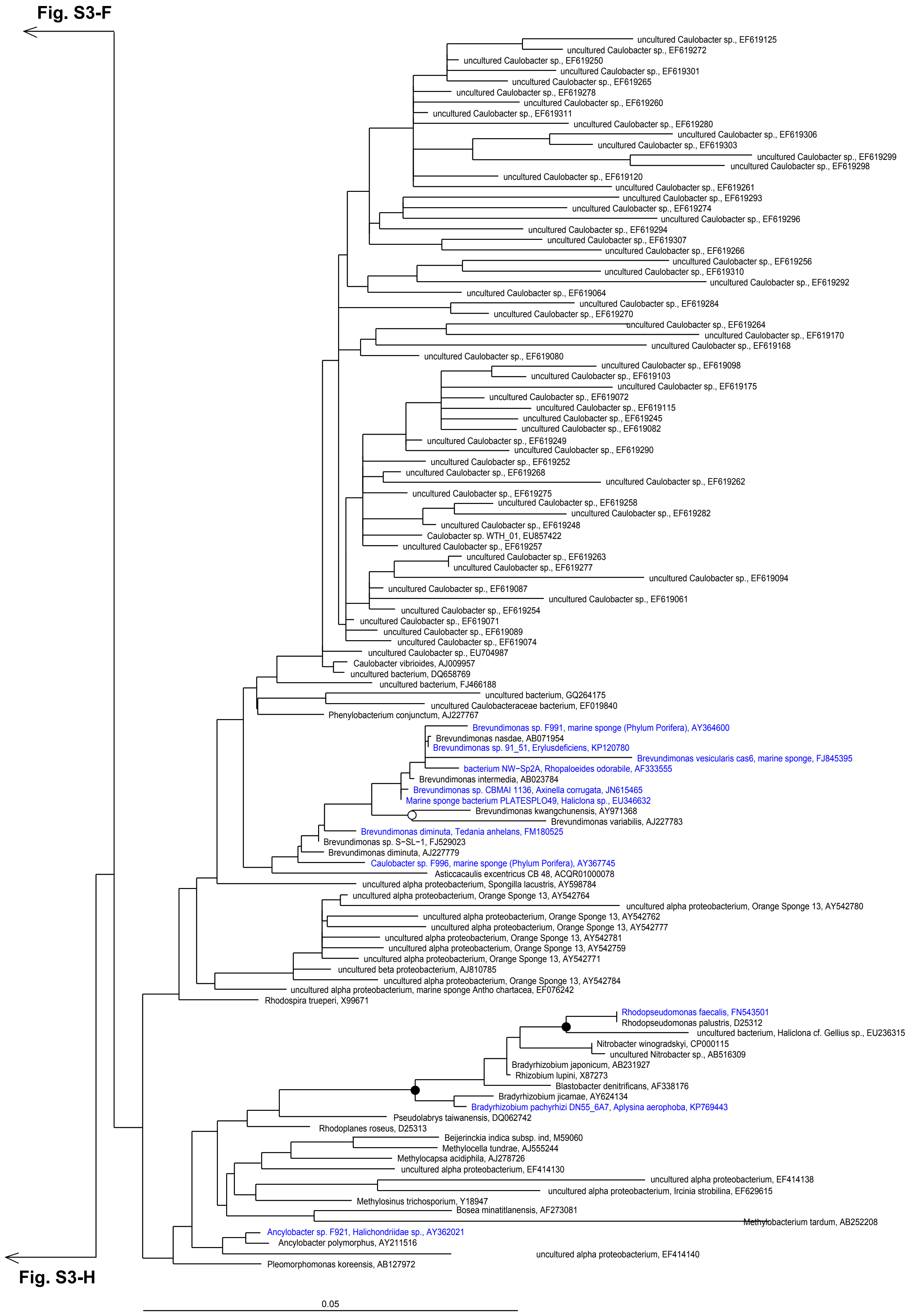

Figure S3-G. 16S rRNA gene-based phylogeny of sponge-associated Alphaproteobacteria. Details are as provided for Figure S1

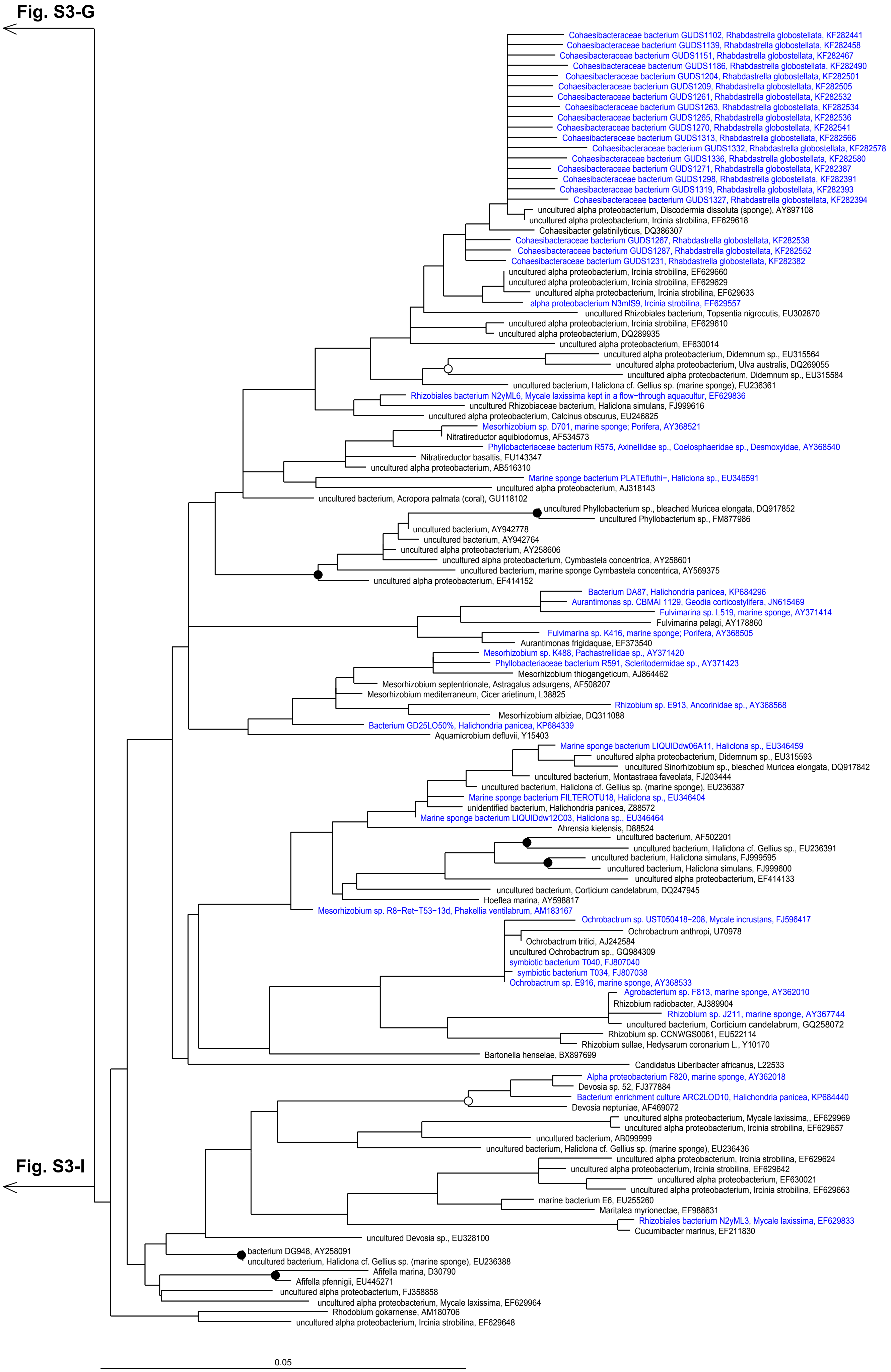

Figure S3-H. 16S rRNA gene-based phylogeny of sponge-associated Alphaproteobacteria. Details are as provided for Figure S1

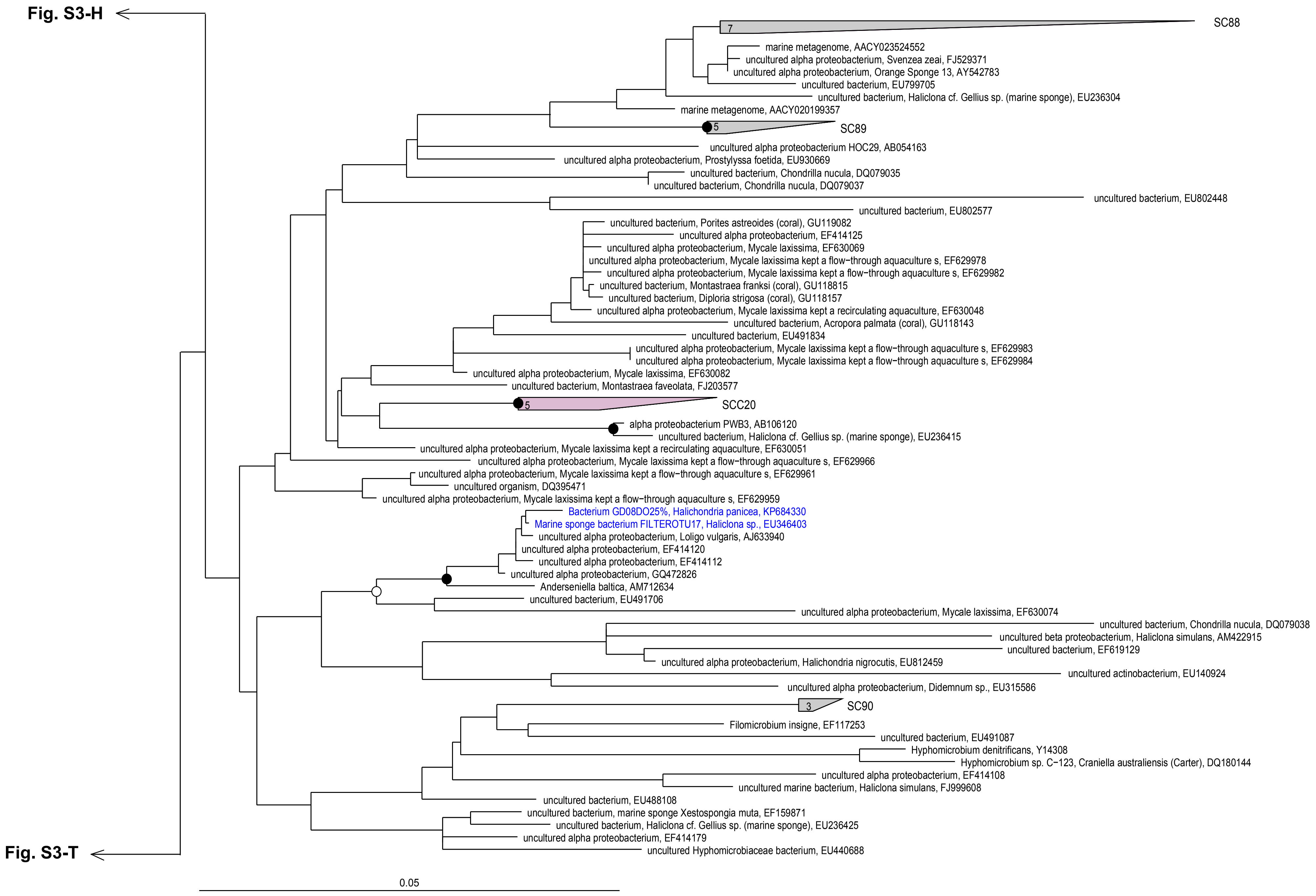

Figure S3-I. 16S rRNA gene-based phylogeny of sponge-associated Alphaproteobacteria. Details are as provided for Figure S1

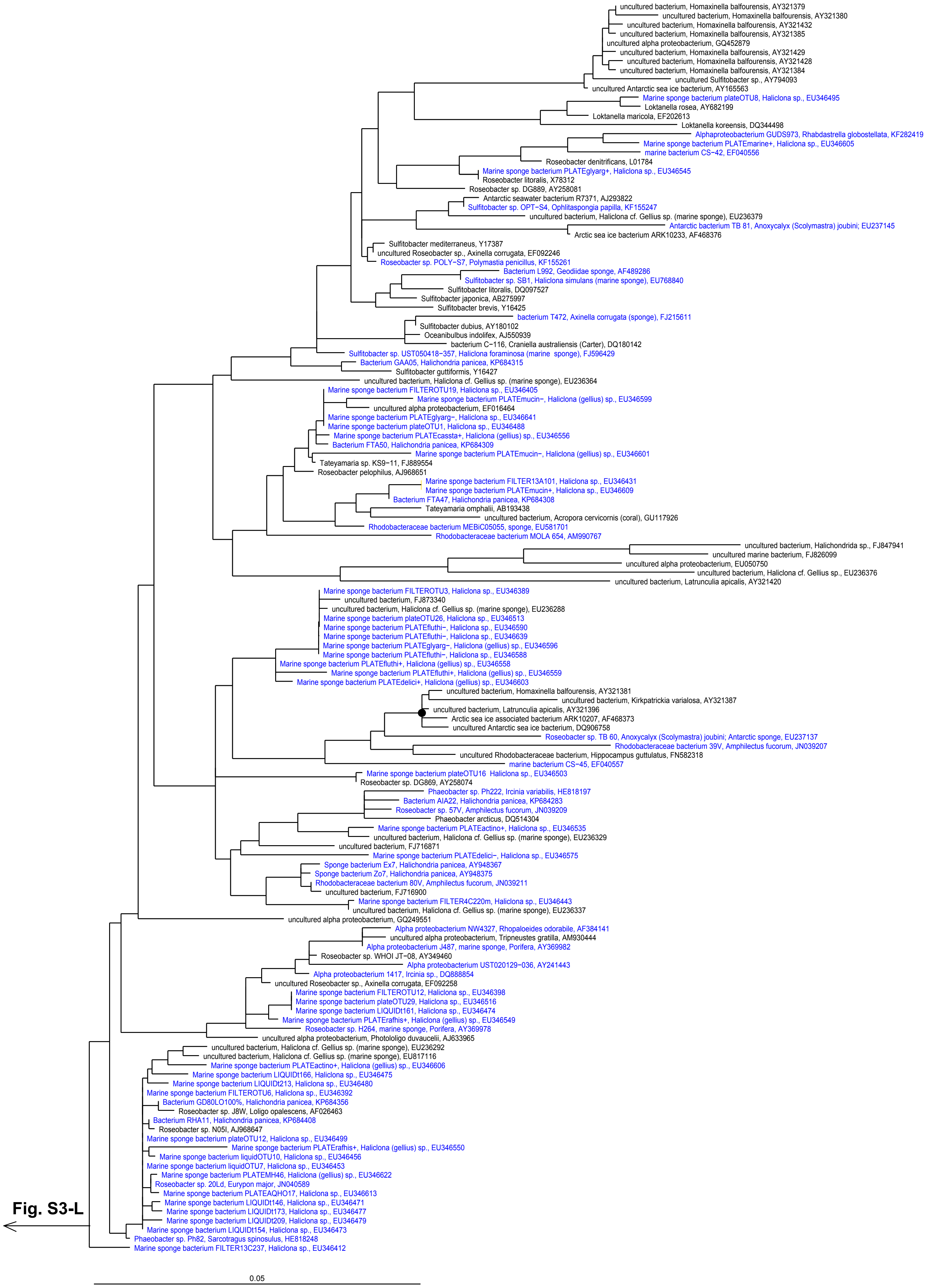

Figure S3-K. 16S rRNA gene-based phylogeny of sponge-associated Alphaproteobacteria. Details are as provided for Figure S1

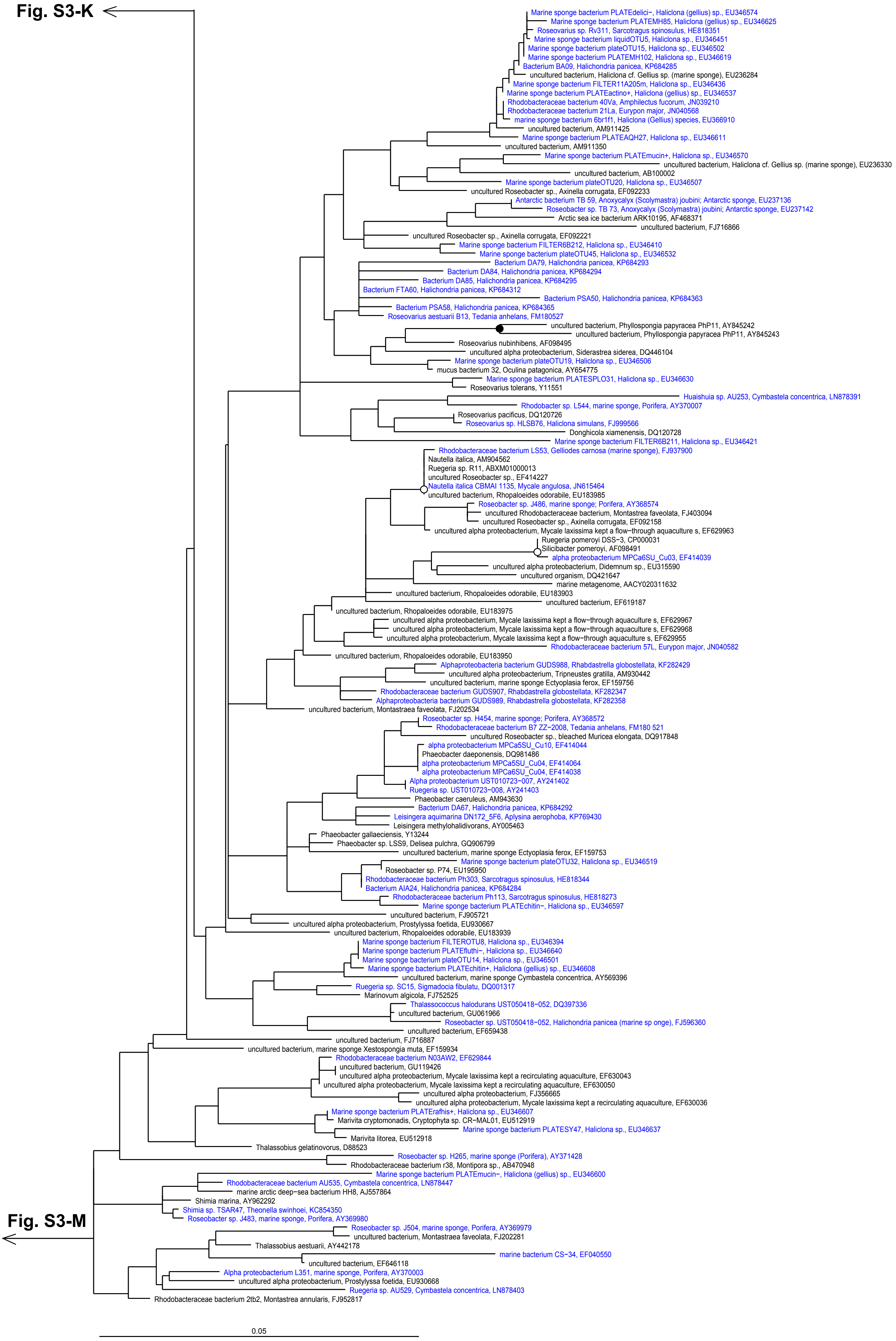

Figure S3-L. 16S rRNA gene-based phylogeny of sponge-associated Alphaproteobacteria. Details are as provided for Figure S1

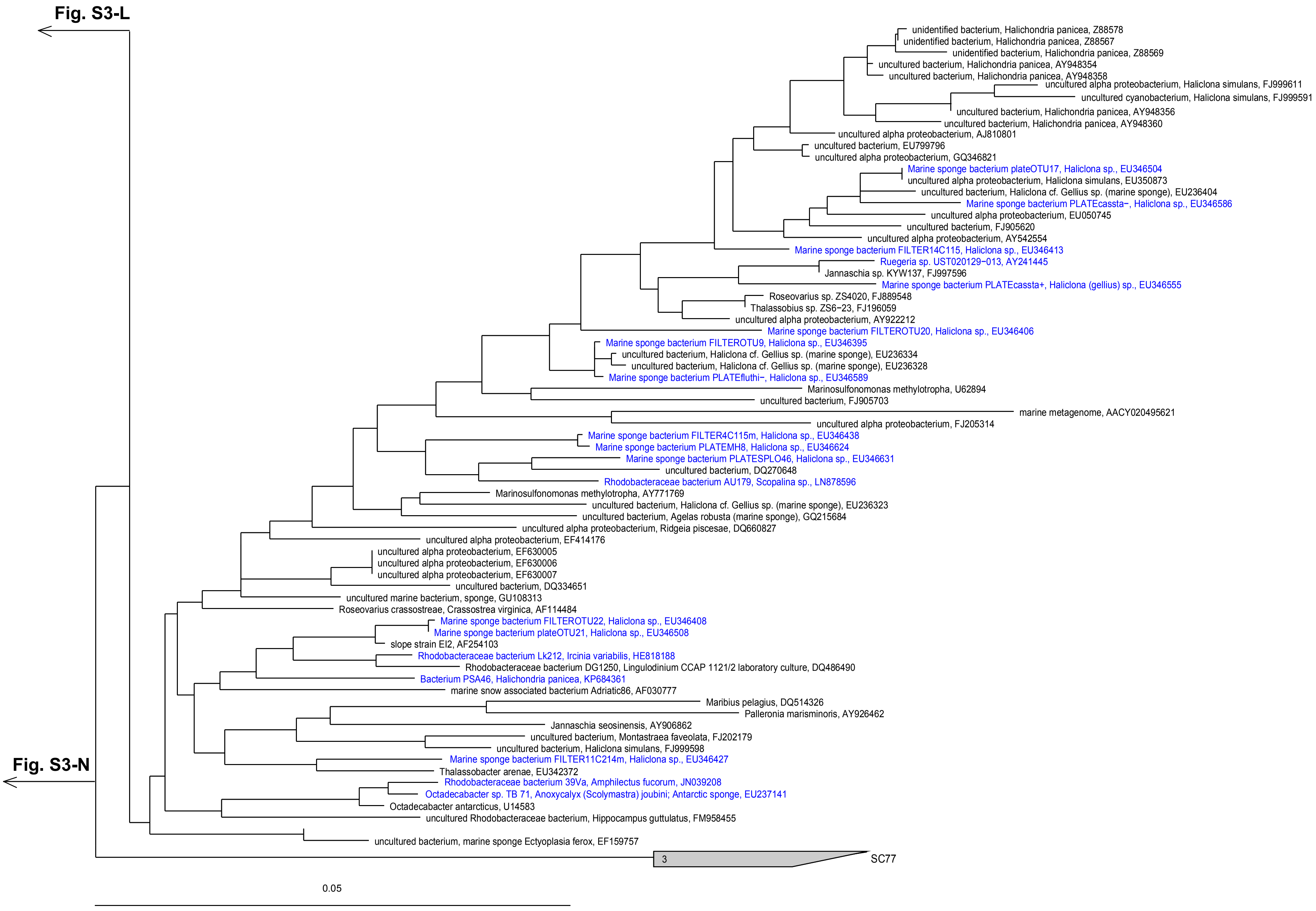

Figure S3-M. 16S rRNA gene-based phylogeny of sponge-associated Alphaproteobacteria. Details are as provided for Figure S1

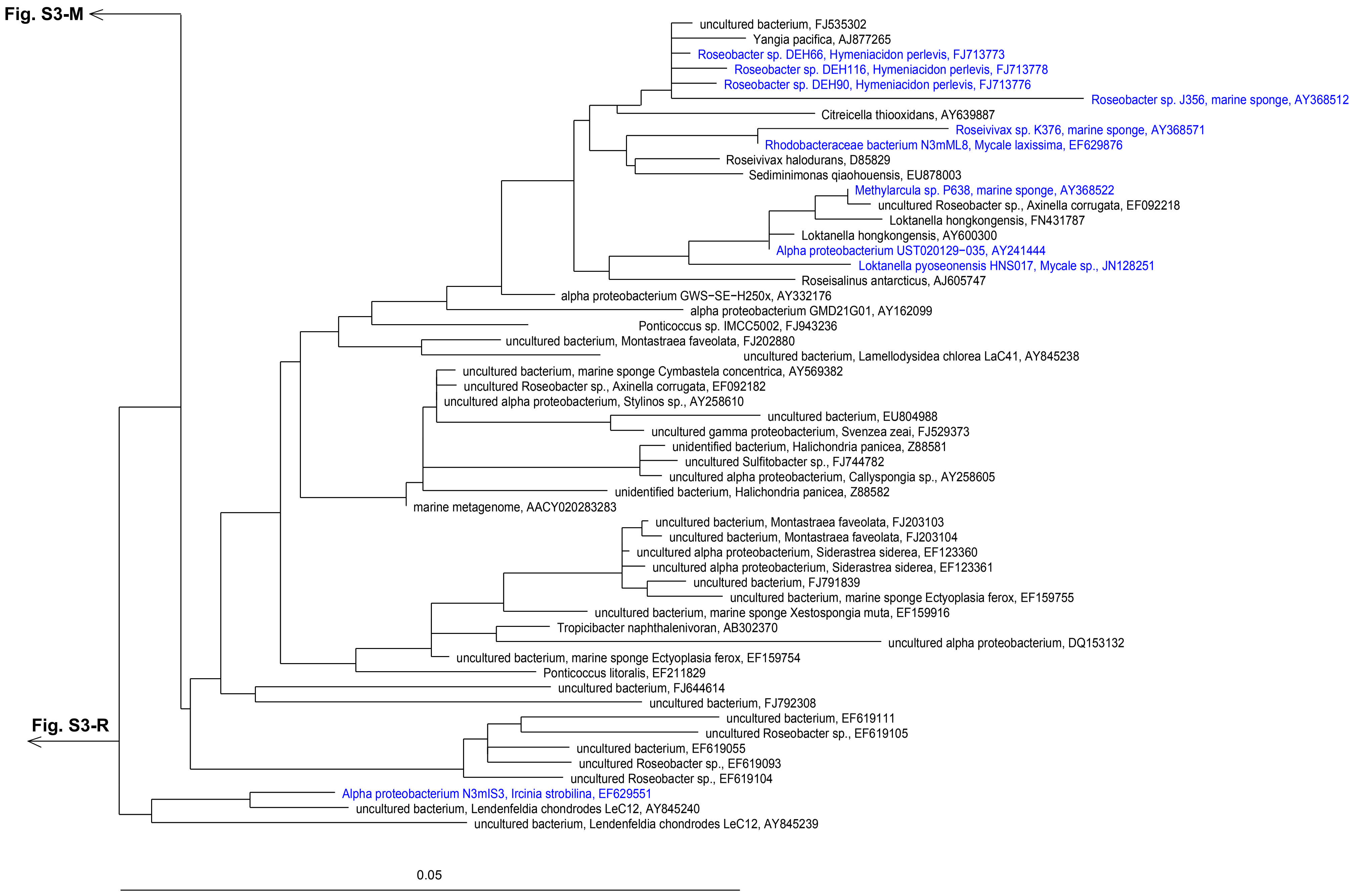

Figure S3-N. 16S rRNA gene-based phylogeny of sponge-associated Alphaproteobacteria. Details are as provided for Figure S1

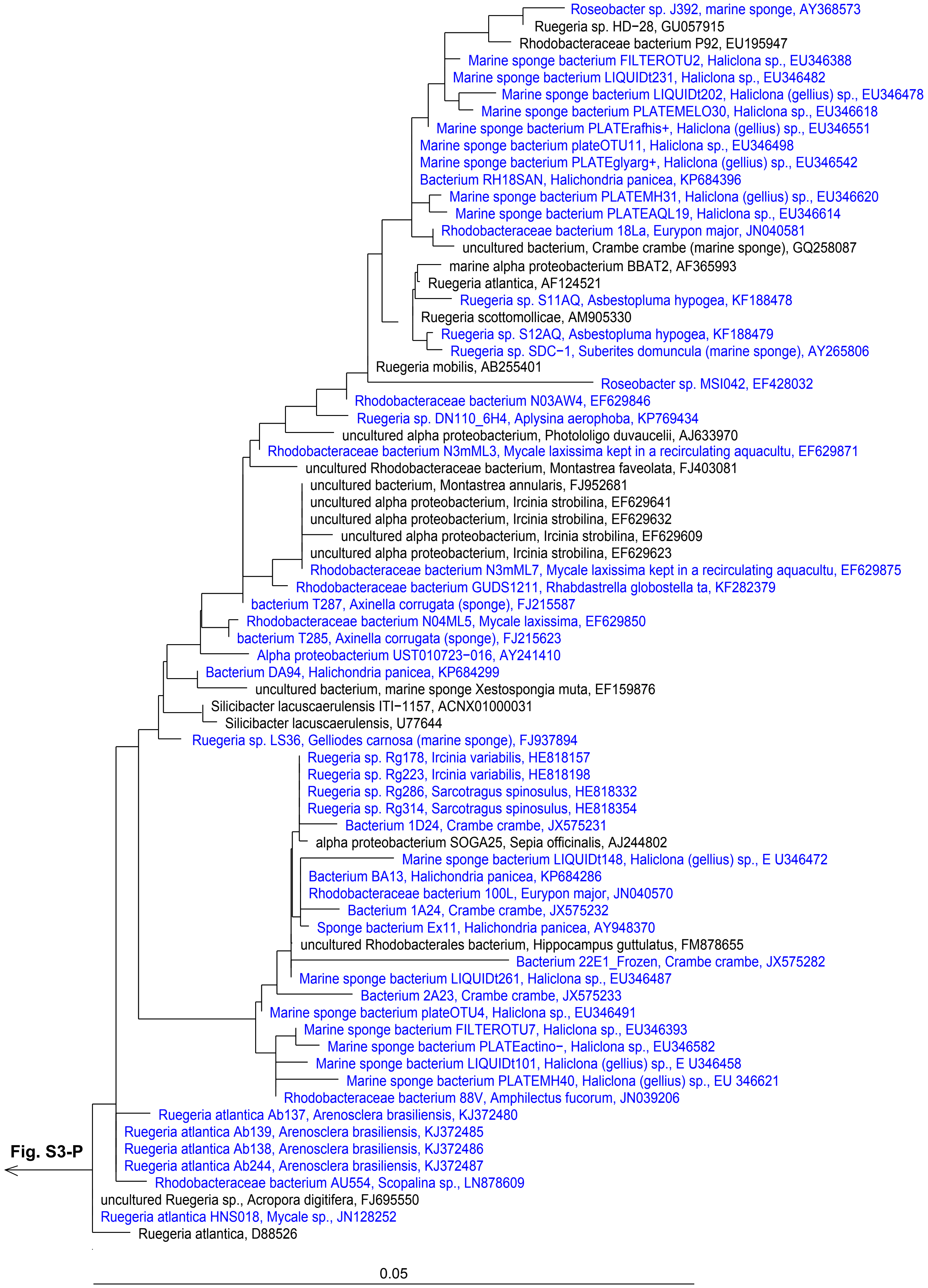

Figure S3-O. 16S rRNA gene-based phylogeny of sponge-associated Alphaproteobacteria. Details are as provided for Figure S1

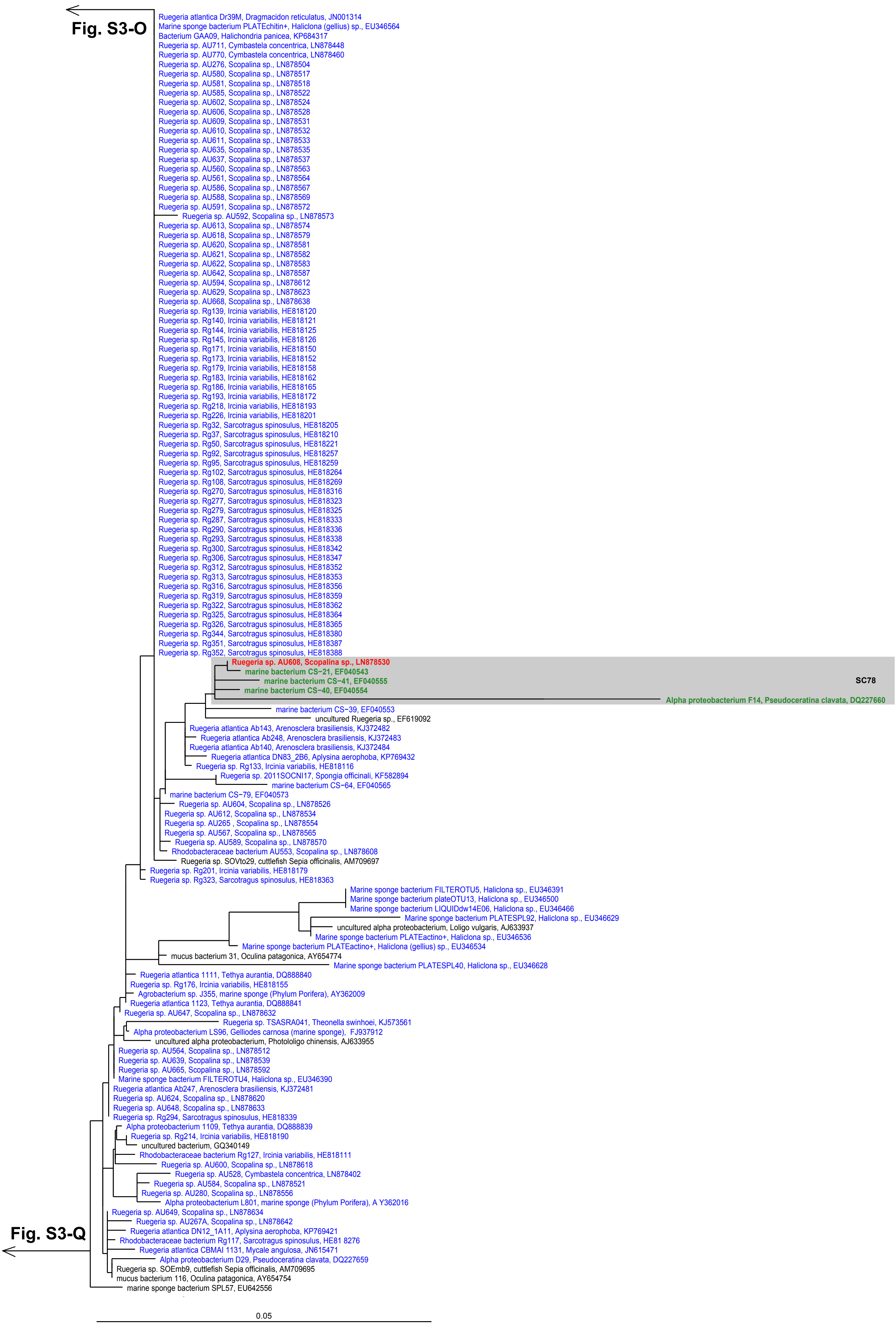

Figure S3-P. 16S rRNA gene-based phylogeny of sponge-associated Alphaproteobacteria. Details are as provided for Figure S1

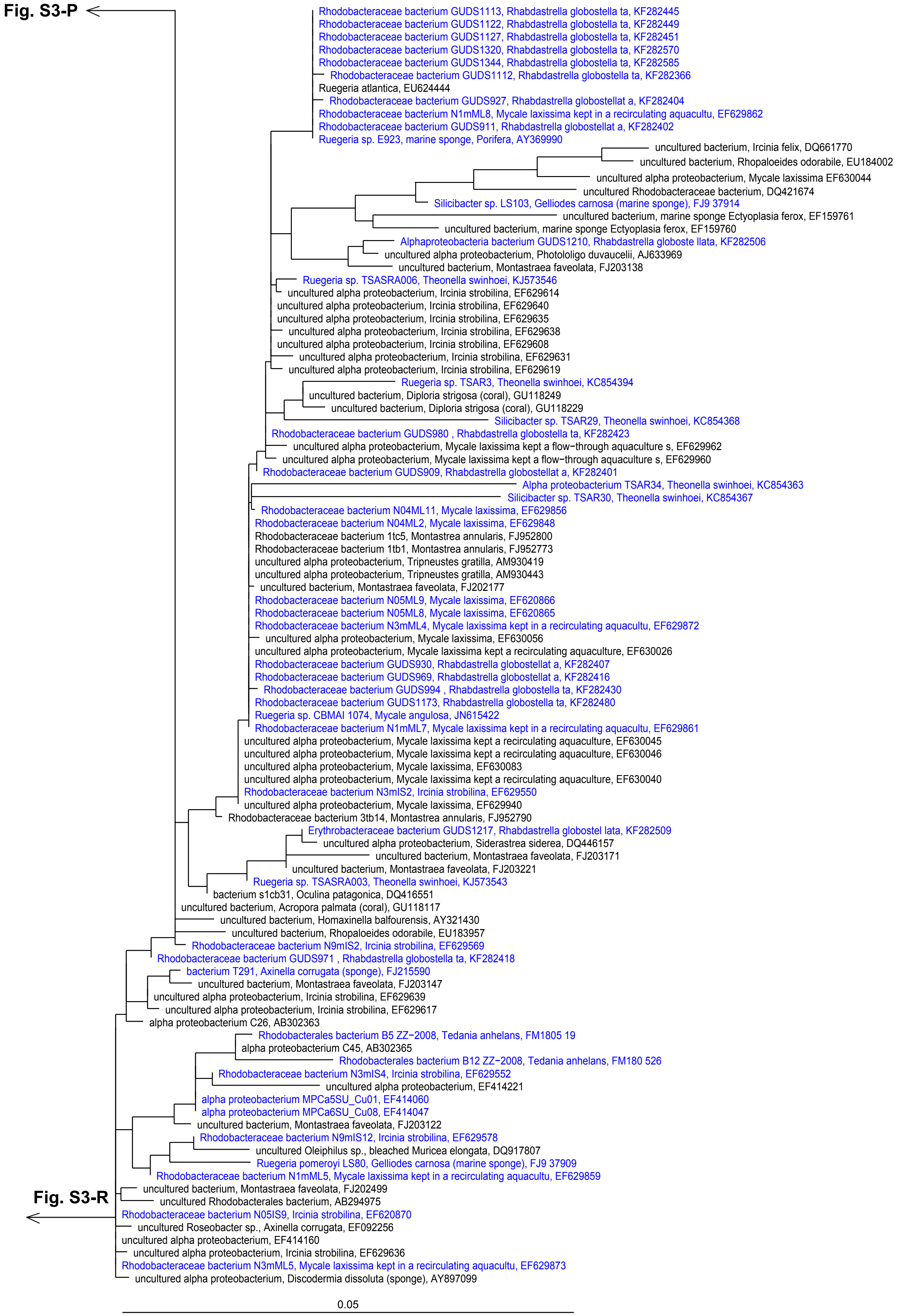

Figure S3-Q. 16S rRNA gene-based phylogeny of sponge-associated Alphaproteobacteria. Details are as provided for Figure S1

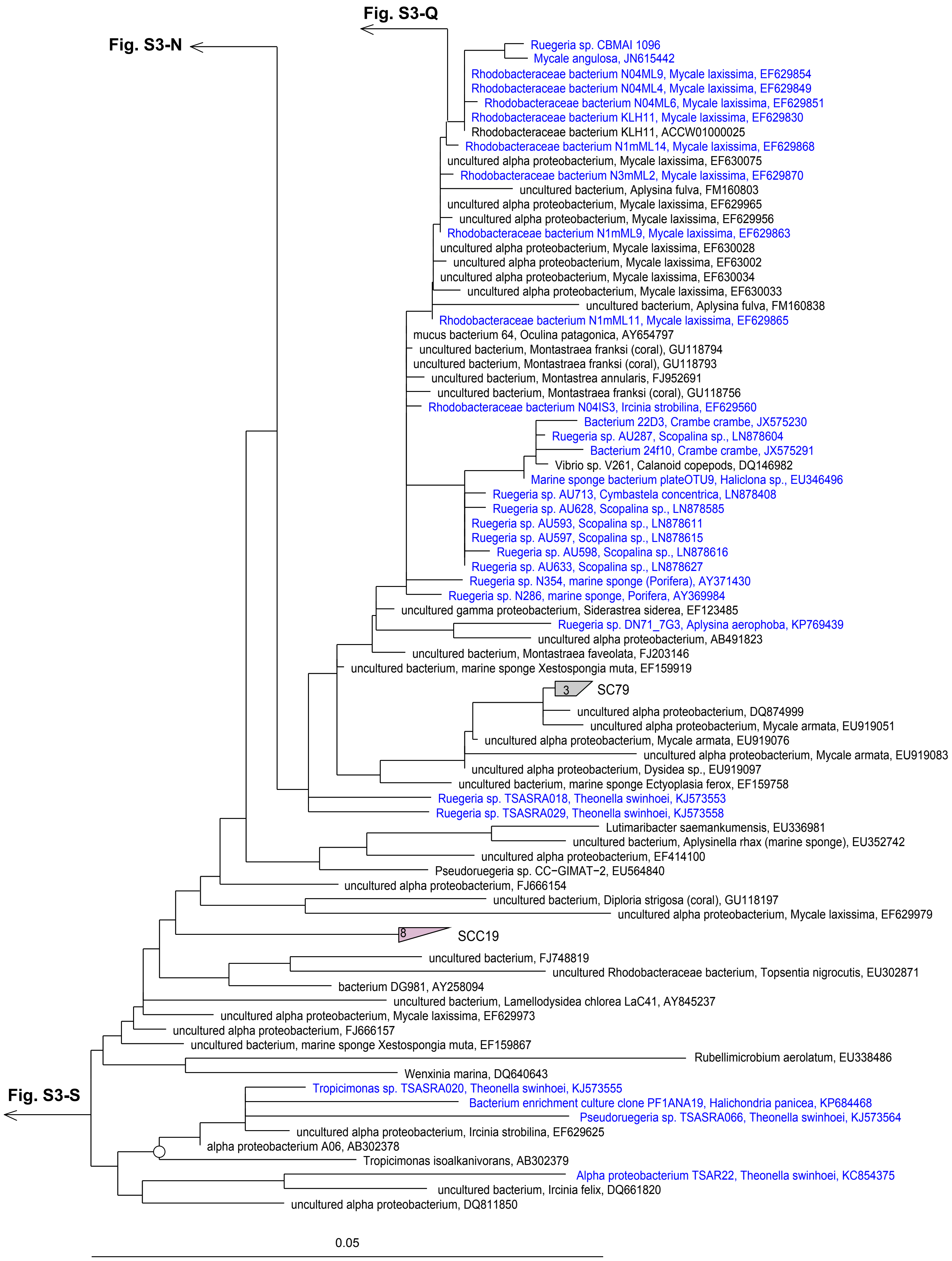

Figure S3-R. 16S rRNA gene-based phylogeny of sponge-associated Alphaproteobacteria. Details are as provided for Figure S1

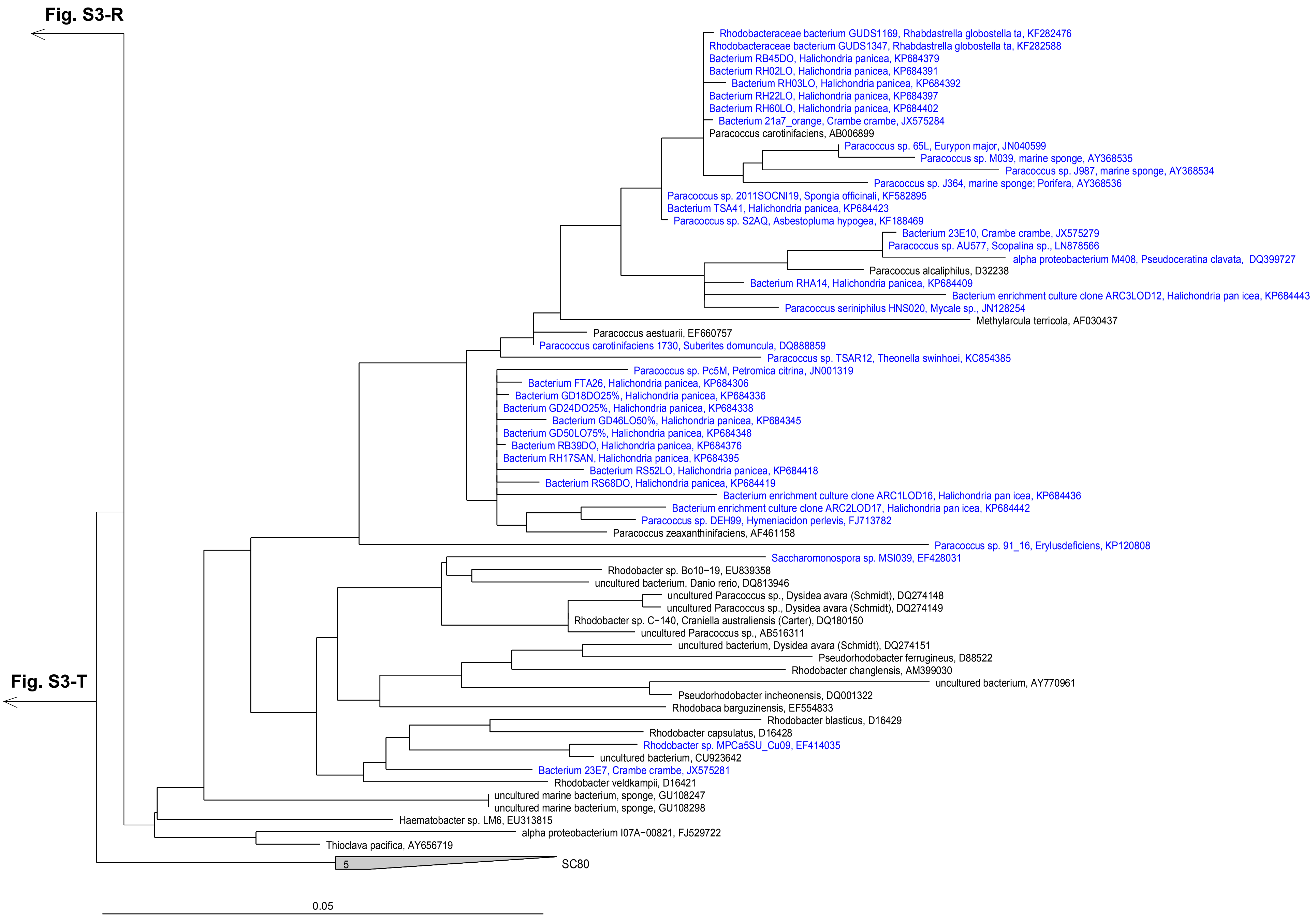

Figure S3-S. 16S rRNA gene-based phylogeny of sponge-associated Alphaproteobacteria. Details are as provided for Figure S1

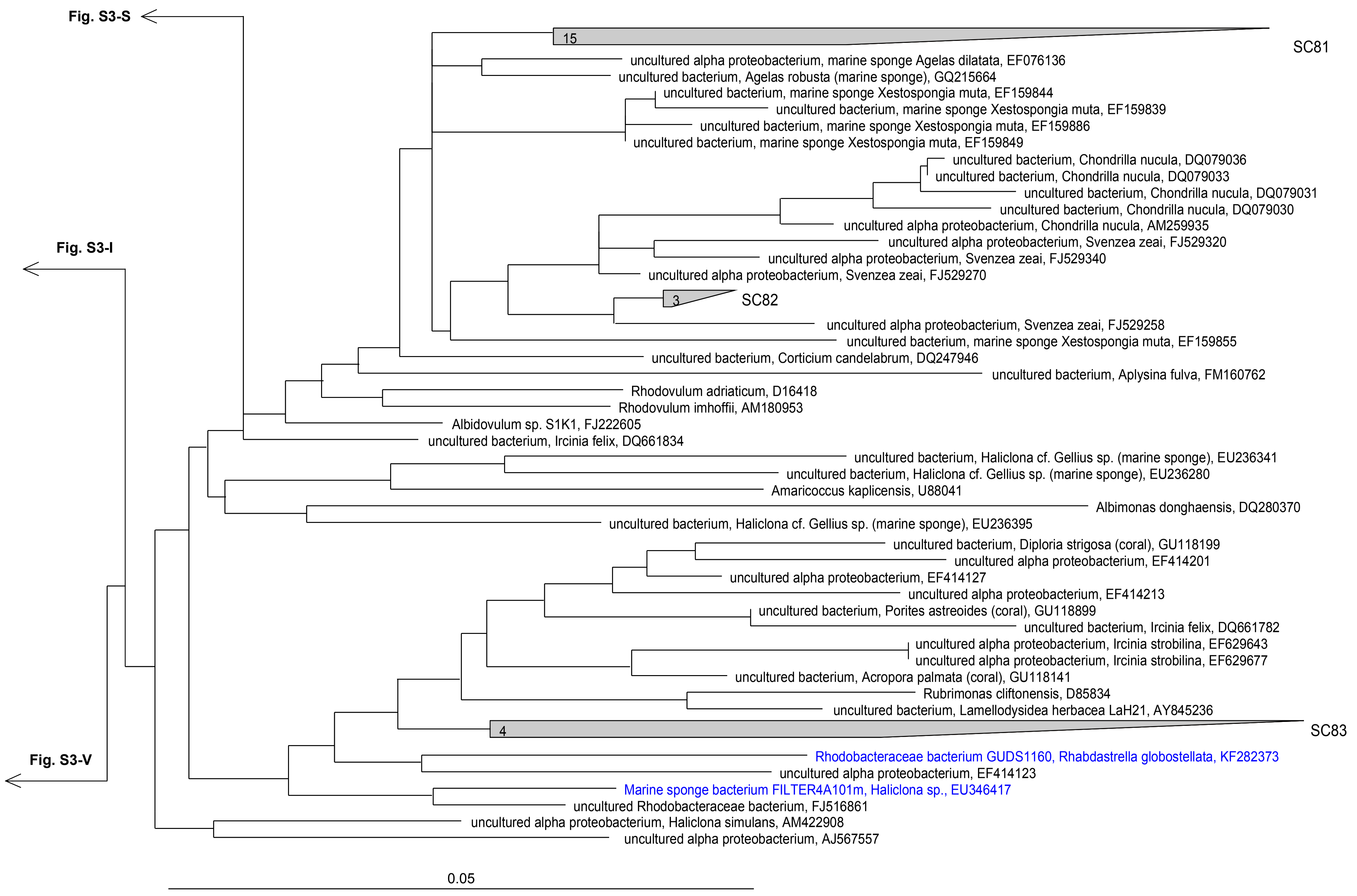

Figure S3-T. 16S rRNA gene-based phylogeny of sponge-associated Alphaproteobacteria. Details are as provided for Figure S1

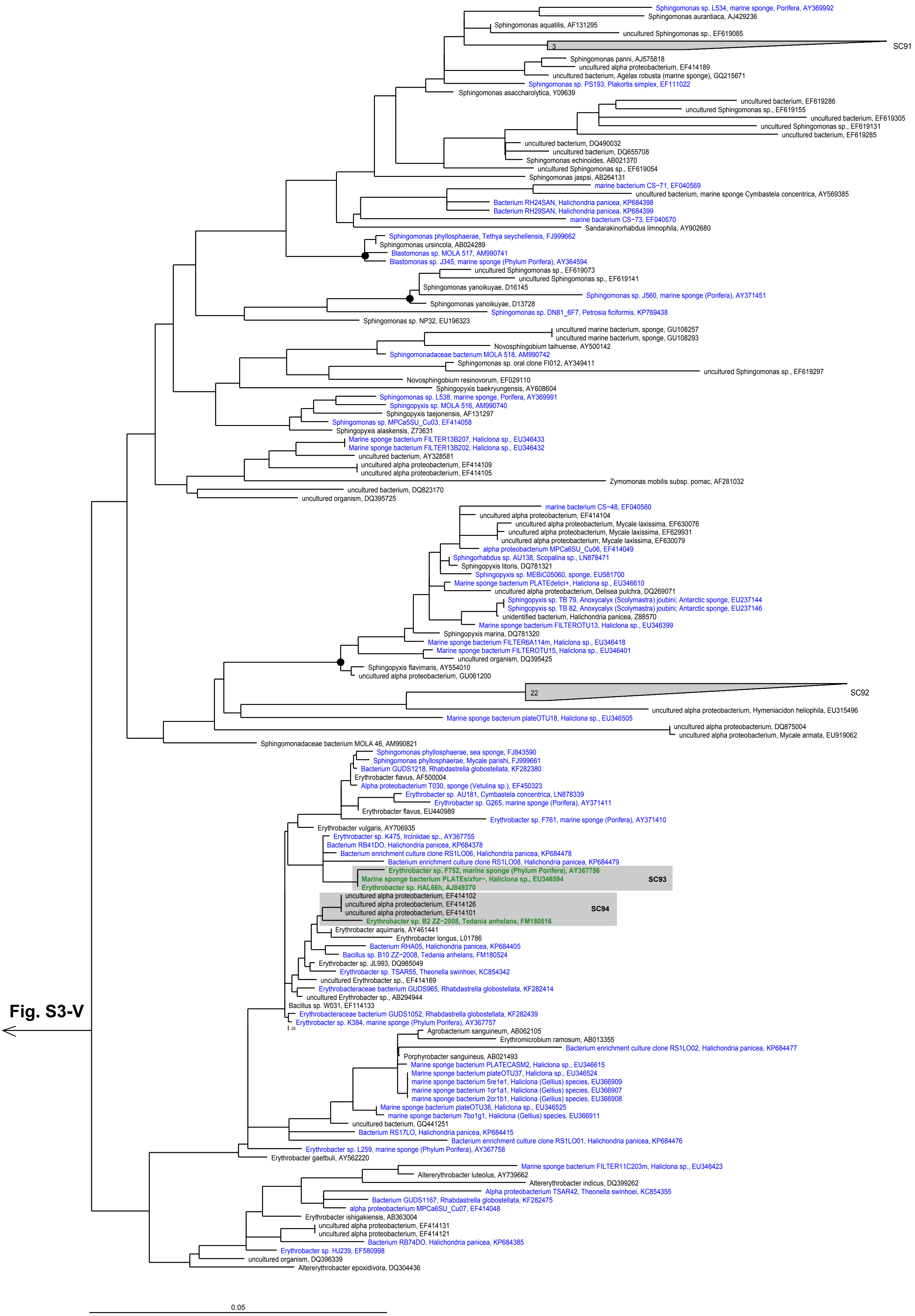

Figure S3-U. 16S rRNA gene-based phylogeny of sponge-associated Alphaproteobacteria. Details are as provided for Figure S1

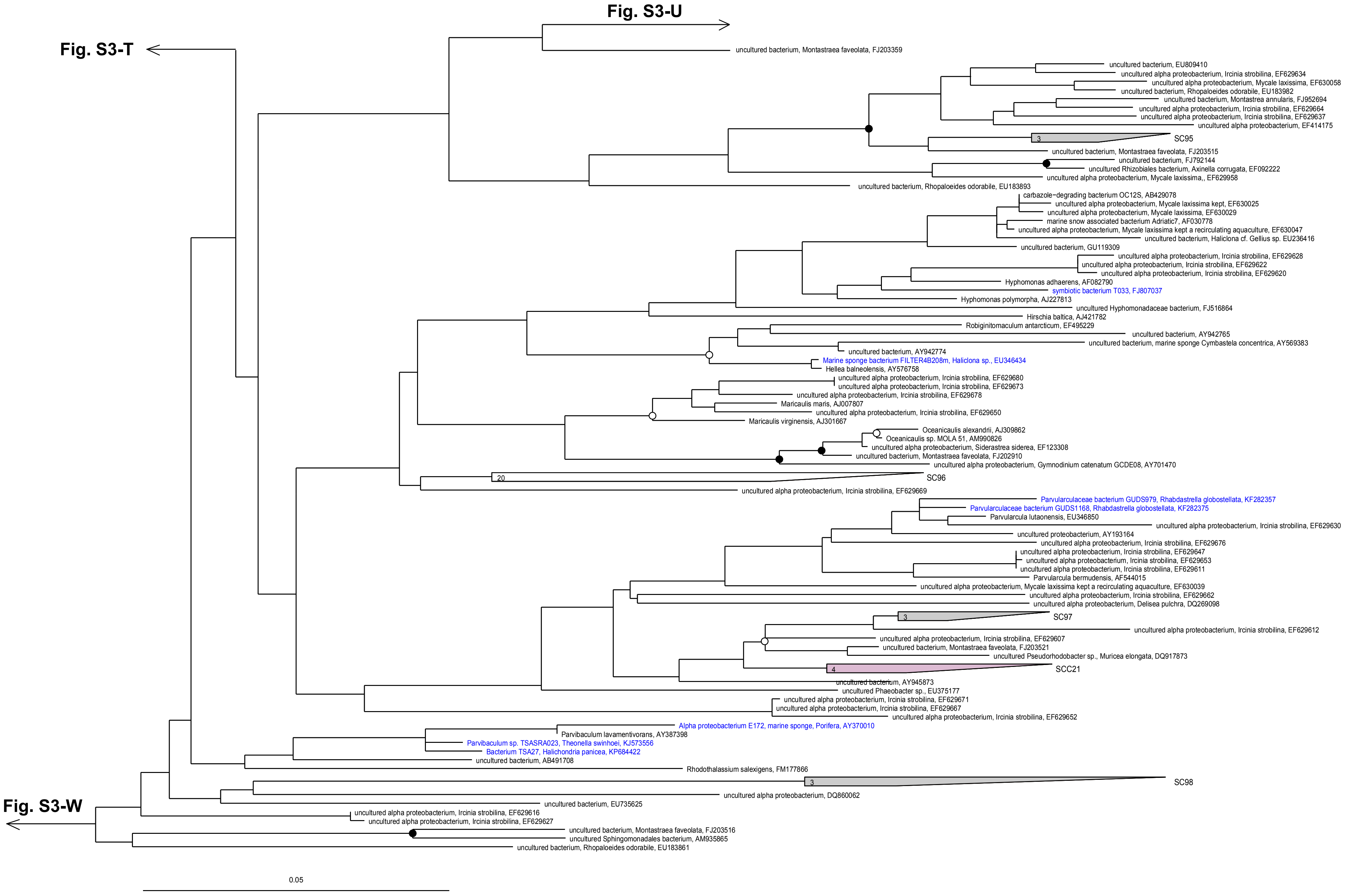

Figure S3-V. 16S rRNA gene-based phylogeny of sponge-associated Alphaproteobacteria. Details are as provided for Figure S1

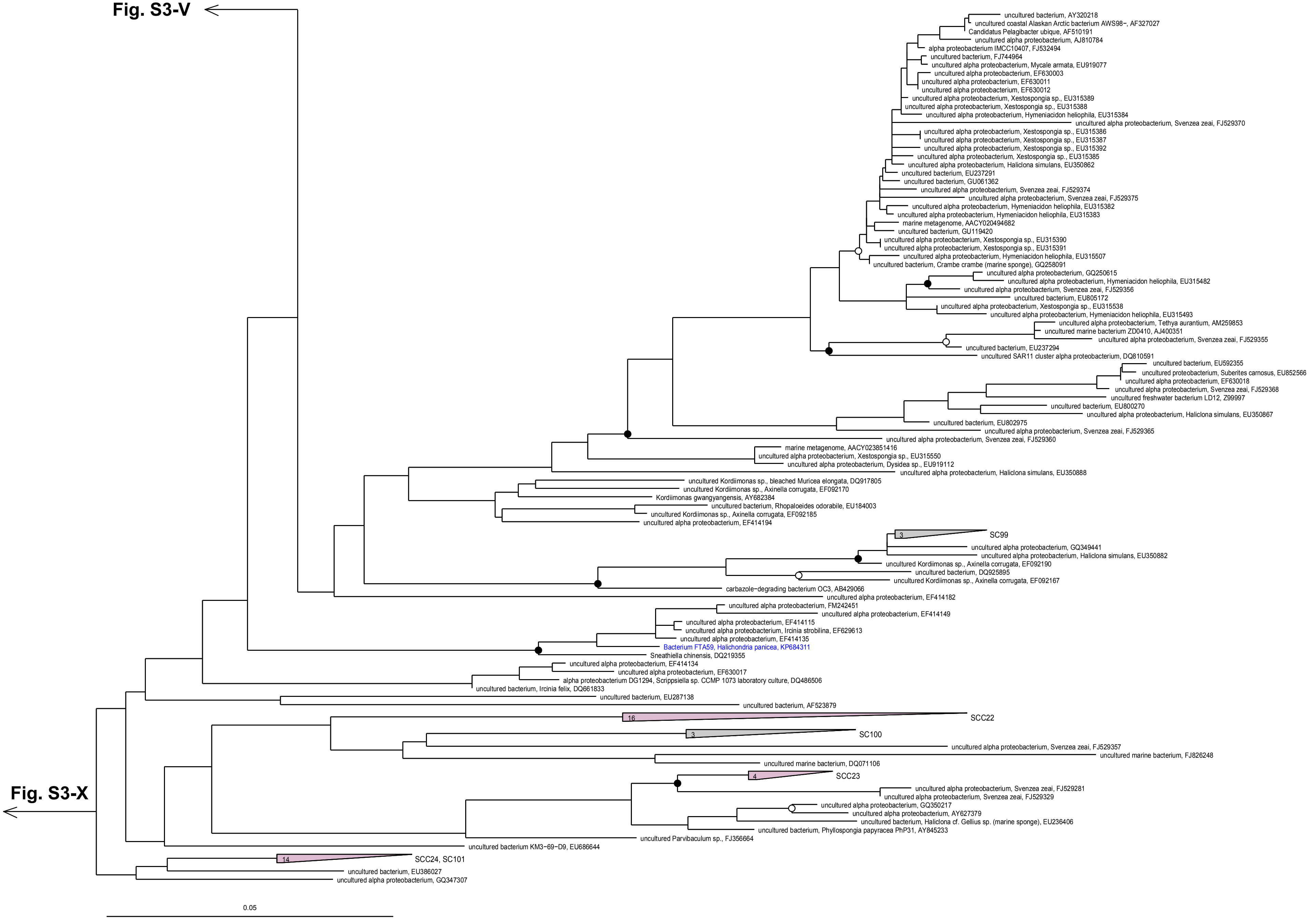

Figure S3-W. 16S rRNA gene-based phylogeny of sponge-associated Alphaproteobacteria. Details are as provided for Figure S1

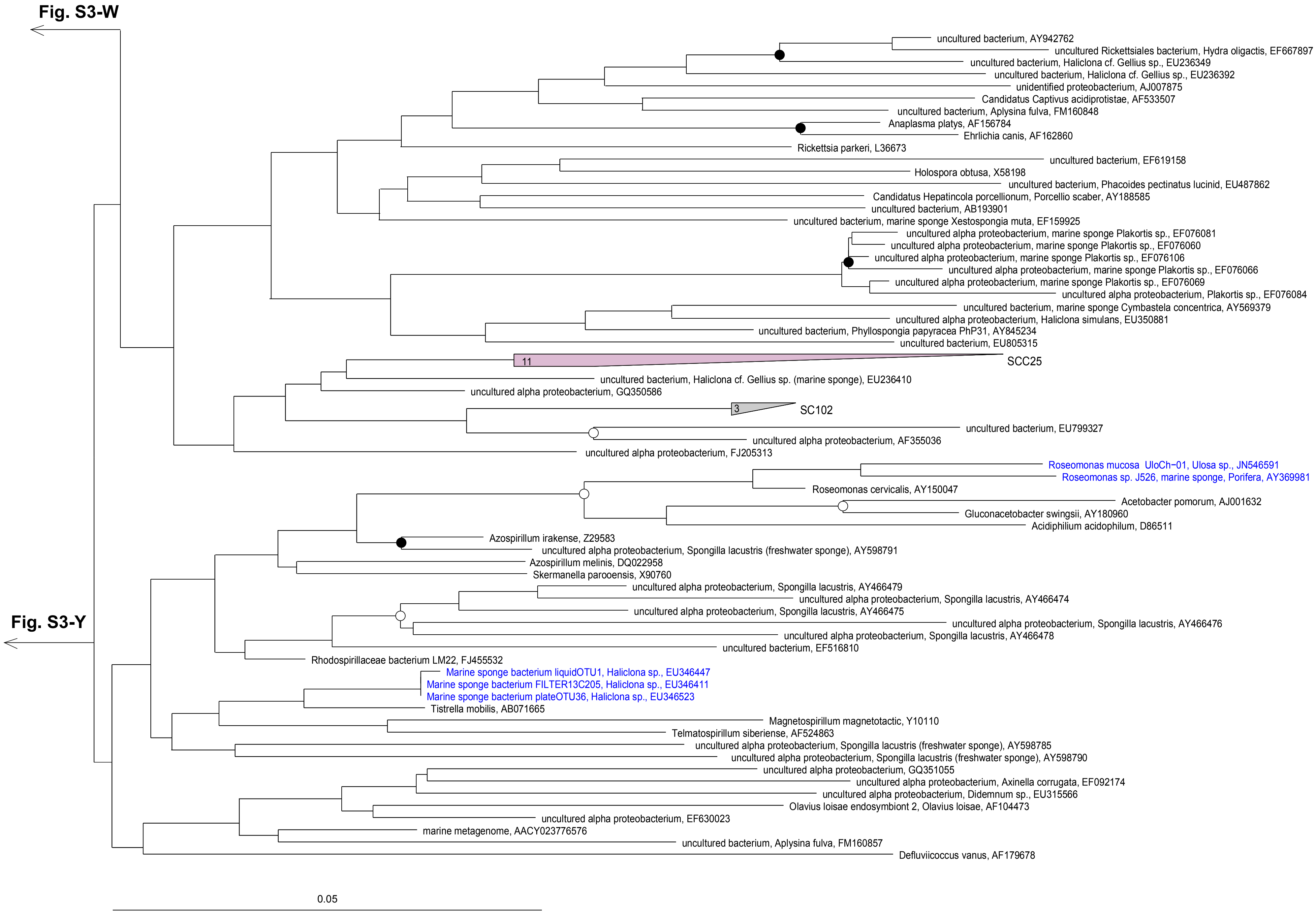

Figure S3-X. 16S rRNA gene-based phylogeny of sponge-associated Alphaproteobacteria. Details are as provided for Figure S1

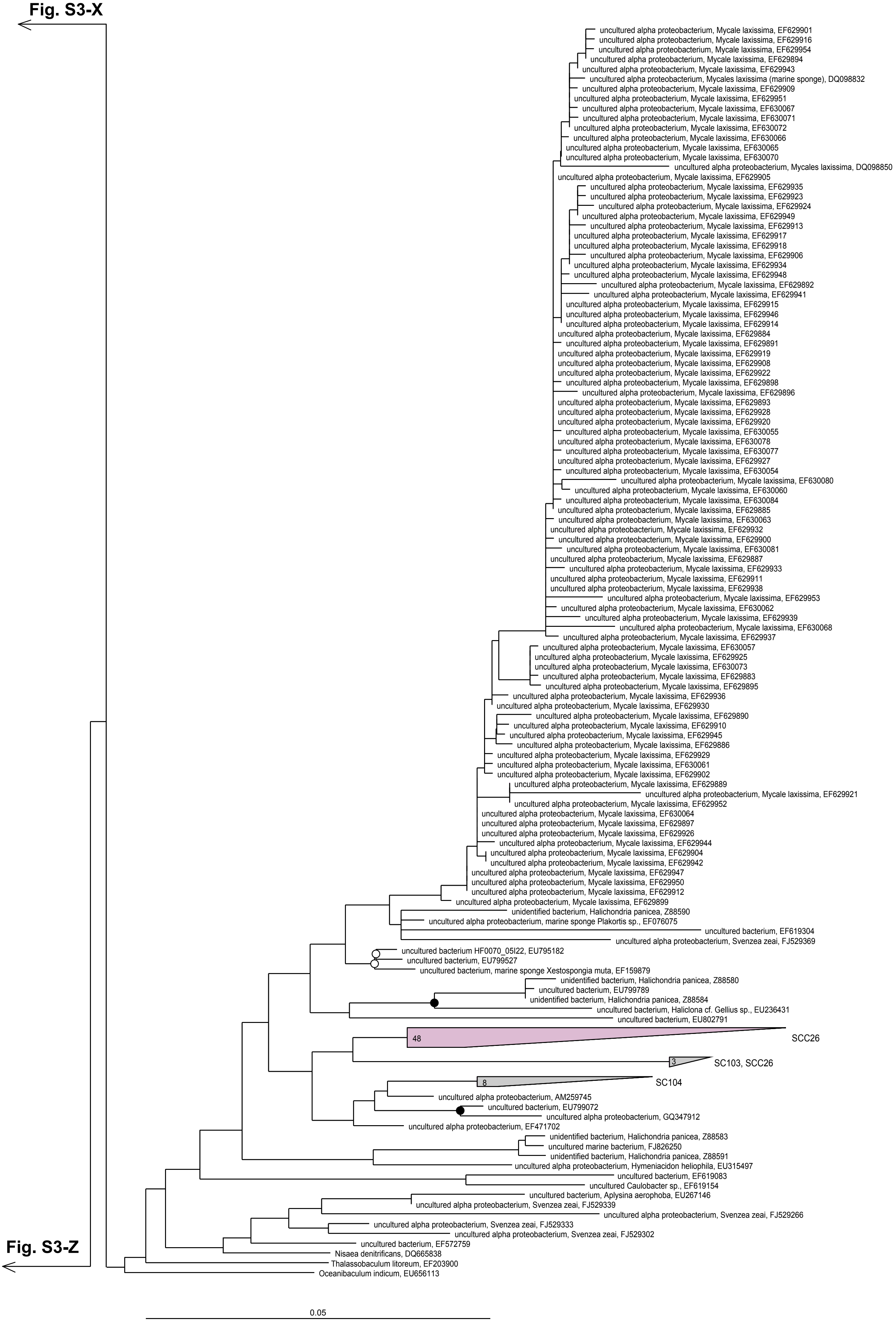

Figure S3-Y. 16S rRNA gene-based phylogeny of sponge-associated Alphaproteobacteria. Details are as provided for Figure S1

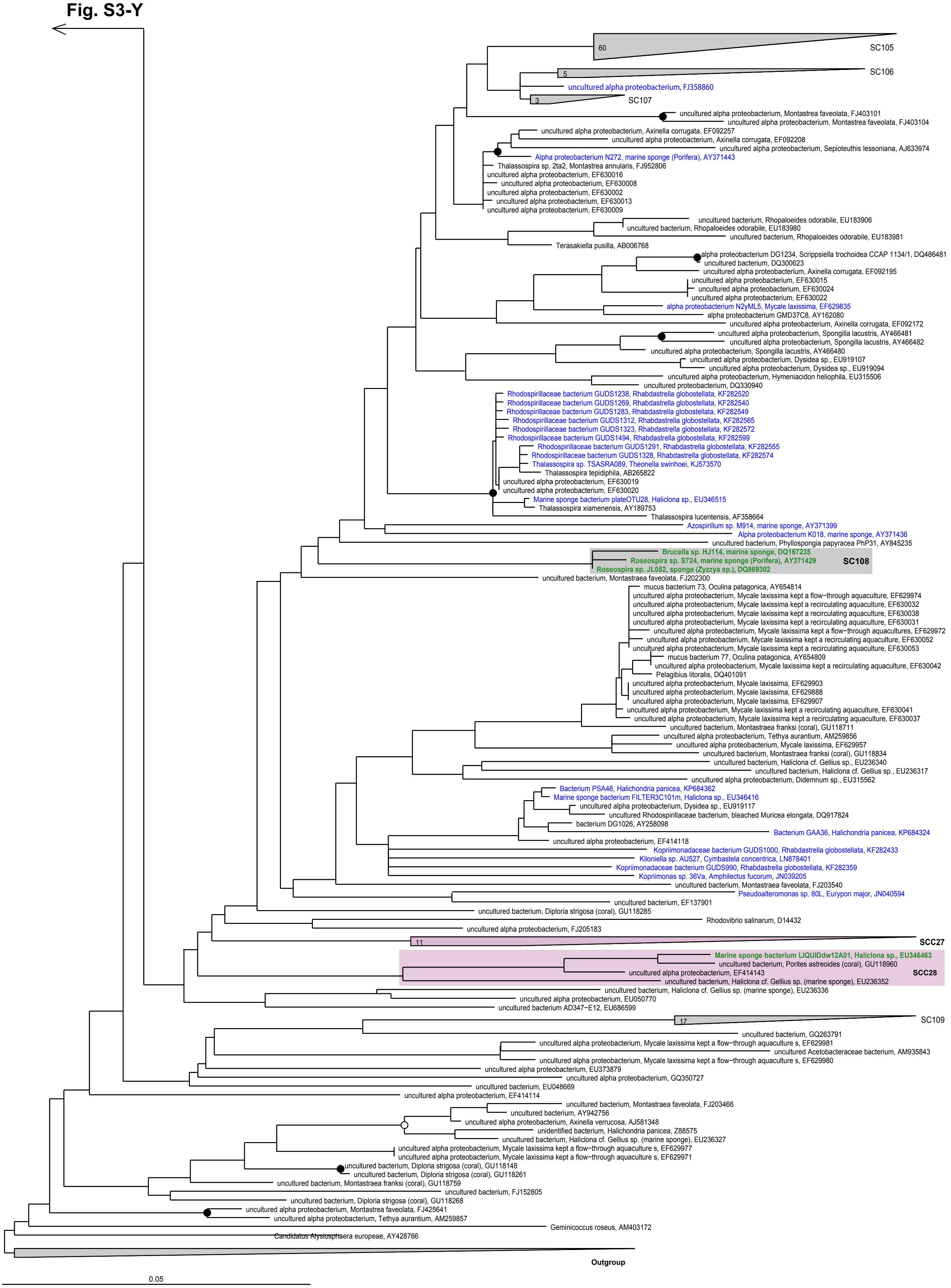

Figure S3-Z. 16S rRNA gene-based phylogeny of sponge-associated Alphaproteobacteria. Details are as provided for Figure S1
