# Supplementary material for: Bacteria Cultivated From Sponges and Bacteria Not Yet Cultivated From Sponges—A Review
Source: Front Microbiol. 2021 Nov 10;12:737925. doi: 10.3389/fmicb.2021.737925 (PMC8634882; doi:10.3389/fmicb.2021.737925)
Supplement: Supplementary file 6 [file Image_6.pdf]

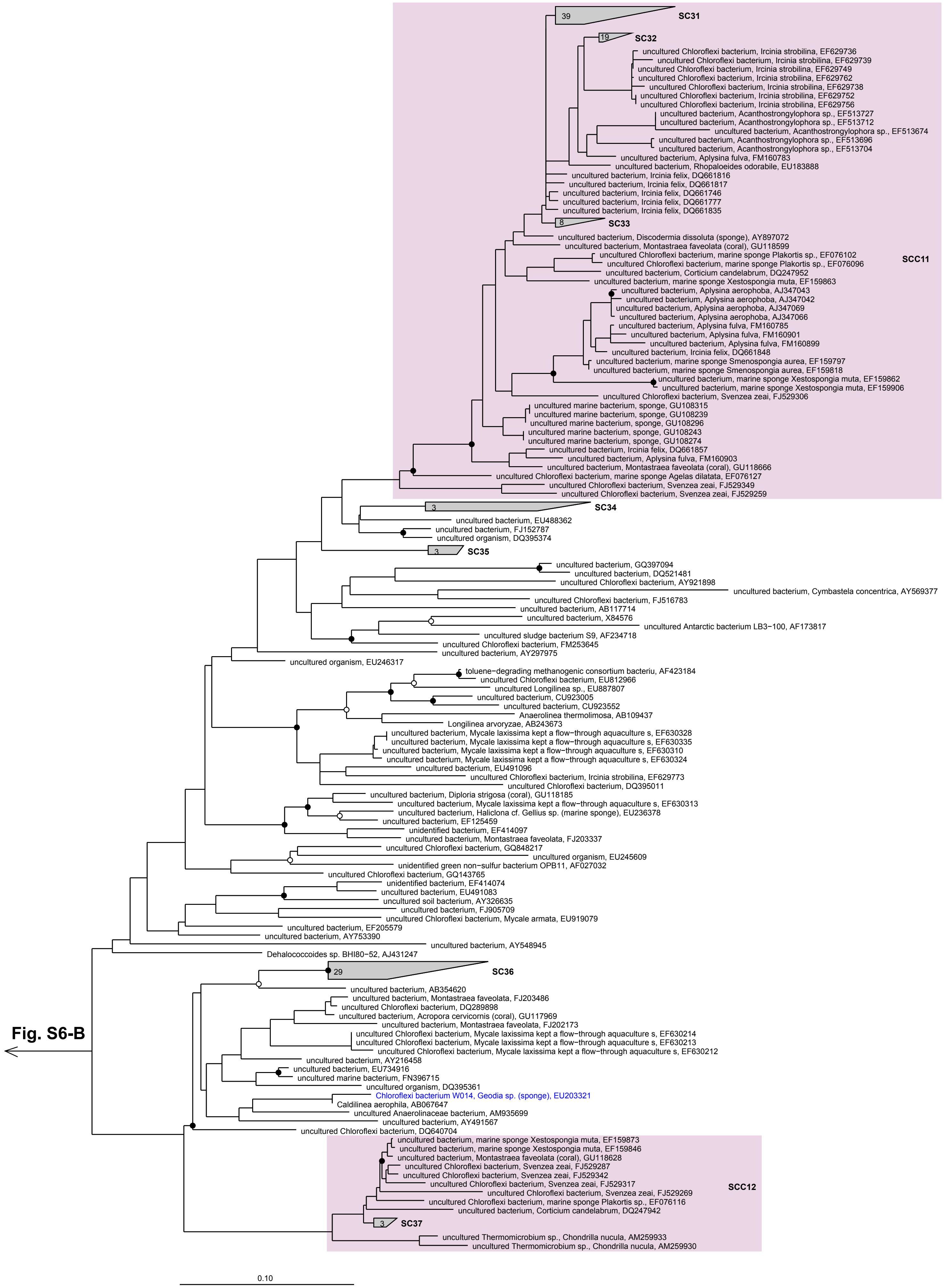

Figure S6-A. 16S rRNA gene-based phylogeny of sponge-associated Chloroflexi. Details are as provided for Figure S1

Fig. S6-A

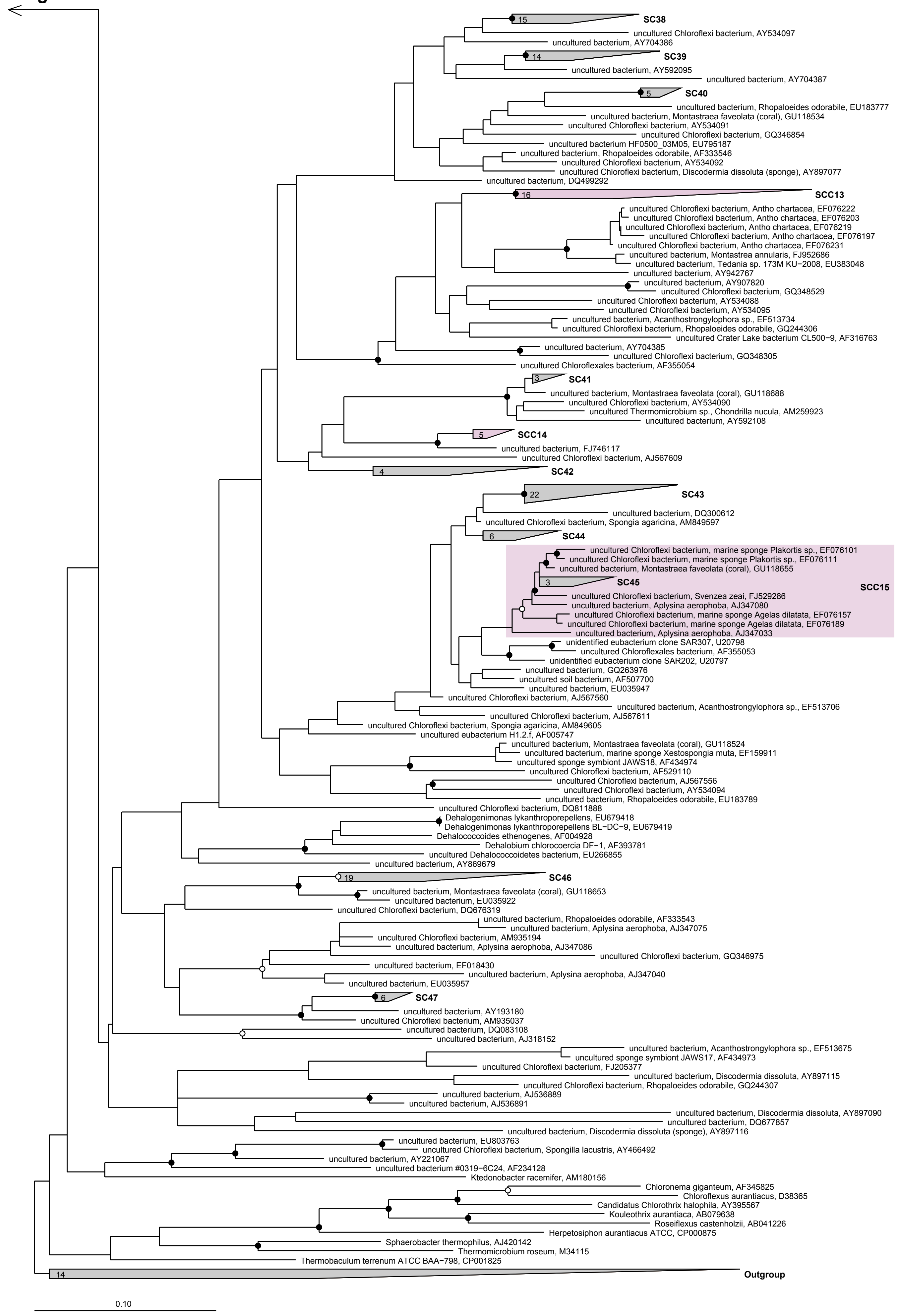

Figure S6-B. 16S rRNA gene-based phylogeny of sponge-associated Chloroflexi. Details are as provided for Figure S1
