# Supplementary material for: Bacteria Cultivated From Sponges and Bacteria Not Yet Cultivated From Sponges—A Review
Source: Front Microbiol. 2021 Nov 10;12:737925. doi: 10.3389/fmicb.2021.737925 (PMC8634882; doi:10.3389/fmicb.2021.737925)
Supplement: Supplementary file 7 [file Image_7.pdf]

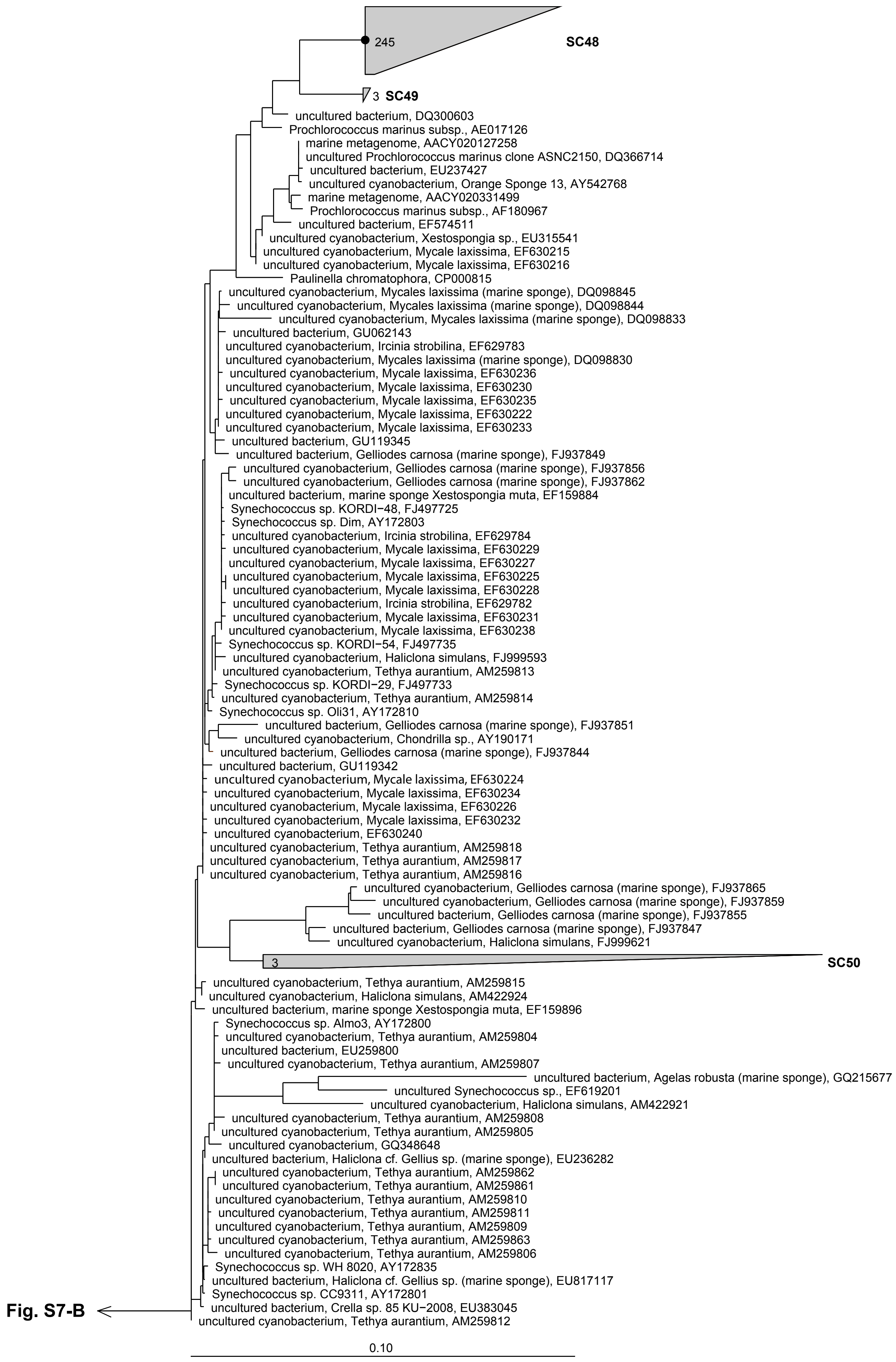

Figure S7-A. 16S rRNA gene-based phylogeny of sponge-associated Cyanobacteria. Details are as provided for Figure S1

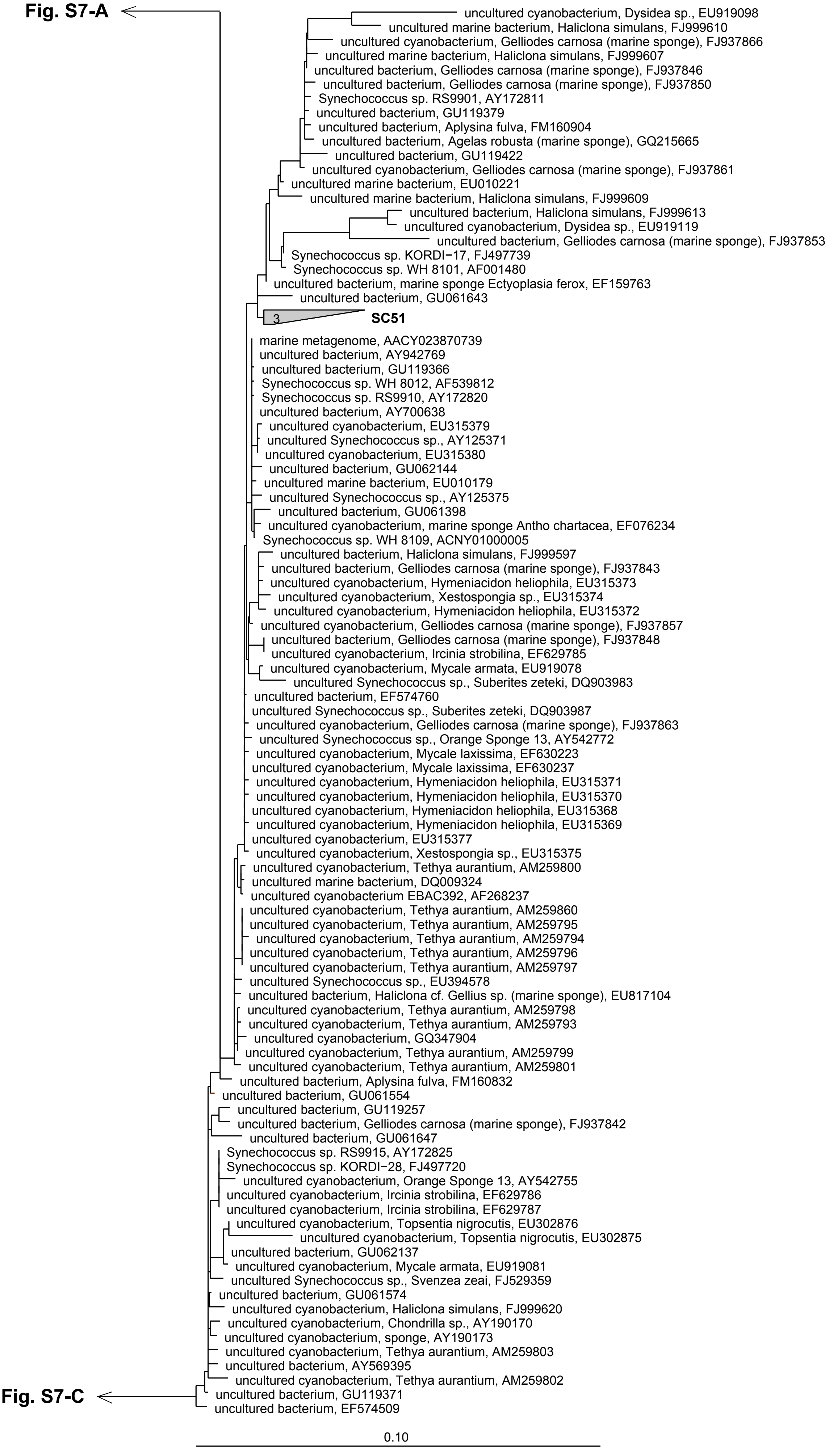

Figure S7-B. 16S rRNA gene-based phylogeny of sponge-associated Cyanobacteria. Details are as provided for Figure S1

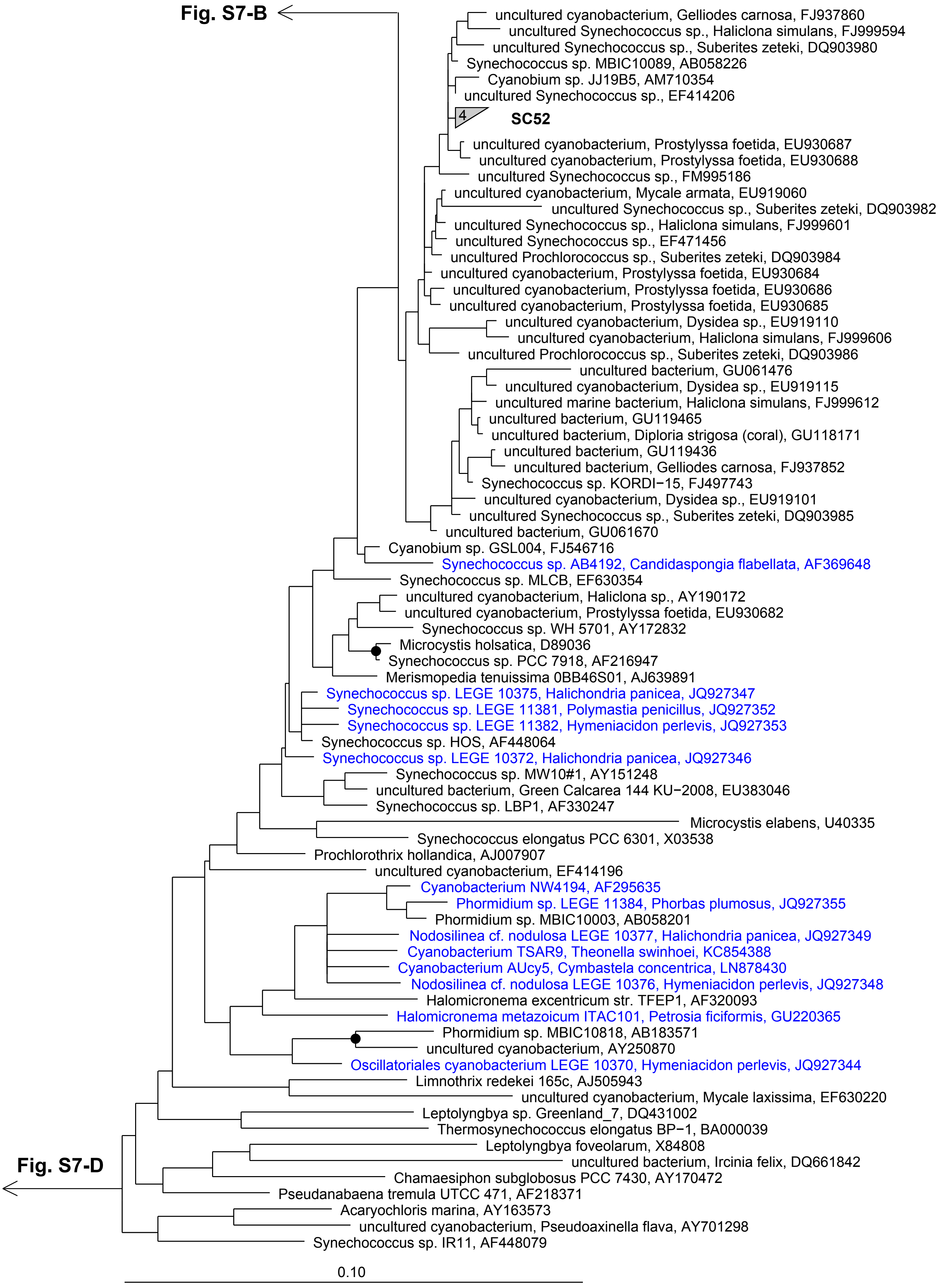

Figure S7-C. 16S rRNA gene-based phylogeny of sponge-associated Cyanobacteria. Details are as provided for Figure S1

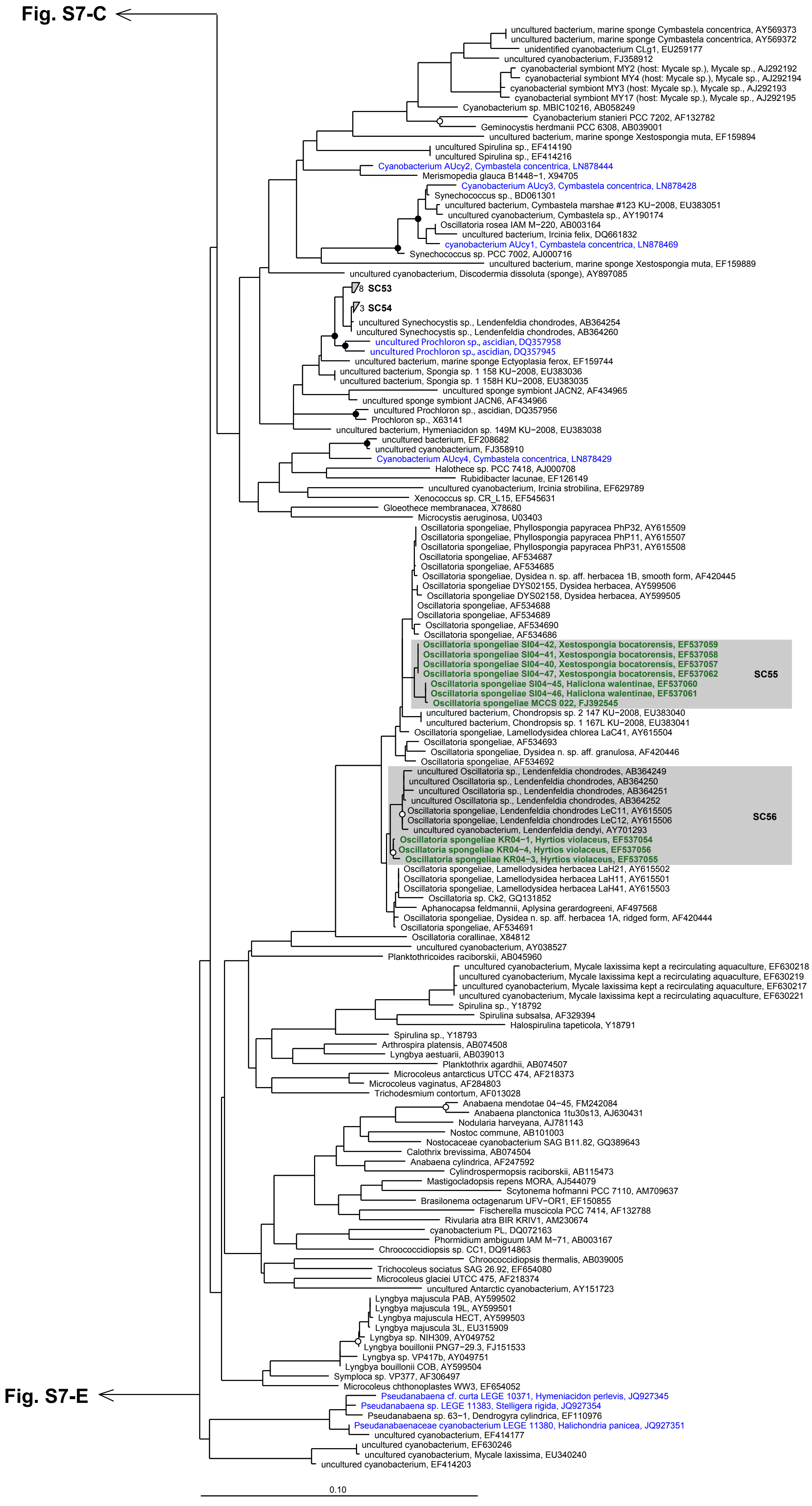

Figure S7-D. 16S rRNA gene-based phylogeny of sponge-associated Cyanobacteria. Details are as provided for Figure S1

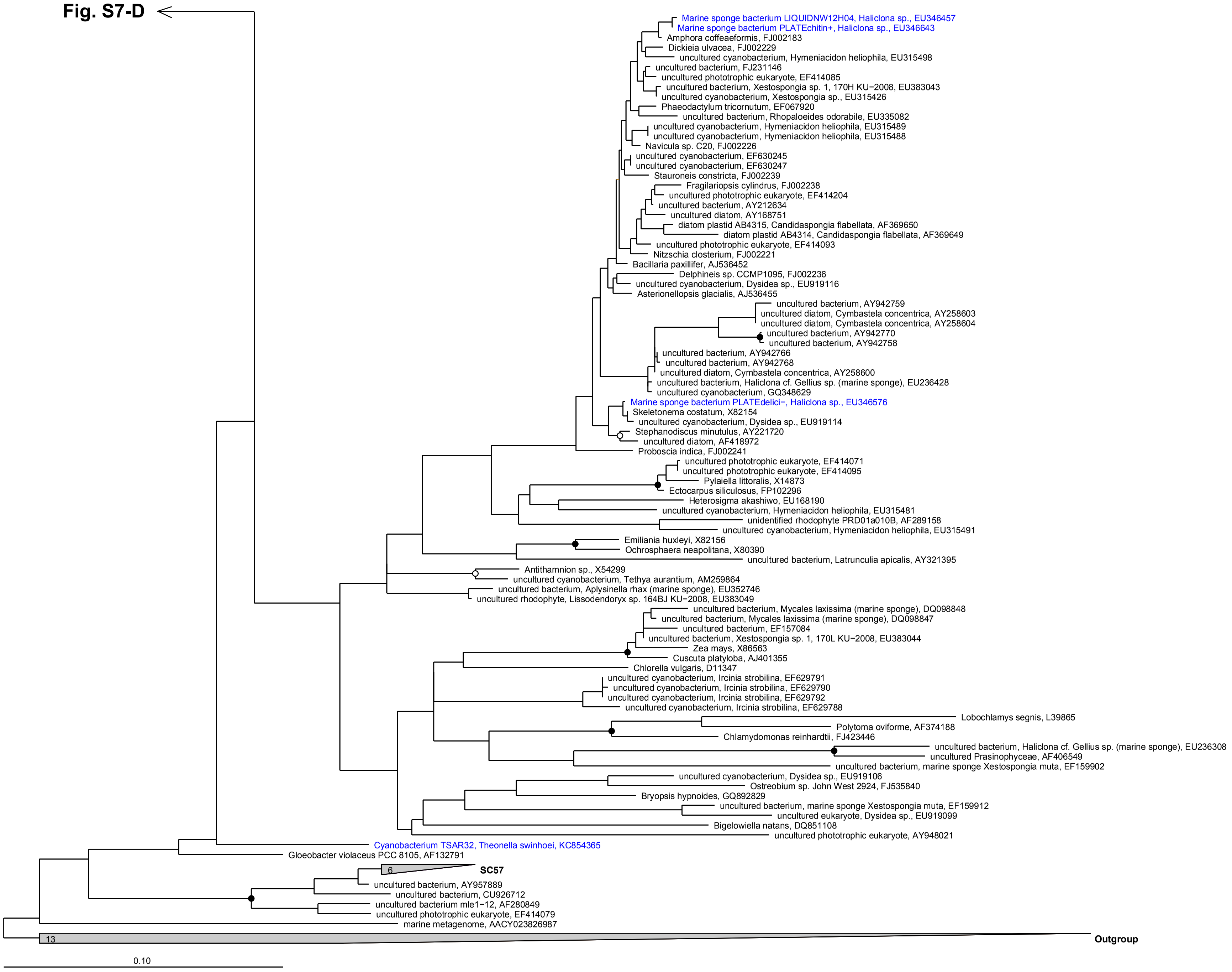

Figure S7-E. 16S rRNA gene-based phylogeny of sponge-associated Cyanobacteria. Details are as provided for Figure S1
