# Supplementary material for: Bacteria Cultivated From Sponges and Bacteria Not Yet Cultivated From Sponges—A Review
Source: Front Microbiol. 2021 Nov 10;12:737925. doi: 10.3389/fmicb.2021.737925 (PMC8634882; doi:10.3389/fmicb.2021.737925)
Supplement: Supplementary file 9 [file Image_9.pdf]

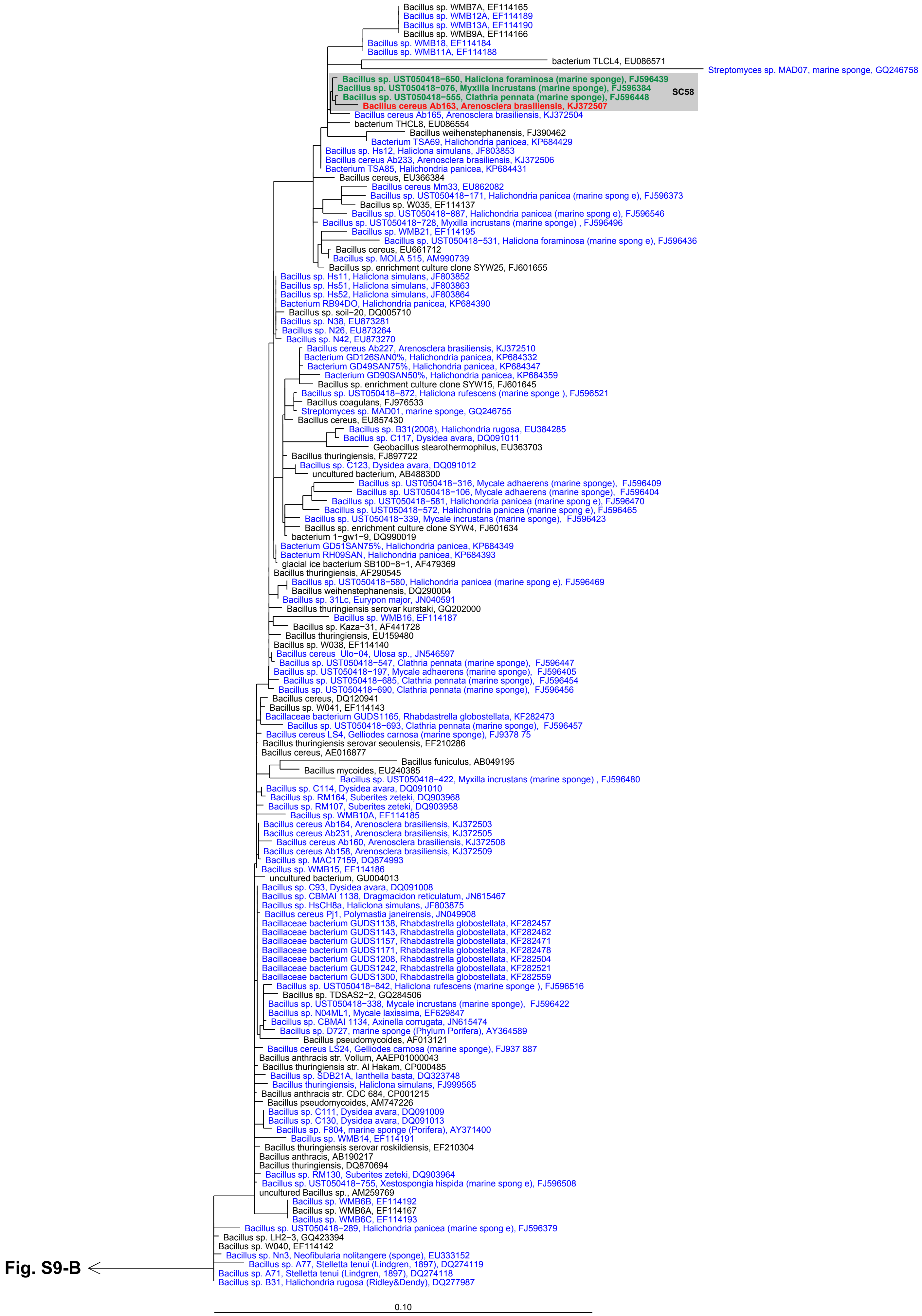

Figure S9-A. 16S rRNA gene-based phylogeny of sponge-associated Firmicutes. Details are as provided for Figure S1

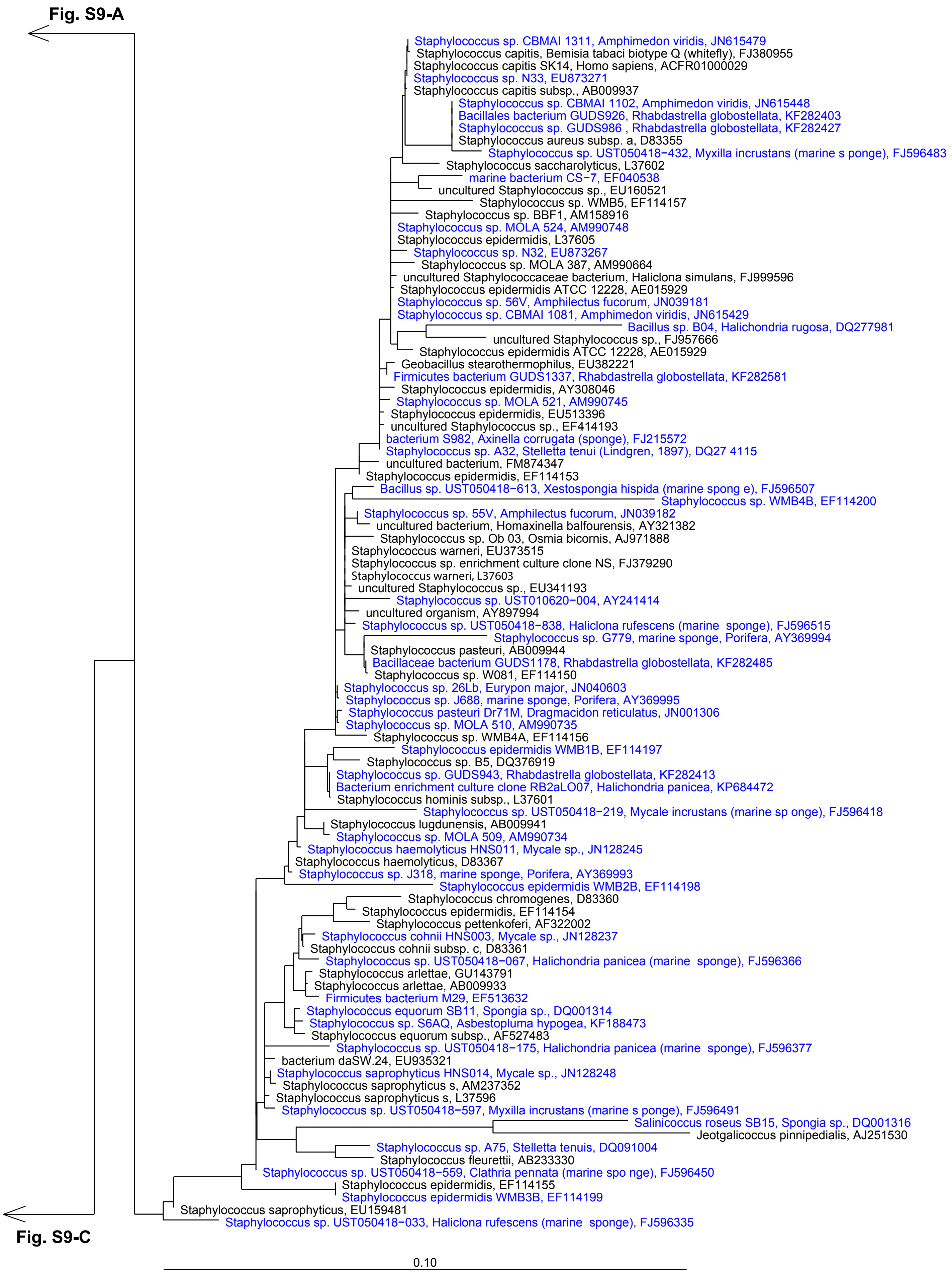

Figure S9-B. 16S rRNA gene-based phylogeny of sponge-associated Firmicutes. Details are as provided for Figure S1

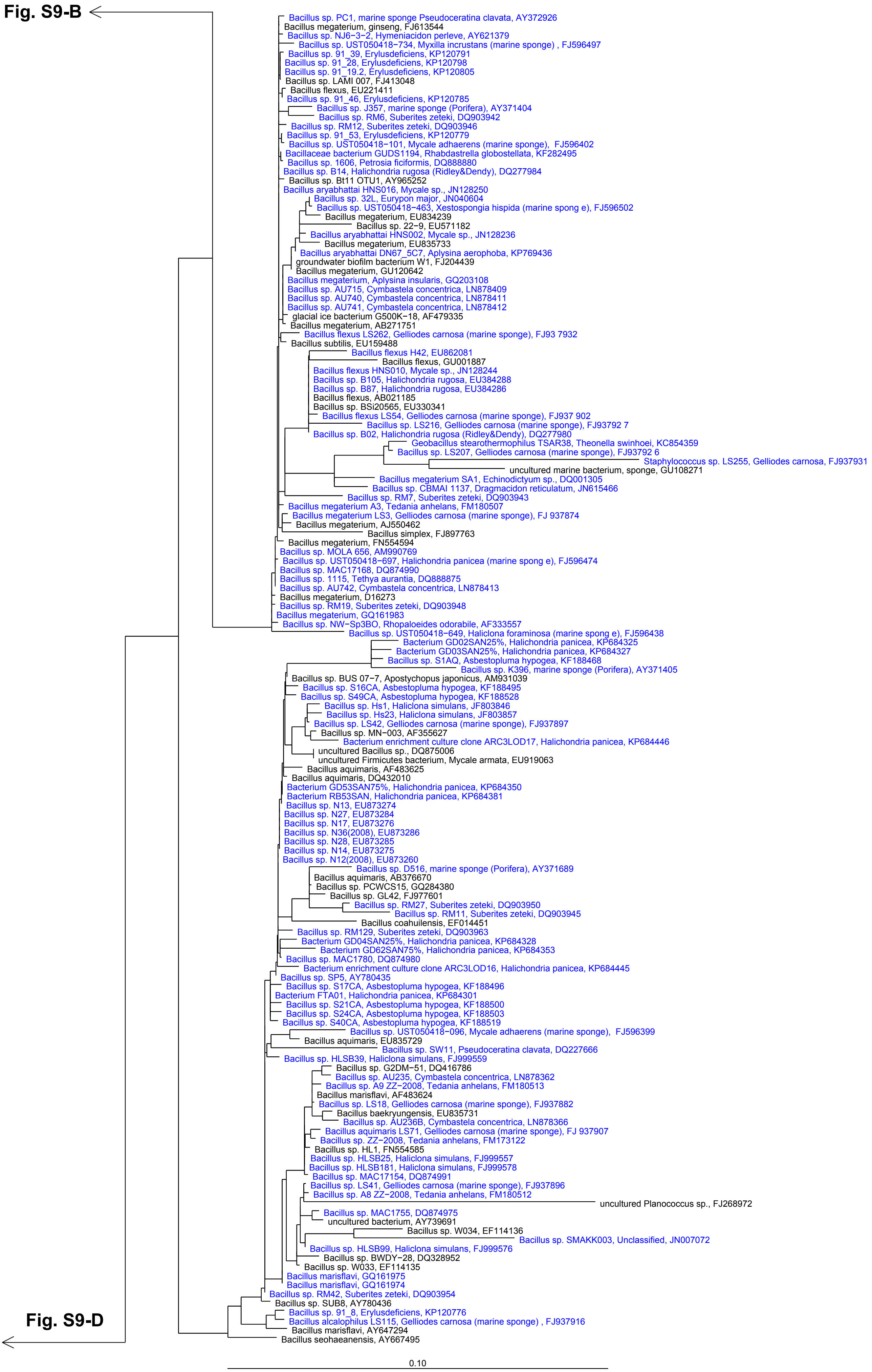

Figure S9-C. 16S rRNA gene-based phylogeny of sponge-associated Firmicutes. Details are as provided for Figure S1

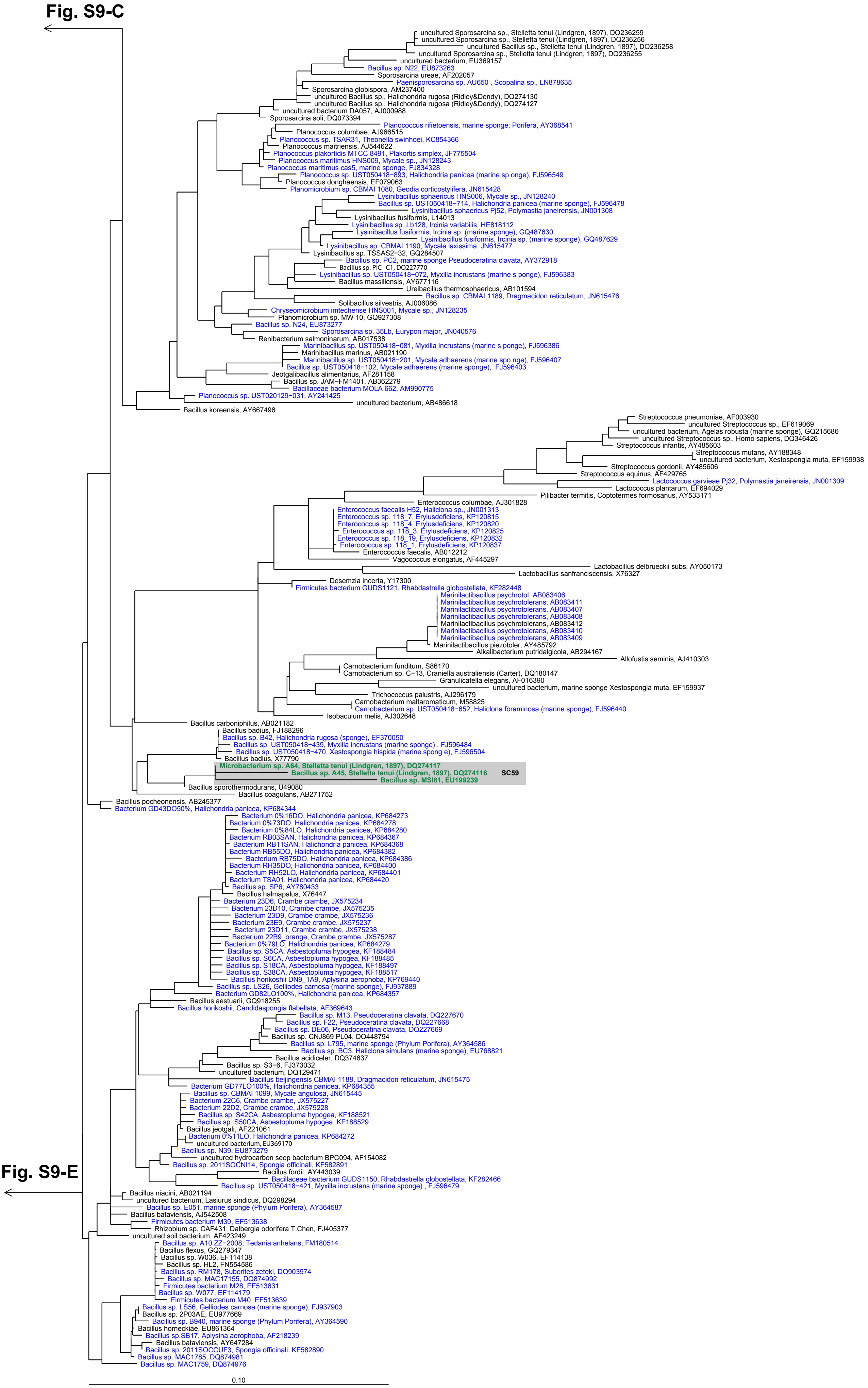

Figure S9-D. 16S rRNA gene-based phylogeny of sponge-associated Firmicutes. Details are as provided for Figure S1

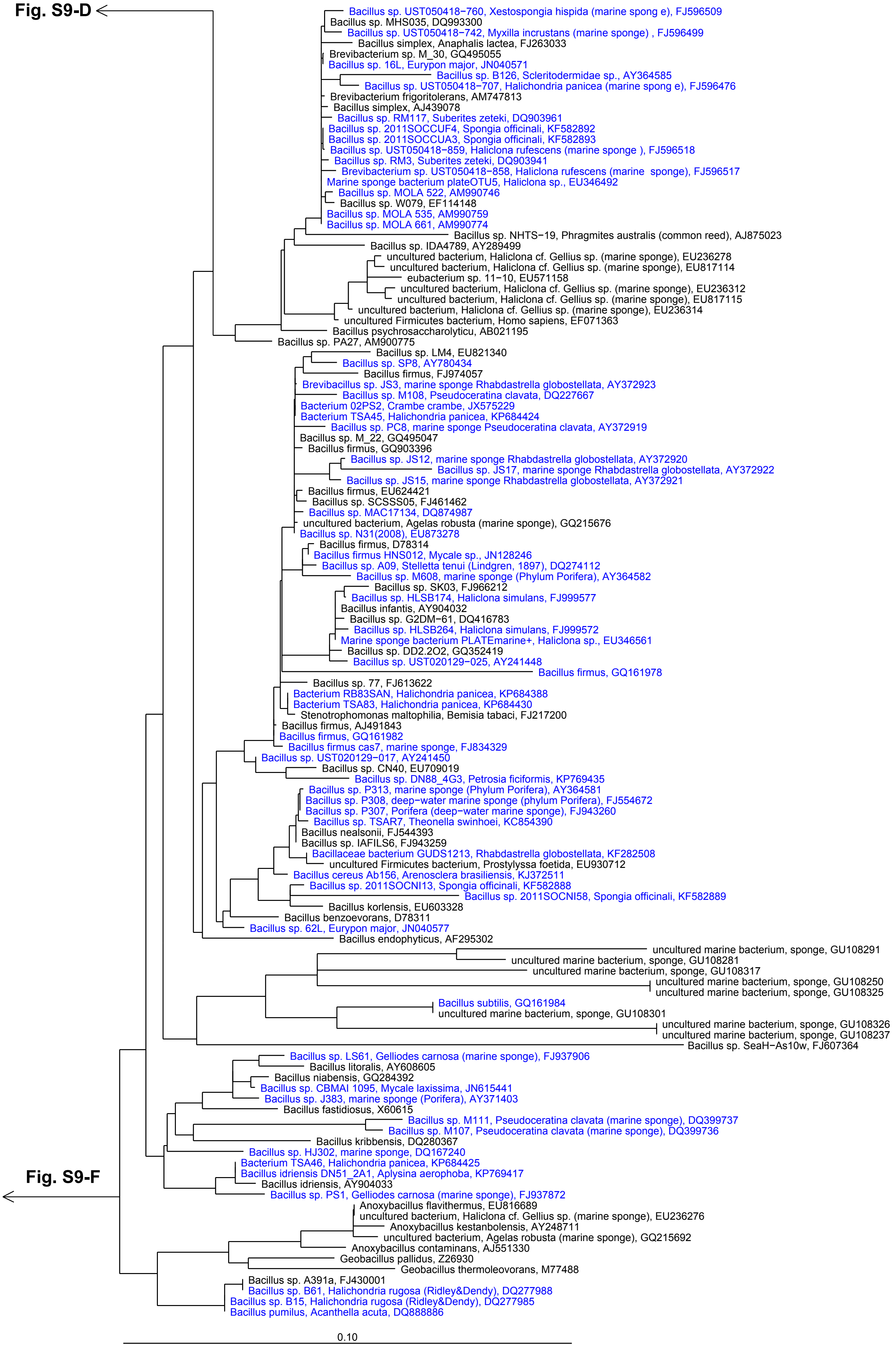

Figure S9-E. 16S rRNA gene-based phylogeny of sponge-associated Firmicutes. Details are as provided for Figure S1

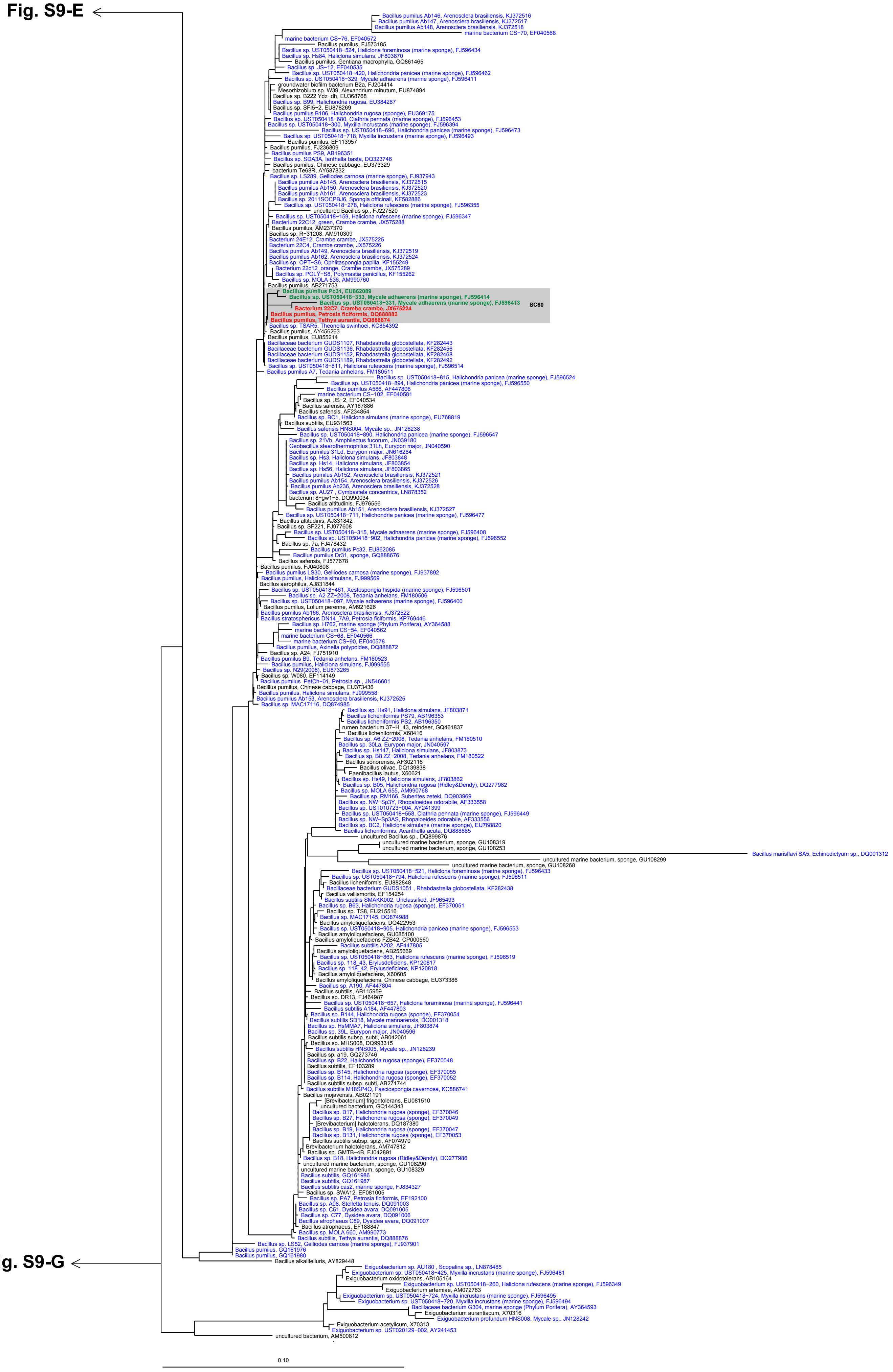

Figure S9-F. 16S rRNA gene-based phylogeny of sponge-associated Firmicutes. Details are as provided for Figure S1

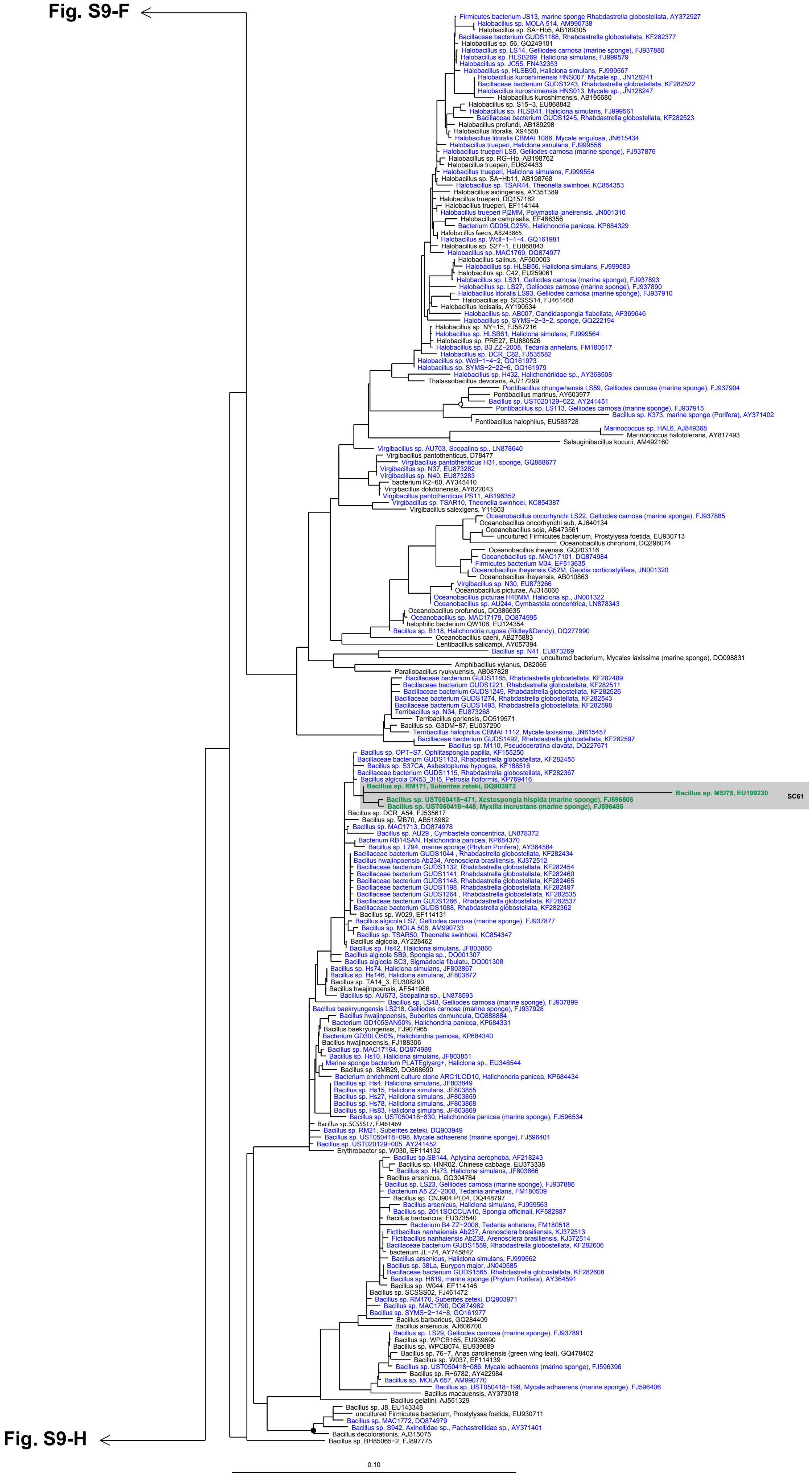

Figure S9-G. 16S rRNA gene-based phylogeny of sponge-associated Firmicutes. Details are as provided for Figure S1

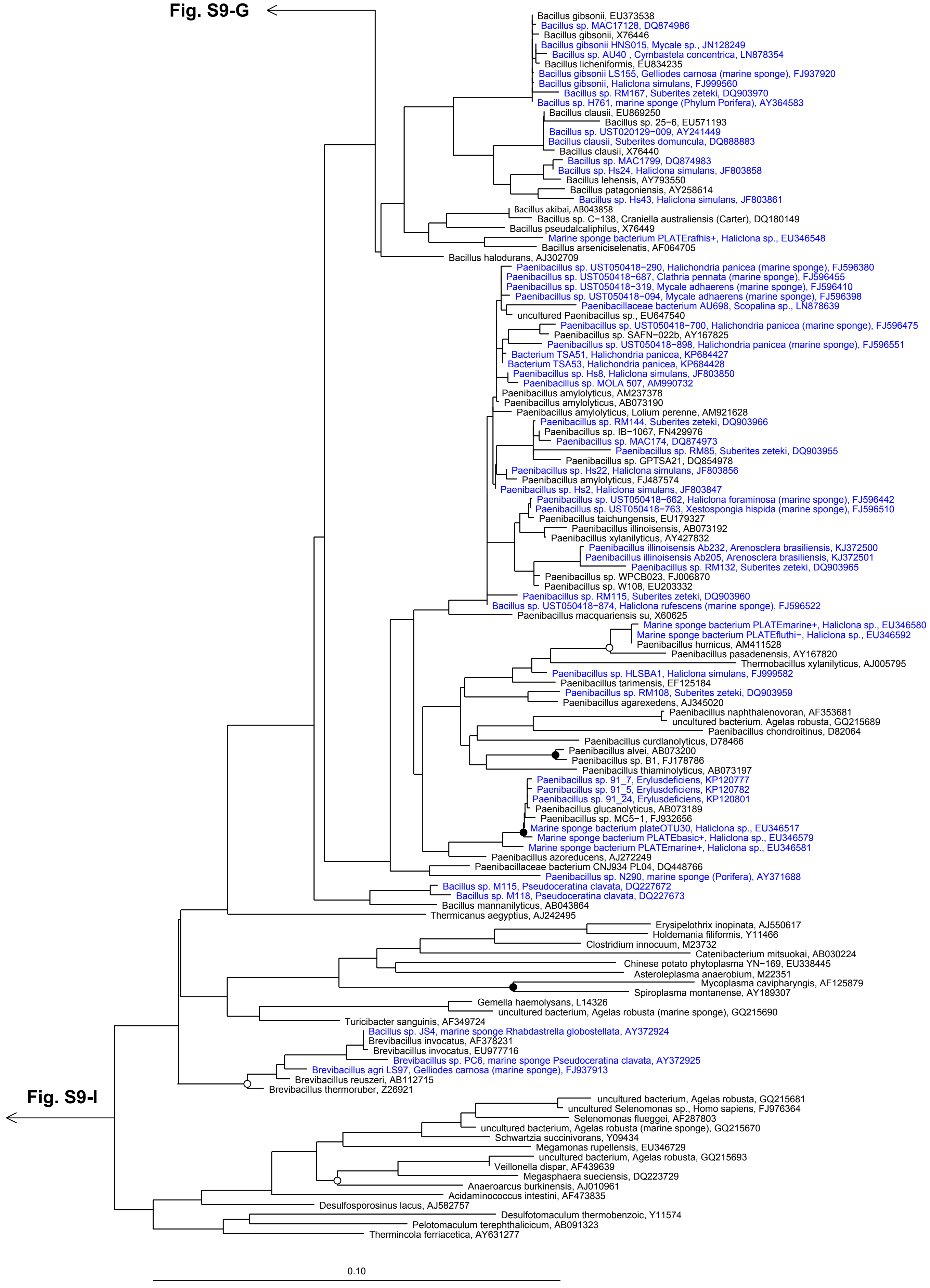

Figure S9-H. 16S rRNA gene-based phylogeny of sponge-associated Firmicutes. Details are as provided for Figure S1

Fig. S9-H

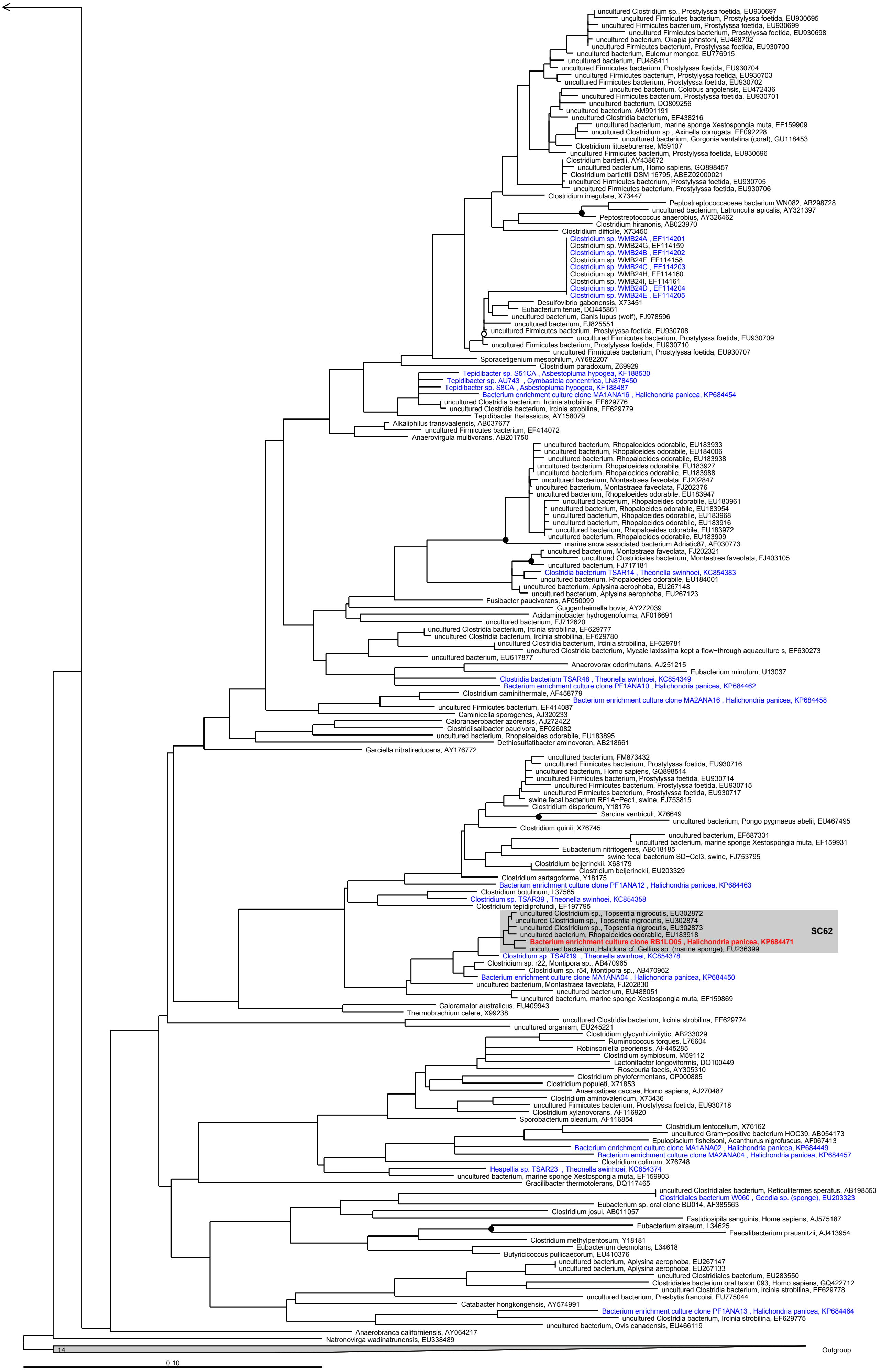

Figure S9-I. 16S rRNA gene-based phylogeny of sponge-associated Firmicutes. Details are as provided for Figure S1
