# Supplementary material for: Bacteria Cultivated From Sponges and Bacteria Not Yet Cultivated From Sponges—A Review
Source: Front Microbiol. 2021 Nov 10;12:737925. doi: 10.3389/fmicb.2021.737925 (PMC8634882; doi:10.3389/fmicb.2021.737925)
Supplement: Supplementary file 10 [file Image_10.pdf]

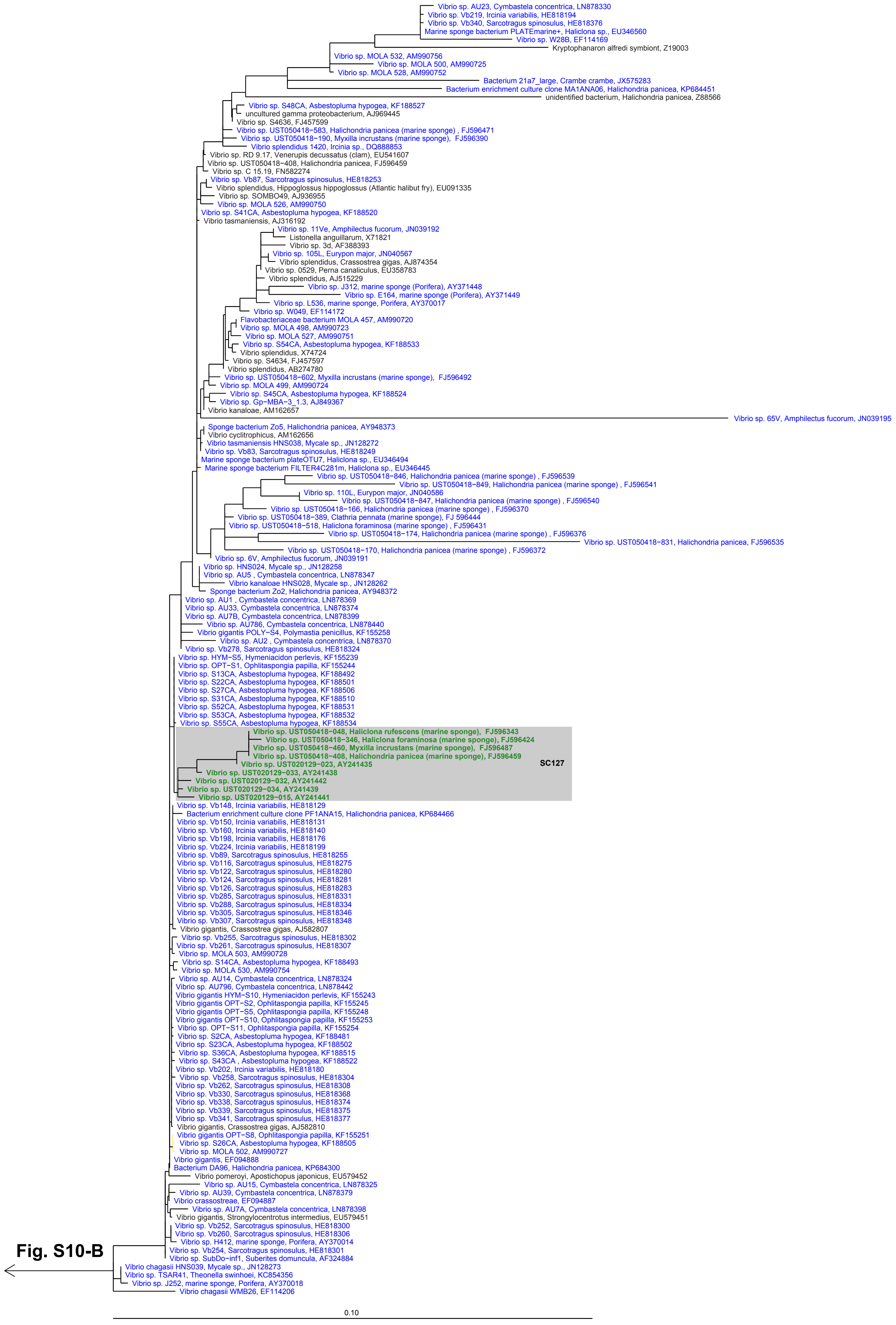


**Figure S10-A** 16S rRNA gene-based phylogeny of sponge-associated Gammaproteobacteria. Details are as provided for Figure S1

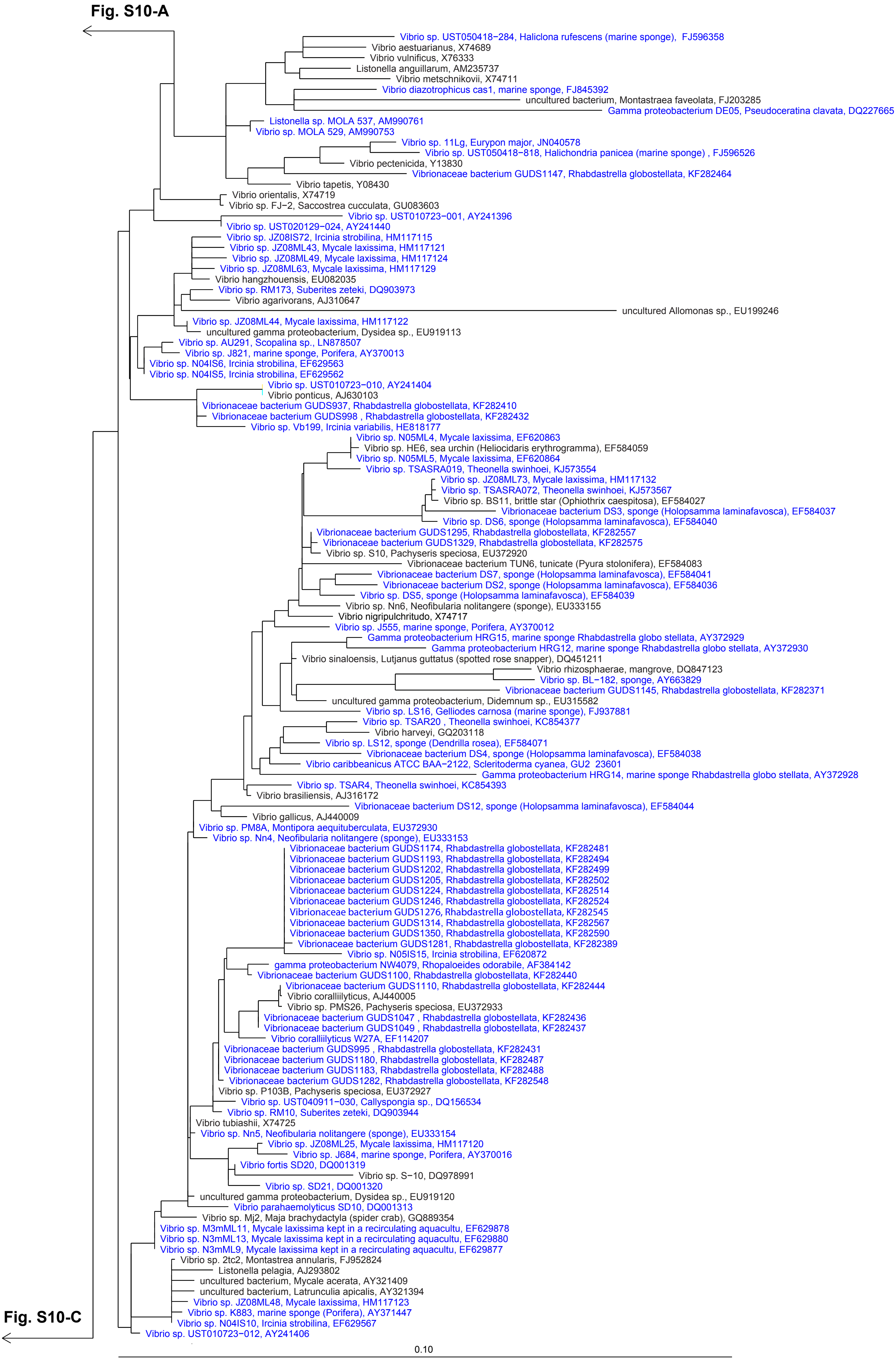


**Figure S10-B.** 16S rRNA gene-based phylogeny of sponge-associated Gammaproteobacteria. Details are as provided for Figure S1

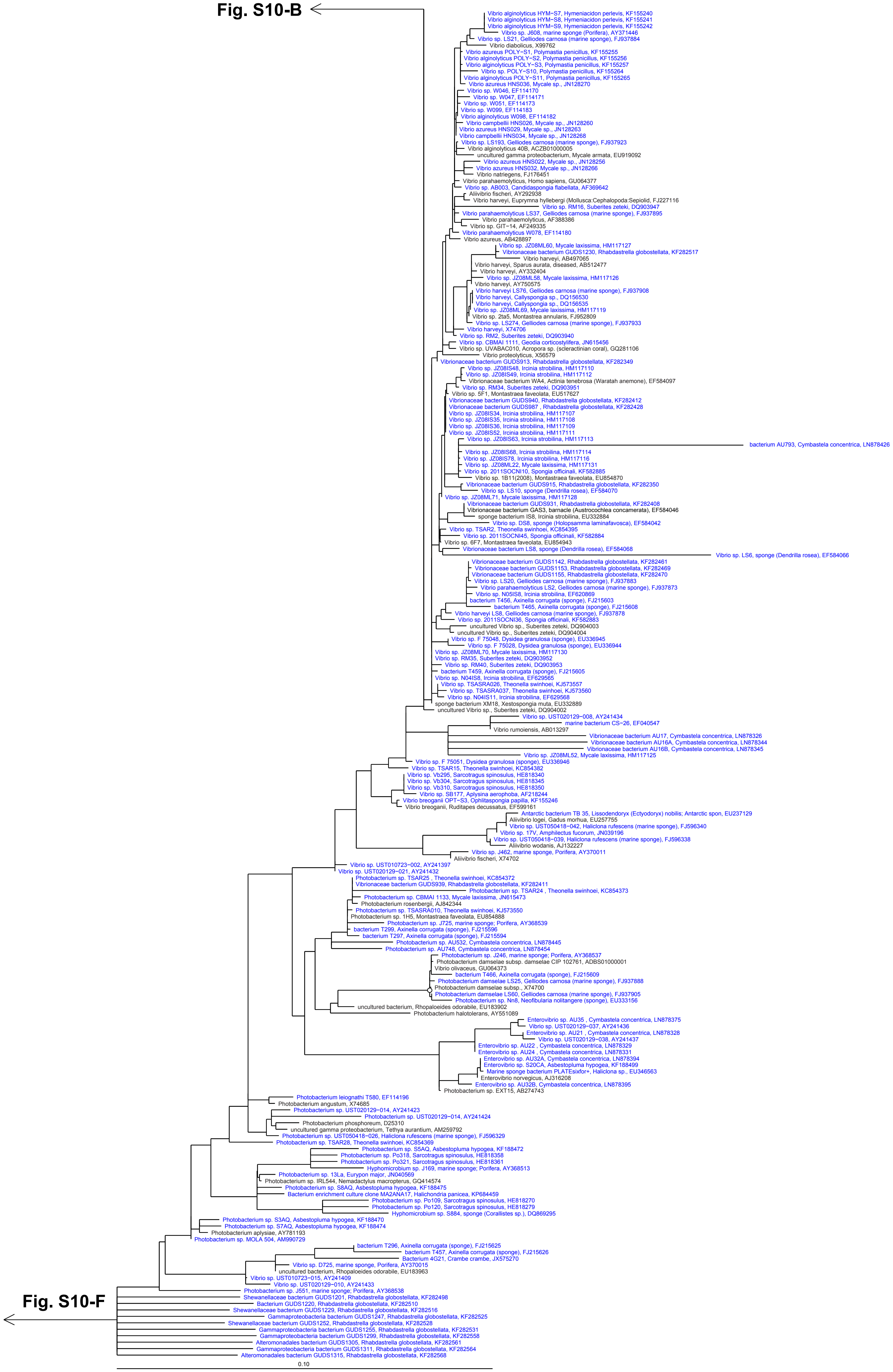


**Figure S10-C.** 16S rRNA gene-based phylogeny of sponge-associated Gammaproteobacteria. Details are as provided for Figure S1

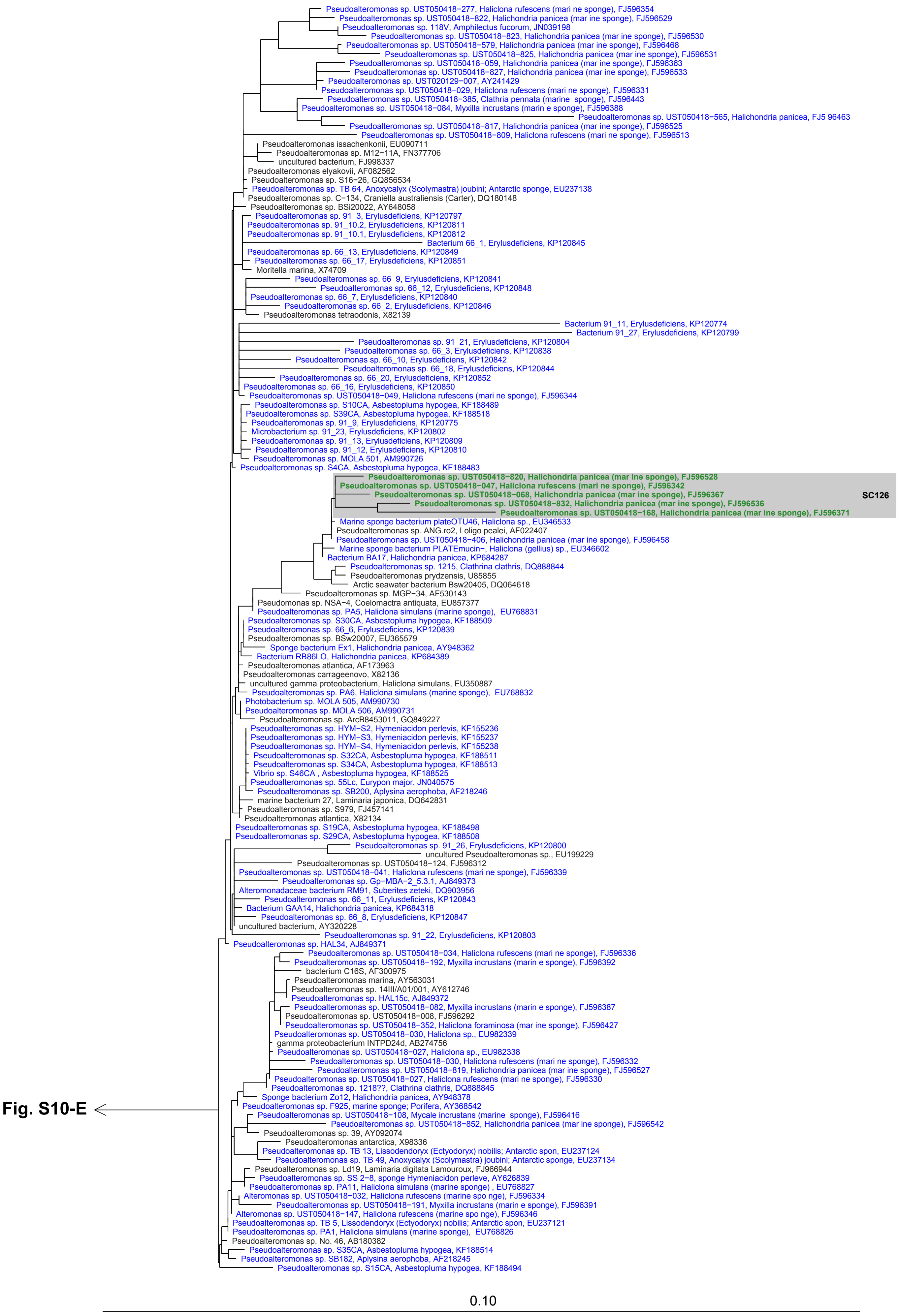


**Figure S10-D.** 16S rRNA gene-based phylogeny of sponge-associated Gammaproteobacteria. Details are as provided for Figure S1

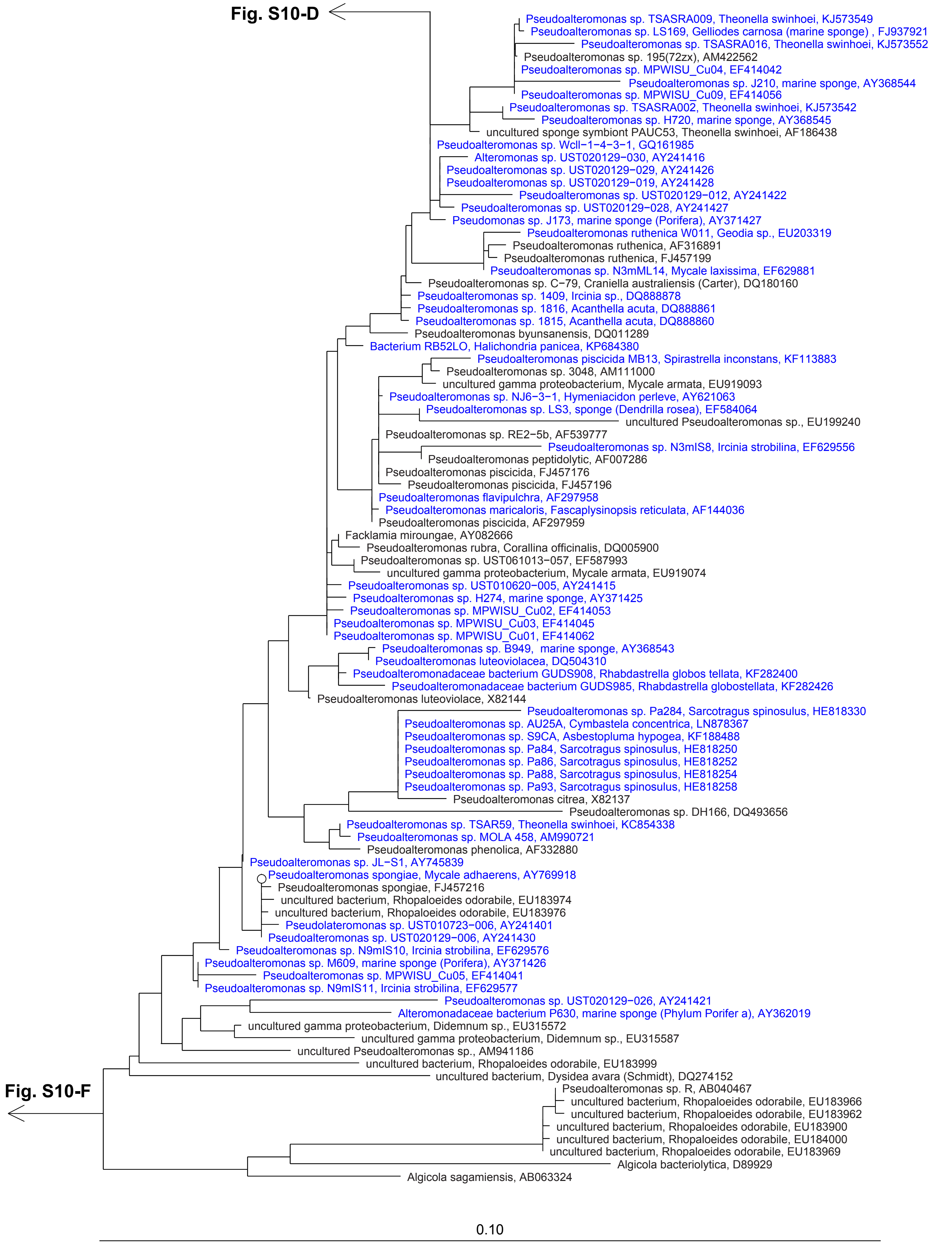


**Figure S10-E.** 16S rRNA gene-based phylogeny of sponge-associated Gammaproteobacteria. Details are as provided for Figure S1

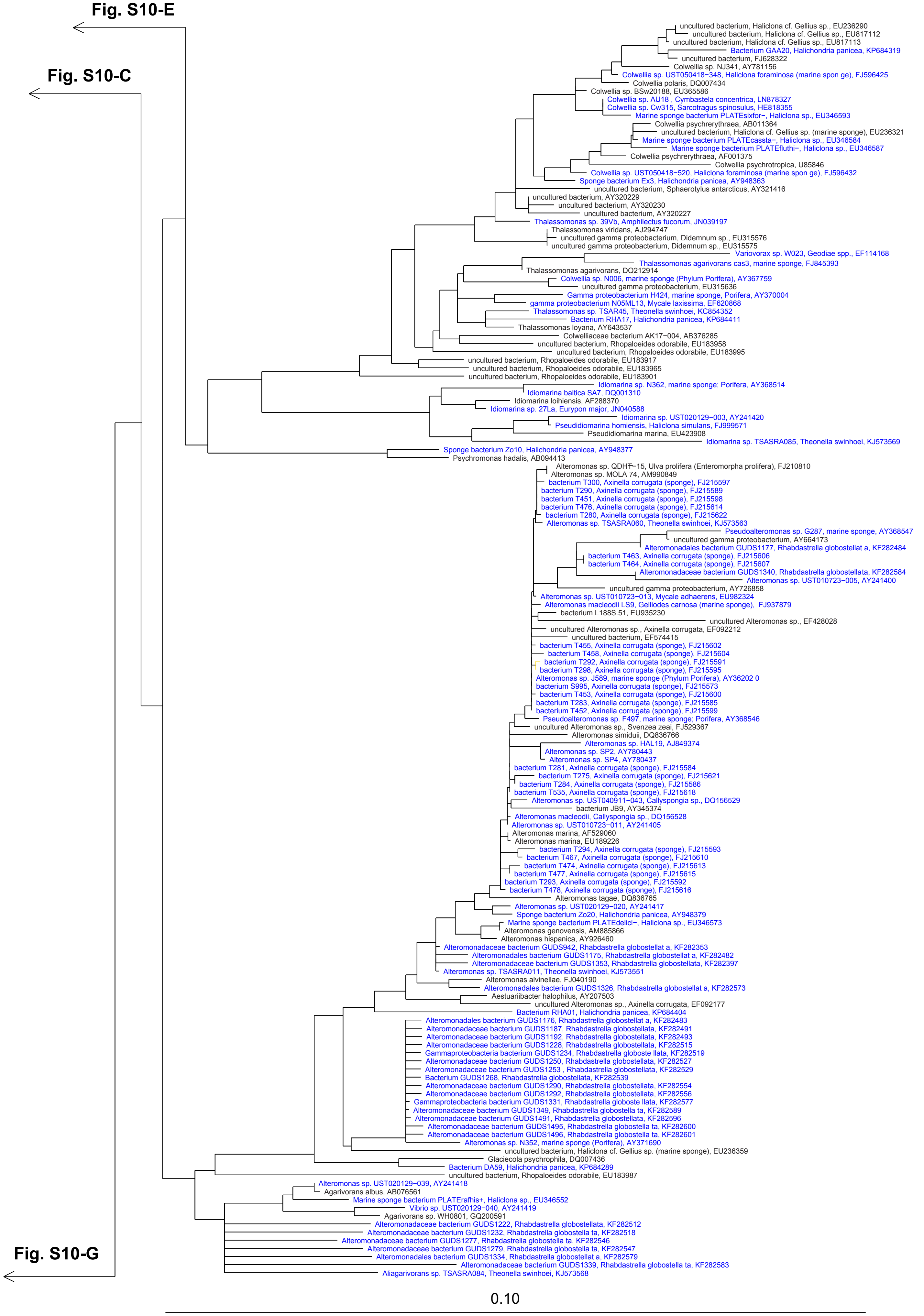


**Figure S10-F.** 16S rRNA gene-based phylogeny of sponge-associated Gammaproteobacteria. Details are as provided for Figure S1

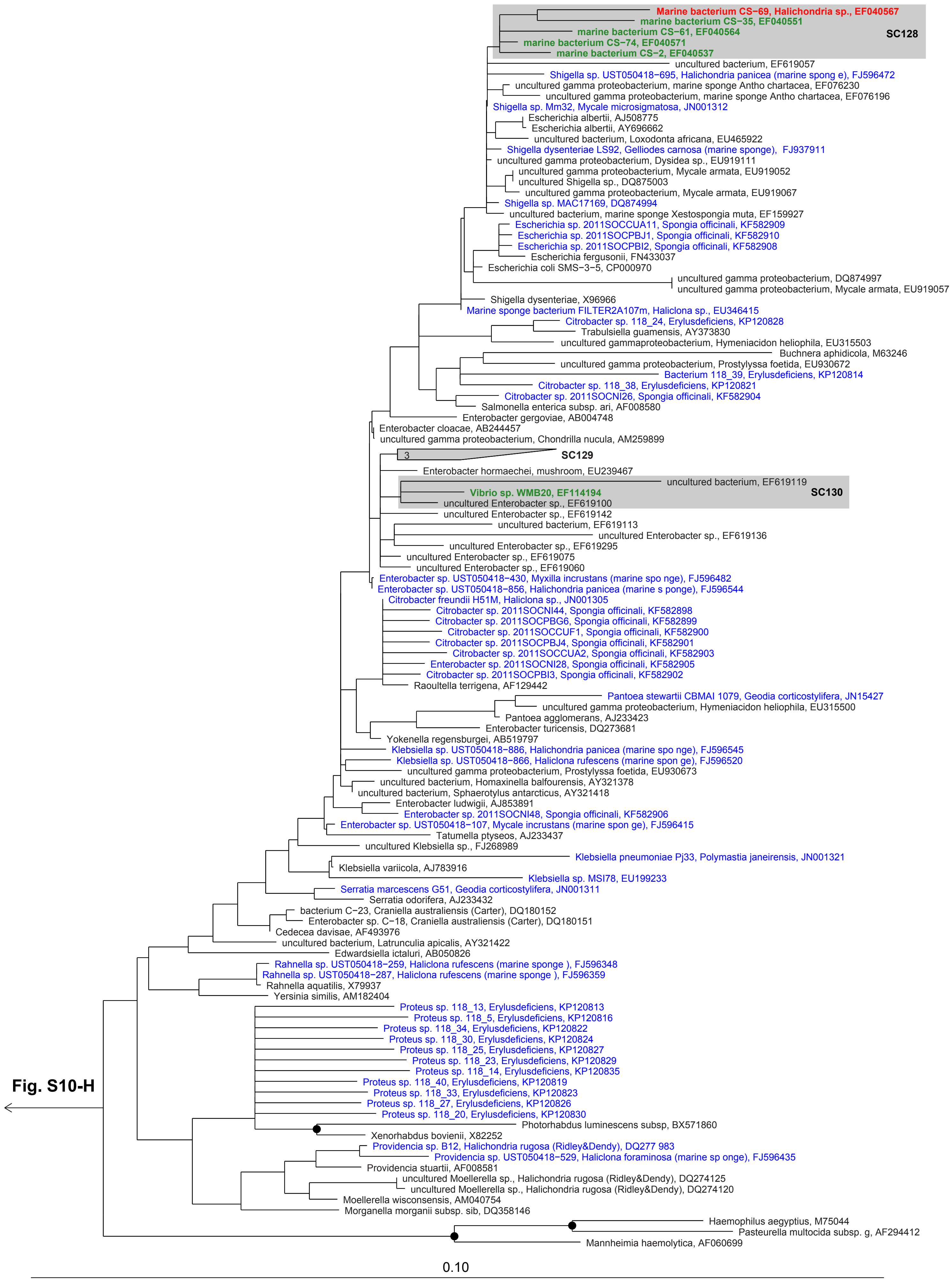


**Figure S10-G.** 16S rRNA gene-based phylogeny of sponge-associated Gammaproteobacteria. Details are as provided for Figure S1

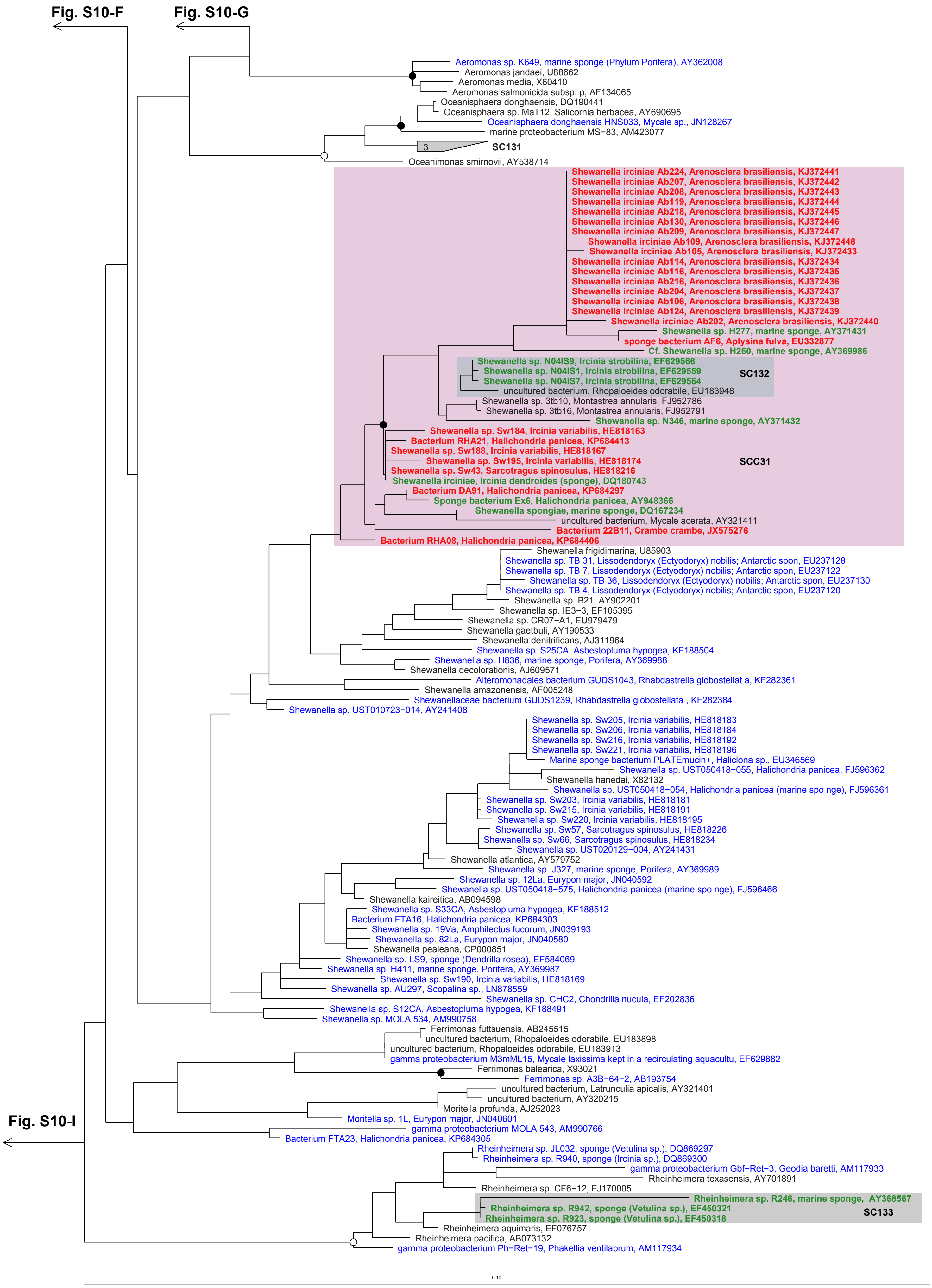


**Figure S10-H.** 16S rRNA gene-based phylogeny of sponge-associated Gammaproteobacteria. Details are as provided for Figure S1

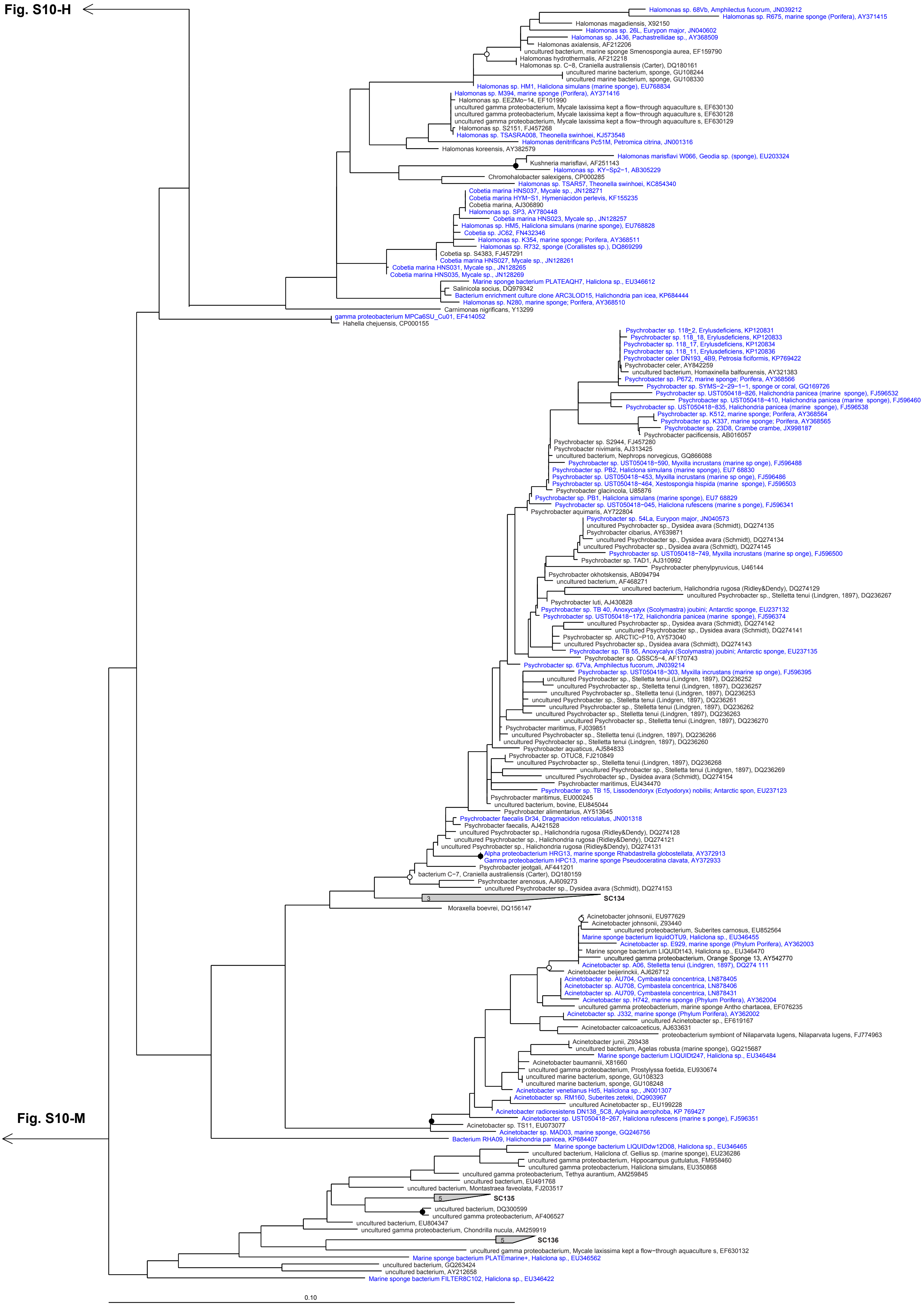


**Figure S10-I.** 16S rRNA gene-based phylogeny of sponge-associated Gammaproteobacteria. Details are as provided for Figure S1

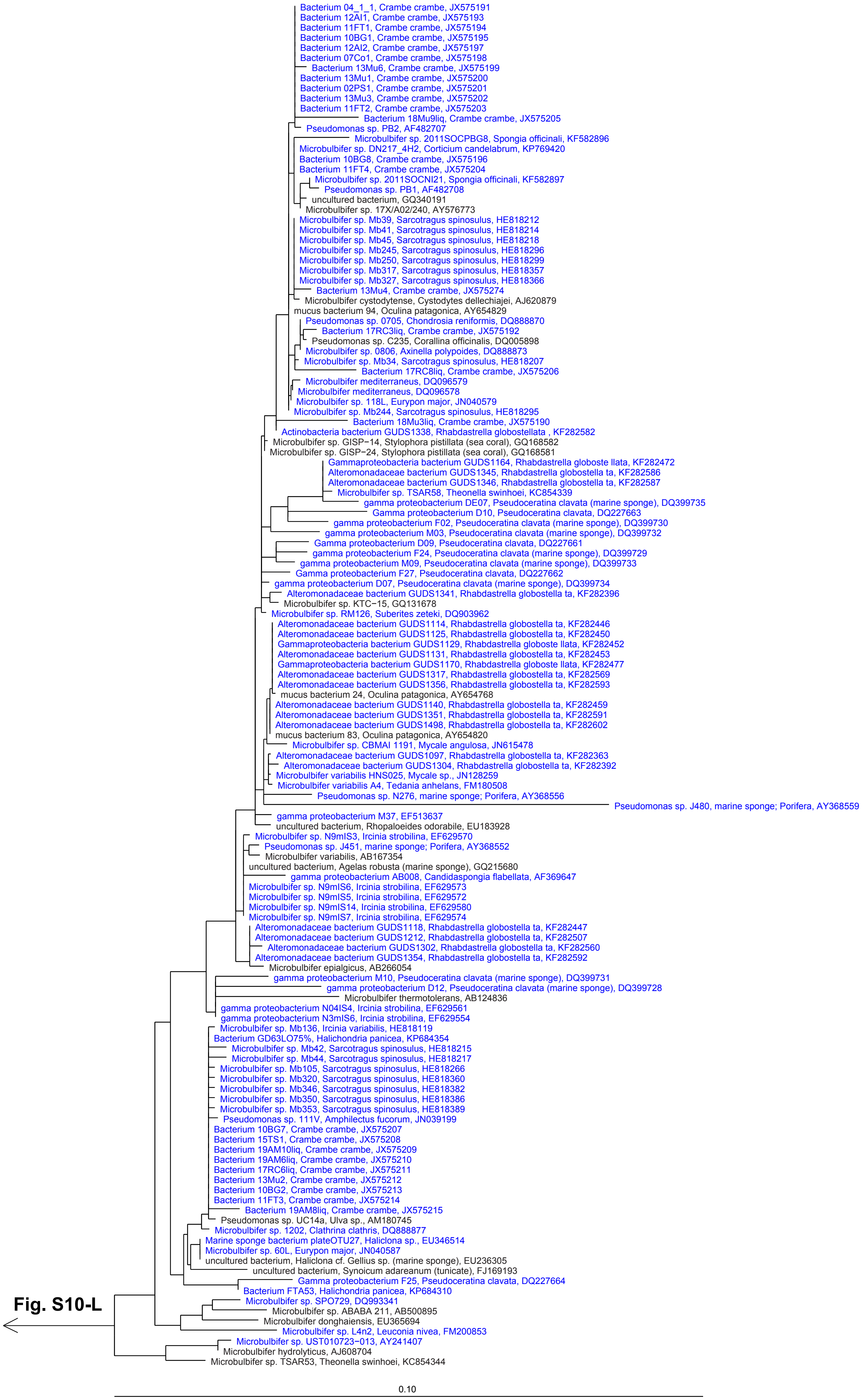


**Figure S10-K.** 16S rRNA gene-based phylogeny of sponge-associated Gammaproteobacteria. Details are as provided for Figure S1

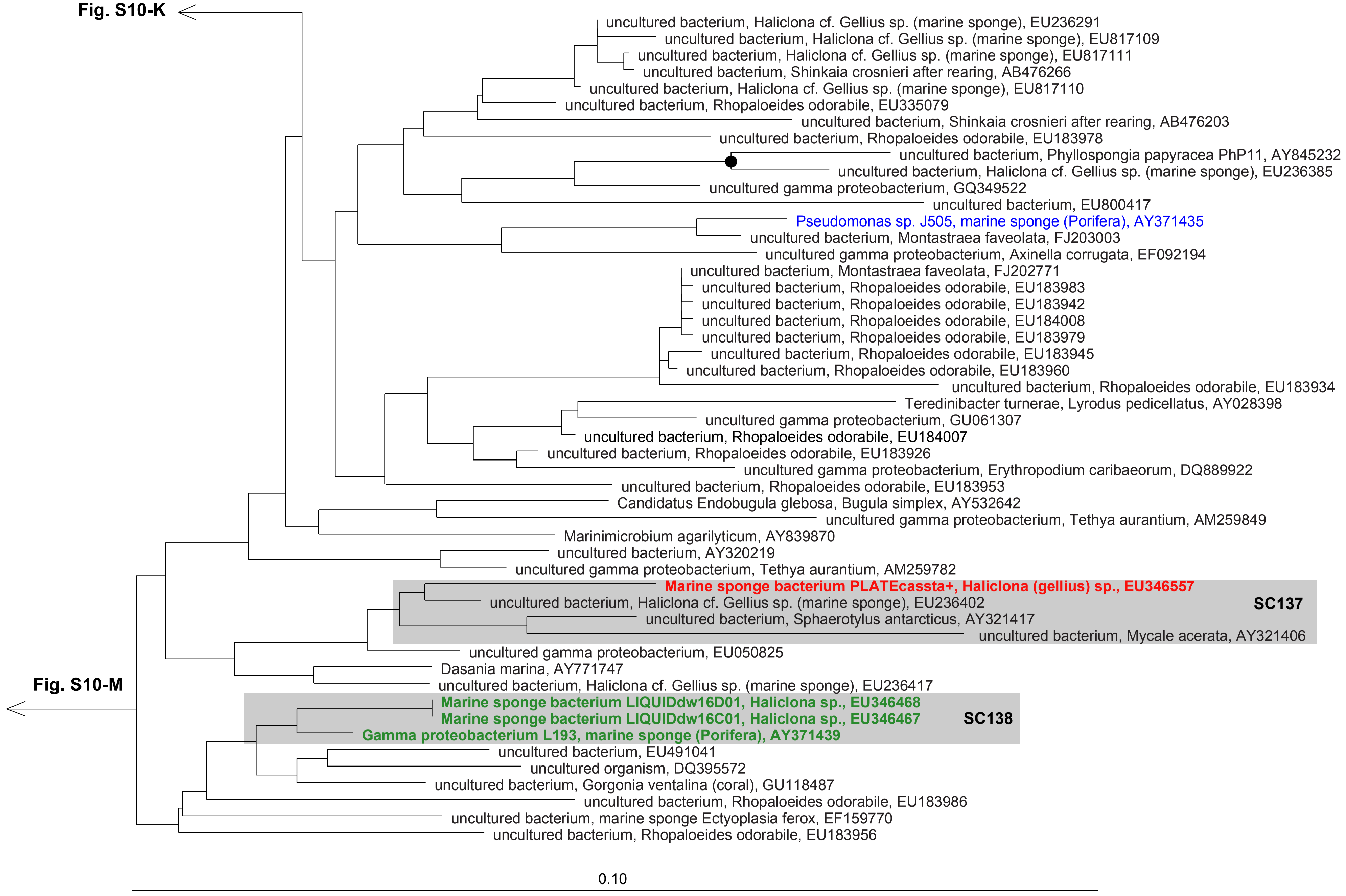


**Figure S10-L.** 16S rRNA gene-based phylogeny of sponge-associated Gammaproteobacteria. Details are as provided for Figure S1

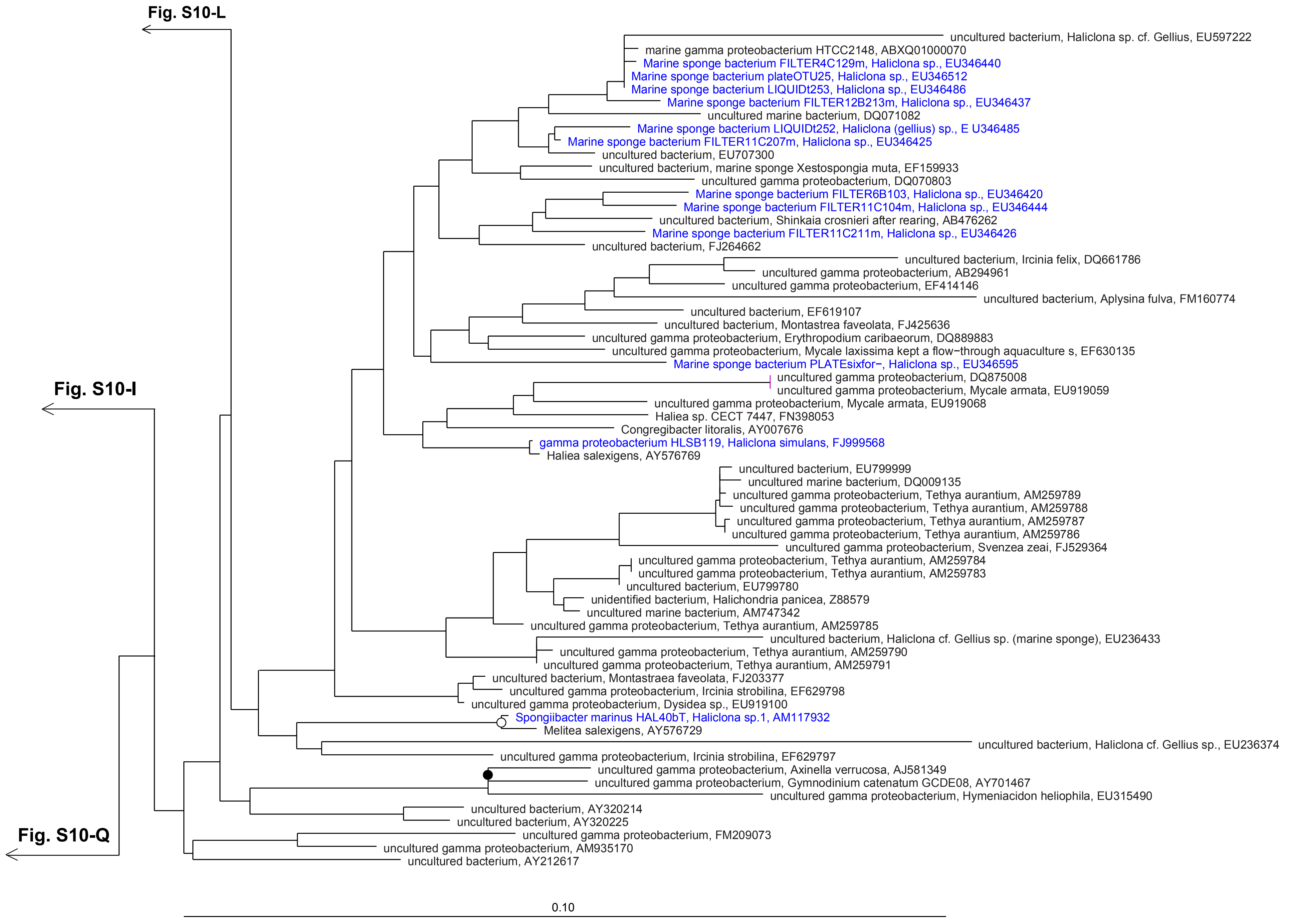


**Figure S10-M.** 16S rRNA gene-based phylogeny of sponge-associated Gammaproteobacteria. Details are as provided for Figure S1

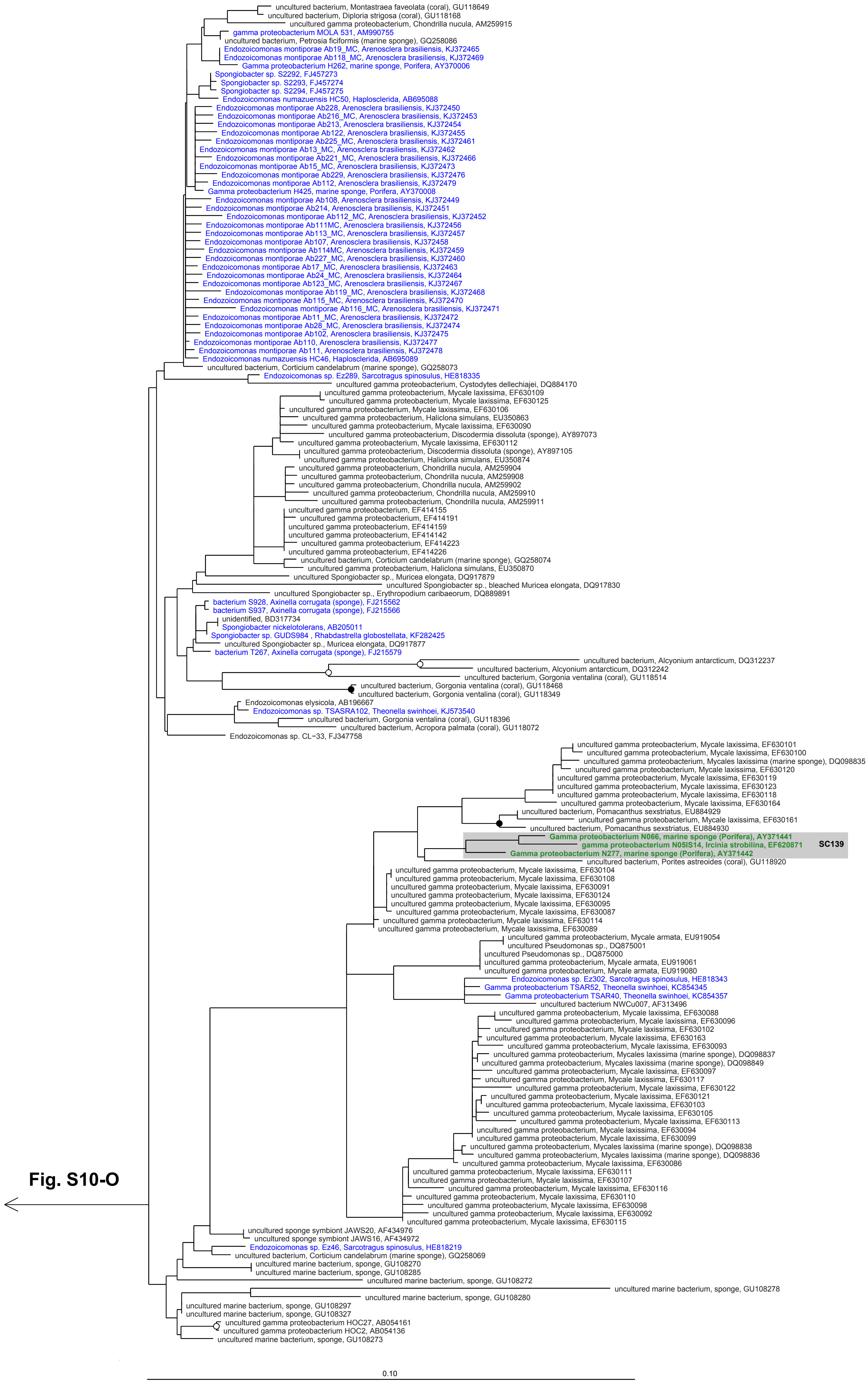


**Figure S10-N.** 16S rRNA gene-based phylogeny of sponge-associated Gammaproteobacteria. Details are as provided for Figure S1

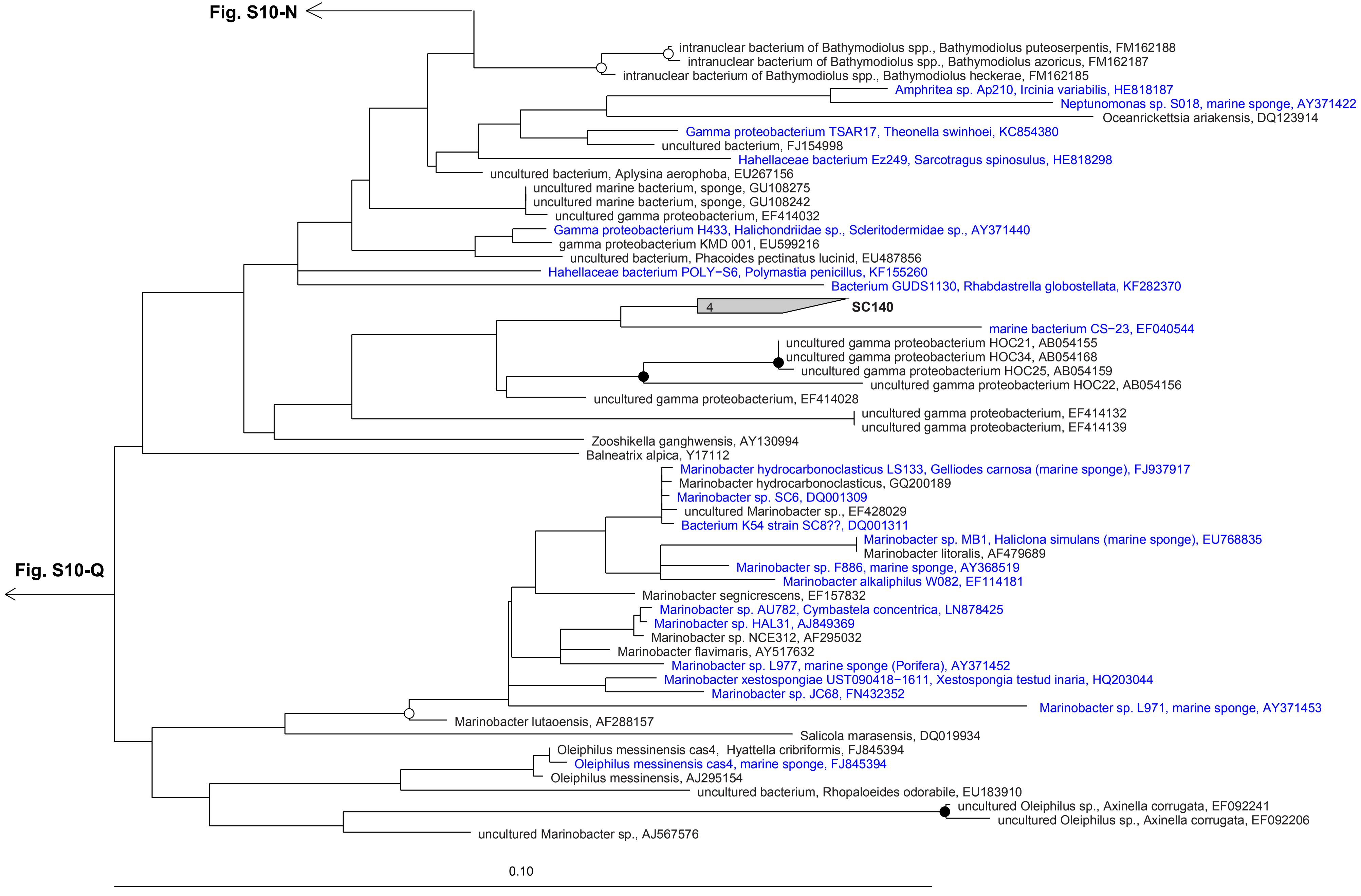


**Figure S10-O.** 16S rRNA gene-based phylogeny of sponge-associated Gammaproteobacteria. Details are as provided for Figure S1

**Fig. S10-Q**

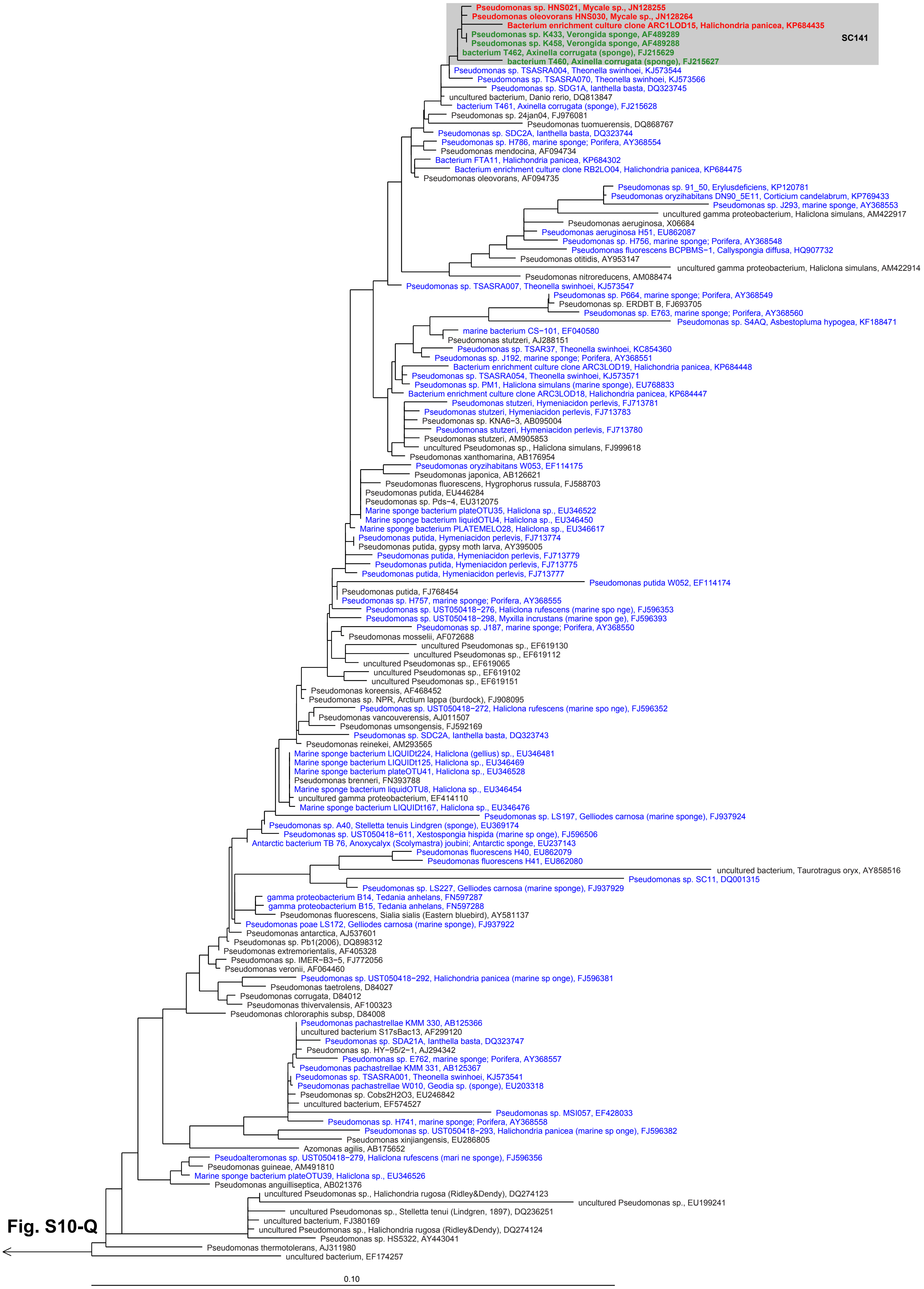


**Figure S10-P.** 16S rRNA gene-based phylogeny of sponge-associated Gammaproteobacteria. Details are as provided for Figure S1

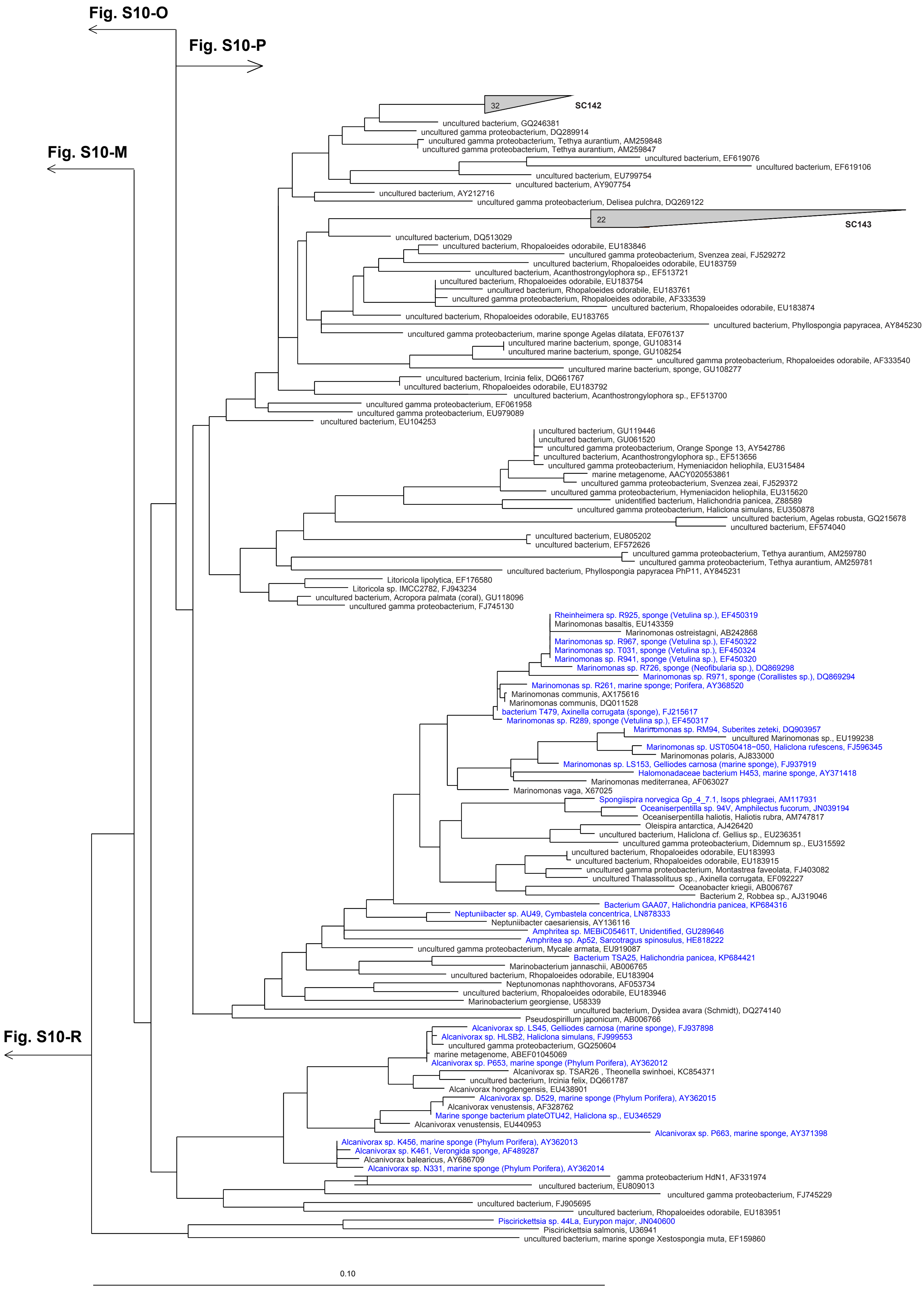


**Figure S10-Q.** 16S rRNA gene-based phylogeny of sponge-associated Gammaproteobacteria. Details are as provided for Figure S1

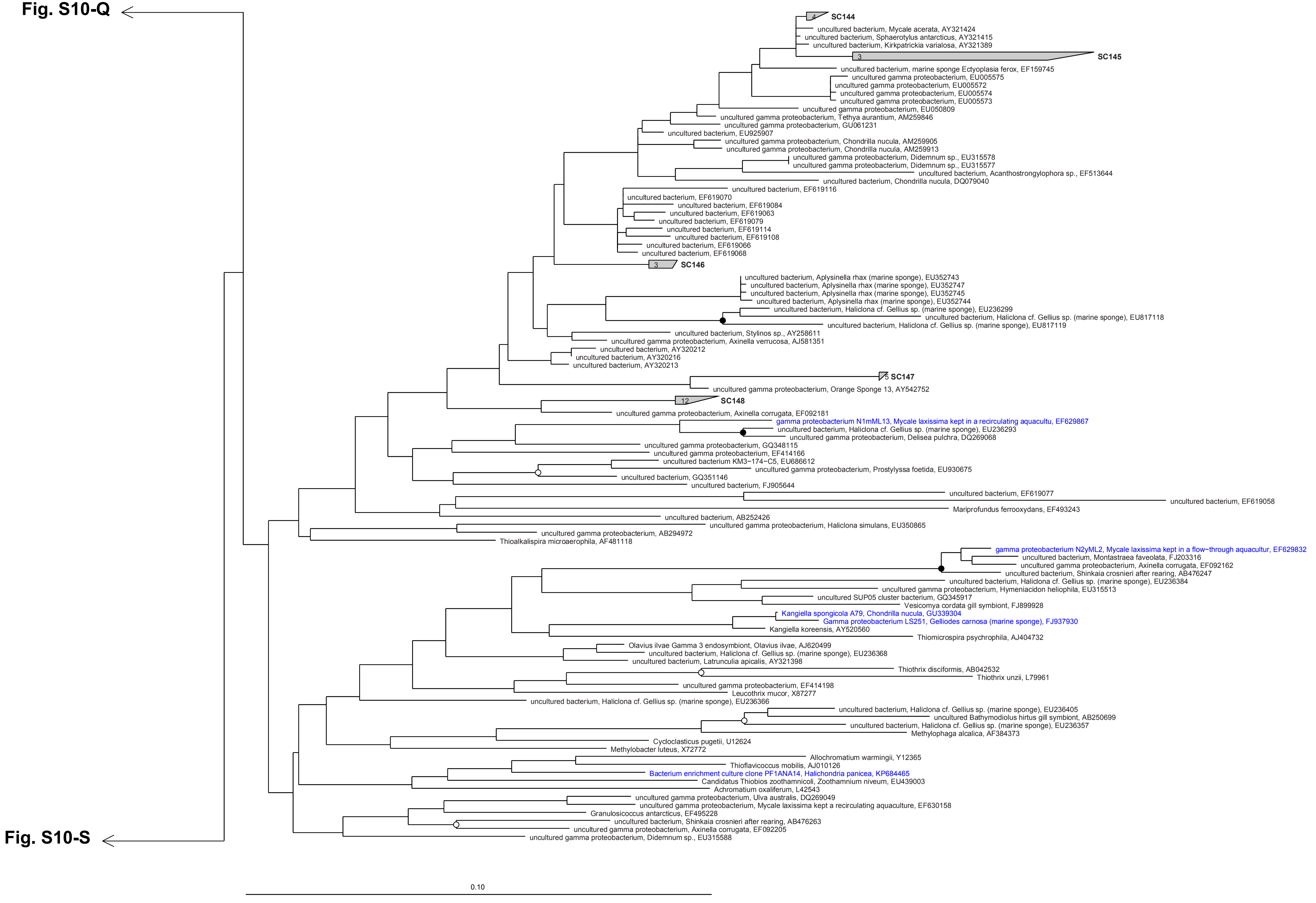


**Figure S10-R.** 16S rRNA gene-based phylogeny of sponge-associated Gammaproteobacteria. Details are as provided for Figure S1

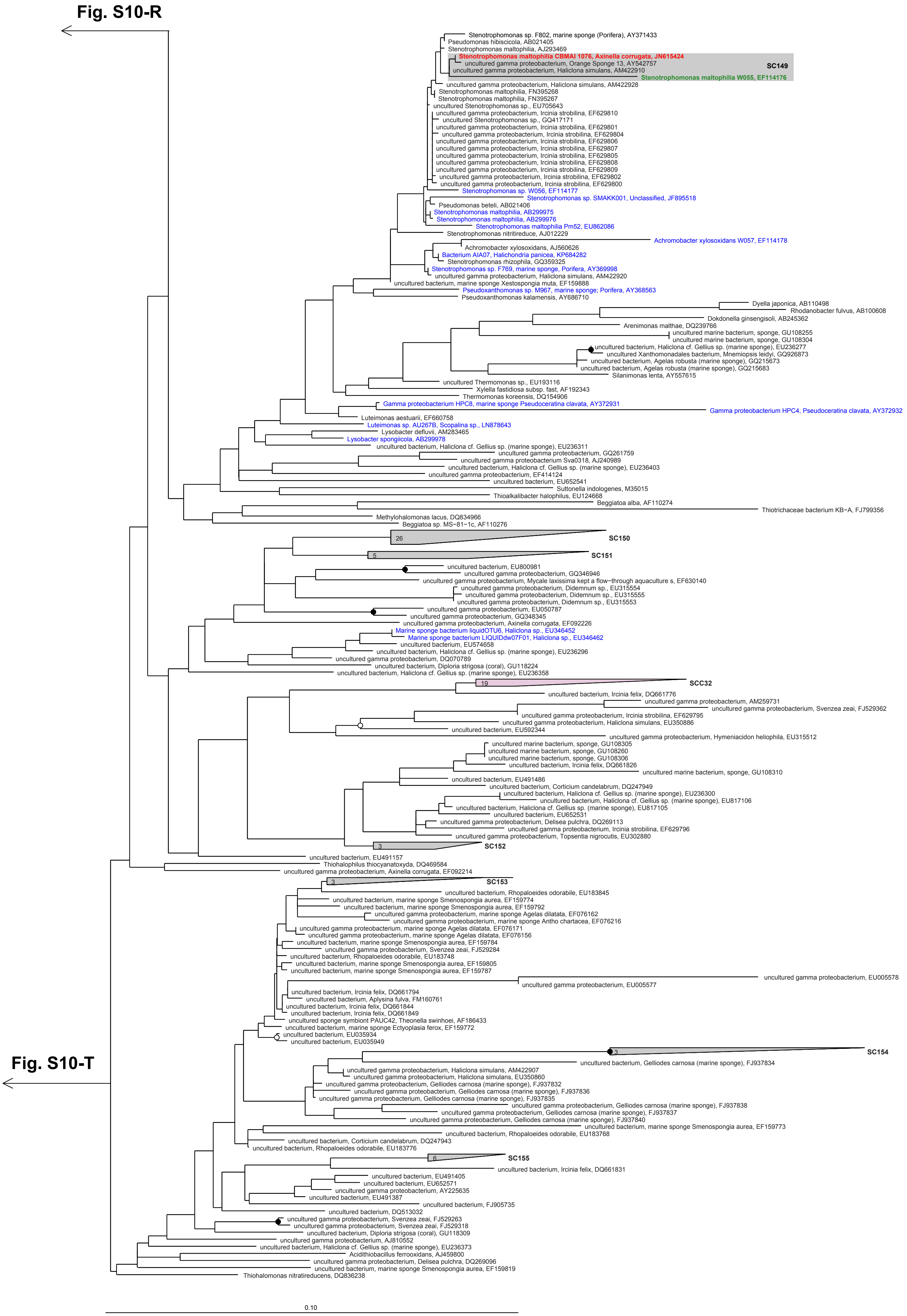


**Figure S10-S.** 16S rRNA gene-based phylogeny of sponge-associated Gammaproteobacteria. Details are as provided for Figure S1

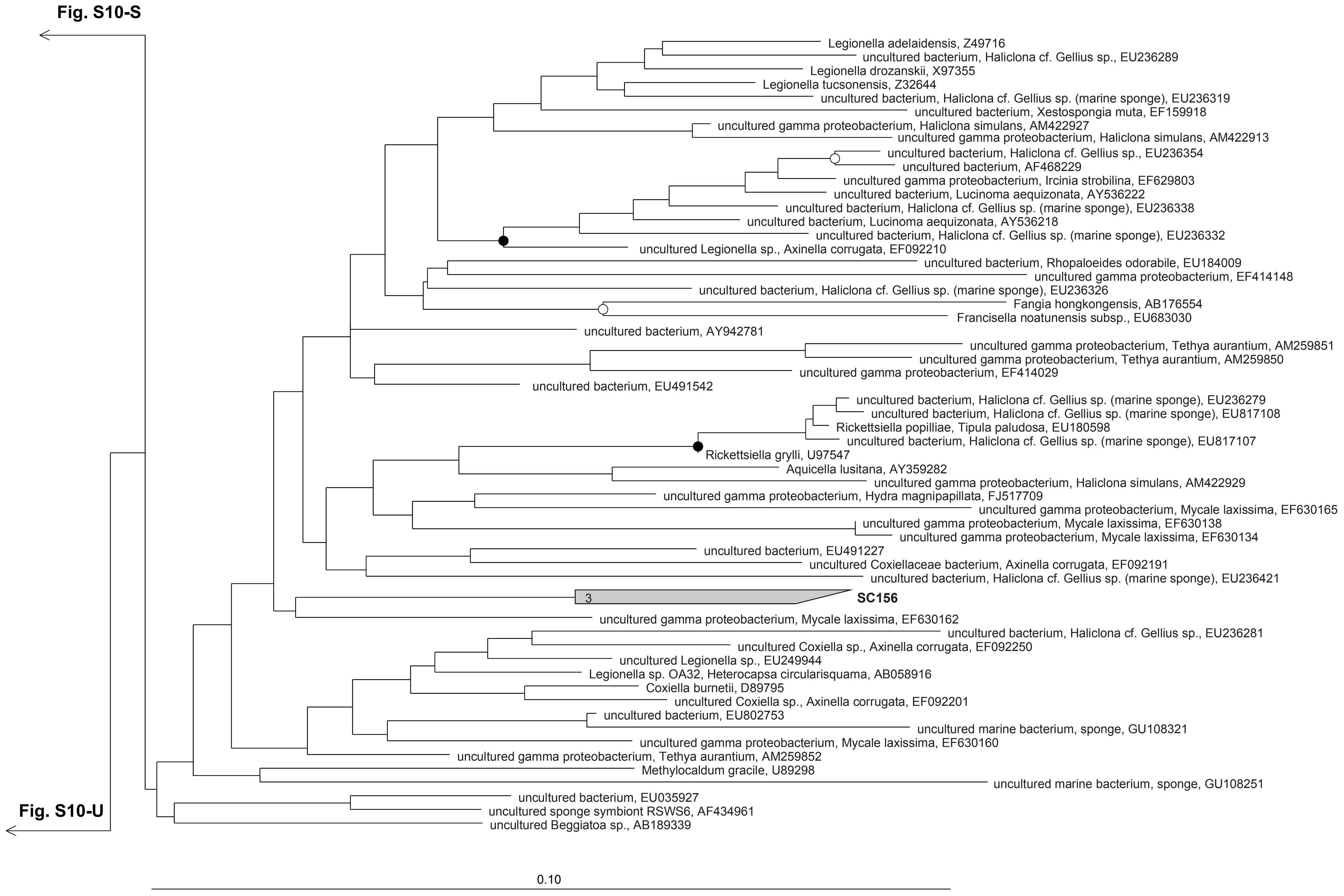


**Figure S10-T.** 16S rRNA gene-based phylogeny of sponge-associated Gammaproteobacteria. Details are as provided for Figure S1

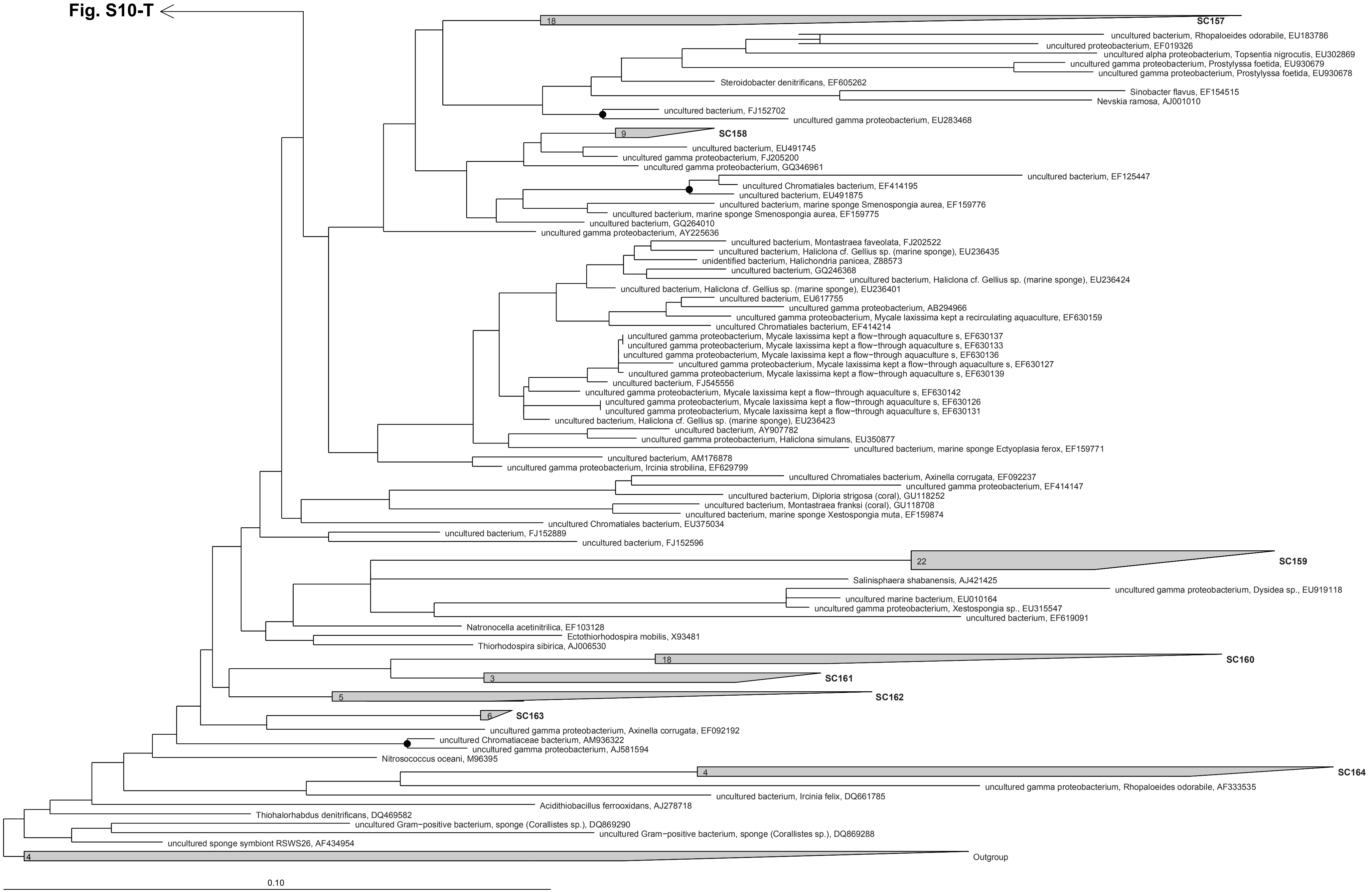


**Figure S10-U.** 16S rRNA gene-based phylogeny of sponge-associated Gammaproteobacteria. Details are as provided for Figure S1
