# Supplementary material for: Bacteria Cultivated From Sponges and Bacteria Not Yet Cultivated From Sponges—A Review
Source: Front Microbiol. 2021 Nov 10;12:737925. doi: 10.3389/fmicb.2021.737925 (PMC8634882; doi:10.3389/fmicb.2021.737925)
Supplement: Supplementary file 11 [file Image_11.pdf]

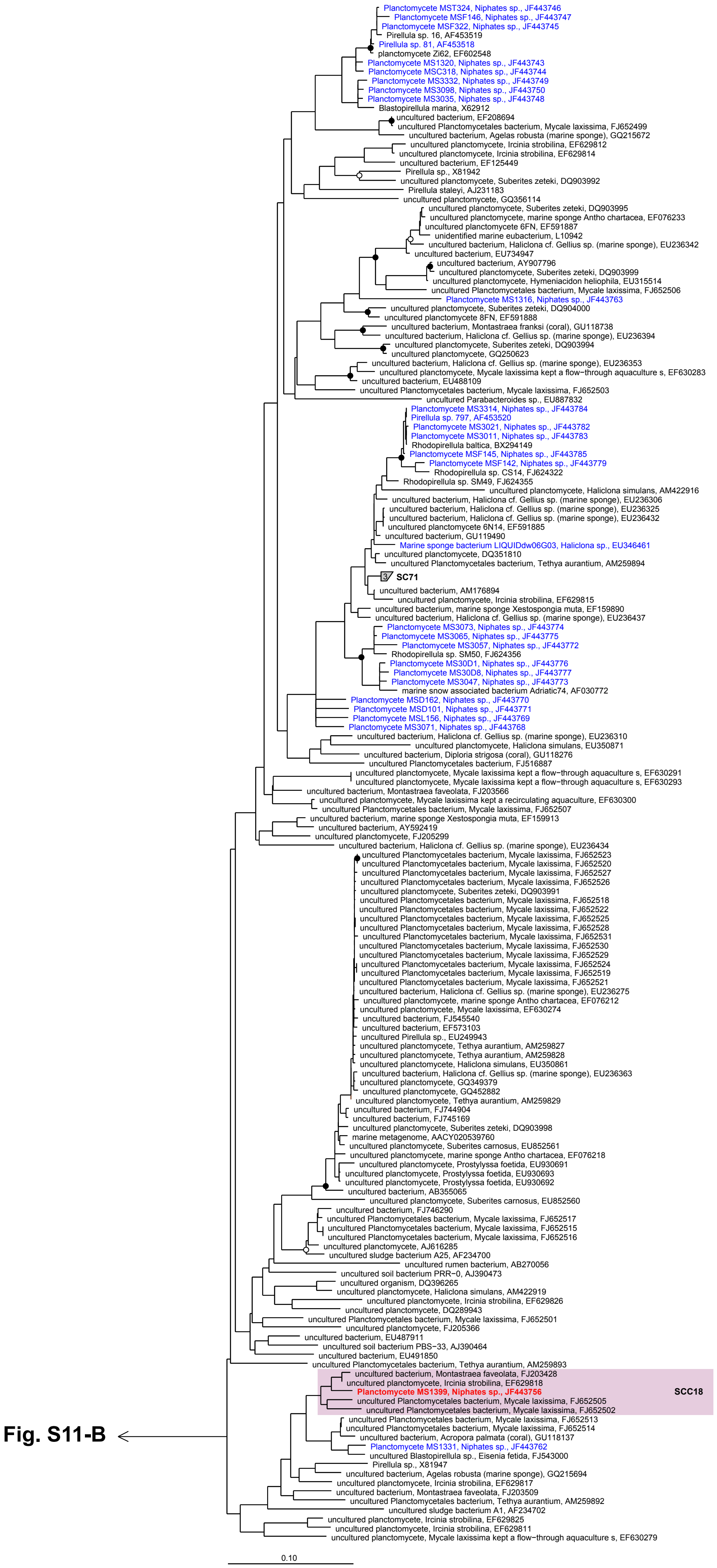

Figure S11-A. 16S rRNA gene-based phylogeny of sponge-associated Caldithrix/Deferribacteres, Chlamydiae, Halanaerobiales, Lentisphaerae, NPL-UP2, OP3, Planctomycetes, "Poribacteria", Verrucomicrobia organisms and SAUL (sponge-associated unidentified lineage). These taxa are affiliated with the Planctomycetes-Verrucomicrobia-Chlamydiae (PVC) superphylum. Details are as provided for Figure S1

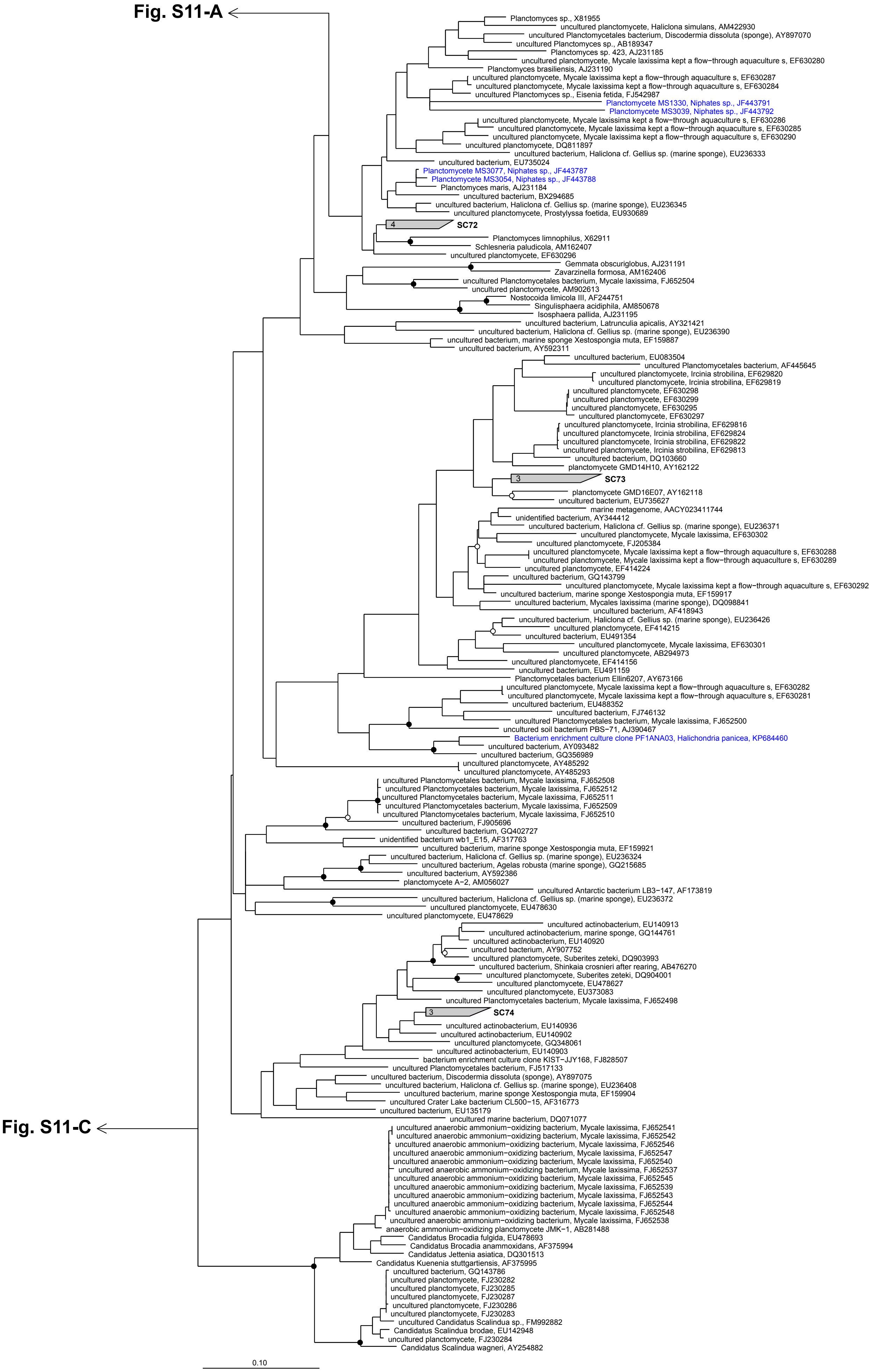

Figure S11-B. 16S rRNA gene-based phylogeny of sponge-associated Caldithrix/Deferribacteres, Chlamydiae, Halanaerobiales, Lentisphaerae, NPL-UP2, OP3, Planctomycetes, "Poribacteria", Verrucomicrobia organisms and SAUL (sponge-associated unidentified lineage). These taxa are affiliated with the Planctomycetes-Verrucomicrobia-Chlamydiae (PVC) superphylum. Details are as provided for Figure S1

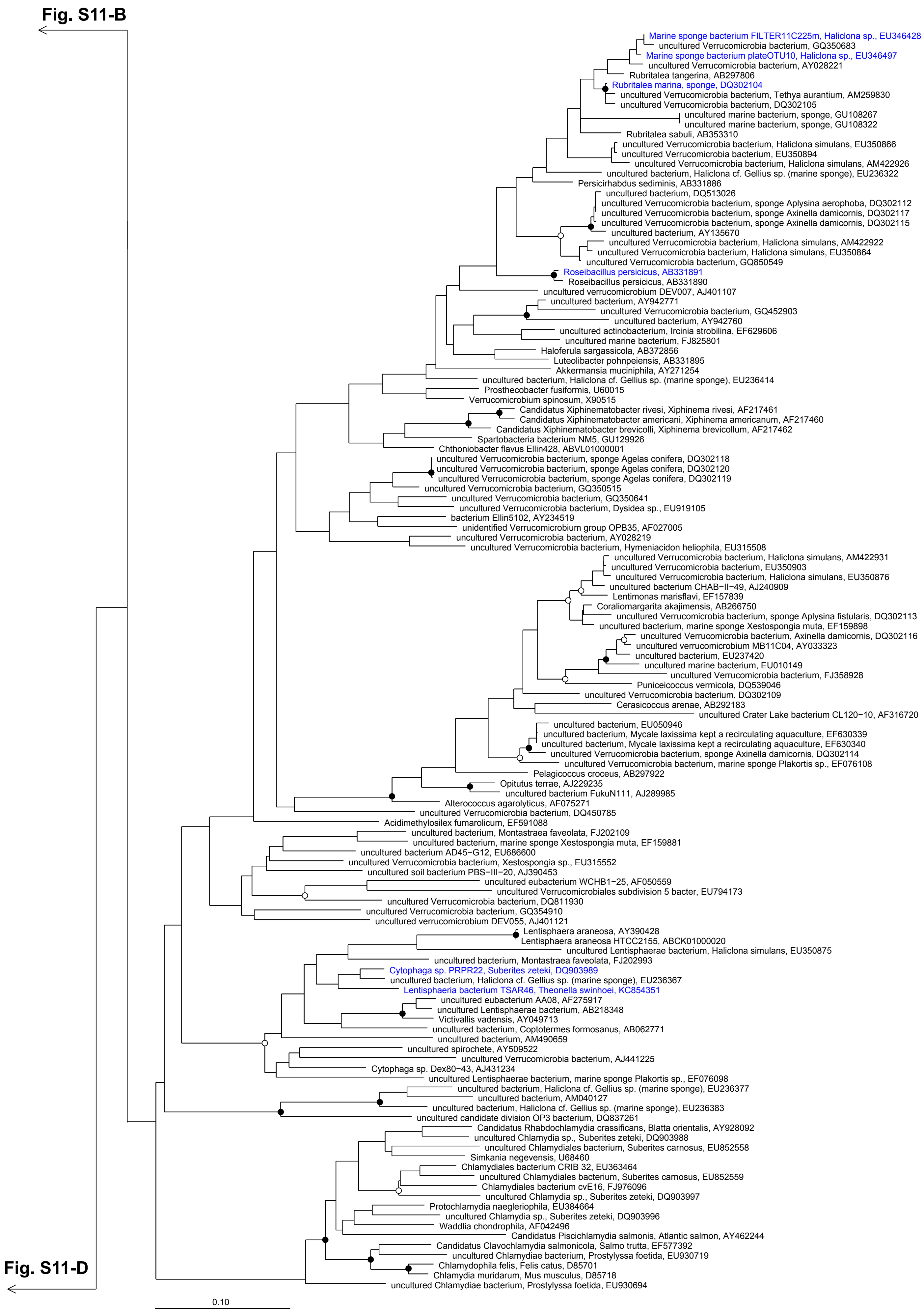

Figure S11-C. 16S rRNA gene-based phylogeny of sponge-associated Caldithrix/Deferribacteres, Chlamydiae, Halanaerobiales, Lentisphaerae, NPL-UP2, OP3, Planctomycetes, "Poribacteria", Verrucomicrobia organisms and SAUL (sponge-associated unidentified lineage). These taxa are affiliated with the Planctomycetes-Verrucomicrobia-Chlamydiae (PVC) superphylum. Details are as provided for Figure S1

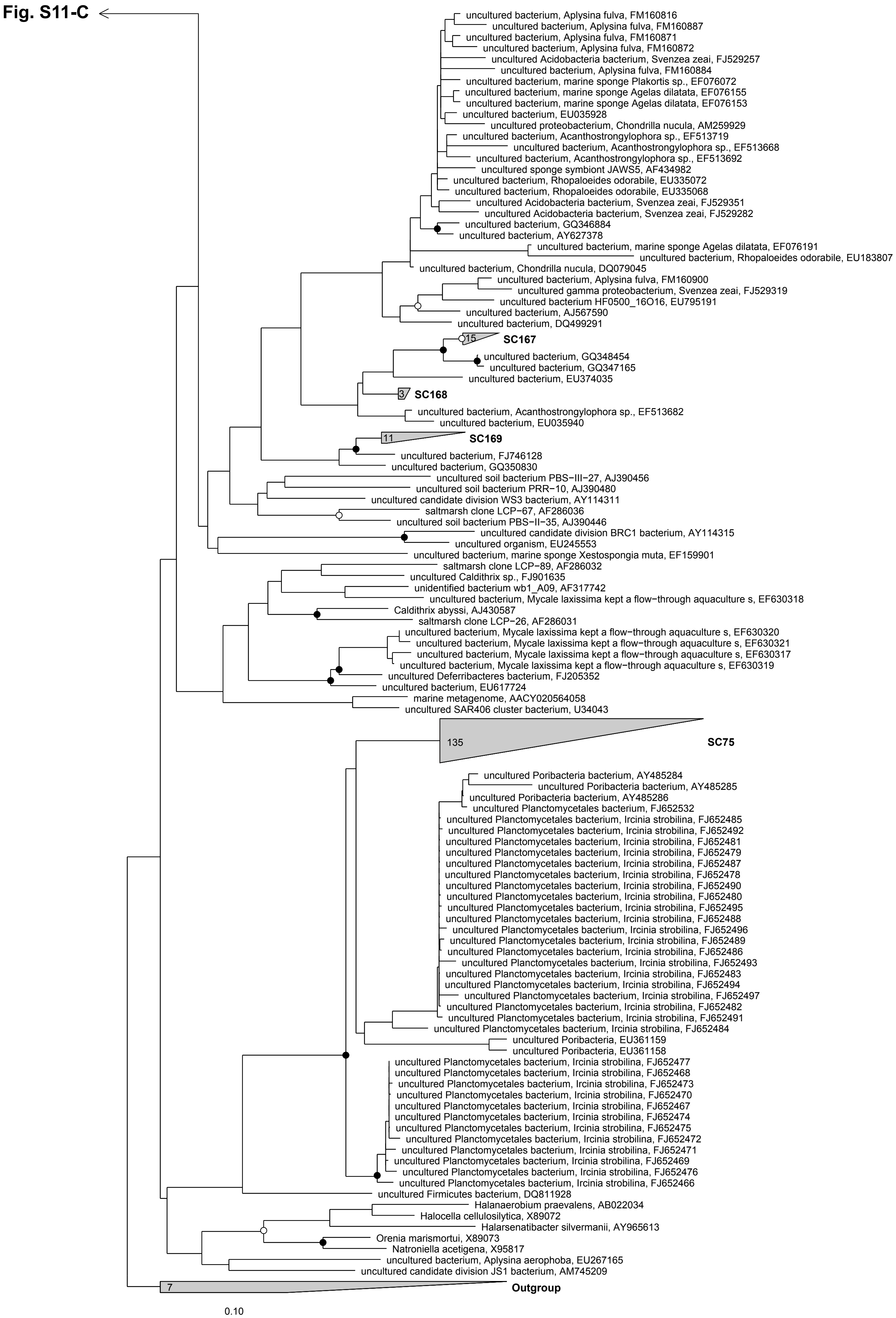

Figure S11-D. 16S rRNA gene-based phylogeny of sponge-associated Caldithrix/Deferribacteres, Chlamydiae, Halanaerobiales, Lentisphaerae, NPL-UP2, OP3, Planctomycetes, "Poribacteria", Verrucomicrobia organisms and SAUL (sponge-associated unidentified lineage). These taxa are affiliated with the Planctomycetes-Verrucomicrobia-Chlamydiae (PVC) superphylum. Details are as provided for Figure S1
