# Supplementary material for: Bacteria Cultivated From Sponges and Bacteria Not Yet Cultivated From Sponges—A Review
Source: Front Microbiol. 2021 Nov 10;12:737925. doi: 10.3389/fmicb.2021.737925 (PMC8634882; doi:10.3389/fmicb.2021.737925)
Supplement: Supplementary file 12 [file Data_Sheet_1.docx]

**Supplementary 1. MATERIALS AND METHODS**

**Data collection**

In this review, we collected 4915 16S ribosomal RNA (rRNA) gene sequences of cultivable bacteria associated with sponges from the NCBI database via published papers. Firstly, published papers reporting cultivable bacteria from sponges were retrieved from literature databases, including PubMed, Scopus, Web of Science, Google Scholar, using a query containing keywords related to cultivable bacteria from sponges: “(sponge OR porifera) AND (16S rRNA OR bacteria) AND (cultivation OR cultivable OR culture)” (updated to 2017). From the papers, accession numbers of cultivable bacteria from sponges were collected and used to retrieve the 16S rRNA gene sequences from the NCBI database. Corresponding data concerning culture conditions and culture media was also collected from published papers and/or from authors of papers. The metadata of sponge-associated culture bacteria can be accessed at the FigShare online Repository (<https://figshare.com/s/9daf8aa8261540110fbb>). For taxonomy, all 16S rRNA gene sequences of cultivable bacteria were re-classified based on the Silva database (v128) using the script *classify.seqs* in the Mothur pipeline with default parameters (Schloss et al., 2009).

**Heat map and Venn diagram**

In order to investigate the influence of carbon content (amount) in cultivation media on the composition of isolated sponge-associated bacteria, the amount of carbon in cultivation media was roughly estimated based on the amount of substrates containing carbon in culture media. For media using only seawater and agar for cultivation, we assumed that the amount of carbon in the media was zero. A heatmap of the 50 most abundant genera based on the amount of carbon and culture conditions was plotted using JColorGrid v.1.860 (Joachimiak et al., 2006). Venn diagrams of sponge-associated bacteria recovered under different cultivation conditions were created at the genus level using the online tool (<http://bioinformatics.psb.ugent.be/webtools/Venn/>).

**Identification of additional sequences as part of known sponge-specific and sponge coral-specific clusters**

The 16S rRNA gene sequences of cultured bacteria in our dataset and the 16S rRNA gene sequences from (Simister et al., 2012) were grouped per phylum. For each phylum, the sequences were aligned using the online Silva aligner (<https://www.arb-silva.de/aligner/>) with the global alignment mode (Pruesse et al., 2012). The aligned 16S rRNA gene sequences of cultured bacteria were added to available phylogenetic trees constructed by [Simister *et al.* (2012)](#_ENREF_1) using RAxML version 8.2 (Stamatakis, 2014). The additional sequences were identified as part of known SC/SCC clusters if their positions in the tree were inside known SC/SCCs.

**Sponge-enriched cultured bacteria**

In brief, the 16S rRNA gene sequences of cultured bacteria from our dataset (n = 4915) were subjected to a BLAST search (Altschul et al., 1990) against a curated sponge microbiome database, containing 64,424 high quality deblurred subOTU sequences that were extracted from the sponge Earth Microbiome Project (EMP) database (<https://github.com/amnona/SpongeEMP>). The curated spongeEMP BLAST database and additional information describing the database creation can be accessed here: <https://github.com/marinemoleco/spongeEMP_BLASTdb>. The sponge microbiome project subOTU sequences with 100% similarity to the 4915 bacterial isolate sequences were uploaded to the spongeEMP online server ([www.spongeemp.com](http://www.spongeemp.com)) in order to identify isolates that are significantly enriched in sponge specimens (for the category “host-associated” in the field env_package) using ranksum_p (relative frequency–based ranksum test) or binomial_p (presence/absence binomial test) as described by (Moitinho-Silva et al., 2017).

**Supplementary references**

Altschul SF, Gish W, Miller W, Myers EW, and Lipman DJ (1990) Basic local alignment search tool. *Journal of Molecular Biology* **215**: 403-410.

Joachimiak MP1, Weisman JL, May BCh (2006) JColorGrid: software for the visualization of biological measurements. *BMC Bioinformatics* **7**:225.

Moitinho-Silva L, Nielsen S, Amir A*, et al.* (2017) The sponge microbiome project. *GigaScience* **6**: 1-7.

Pruesse E, Peplies J, Glöckner FO (2012) SINA: Accurate high-throughput multiple sequence alignment of ribosomal RNA genes. *Bioinformatics* **28**: 1823-1829.

Schloss PD, Westcott SL, Ryabin T*, et al.* (2009) Introducing mothur: Open-Source, Platform-Independent, Community-Supported Software for Describing and Comparing Microbial Communities. *Applied and Environmental Microbiology* **75**: 7537-7541.

Stamatakis A (2014). RAxML Version 8: A tool for Phylogenetic Analysis and Post-Analysis of Large Phylogenies. *Bioinformatics* **30**: 1312-1313.

Simister RL, Deines P, Botté ES, Webster NS & Taylor MW (2012) Sponge-specific clusters revisited: a comprehensive phylogeny of sponge-associated microorganisms. *Environmental Microbiology* **14**: 517-524.
