# Supplementary material for: Bacteria Cultivated From Sponges and Bacteria Not Yet Cultivated From Sponges—A Review
Source: Front Microbiol. 2021 Nov 10;12:737925. doi: 10.3389/fmicb.2021.737925 (PMC8634882; doi:10.3389/fmicb.2021.737925)
Supplement: Supplementary file 13 [file Table_1.docx]

Table S1. Sponge-associated cultivable bacteria belonging to SC(C) clusters

| **Accesion number** | **SC(C)s** | **Phylum/ class** | **Genus** |
| --- | --- | --- | --- |
| EF198904 | SC11 | *Actinobacteria* | *Nocardiopsis* |
| EF198903 | SC11 | *Actinobacteria* | *Nocardiopsis* |
| EF198900 | SC11 | *Actinobacteria* | *Nocardiopsis* |
| EU873251 | SC11 | *Actinobacteria* | *Nocardiopsis* |
| EU554278 | SC11 | *Actinobacteria* | *Nocardiopsis* |
| EU554275 | SC11 | *Actinobacteria* | *Nocardiopsis* |
| FJ596490 | SC13 | *Actinobacteria* | *Brevibacterium* |
| FJ596452 | SC13 | *Actinobacteria* | - |
| FJ596451 | SC13 | *Actinobacteria* | - |
| FJ596420 | SC13 | *Actinobacteria* | *Brevibacterium* |
| FJ596548 | SC14 | *Actinobacteria* | *Brevibacterium* |
| FJ596537 | SC14 | *Actinobacteria* | *Brevibacterium* |
| GQ153943 | SC14 | *Actinobacteria* | *Brevibacterium* |
| EU346604 | SC26 | *Bacteroidetes* | - |
| EU346568 | SC26 | *Bacteroidetes* | *Gaetbulibacter* |
| EU346429 | SC26 | *Bacteroidetes* | *Gaetbulibacter* |
| AM990722 | SC27 | *Bacteroidetes* | - |
| KF282348 | SC27 | *Bacteroidetes* | - |
| FM180520 | SC28 | *Bacteroidetes* | *Aquimarina* |
| FJ348470 | SC28 | *Bacteroidetes* | *Aquimarina* |
| KF282352 | SC28 | *Bacteroidetes* | *Aquimarina* |
| FJ392545 | SC55 | *Cyanobacteria* | *Oscillatoria* |
| EF537057 | SC55 | *Cyanobacteria* | *Oscillatoria* |
| EF537058 | SC55 | *Cyanobacteria* | *Oscillatoria* |
| EF537059 | SC55 | *Cyanobacteria* | *Oscillatoria* |
| EF537060 | SC55 | *Cyanobacteria* | *Oscillatoria* |
| EF537061 | SC55 | *Cyanobacteria* | *Oscillatoria* |
| EF537062 | SC55 | *Cyanobacteria* | *Oscillatoria* |
| EF537054 | SC56 | *Cyanobacteria* | *Oscillatoria* |
| EF537055 | SC56 | *Cyanobacteria* | *Oscillatoria* |
| EF537056 | SC56 | *Cyanobacteria* | *Oscillatoria* |
| FJ596448 | SC58 | *Firmicutes* | *Bacillus* |
| FJ596439 | SC58 | *Firmicutes* | *Bacillus* |
| FJ596384 | SC58 | *Firmicutes* | *Bacillus* |
| KJ372507 | SC58 | *Firmicutes* | *Bacillus* |
| EU199239 | SC59 | *Firmicutes* | - |
| DQ274117 | SC59 | *Firmicutes* | *Bacillus* |
| DQ274116 | SC59 | *Firmicutes* | *Falsibacillus* |
| EU862089 | SC60 | *Firmicutes* | *Bacillus* |
| FJ596414 | SC60 | *Firmicutes* | *Bacillus* |
| FJ596413 | SC60 | *Firmicutes* | *Falsibacillus* |
| JX575224 | SC60 | *Firmicutes* | *Bacillus* |
| DQ888874 | SC60 | *Firmicutes* | *Bacillus* |
| DQ888882 | SC60 | *Firmicutes* | *Bacillus* |
| EU199230 | SC61 | *Firmicutes* | - |
| FJ596505 | SC61 | *Firmicutes* | - |
| FJ596485 | SC61 | *Firmicutes* | - |
| DQ903972 | SC61 | *Firmicutes* | *Bacillus* |
| KP684471 | SC62 | *Firmicutes* | - |
| DQ227660 | SC78 | *Alphaproteobacteria* | - |
| EF040555 | SC78 | *Alphaproteobacteria* | *Ruegeria* |
| EF040554 | SC78 | *Alphaproteobacteria* | *Ruegeria* |
| EF040543 | SC78 | *Alphaproteobacteria* | *Ruegeria* |
| EU346642 | SC84 | *Alphaproteobacteria* | *Pseudovibrio* |
| EU346493 | SC84 | *Alphaproteobacteria* | *Pseudovibrio* |
| FJ596464 | SC84 | *Alphaproteobacteria* | *Pseudovibrio* |
| FJ596368 | SC84 | *Alphaproteobacteria* | *Pseudovibrio* |
| HQ647081 | SC84 | *Alphaproteobacteria* | *Pseudovibrio* |
| HQ647070 | SC84 | *Alphaproteobacteria* | *Pseudovibrio* |
| HQ647064 | SC84 | *Alphaproteobacteria* | *Pseudovibrio* |
| HQ647057 | SC84 | *Alphaproteobacteria* | *Pseudovibrio* |
| HQ647053 | SC84 | *Alphaproteobacteria* | *Pseudovibrio* |
| HQ647045 | SC84 | *Alphaproteobacteria* | *Pseudovibrio* |
| HQ647039 | SC84 | *Alphaproteobacteria* | *Pseudovibrio* |
| HQ647038 | SC84 | *Alphaproteobacteria* | *Pseudovibrio* |
| HQ647036 | SC84 | *Alphaproteobacteria* | *Pseudovibrio* |
| HQ647031 | SC84 | *Alphaproteobacteria* | *Pseudovibrio* |
| EU346547 | SC84 | *Alphaproteobacteria* | *Pseudovibrio* |
| EU346538 | SC84 | *Alphaproteobacteria* | *Pseudovibrio* |
| KP684314 | SC84 | *Alphaproteobacteria* | *Pseudovibrio* |
| KP319339 | SC84 | *Alphaproteobacteria* | *Pseudovibrio* |
| KP319303 | SC84 | *Alphaproteobacteria* | *Pseudovibrio* |
| LN878392 | SC84 | *Alphaproteobacteria* | *Pseudovibrio* |
| LN878393 | SC84 | *Alphaproteobacteria* | *Pseudovibrio* |
| LN878600 | SC84 | *Alphaproteobacteria* | *Pseudovibrio* |
| EU346554 | SC85 | *Alphaproteobacteria* | *Pseudovibrio* |
| EU346546 | SC85 | *Alphaproteobacteria* | *Pseudovibrio* |
| EU346483 | SC85 | *Alphaproteobacteria* | *Pseudovibrio* |
| DQ888857 | SC85 | *Alphaproteobacteria* | *Pseudovibrio* |
| DQ888843 | SC85 | *Alphaproteobacteria* | *Pseudovibrio* |
| DQ888838 | SC85 | *Alphaproteobacteria* | *Pseudovibrio* |
| DQ888830 | SC85 | *Alphaproteobacteria* | *Pseudovibrio* |
| DQ888829 | SC85 | *Alphaproteobacteria* | *Pseudovibrio* |
| HQ647088 | SC85 | *Alphaproteobacteria* | *Pseudovibrio* |
| HQ647083 | SC85 | *Alphaproteobacteria* | *Pseudovibrio* |
| HQ647074 | SC85 | *Alphaproteobacteria* | *Pseudovibrio* |
| HQ647073 | SC85 | *Alphaproteobacteria* | *Pseudovibrio* |
| HQ647072 | SC85 | *Alphaproteobacteria* | *Pseudovibrio* |
| HQ647069 | SC85 | *Alphaproteobacteria* | *Pseudovibrio* |
| HQ647054 | SC85 | *Alphaproteobacteria* | *Pseudovibrio* |
| HQ647051 | SC85 | *Alphaproteobacteria* | *Pseudovibrio* |
| JX575241 | SC85 | *Alphaproteobacteria* | *Pseudovibrio* |
| EU346585 | SC85 | *Alphaproteobacteria* | *Pseudovibrio* |
| EU346577 | SC85 | *Alphaproteobacteria* | *Pseudovibrio* |
| EU346543 | SC85 | *Alphaproteobacteria* | *Pseudovibrio* |
| KF582876 | SC85 | *Alphaproteobacteria* | *Pseudovibrio* |
| KF582850 | SC85 | *Alphaproteobacteria* | *Pseudovibrio* |
| HE818274 | SC85 | *Alphaproteobacteria* | *Pseudovibrio* |
| HE818278 | SC85 | *Alphaproteobacteria* | *Pseudovibrio* |
| KP319326 | SC85 | *Alphaproteobacteria* | *Pseudovibrio* |
| KP319316 | SC85 | *Alphaproteobacteria* | *Pseudovibrio* |
| KP319314 | SC85 | *Alphaproteobacteria* | *Pseudovibrio* |
| KP319311 | SC85 | *Alphaproteobacteria* | *Pseudovibrio* |
| KP319307 | SC85 | *Alphaproteobacteria* | *Pseudovibrio* |
| KP319306 | SC85 | *Alphaproteobacteria* | *Pseudovibrio* |
| KP319299 | SC85 | *Alphaproteobacteria* | *Pseudovibrio* |
| LN878487 | SC85 | *Alphaproteobacteria* | *Pseudovibrio* |
| AY372908 | SC86 | *Alphaproteobacteria* | *Pseudovibrio* |
| AY372915 | SC86 | *Alphaproteobacteria* | *Pseudovibrio* |
| EU346594 | SC93 | *Alphaproteobacteria* | *Erythrobacter* |
| AY367756 | SC93 | *Alphaproteobacteria* | *Erythrobacter* |
| AJ849370 | SC93 | *Alphaproteobacteria* | *Erythrobacter* |
| FM180516 | SC94 | *Alphaproteobacteria* | - |
| AY371429 | SC108 | *Alphaproteobacteria* | *Tistlia* |
| DQ869302 | SC108 | *Alphaproteobacteria* | *Tistlia* |
| DQ167235 | SC108 | *Alphaproteobacteria* | *Tistlia* |
| EU346489 | SC112 | *Betaproteobacteria* | *Candidatus_Branchiomonas* |
| EU346541 | SC112 | *Betaproteobacteria* | *Candidatus_Branchiomonas* |
| EU346540 | SC112 | *Betaproteobacteria* | *Candidatus_Branchiomonas* |
| EU346539 | SC112 | *Betaproteobacteria* | *Candidatus_Branchiomonas* |
| FJ596536 | SC126 | *Gammaproteobacteria* | - |
| FJ596528 | SC126 | *Gammaproteobacteria* | *Pseudoalteromonas* |
| FJ596371 | SC126 | *Gammaproteobacteria* | - |
| FJ596367 | SC126 | *Gammaproteobacteria* | *Pseudoalteromonas* |
| FJ596342 | SC126 | *Gammaproteobacteria* | *Pseudoalteromonas* |
| AY241442 | SC127 | *Gammaproteobacteria* | *Vibrio* |
| AY241441 | SC127 | *Gammaproteobacteria* | *Vibrio* |
| AY241439 | SC127 | *Gammaproteobacteria* | *Vibrio* |
| AY241438 | SC127 | *Gammaproteobacteria* | *Vibrio* |
| AY241435 | SC127 | *Gammaproteobacteria* | *Vibrio* |
| FJ596487 | SC127 | *Gammaproteobacteria* | *Vibrio* |
| FJ596459 | SC127 | *Gammaproteobacteria* | *Vibrio* |
| FJ596424 | SC127 | *Gammaproteobacteria* | *Vibrio* |
| FJ596343 | SC127 | *Gammaproteobacteria* | *Vibrio* |
| EF040571 | SC128 | *Gammaproteobacteria* | *Escherichia-Shigella* |
| EF040564 | SC128 | *Gammaproteobacteria* | *Escherichia-Shigella* |
| EF040551 | SC128 | *Gammaproteobacteria* | - |
| EF040537 | SC128 | *Gammaproteobacteria* | *Escherichia-Shigella* |
| EF040567 | SC128 | *Gammaproteobacteria* | - |
| EF114194 | SC130 | *Gammaproteobacteria* | - |
| AY368567 | SC133 | *Gammaproteobacteria* | *Rheinheimera* |
| EF450321 | SC133 | *Gammaproteobacteria* | *Rheinheimera* |
| EF450318 | SC133 | *Gammaproteobacteria* | *Rheinheimera* |
| EU346557 | SC137 | *Gammaproteobacteria* | *BD1-7_clade* |
| EU346468 | SC138 | *Gammaproteobacteria* | *BD1-7_clade* |
| EU346467 | SC138 | *Gammaproteobacteria* | *BD1-7_clade* |
| AY371439 | SC138 | *Gammaproteobacteria* | *BD1-7_clade* |
| AY371442 | SC139 | *Gammaproteobacteria* | *Endozoicomonas* |
| AY371441 | SC139 | *Gammaproteobacteria* | *Endozoicomonas* |
| EF620871 | SC139 | *Gammaproteobacteria* | *Endozoicomonas* |
| AF489288 | SC141 | *Gammaproteobacteria* | *Pseudomonas* |
| AF489289 | SC141 | *Gammaproteobacteria* | *Pseudomonas* |
| FJ215629 | SC141 | *Gammaproteobacteria* | *Pseudomonas* |
| FJ215627 | SC141 | *Gammaproteobacteria* | *Pseudomonas* |
| JN128264 | SC141 | *Gammaproteobacteria* | *Pseudomonas* |
| JN128255 | SC141 | *Gammaproteobacteria* | *Pseudomonas* |
| KP684435 | SC141 | *Gammaproteobacteria* | *Pseudomonas* |
| EF114176 | SC149 | *Gammaproteobacteria* | *Stenotrophomonas* |
| JN615424 | SC149 | *Gammaproteobacteria* | *Stenotrophomonas* |
| DQ994722 | SCC6 | *Acidobacteria* | - |
| EF629874 | SCC7 | *Bacteroidetes* | *Fabibacter* |
| EF629869 | SCC7 | *Bacteroidetes* | *Fabibacter* |
| EF629864 | SCC7 | *Bacteroidetes* | *Fabibacter* |
| EF629858 | SCC7 | *Bacteroidetes* | *Fabibacter* |
| EF629855 | SCC7 | *Bacteroidetes* | *Fabibacter* |
| EF629831 | SCC7 | *Bacteroidetes* | *Fabibacter* |
| JF443756 | SCC18 | *Planctomycetes* | *Pir4_lineage* |
| EU346463 | SCC28 | *Alphaproteobacteria* | - |
| AY369986 | SCC31 | *Gammaproteobacteria* | *Shewanella* |
| AY371432 | SCC31 | *Gammaproteobacteria* | - |
| AY371431 | SCC31 | *Gammaproteobacteria* | *Paraferrimonas* |
| AY948366 | SCC31 | *Gammaproteobacteria* | *Shewanella* |
| DQ167234 | SCC31 | *Gammaproteobacteria* | *Shewanella* |
| DQ180743 | SCC31 | *Gammaproteobacteria* | *Shewanella* |
| JX575276 | SCC31 | *Gammaproteobacteria* | *Shewanella* |
| KJ372448 | SCC31 | *Gammaproteobacteria* | *Shewanella* |
| KJ372447 | SCC31 | *Gammaproteobacteria* | *Shewanella* |
| KJ372446 | SCC31 | *Gammaproteobacteria* | *Shewanella* |
| KJ372445 | SCC31 | *Gammaproteobacteria* | *Shewanella* |
| KJ372444 | SCC31 | *Gammaproteobacteria* | *Shewanella* |
| KJ372443 | SCC31 | *Gammaproteobacteria* | *Shewanella* |
| KJ372442 | SCC31 | *Gammaproteobacteria* | *Shewanella* |
| KJ372441 | SCC31 | *Gammaproteobacteria* | *Shewanella* |
| KJ372440 | SCC31 | *Gammaproteobacteria* | - |
| KJ372439 | SCC31 | *Gammaproteobacteria* | *Shewanella* |
| KJ372438 | SCC31 | *Gammaproteobacteria* | *Shewanella* |
| KJ372437 | SCC31 | *Gammaproteobacteria* | *Shewanella* |
| KJ372436 | SCC31 | *Gammaproteobacteria* | *Shewanella* |
| KJ372435 | SCC31 | *Gammaproteobacteria* | *Shewanella* |
| KJ372434 | SCC31 | *Gammaproteobacteria* | *Shewanella* |
| KJ372433 | SCC31 | *Gammaproteobacteria* | *Shewanella* |
| HE818163 | SCC31 | *Gammaproteobacteria* | *Shewanella* |
| HE818167 | SCC31 | *Gammaproteobacteria* | *Shewanella* |
| HE818174 | SCC31 | *Gammaproteobacteria* | *Shewanella* |
| HE818216 | SCC31 | *Gammaproteobacteria* | *Shewanella* |
| KP684413 | SCC31 | *Gammaproteobacteria* | *Shewanella* |
| KP684406 | SCC31 | *Gammaproteobacteria* | *Shewanella* |
| KP684297 | SCC31 | *Gammaproteobacteria* | *Shewanella* |
| EF629566 | SCC31, SC132 | *Gammaproteobacteria* | *Shewanella* |
| EF629564 | SCC31, SC132 | *Gammaproteobacteria* | *Shewanella* |
| EF629559 | SCC31, SC132 | *Gammaproteobacteria* | *Shewanella* |
