# Supplementary material for: Bacteria Cultivated From Sponges and Bacteria Not Yet Cultivated From Sponges—A Review
Source: Front Microbiol. 2021 Nov 10;12:737925. doi: 10.3389/fmicb.2021.737925 (PMC8634882; doi:10.3389/fmicb.2021.737925)
Supplement: Supplementary file 14 [file Table_2.docx]

Table S2. Cultivable bacteria enriched in sponge

| **Accession number** | **OTU** | **p_value** | **Category** | **Phylum/ class** | **Genus** |
| --- | --- | --- | --- | --- | --- |
| KC854357 | OTU93 | binomial_p=0.071110, ranksum_p=0.000005 | Sponge-enriched | Gammaproteobacteria | Endozoicomonas |
| AB012864 | OTU1146 | binomial_p=0.244977, ranksum_p=0.025374 | Sponge-enriched | Alphaproteobacteria | Pseudovibrio |
| AF295099 | OTU1146 | binomial_p=0.244977, ranksum_p=0.025374 | Sponge-enriched | Alphaproteobacteria | Pseudovibrio |
| AY364592 | OTU1146 | binomial_p=0.244977, ranksum_p=0.025374 | Sponge-enriched | Alphaproteobacteria | Pseudovibrio |
| AY372904 | OTU1146 | binomial_p=0.244977, ranksum_p=0.025374 | Sponge-enriched | Alphaproteobacteria | Pseudovibrio |
| AY372911 | OTU1146 | binomial_p=0.244977, ranksum_p=0.025374 | Sponge-enriched | Alphaproteobacteria | Pseudovibrio |
| DQ097237 | OTU1146 | binomial_p=0.244977, ranksum_p=0.025374 | Sponge-enriched | Alphaproteobacteria | Pseudovibrio |
| DQ097238 | OTU1146 | binomial_p=0.244977, ranksum_p=0.025374 | Sponge-enriched | Alphaproteobacteria | Pseudovibrio |
| DQ097239 | OTU1146 | binomial_p=0.244977, ranksum_p=0.025374 | Sponge-enriched | Alphaproteobacteria | Pseudovibrio |
| DQ097240 | OTU1146 | binomial_p=0.244977, ranksum_p=0.025374 | Sponge-enriched | Alphaproteobacteria | Pseudovibrio |
| DQ097241 | OTU1146 | binomial_p=0.244977, ranksum_p=0.025374 | Sponge-enriched | Alphaproteobacteria | Pseudovibrio |
| DQ097242 | OTU1146 | binomial_p=0.244977, ranksum_p=0.025374 | Sponge-enriched | Alphaproteobacteria | Pseudovibrio |
| DQ097243 | OTU1146 | binomial_p=0.244977, ranksum_p=0.025374 | Sponge-enriched | Alphaproteobacteria | Pseudovibrio |
| DQ097244 | OTU1146 | binomial_p=0.244977, ranksum_p=0.025374 | Sponge-enriched | Alphaproteobacteria | Pseudovibrio |
| DQ097245 | OTU1146 | binomial_p=0.244977, ranksum_p=0.025374 | Sponge-enriched | Alphaproteobacteria | Pseudovibrio |
| DQ097246 | OTU1146 | binomial_p=0.244977, ranksum_p=0.025374 | Sponge-enriched | Alphaproteobacteria | Pseudovibrio |
| DQ097247 | OTU1146 | binomial_p=0.244977, ranksum_p=0.025374 | Sponge-enriched | Alphaproteobacteria | Pseudovibrio |
| DQ097248 | OTU1146 | binomial_p=0.244977, ranksum_p=0.025374 | Sponge-enriched | Alphaproteobacteria | Pseudovibrio |
| DQ097249 | OTU1146 | binomial_p=0.244977, ranksum_p=0.025374 | Sponge-enriched | Alphaproteobacteria | Pseudovibrio |
| DQ097250 | OTU1146 | binomial_p=0.244977, ranksum_p=0.025374 | Sponge-enriched | Alphaproteobacteria | Pseudovibrio |
| DQ097251 | OTU1146 | binomial_p=0.244977, ranksum_p=0.025374 | Sponge-enriched | Alphaproteobacteria | Pseudovibrio |
| DQ097252 | OTU1146 | binomial_p=0.244977, ranksum_p=0.025374 | Sponge-enriched | Alphaproteobacteria | Pseudovibrio |
| DQ097253 | OTU1146 | binomial_p=0.244977, ranksum_p=0.025374 | Sponge-enriched | Alphaproteobacteria | Pseudovibrio |
| DQ097254 | OTU1146 | binomial_p=0.244977, ranksum_p=0.025374 | Sponge-enriched | Alphaproteobacteria | Pseudovibrio |
| DQ097256 | OTU1146 | binomial_p=0.244977, ranksum_p=0.025374 | Sponge-enriched | Alphaproteobacteria | Pseudovibrio |
| DQ097257 | OTU1146 | binomial_p=0.244977, ranksum_p=0.025374 | Sponge-enriched | Alphaproteobacteria | Pseudovibrio |
| DQ097259 | OTU1146 | binomial_p=0.244977, ranksum_p=0.025374 | Sponge-enriched | Alphaproteobacteria | Pseudovibrio |
| DQ097260 | OTU1146 | binomial_p=0.244977, ranksum_p=0.025374 | Sponge-enriched | Alphaproteobacteria | Pseudovibrio |
| DQ097261 | OTU1146 | binomial_p=0.244977, ranksum_p=0.025374 | Sponge-enriched | Alphaproteobacteria | Pseudovibrio |
| DQ097262 | OTU1146 | binomial_p=0.244977, ranksum_p=0.025374 | Sponge-enriched | Alphaproteobacteria | Pseudovibrio |
| DQ097263 | OTU1146 | binomial_p=0.244977, ranksum_p=0.025374 | Sponge-enriched | Alphaproteobacteria | Pseudovibrio |
| DQ156536 | OTU1146 | binomial_p=0.244977, ranksum_p=0.025374 | Sponge-enriched | Alphaproteobacteria | Pseudovibrio |
| DQ227655 | OTU1146 | binomial_p=0.244977, ranksum_p=0.025374 | Sponge-enriched | Alphaproteobacteria | Pseudovibrio |
| DQ227656 | OTU1146 | binomial_p=0.244977, ranksum_p=0.025374 | Sponge-enriched | Alphaproteobacteria | Pseudovibrio |
| DQ227657 | OTU1146 | binomial_p=0.244977, ranksum_p=0.025374 | Sponge-enriched | Alphaproteobacteria | Pseudovibrio |
| DQ227658 | OTU1146 | binomial_p=0.244977, ranksum_p=0.025374 | Sponge-enriched | Alphaproteobacteria | Pseudovibrio |
| DQ399712 | OTU1146 | binomial_p=0.244977, ranksum_p=0.025374 | Sponge-enriched | Alphaproteobacteria | Pseudovibrio |
| DQ399713 | OTU1146 | binomial_p=0.244977, ranksum_p=0.025374 | Sponge-enriched | Alphaproteobacteria | Pseudovibrio |
| DQ399714 | OTU1146 | binomial_p=0.244977, ranksum_p=0.025374 | Sponge-enriched | Alphaproteobacteria | Pseudovibrio |
| DQ399715 | OTU1146 | binomial_p=0.244977, ranksum_p=0.025374 | Sponge-enriched | Alphaproteobacteria | Pseudovibrio |
| DQ399717 | OTU1146 | binomial_p=0.244977, ranksum_p=0.025374 | Sponge-enriched | Alphaproteobacteria | Pseudovibrio |
| DQ399719 | OTU1146 | binomial_p=0.244977, ranksum_p=0.025374 | Sponge-enriched | Alphaproteobacteria | Pseudovibrio |
| DQ399720 | OTU1146 | binomial_p=0.244977, ranksum_p=0.025374 | Sponge-enriched | Alphaproteobacteria | Pseudovibrio |
| DQ399721 | OTU1146 | binomial_p=0.244977, ranksum_p=0.025374 | Sponge-enriched | Alphaproteobacteria | Pseudovibrio |
| DQ399722 | OTU1146 | binomial_p=0.244977, ranksum_p=0.025374 | Sponge-enriched | Alphaproteobacteria | Pseudovibrio |
| DQ399723 | OTU1146 | binomial_p=0.244977, ranksum_p=0.025374 | Sponge-enriched | Alphaproteobacteria | Pseudovibrio |
| DQ399724 | OTU1146 | binomial_p=0.244977, ranksum_p=0.025374 | Sponge-enriched | Alphaproteobacteria | Pseudovibrio |
| DQ399725 | OTU1146 | binomial_p=0.244977, ranksum_p=0.025374 | Sponge-enriched | Alphaproteobacteria | Pseudovibrio |
| DQ399726 | OTU1146 | binomial_p=0.244977, ranksum_p=0.025374 | Sponge-enriched | Alphaproteobacteria | Pseudovibrio |
| DQ869304 | OTU1146 | binomial_p=0.244977, ranksum_p=0.025374 | Sponge-enriched | Alphaproteobacteria | Pseudovibrio |
| DQ874974 | OTU1146 | binomial_p=0.244977, ranksum_p=0.025374 | Sponge-enriched | Alphaproteobacteria | Pseudovibrio |
| EF040574 | OTU1146 | binomial_p=0.244977, ranksum_p=0.025374 | Sponge-enriched | Alphaproteobacteria | Pseudovibrio |
| EF040575 | OTU1146 | binomial_p=0.244977, ranksum_p=0.025374 | Sponge-enriched | Alphaproteobacteria | Pseudovibrio |
| EF040579 | OTU1146 | binomial_p=0.244977, ranksum_p=0.025374 | Sponge-enriched | Alphaproteobacteria | Pseudovibrio |
| EF414057 | OTU1146 | binomial_p=0.244977, ranksum_p=0.025374 | Sponge-enriched | Alphaproteobacteria | Pseudovibrio |
| EF414061 | OTU1146 | binomial_p=0.244977, ranksum_p=0.025374 | Sponge-enriched | Alphaproteobacteria | Pseudovibrio |
| EF513634 | OTU1146 | binomial_p=0.244977, ranksum_p=0.025374 | Sponge-enriched | Alphaproteobacteria | Pseudovibrio |
| EF513636 | OTU1146 | binomial_p=0.244977, ranksum_p=0.025374 | Sponge-enriched | Alphaproteobacteria | Pseudovibrio |
| EF620867 | OTU1146 | binomial_p=0.244977, ranksum_p=0.025374 | Sponge-enriched | Alphaproteobacteria | Pseudovibrio |
| EF629879 | OTU1146 | binomial_p=0.244977, ranksum_p=0.025374 | Sponge-enriched | Alphaproteobacteria | Pseudovibrio |
| FJ215561 | OTU1146 | binomial_p=0.244977, ranksum_p=0.025374 | Sponge-enriched | Alphaproteobacteria | Pseudovibrio |
| FJ215563 | OTU1146 | binomial_p=0.244977, ranksum_p=0.025374 | Sponge-enriched | Alphaproteobacteria | Pseudovibrio |
| FJ215564 | OTU1146 | binomial_p=0.244977, ranksum_p=0.025374 | Sponge-enriched | Alphaproteobacteria | Pseudovibrio |
| FJ215565 | OTU1146 | binomial_p=0.244977, ranksum_p=0.025374 | Sponge-enriched | Alphaproteobacteria | Pseudovibrio |
| FJ215567 | OTU1146 | binomial_p=0.244977, ranksum_p=0.025374 | Sponge-enriched | Alphaproteobacteria | Pseudovibrio |
| FJ215568 | OTU1146 | binomial_p=0.244977, ranksum_p=0.025374 | Sponge-enriched | Alphaproteobacteria | Pseudovibrio |
| FJ215569 | OTU1146 | binomial_p=0.244977, ranksum_p=0.025374 | Sponge-enriched | Alphaproteobacteria | Pseudovibrio |
| FJ215577 | OTU1146 | binomial_p=0.244977, ranksum_p=0.025374 | Sponge-enriched | Alphaproteobacteria | Pseudovibrio |
| FJ215578 | OTU1146 | binomial_p=0.244977, ranksum_p=0.025374 | Sponge-enriched | Alphaproteobacteria | Pseudovibrio |
| FJ215580 | OTU1146 | binomial_p=0.244977, ranksum_p=0.025374 | Sponge-enriched | Alphaproteobacteria | Pseudovibrio |
| FJ215583 | OTU1146 | binomial_p=0.244977, ranksum_p=0.025374 | Sponge-enriched | Alphaproteobacteria | Pseudovibrio |
| FJ215619 | OTU1146 | binomial_p=0.244977, ranksum_p=0.025374 | Sponge-enriched | Alphaproteobacteria | Pseudovibrio |
| FJ215620 | OTU1146 | binomial_p=0.244977, ranksum_p=0.025374 | Sponge-enriched | Alphaproteobacteria | Pseudovibrio |
| FM180515 | OTU1146 | binomial_p=0.244977, ranksum_p=0.025374 | Sponge-enriched | Alphaproteobacteria | Pseudovibrio |
| GQ169725 | OTU1146 | binomial_p=0.244977, ranksum_p=0.025374 | Sponge-enriched | Alphaproteobacteria | Pseudovibrio |
| JN128253 | OTU1146 | binomial_p=0.244977, ranksum_p=0.025374 | Sponge-enriched | Alphaproteobacteria | Pseudovibrio |
| JN615421 | OTU1146 | binomial_p=0.244977, ranksum_p=0.025374 | Sponge-enriched | Alphaproteobacteria | Pseudovibrio |
| JN615432 | OTU1146 | binomial_p=0.244977, ranksum_p=0.025374 | Sponge-enriched | Alphaproteobacteria | Pseudovibrio |
| JN615449 | OTU1146 | binomial_p=0.244977, ranksum_p=0.025374 | Sponge-enriched | Alphaproteobacteria | Pseudovibrio |
| KC854396 | OTU1146 | binomial_p=0.244977, ranksum_p=0.025374 | Sponge-enriched | Alphaproteobacteria | Pseudovibrio |
| KF282364 | OTU1146 | binomial_p=0.244977, ranksum_p=0.025374 | Sponge-enriched | Alphaproteobacteria | Pseudovibrio |
| KF282365 | OTU1146 | binomial_p=0.244977, ranksum_p=0.025374 | Sponge-enriched | Alphaproteobacteria | Pseudovibrio |
| KF282368 | OTU1146 | binomial_p=0.244977, ranksum_p=0.025374 | Sponge-enriched | Alphaproteobacteria | Pseudovibrio |
| KF282369 | OTU1146 | binomial_p=0.244977, ranksum_p=0.025374 | Sponge-enriched | Alphaproteobacteria | Pseudovibrio |
| KF282374 | OTU1146 | binomial_p=0.244977, ranksum_p=0.025374 | Sponge-enriched | Alphaproteobacteria | Pseudovibrio |
| KF282424 | OTU1146 | binomial_p=0.244977, ranksum_p=0.025374 | Sponge-enriched | Alphaproteobacteria | Pseudovibrio |
| KF282442 | OTU1146 | binomial_p=0.244977, ranksum_p=0.025374 | Sponge-enriched | Alphaproteobacteria | Pseudovibrio |
| KF282463 | OTU1146 | binomial_p=0.244977, ranksum_p=0.025374 | Sponge-enriched | Alphaproteobacteria | Pseudovibrio |
| KF282533 | OTU1146 | binomial_p=0.244977, ranksum_p=0.025374 | Sponge-enriched | Alphaproteobacteria | Pseudovibrio |
| KF282544 | OTU1146 | binomial_p=0.244977, ranksum_p=0.025374 | Sponge-enriched | Alphaproteobacteria | Pseudovibrio |
| KF282576 | OTU1146 | binomial_p=0.244977, ranksum_p=0.025374 | Sponge-enriched | Alphaproteobacteria | Pseudovibrio |
| KJ372497 | OTU1146 | binomial_p=0.244977, ranksum_p=0.025374 | Sponge-enriched | Alphaproteobacteria | Pseudovibrio |
| KJ372498 | OTU1146 | binomial_p=0.244977, ranksum_p=0.025374 | Sponge-enriched | Alphaproteobacteria | Pseudovibrio |
| KJ372499 | OTU1146 | binomial_p=0.244977, ranksum_p=0.025374 | Sponge-enriched | Alphaproteobacteria | Pseudovibrio |
| KJ573545 | OTU1146 | binomial_p=0.244977, ranksum_p=0.025374 | Sponge-enriched | Alphaproteobacteria | Pseudovibrio |
| AY368536 | OTU12079 | binomial_p=0.312694, ranksum_p=0.087930 | Sponge-enriched | Alphaproteobacteria | Paracocccus |
| AB695088 | OTU14946 | binomial_p=0.084680, ranksum_p=0.000019 | Sponge-enriched | Gammaproteobacteria | Endozoicomonas |
| AB695089 | OTU14946 | binomial_p=0.084680, ranksum_p=0.000019 | Sponge-enriched | Gammaproteobacteria | Endozoicomonas |
| AM990755 | OTU14946 | binomial_p=0.084680, ranksum_p=0.000019 | Sponge-enriched | Gammaproteobacteria | Endozoicomonas |
| AY370008 | OTU14946 | binomial_p=0.084680, ranksum_p=0.000019 | Sponge-enriched | Gammaproteobacteria | Endozoicomonas |
| KJ372449 | OTU14946 | binomial_p=0.084680, ranksum_p=0.000019 | Sponge-enriched | Gammaproteobacteria | Endozoicomonas |
| KJ372450 | OTU14946 | binomial_p=0.084680, ranksum_p=0.000019 | Sponge-enriched | Gammaproteobacteria | Endozoicomonas |
| KJ372451 | OTU14946 | binomial_p=0.084680, ranksum_p=0.000019 | Sponge-enriched | Gammaproteobacteria | Endozoicomonas |
| KJ372452 | OTU14946 | binomial_p=0.084680, ranksum_p=0.000019 | Sponge-enriched | Gammaproteobacteria | Endozoicomonas |
| KJ372453 | OTU14946 | binomial_p=0.084680, ranksum_p=0.000019 | Sponge-enriched | Gammaproteobacteria | Endozoicomonas |
| KJ372454 | OTU14946 | binomial_p=0.084680, ranksum_p=0.000019 | Sponge-enriched | Gammaproteobacteria | Endozoicomonas |
| KJ372455 | OTU14946 | binomial_p=0.084680, ranksum_p=0.000019 | Sponge-enriched | Gammaproteobacteria | Endozoicomonas |
| KJ372456 | OTU14946 | binomial_p=0.084680, ranksum_p=0.000019 | Sponge-enriched | Gammaproteobacteria | Endozoicomonas |
| KJ372457 | OTU14946 | binomial_p=0.084680, ranksum_p=0.000019 | Sponge-enriched | Gammaproteobacteria | Endozoicomonas |
| KJ372458 | OTU14946 | binomial_p=0.084680, ranksum_p=0.000019 | Sponge-enriched | Gammaproteobacteria | Endozoicomonas |
| KJ372459 | OTU14946 | binomial_p=0.084680, ranksum_p=0.000019 | Sponge-enriched | Gammaproteobacteria | Endozoicomonas |
| KJ372463 | OTU14946 | binomial_p=0.084680, ranksum_p=0.000019 | Sponge-enriched | Gammaproteobacteria | Endozoicomonas |
| KJ372464 | OTU14946 | binomial_p=0.084680, ranksum_p=0.000019 | Sponge-enriched | Gammaproteobacteria | Endozoicomonas |
| KJ372470 | OTU14946 | binomial_p=0.084680, ranksum_p=0.000019 | Sponge-enriched | Gammaproteobacteria | Endozoicomonas |
| KJ372472 | OTU14946 | binomial_p=0.084680, ranksum_p=0.000019 | Sponge-enriched | Gammaproteobacteria | Endozoicomonas |
| KJ372476 | OTU14946 | binomial_p=0.084680, ranksum_p=0.000019 | Sponge-enriched | Gammaproteobacteria | Endozoicomonas |
| KJ372477 | OTU14946 | binomial_p=0.084680, ranksum_p=0.000019 | Sponge-enriched | Gammaproteobacteria | Endozoicomonas |
| KJ372478 | OTU14946 | binomial_p=0.084680, ranksum_p=0.000019 | Sponge-enriched | Gammaproteobacteria | Endozoicomonas |
| KJ372479 | OTU14946 | binomial_p=0.084680, ranksum_p=0.000019 | Sponge-enriched | Gammaproteobacteria | Endozoicomonas |
| AY371429 | OTU1500 | binomial_p=0.169591, ranksum_p=0.002361 | Sponge-enriched | Alphaproteobacteria | Tistlia |
| DQ167235 | OTU1500 | binomial_p=0.169591, ranksum_p=0.002361 | Sponge-enriched | Alphaproteobacteria | Tistlia |
| DQ869302 | OTU1500 | binomial_p=0.169591, ranksum_p=0.002361 | Sponge-enriched | Alphaproteobacteria | Tistlia |
| EU346389 | OTU15073 | binomial_p=0.253078, ranksum_p=0.027941 | Sponge-enriched | Alphaproteobacteria | Unclassified |
| EU346513 | OTU15073 | binomial_p=0.253078, ranksum_p=0.027941 | Sponge-enriched | Alphaproteobacteria | Unclassified |
| EU346590 | OTU15073 | binomial_p=0.253078, ranksum_p=0.027941 | Sponge-enriched | Alphaproteobacteria | Unclassified |
| EU346639 | OTU15073 | binomial_p=0.253078, ranksum_p=0.027941 | Sponge-enriched | Alphaproteobacteria | Unclassified |
| KP684322 | OTU15955 | binomial_p=0.290131, ranksum_p=0.059546 | Sponge-enriched | Bacteroidetes | Unclassified |
| AY371442 | OTU17006 | binomial_p=0.183246, ranksum_p=0.003901 | Sponge-enriched | Gammaproteobacteria | Endozoicomonas |
| HE818343 | OTU17006 | binomial_p=0.183246, ranksum_p=0.003901 | Sponge-enriched | Gammaproteobacteria | Endozoicomonas |
| AY370006 | OTU17751 | binomial_p=0.246630, ranksum_p=0.024079 | Sponge-enriched | Gammaproteobacteria | Endozoicomonas |
| HE818219 | OTU1905 | binomial_p=0.011580, ranksum_p=0.000000 | Sponge-enriched | Gammaproteobacteria | Endozoicomonas |
| HE818335 | OTU1905 | binomial_p=0.011580, ranksum_p=0.000000 | Sponge-enriched | Gammaproteobacteria | Endozoicomonas |
| KJ573540 | OTU1905 | binomial_p=0.011580, ranksum_p=0.000000 | Sponge-enriched | Gammaproteobacteria | Endozoicomonas |
| EF040542 | OTU3421 | binomial_p=0.202217, ranksum_p=0.007663 | Sponge-enriched | Bacteroidetes | Flagellimonas |
| EF040552 | OTU3421 | binomial_p=0.202217, ranksum_p=0.007663 | Sponge-enriched | Bacteroidetes | Flagellimonas |
| EF040559 | OTU3421 | binomial_p=0.202217, ranksum_p=0.007663 | Sponge-enriched | Bacteroidetes | Flagellimonas |
| EF040561 | OTU3421 | binomial_p=0.202217, ranksum_p=0.007663 | Sponge-enriched | Bacteroidetes | Flagellimonas |
| DQ994722 | OTU3788 | binomial_p=0.104632, ranksum_p=0.000087 | Sponge-enriched | Acidobacteria | Unclassified |
| KP684366 | OTU4055 | binomial_p=0.312694, ranksum_p=0.087929 | Sponge-enriched | Bacteroidetes | Unclassified |
| EU199233 | OTU4651 | binomial_p=0.231725, ranksum_p=0.021053 | Sponge-enriched | Gammaproteobacteria | Klebsiella |
| FJ596415 | OTU4651 | binomial_p=0.231725, ranksum_p=0.021053 | Sponge-enriched | Gammaproteobacteria | Enterobacter |
| FJ596544 | OTU4651 | binomial_p=0.231725, ranksum_p=0.021053 | Sponge-enriched | Gammaproteobacteria | Enterobacter |
| KF582898 | OTU4651 | binomial_p=0.231725, ranksum_p=0.021053 | Sponge-enriched | Gammaproteobacteria | Citrobacter |
| KF582900 | OTU4651 | binomial_p=0.231725, ranksum_p=0.021053 | Sponge-enriched | Gammaproteobacteria | Citrobacter |
| KF582901 | OTU4651 | binomial_p=0.231725, ranksum_p=0.021053 | Sponge-enriched | Gammaproteobacteria | Citrobacter |
| KF582903 | OTU4651 | binomial_p=0.231725, ranksum_p=0.021053 | Sponge-enriched | Gammaproteobacteria | Citrobacter |
| KF582904 | OTU4651 | binomial_p=0.231725, ranksum_p=0.021053 | Sponge-enriched | Gammaproteobacteria | Citrobacter |
| KF582905 | OTU4651 | binomial_p=0.231725, ranksum_p=0.021053 | Sponge-enriched | Gammaproteobacteria | Unclassified |
| KF582906 | OTU4651 | binomial_p=0.231725, ranksum_p=0.021053 | Sponge-enriched | Gammaproteobacteria | Kluyvera |
| AY371441 | OTU5132 | binomial_p=0.144449, ranksum_p=0.000960 | Sponge-enriched | Gammaproteobacteria | Endozoicomonas |
| KC854345 | OTU5132 | binomial_p=0.144449, ranksum_p=0.000960 | Sponge-enriched | Gammaproteobacteria | Endozoicomonas |
| AY371432 | OTU5789 | binomial_p=0.158666, ranksum_p=0.001637 | Sponge-enriched | Gammaproteobacteria | Unclassified |
| DQ180743 | OTU5891 | binomial_p=0.103132, ranksum_p=0.000077 | Sponge-enriched | Gammaproteobacteria | Shewanella |
| HE818163 | OTU5891 | binomial_p=0.103132, ranksum_p=0.000077 | Sponge-enriched | Gammaproteobacteria | Shewanella |
| HE818167 | OTU5891 | binomial_p=0.103132, ranksum_p=0.000077 | Sponge-enriched | Gammaproteobacteria | Shewanella |
| HE818174 | OTU5891 | binomial_p=0.103132, ranksum_p=0.000077 | Sponge-enriched | Gammaproteobacteria | Shewanella |
| HE818216 | OTU5891 | binomial_p=0.103132, ranksum_p=0.000077 | Sponge-enriched | Gammaproteobacteria | Shewanella |
| KP684297 | OTU5891 | binomial_p=0.103132, ranksum_p=0.000077 | Sponge-enriched | Gammaproteobacteria | Shewanella |
| KP684406 | OTU5891 | binomial_p=0.103132, ranksum_p=0.000077 | Sponge-enriched | Gammaproteobacteria | Shewanella |
| KP684413 | OTU5891 | binomial_p=0.103132, ranksum_p=0.000077 | Sponge-enriched | Gammaproteobacteria | Shewanella |
| AB205011 | OTU6081 | binomial_p=0.060665, ranksum_p=0.000002 | Sponge-enriched | Gammaproteobacteria | Endozoicomonas |
| FJ215562 | OTU6081 | binomial_p=0.060665, ranksum_p=0.000002 | Sponge-enriched | Gammaproteobacteria | Endozoicomonas |
| FJ215566 | OTU6081 | binomial_p=0.060665, ranksum_p=0.000002 | Sponge-enriched | Gammaproteobacteria | Endozoicomonas |
| KF282425 | OTU6081 | binomial_p=0.060665, ranksum_p=0.000002 | Sponge-enriched | Gammaproteobacteria | Endozoicomonas |
| AY367748 | OTU6229 | binomial_p=0.299246, ranksum_p=0.082665 | Sponge-enriched | Actinobacteria | Corynebacterium_1 |
| AY371431 | OTU6659 | binomial_p=0.274222, ranksum_p=0.043864 | Sponge-enriched | Gammaproteobacteria | Paraferrimonas |
| KJ372433 | OTU6659 | binomial_p=0.274222, ranksum_p=0.043864 | Sponge-enriched | Gammaproteobacteria | Shewanella |
| KJ372435 | OTU6659 | binomial_p=0.274222, ranksum_p=0.043864 | Sponge-enriched | Gammaproteobacteria | Shewanella |
| KJ372436 | OTU6659 | binomial_p=0.274222, ranksum_p=0.043864 | Sponge-enriched | Gammaproteobacteria | Shewanella |
| KJ372437 | OTU6659 | binomial_p=0.274222, ranksum_p=0.043864 | Sponge-enriched | Gammaproteobacteria | Shewanella |
| KJ372438 | OTU6659 | binomial_p=0.274222, ranksum_p=0.043864 | Sponge-enriched | Gammaproteobacteria | Shewanella |
| KJ372440 | OTU6659 | binomial_p=0.274222, ranksum_p=0.043864 | Sponge-enriched | Gammaproteobacteria | Unclassified |
| KJ372441 | OTU6659 | binomial_p=0.274222, ranksum_p=0.043864 | Sponge-enriched | Gammaproteobacteria | Shewanella |
| KJ372442 | OTU6659 | binomial_p=0.274222, ranksum_p=0.043864 | Sponge-enriched | Gammaproteobacteria | Shewanella |
| KJ372443 | OTU6659 | binomial_p=0.274222, ranksum_p=0.043864 | Sponge-enriched | Gammaproteobacteria | Shewanella |
| KJ372444 | OTU6659 | binomial_p=0.274222, ranksum_p=0.043864 | Sponge-enriched | Gammaproteobacteria | Shewanella |
| KJ372445 | OTU6659 | binomial_p=0.274222, ranksum_p=0.043864 | Sponge-enriched | Gammaproteobacteria | Shewanella |
| KJ372446 | OTU6659 | binomial_p=0.274222, ranksum_p=0.043864 | Sponge-enriched | Gammaproteobacteria | Shewanella |
| KJ372447 | OTU6659 | binomial_p=0.274222, ranksum_p=0.043864 | Sponge-enriched | Gammaproteobacteria | Shewanella |
| KJ372448 | OTU6659 | binomial_p=0.274222, ranksum_p=0.043864 | Sponge-enriched | Gammaproteobacteria | Shewanella |
